# Global assessment of marine plastic exposure risk for oceanic birds

**DOI:** 10.1038/s41467-023-38900-z

**Published:** 2023-07-04

**Authors:** Bethany L. Clark, Ana P. B. Carneiro, Elizabeth J. Pearmain, Marie-Morgane Rouyer, Thomas A. Clay, Win Cowger, Richard A. Phillips, Andrea Manica, Carolina Hazin, Marcus Eriksen, Jacob González-Solís, Josh Adams, Yuri V. Albores-Barajas, Joanna Alfaro-Shigueto, Maria Saldanha Alho, Deusa Teixeira Araujo, José Manuel Arcos, John P. Y. Arnould, Nadito J. P. Barbosa, Christophe Barbraud, Annalea M. Beard, Jessie Beck, Elizabeth A. Bell, Della G. Bennet, Maud Berlincourt, Manuel Biscoito, Oskar K. Bjørnstad, Mark Bolton, Katherine A. Booth Jones, John J. Borg, Karen Bourgeois, Vincent Bretagnolle, Joël Bried, James V. Briskie, M. de L. Brooke, Katherine C. Brownlie, Leandro Bugoni, Licia Calabrese, Letizia Campioni, Mark J. Carey, Ryan D. Carle, Nicholas Carlile, Ana R. Carreiro, Paulo Catry, Teresa Catry, Jacopo G. Cecere, Filipe R. Ceia, Yves Cherel, Chang-Yong Choi, Marco Cianchetti-Benedetti, Rohan H. Clarke, Jaimie B. Cleeland, Valentina Colodro, Bradley C. Congdon, Jóhannis Danielsen, Federico De Pascalis, Zoe Deakin, Nina Dehnhard, Giacomo Dell’Omo, Karine Delord, Sébastien Descamps, Ben J. Dilley, Herculano A. Dinis, Jerome Dubos, Brendon J. Dunphy, Louise M. Emmerson, Ana Isabel Fagundes, Annette L. Fayet, Jonathan J. Felis, Johannes H. Fischer, Amanda N. D. Freeman, Aymeric Fromant, Giorgia Gaibani, David García, Carina Gjerdrum, Ivandra Soeli Gonçalves Correia Gomes, Manuela G. Forero, José P. Granadeiro, W. James Grecian, David Grémillet, Tim Guilford, Gunnar Thor Hallgrimsson, Luke R. Halpin, Erpur Snær Hansen, April Hedd, Morten Helberg, Halfdan H. Helgason, Leeann M. Henry, Hannah F. R. Hereward, Marcos Hernandez-Montero, Mark A. Hindell, Peter J. Hodum, Simona Imperio, Audrey Jaeger, Mark Jessopp, Patrick G. R. Jodice, Carl G. Jones, Christopher W. Jones, Jón Einar Jónsson, Adam Kane, Sven Kapelj, Yuna Kim, Holly Kirk, Yann Kolbeinsson, Philipp L. Kraemer, Lucas Krüger, Paulo Lago, Todd J. Landers, Jennifer L. Lavers, Matthieu Le Corre, Andreia Leal, Maite Louzao, Jeremy Madeiros, Maria Magalhães, Mark L. Mallory, Juan F. Masello, Bruno Massa, Sakiko Matsumoto, Fiona McDuie, Laura McFarlane Tranquilla, Fernando Medrano, Benjamin J. Metzger, Teresa Militão, William A. Montevecchi, Rosalinda C. Montone, Leia Navarro-Herrero, Verónica C. Neves, David G. Nicholls, Malcolm A. C. Nicoll, Ken Norris, Steffen Oppel, Daniel Oro, Ellie Owen, Oliver Padget, Vítor H. Paiva, David Pala, Jorge M. Pereira, Clara Péron, Maria V. Petry, Admilton de Pina, Ariete T. Moreira Pina, Patrick Pinet, Pierre A. Pistorius, Ingrid L. Pollet, Benjamin J. Porter, Timothée A. Poupart, Christopher D. L. Powell, Carolina B. Proaño, Júlia Pujol-Casado, Petra Quillfeldt, John L. Quinn, Andre F. Raine, Helen Raine, Iván Ramírez, Jaime A. Ramos, Raül Ramos, Andreas Ravache, Matt J. Rayner, Timothy A. Reid, Gregory J. Robertson, Gerard J. Rocamora, Dominic P. Rollinson, Robert A. Ronconi, Andreu Rotger, Diego Rubolini, Kevin Ruhomaun, Asunción Ruiz, James C. Russell, Peter G. Ryan, Sarah Saldanha, Ana Sanz-Aguilar, Mariona Sardà-Serra, Yvan G. Satgé, Katsufumi Sato, Wiebke C. Schäfer, Stefan Schoombie, Scott A. Shaffer, Nirmal Shah, Akiko Shoji, Dave Shutler, Ingvar A. Sigurðsson, Mónica C. Silva, Alison E. Small, Cecilia Soldatini, Hallvard Strøm, Christopher A. Surman, Akinori Takahashi, Vikash R. V. Tatayah, Graeme A. Taylor, Robert J. Thomas, David R. Thompson, Paul M. Thompson, Thorkell L. Thórarinsson, Diego Vicente-Sastre, Eric Vidal, Ewan D. Wakefield, Susan M. Waugh, Henri Weimerskirch, Heiko U. Wittmer, Takashi Yamamoto, Ken Yoda, Carlos B. Zavalaga, Francis J. Zino, Maria P. Dias

**Affiliations:** 1 BirdLife International, Cambridge, UK; 2 Department of Zoology, University of Cambridge, Cambridge, UK; 3 British Antarctic Survey, Natural Environment Research Council, Cambridge, UK; 4 CEFE, Univ Montpellier, CNRS, EPHE, IRD, Montpellier, France; 5 Institute of Marine Sciences, University of California Santa Cruz, Santa Cruz, CA USA; 6 People and Nature, Environmental Defense Fund, Monterey, CA USA; 7 School of Environmental Sciences, University of Liverpool, Liverpool, UK; 8 University of California, Riverside, CA USA; 9 The Nature Conservancy, London, UK; 10 5 Gyres Institute, Los Angeles, CA USA; 11 Institut de Recerca de la Biodiversitat (IRBio), Universitat de Barcelona, Barcelona, Spain; 12 Departament de Biologia Evolutiva, Ecologia i Ciències Ambientals, Universitat de Barcelona, Barcelona, Spain; 13 U.S. Geological Survey, Western Ecological Research Center, Santa Cruz Field Station, Santa Cruz, CA USA; 14 Universidad Autonoma de Baja California Sur - UABCS, La Paz, Mexico; 15 Consejo Nacional de Ciencia y Tecnología (CONACYT), Mexico City, Mexico; 16 Carrera de Biologia Marina, Universidad Cientifica del Sur, Lima, Peru; 17 ProDelphinus, Lima, Peru; 18 University of Exeter, School of Biosciences, Cornwall Campus, Exeter, UK; 19 MARE - Marine and Environmental Sciences Centre/ARNET - Aquatic Research Network, Ispa - Instituto Universitário, Lisbon, Portugal; 20 Associação Projecto Vitó, São Filipe, Cabo Verde; 21 SEO/BirdLife, Barcelona, Spain; 22 Deakin University, Burwood, VIC Australia; 23 Centre d’Etudes Biologiques de Chizé (CEBC), UMR 7372 du CNRS-La Rochelle Université, Villiers-en-Bois, France; 24 St. Helena Government, Jamestown, St. Helena, UK; 25 Cardiff University, Cardiff, UK; 26 Oikonos Ecosystem Knowledge, Santa Cruz, CA USA; 27 Wildlife Management International Ltd, Blenheim, New Zealand; 28 School of Biological Sciences, University of Canterbury, Christchurch, New Zealand; 29 Marine and Environmental Sciences Centre (MARE), Museu de História Natural do Funchal, Funchal, Portugal; 30 Skudeneshavn, Norway; 31 RSPB Centre for Conservation Science, Aberdeen, UK; 32 British Trust for Ornithology, Belfast, UK; 33 National Museum of Natural History, Mdina, Malta; 34 3 Institut Méditerranéen de Biodiversité et d’Ecologie marine et continentale (IMBE), Aix Marseille Université, CNRS, IRD, Avignon Université, Nouméa, New Caledonia France; 35 School of Biological Sciences, University of Auckland, Auckland, New Zealand; 36 Institute of Marine Sciences - OKEANOS, University of the Azores, 9901-862 Horta, Portugal; 37 Universidade Federal do Rio Grande - FURG, Rio Grande, Brazil; 38 Island Conservation Society, Mahé, Seychelles; 39 Université Pierre et Marie Curie, Paris, France; 40 Island Biodiversity and Conservation Centre, University of Seychelles, Anse Royale, Seychelles; 41 Department of Environmental Management and Ecology, La Trobe University, Wodonga, NSW Australia; 42 Science, Economics and Insights Division, Department of Planning and Environment, Sydney, Australia; 43 University of Coimbra, MARE - Marine and Environmental Sciences Centre/ARNET - Aquatic Research Network, Department of Life Sciences, Coimbra, Portugal; 44 CIBIO, Centro de Investigação em Biodiversidade e Recursos Genéticos, InBIO Laboratório Associado, Campus Agrário de Vairão, Fornelo e Vairão, Portugal; 45 CESAM - Centro de Estudos do Ambiente e do Mar, Departamento de Biologia Animal, Faculdade de Ciências, Universidade de Lisboa, Lisbon, Portugal; 46 Area Avifauna Migratrice, Istituto Superiore per la Protezione e la Ricerca Ambientale (ISPRA), Ozzano dell’Emilia, Italy; 47 Department of Agriculture, Forestry, and Bioresources, Seoul National University, Seoul, South Korea; 48 Ornis Italica, Rome, Italy; 49 School of Biological Sciences, Monash University, Melbourne, VIC Australia; 50 Institute for Marine and Antarctic Studies, University of Tasmania, Hobart, Australia; 51 Australian Antarctic Division, Kingston, TAS Australia; 52 Oikonos Ecosystem Knowledge, Valparaiso, Chile; 53 College of Science and Engineering, James Cook University, Cairns, Australia; 54 Faroe Marine Research Institute, Tórshavn, Faroe Islands; 55 Department of Environmental Science and Policy, University of Milan, Milan, Italy; 56 RSPB Centre for Conservation Science, Cambridge, UK; 57 Norwegian Institute for Nature Research (NINA), Trondheim, Norway; 58 Department of Biology, Behavioural Ecology and Ecophysiology Group, University of Antwerp, Antwerp, Belgium; 59 Norwegian Polar Institute, Tromsø, Norway; 60 FitzPatrick Institute of African Ornithology, DST-NRF Centre of Excellence, University of Cape Town, Cape Town, South Africa; 61 UMR ENTROPIE, Université de la Réunion, Saint-Denis, Réunion France; 62 Institute of Marine Sciences/School of Biological Sciences, University of Auckland, Auckland, New Zealand; 63 SPEA, Lisbon, Portugal; 64 Department of Biology, University of Oxford, Oxford, UK; 65 United States Geological Survey, Santa Cruz, CA USA; 66 Aquatic Unit, Department of Conservation, Wellington, New Zealand; 67 Nature North, Malanda, QLD Australia; 68 LIPU-BirdLife Italy, Parma, Italy; 69 Iniciativa de Recerca de la Biodiversitat de les Illes (IRBI), Pina, Spain; 70 Canadian Wildlife Service, Environment and Climate Change Canada, Dartmouth, Nova Scotia Canada; 71 Departamento de Biología de la Conservación, Estación Biológica de Doñana (EBD), Consejo Superior de Investigaciones Científicas (CSIC), Sevilla, Spain; 72 Departamento de Biologia Animal, Faculdade de Ciências, Universidade de Lisboa & CESAM - Centre for Environmental and Marine Studies, Lisboa, Portugal; 73 Department of Geography, Durham University, Durham, UK; 74 Department of Life and Environmental Sciences, University of Iceland, Reykjavík, Iceland; 75 Monash University, Clayton, VIC Australia; 76 Halpin Wildlife Research, Vancouver, BC Canada; 77 South Iceland Nature Research Centre, Vestmannaeyjar, Iceland; 78 Wildlife Research Division, Environment and Climate Change Canada, Mount Pearl, NC Canada; 79 Østfold University College, Halden, Norway; 80 BirdLife Norway, Sandgata 30 B, 7012 Trondheim, Norway; 81 East Iceland Nature Research Centre, Egilsstaðir, Iceland; 82 British Trust for Ornithology Cymru, Thoday Building, Deiniol Road, Bangor, Wales UK; 83 Associação Projeto Biodiversidade, Santa Maria, Ilha do Sal Cabo Verde; 84 Oikonos Ecosystem Knowledge, Tacoma, WA USA; 85 Institute of Geosciences and Earth Resources, CNR, Pisa, Italy; 86 School of Biological, Earth & Environmental Sciences, University College Cork, Cork, Ireland; 87 MaREI Centre, Environmental Research Institute, University College Cork, Cork, Ireland; 88 U.S. Geological Survey South Carolina Cooperative Fish and Wildlife Research Unit, Clemson University, Clemson, SC USA; 89 Mauritian Wildlife Foundation, Vacoas, Mauritius; 90 Durrell Wildlife Conservation Trust, Trinity, Jersey; 91 University of Iceland’s Research Center at Snæfellsnes, Stykkishólmur, Iceland; 92 University College Dublin, Dublin, Ireland; 93 Association Biom, Zagreb, Croatia; 94 Macquarie University, Sydney, Australia; 95 RMIT University, Melbourne, Australia; 96 Northeast Iceland Nature Research Centre, Húsavík, Iceland; 97 Department of Animal Ecology and Systematics, Justus Liebig University Giessen, Giessen, Germany; 98 Instituto Antártico Chileno, Punta Arenas, Chile; 99 Instituto Milénio Biodiversidad de Ecosistemas Antárticos y Subantárticos (BASE), Santiago, Chile; 100 BirdLife Malta, Ta’ Xbiex, Malta; 101 Auckland Council, Auckland, New Zealand; 102 Tjaltjraak Native Title Aboriginal Corporation, Esperance, WA Australia; 103 AZTI, Pasaia, Spain; 104 Dept. of Environment and Natural Resources, Bermuda Government, Flatts, Bermuda; 105 Regional Directorate for Marine Policies, Azores Government, Horta, Azores Portugal; 106 Acadia University, Wolfville, NS Canada; 107 Department of Agriculture, Food and Forest Sciences, University of Palermo, Palermo, Italy; 108 Nagoya University, Nagoya, Japan; 109 San Jose State University Research Foundation, San Jose, CA USA; 110 Birds Canada, Sackville, NB Canada; 111 Memorial University of Newfoundland and Labrador, St. John’s, Canada; 112 Universidade de São Paulo - Instituto Oceanográfico, São Paulo, Brazil; 113 IMAR Instituto do Mar, Universidade dos Açores, Horta, Portugal; 114 Chisholm Institute, Dandenong, Australia; 115 Institute of Zoology, Zoological Society of London, London, UK; 116 Natural History Museum, London, UK; 117 CEAB-CSIC, Centre d’Estudis Avançats de Blanes, Blanes, Spain; 118 RSPB Centre for Conservation Science, Inverness, UK; 119 The National Trust for Scotland, Balnain House, Huntly Street, Inverness, UK; 120 Parco naturale Regionale di Porto Conte, Alghero, Italy; 121 Laboratoire de Biologie des Organismes et Ecosystèmes Aquatiques (UMR BOREA) - Muséum national d’Histoire Naturelle (MNHN), CNRS, IRD, SU, UCN, UA, Paris, France; 122 Universidade do Vale do Rio dos Sinos - UNISINOS, São Leopoldo, Brazil; 123 Project Vitó Association, Praia, Cabo Verde; 124 Université de La Réunion, Saint-Denis, Réunion France; 125 Marine Apex Predator Research Unit (MAPRU), Department of Zoology, Institute for Coastal and Marine Research, Nelson Mandela University, Port Elizabeth, South Africa; 126 Albany, WA Australia; 127 Max Planck Institute for Ornithology, Puerto Ayora, Galapagos Islands Ecuador; 128 School of BEES, University College Cork, Cork, Ireland; 129 Archipelago Research and Conservation, Kalaheo, HI USA; 130 Convention on Migratory Species (CMS), Bonn, Germany; 131 UMR ENTROPIE (IRD, Université de La Réunion, CNRS, Université de La Nouvelle-Calédonie, Ifremer), Centre IRD Nouméa, Nouméa, New Caledonia France; 132 Auckland War Memorial Museum, Auckland, New Zealand; 133 CSIRO Oceans and Atmosphere, Hobart, NSW Australia; 134 Environment and Climate Change Canada, Mount Pearl, NL Canada; 135 Animal Demography and Ecology Unit (GEDA), IMEDEA (CSIC-UIB), Esporles, Spain; 136 Dipartimento di Scienze e Politiche Ambientali, Università degli Studi di Milano, Milano, Italy; 137 Istituto di Ricerca sulle Acque - Consiglio Nazionale delle Ricerche (IRSA-CNR), Brugherio, Italy; 138 National Parks and Parks Conservation Service, Reduit, Mauritius; 139 SEO/BirdLife, Madrid, Spain; 140 University of Balearic Islands, Palma, Spain; 141 Department of Forestry and Environmental Conservation, Clemson University, Clemson, SC USA; 142 Atmosphere and Ocean Research Institute, University of Tokyo, Kashiwa City, Japan; 143 Biological Sciences, San Jose State University, San Jose, CA USA; 144 Nature Seychelles, Mahé, Seychelles; 145 University of Tsukuba, Tsukuba, Japan; 146 Icelandic Institute of Natural History, Garðabær, Iceland; 147 cE3c - Centre for Ecology, Evolution and Evolutionary Changes, Faculdade de Ciências, Universidade de Lisboa, Lisboa, Portugal; 148 CICESE - Centro de Investigación Científica y de Educación Superior de Ensenada - Unidad La Paz, La Paz, Mexico; 149 Halfmoon Biosciences, Denmark, Australia; 150 National Institute of Polar Research, Tachikawa, Japan; 151 Department of Conservation, Wellington, New Zealand; 152 National Institute of Water and Atmospheric Research Ltd, Wellington, New Zealand; 153 University of Aberdeen, Cromarty, UK; 154 UMR ENTROPIE (IRD, UR, UNC, CNRS, IFREMER), Nouméa, New Caledonia France; 155 UMR IMBE (IRD, AMU, CNRS, UAPV), Nouméa, France; 156 School of Biological Sciences, Victoria University of Wellington, Wellington, New Zealand; 157 Azabu University, Kanagawa, Japan; 158 Graduate School of Environmental Studies, Nagoya University, Nagoya, Japan; 159 Universidad Cientifica del Sur, Lima, Peru; 160 Freira Conservation Project, Funchal, Madeira Portugal; 161 CHANGE - Global Change and Sustainability Institute, Departamento de Biologia Animal, Faculdade de Ciências, Universidade de Lisboa, Lisboa, Portugal

**Keywords:** Marine biology, Animal migration, Conservation biology

## Abstract

Plastic pollution is distributed patchily around the world’s oceans. Likewise, marine organisms that are vulnerable to plastic ingestion or entanglement have uneven distributions. Understanding where wildlife encounters plastic is crucial for targeting research and mitigation. Oceanic seabirds, particularly petrels, frequently ingest plastic, are highly threatened, and cover vast distances during foraging and migration. However, the spatial overlap between petrels and plastics is poorly understood. Here we combine marine plastic density estimates with individual movement data for 7137 birds of 77 petrel species to estimate relative exposure risk. We identify high exposure risk areas in the Mediterranean and Black seas, and the northeast Pacific, northwest Pacific, South Atlantic and southwest Indian oceans. Plastic exposure risk varies greatly among species and populations, and between breeding and non-breeding seasons. Exposure risk is disproportionately high for Threatened species. Outside the Mediterranean and Black seas, exposure risk is highest in the high seas and Exclusive Economic Zones (EEZs) of the USA, Japan, and the UK. Birds generally had higher plastic exposure risk outside the EEZ of the country where they breed. We identify conservation and research priorities, and highlight that international collaboration is key to addressing the impacts of marine plastic on wide-ranging species.

## Abstract (web summary)

Petrels are wide-ranging, highly threatened seabirds that often ingest plastic. This study used tracking data for 7,137 petrels of 77 species to map global exposure risk and compare regions, species, and populations. The results show higher exposure risk for threatened species and stress the need for international cooperation to tackle marine litter.

## Introduction

Plastic pollution harms marine life worldwide^1^, alongside other threats including fishing, climate change and invasive species^2^. Reports of entanglement and ingestion impacts are mounting^1,3,4^, but there are large gaps in our understanding, including about factors affecting plastic encounter, ingestion rates, mortality and population-level impacts^4,5^. Marine plastic is unevenly distributed^6^, accumulating in patches within ocean gyres and coastal regions^7,8^, and often drifting thousands of kilometres in ocean currents^8,9^. Likewise, marine life is patchily distributed^10^, and many species cross oceans and political boundaries^11,12^. With plastic production and waste generation continuing to increase^13^, identifying at-risk species and populations is crucial for targeting conservation action and research^14–16^ because the vulnerability of populations relates to exposure to a hazard, sensitivity to damage that impacts survival or reproduction, and the resilience of the population^17^.

Many seabird species are sensitive to plastic pollution; they frequently ingest plastic^1^, which can have lethal and sublethal impacts caused by chemical contamination^18^ and physical damage or blockages^19^. Numerous factors affect the amount of plastic accumulated by different species including foraging behaviour, at-sea distribution and gut morphology^20–22^. Among seabirds, albatrosses and petrels can contain particularly high loads of plastic ingested directly or within their prey^1,20^. Many species rarely regurgitate indigestible items, except when feeding their chicks^23^. Petrels are particularly sensitive because they retain plastic for long periods due to their gut morphology^22^, and small species (e.g., storm-petrels and gadfly petrels) can suffer greater physical damage or higher metabolic costs from ingesting plastic relative to larger species^5^. Petrels are a diverse group of 123 wide-ranging species that inhabit all the world’s oceans, making them good sentinels for ocean health^2^. Many populations are unlikely to be resilient to hazards because over half (64) are listed as globally Threatened or Near Threatened by the International Union for the Conservation of Nature (IUCN), including 16 Endangered and 12 Critically Endangered species^2^. Moreover, we know little about the status of many of their populations or if they are impacted by plastic^2^.

Assessing risk to petrel populations from plastic pollution requires a robust understanding of vulnerability to ingestion, for which exposure at sea is a key component^14^. Seabirds risk encountering plastic when they forage near sources associated with dense human populations^24^, fisheries^25^ and shipping lanes^26^, or in mid-ocean gyres where floating debris accumulates^27–29^. Exposure risk can be characterised by estimating contact between organisms and hazards, or their co-occurrence, and a key goal in ecological risk assessment is to consider variation in the amount of time spent by animals in different parts of their range^30,31^. Plastic exposure risk has not been previously quantified using methods that account for the time spent in areas of different densities of plastic pollution, but lightweight tracking devices have recently provided unprecedented detail about the movements of petrels of all sizes^32^, including the time spent in different foraging areas and across the annual cycle^33^.

Here, we estimate relative marine plastic exposure risk for 77 petrel species at a global scale by calculating the spatio-temporal overlap between modelled floating plastic density and the space-use of tracked birds^14^. To inform conservation action and future research, we compare exposure risk across populations, seasons (breeding and non-breeding), Exclusive Economic Zones (EEZ) and areas beyond national jurisdiction (the high seas), and found substantial variation. We identified areas of high risk of exposure to plastic debris in the Mediterranean and Black seas, the northeast Pacific, the northwest Pacific, the South Atlantic and the southwest Indian Ocean. Our results also reveal that Threatened species have greater exposure risk. Because marine debris and seabirds cross multiple political boundaries, our results emphasise that efforts to reduce the amount of plastic waste in the ocean should not only focus on areas of high exposure risk. Improved international cooperation and collaboration are needed to address this global threat.

## Results and discussion

### Plastic exposure risk for petrels

**Fig. 1:**
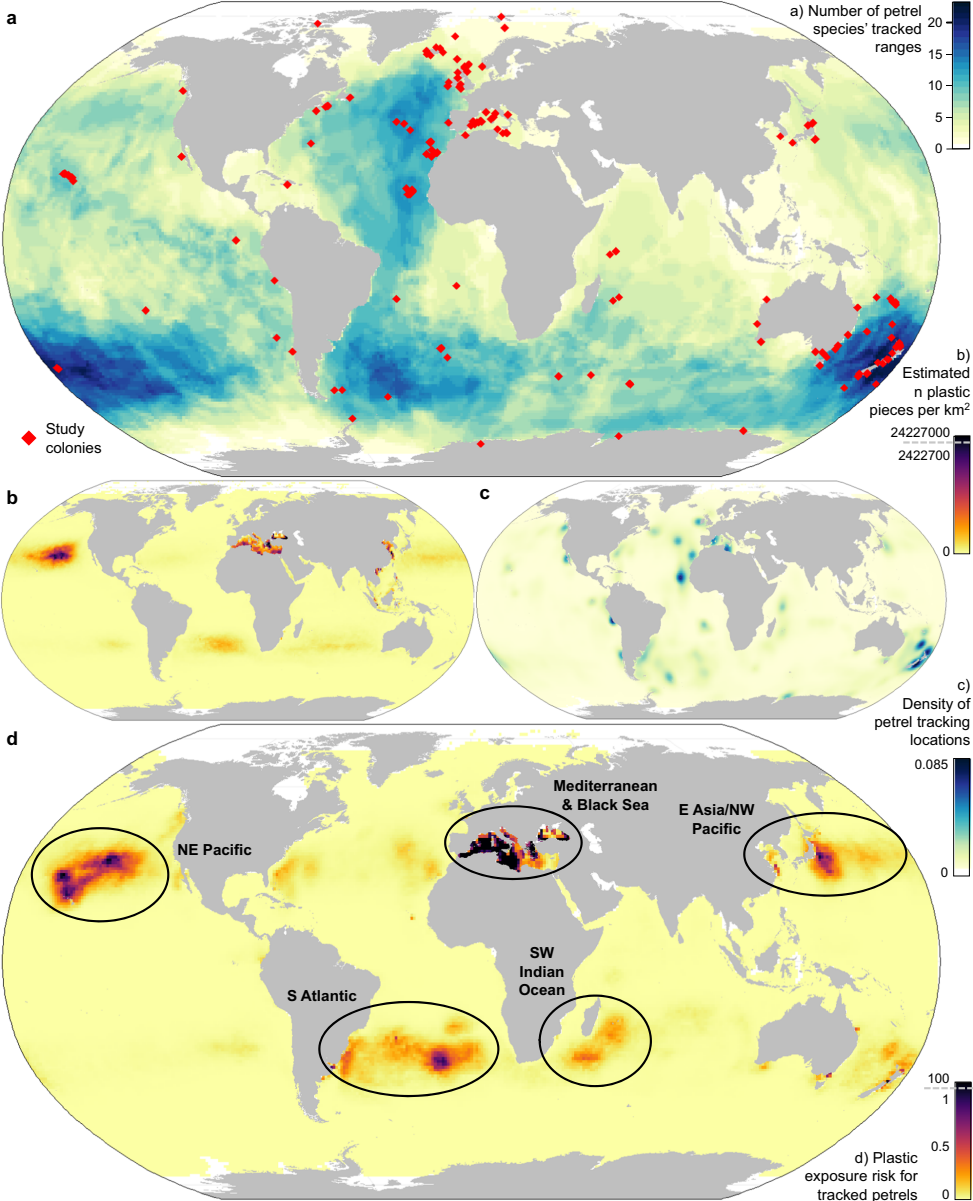
Mapping petrels and plastics. **a** Species richness based on presence within 95% utilisation distributions isopleth contours from tracking data for 77 petrel species. Red diamonds indicate the colonies from which tracking data were obtained. **b** Plastic density at the ocean surface, showing the square root of the number of plastic pieces (~0.333 mm–0.4 m) estimated per km^2^ in each 1 × 1° grid cell. For visualisation only, the values are capped at 10% due to extreme values. **c** Summed 95% utilisation distributions for all species, with species weighted equally if year-round tracks were available or by 0.5 if tracks were only available for the breeding season. If we had data from multiple populations for a species, densities were weighted by approximate population size. **d** Exposure risk to plastic was calculated by multiplying the density value in each cell for plastics (scaled to sum to 1) by the value for petrels (scaled to sum to 1). For visualisation only, the values are capped at 1% due to extreme values, and all other values are shown on a linear scale. Black ellipses relate to the areas identified from the 20 species with the highest exposure risk scores (Fig. 2a). *n* = number. White = no data. Robinson Projection. Land polygons from Natural Earth. Source data for colony locations are provided as a Source Data file.

We analysed 1,736,880 tracked locations for 7137 adults of 77 petrel species (64% of species within Oceanitidae, Hydrobatidae and Procellariidae, excluding the two *Macronectes* species), from 148 populations in 27 countries and Antarctica, between 1995 and 2020 (mean = 2012). For each population, we calculated monthly 95% utilisation distributions (UDs) that estimate time spent by tracked petrels in 10 km grid cells (i.e., smoothed density of 12-hourly tracked locations; Fig. 1a), and combined monthly UDs into seasons (breeding or non-breeding). If data were available from multiple populations of a species, we created species UDs weighted by approximate population size. We calculated a geometric mean of global marine plastic densities estimated by three published models^6,9,34^ for micro- and macro-plastics (~0.333 mm–40 cm) combined for 2014 in 1 × 1° cells (Fig. 1b). We aggregated petrel UDs into 1 × 1° grid cells and created an all-species map by summing species UDs, weighting those tracked only in the breeding season and so not including the non-breeding part of the annual cycle at 0.5 (Fig. 1c). We divided the plastic and petrel grids by their respective cumulative sums so that the values of each global grid summed to one. We then multiplied each petrel UD by the plastic density to map spatial overlap as an indicator of estimated exposure risk^14^ (e.g., Figure 1d). Summing the values across cells provided an exposure risk score, which we multiplied by 10^6^ to provide an easy-to-use scale; this gave us monthly population-level scores ranging from 0.0007 to 1091.

**Fig. 2:**
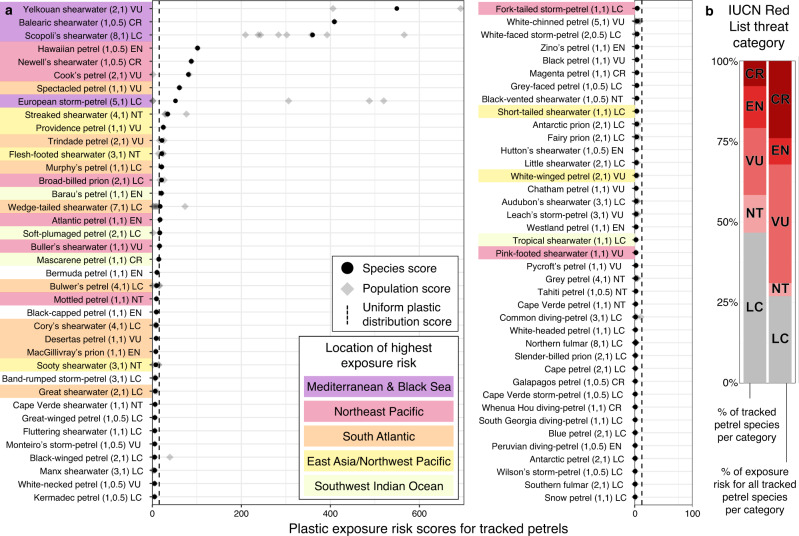
Plastic exposure risk scores for 77 petrel species. **a** Species are ranked by exposure risk from the top-left to the bottom-right. Colours represent the location that contributed most to the score for the five areas of highest exposure risk. Where there are multiple populations per species (grey diamonds), the mean of all populations (black circles) is weighted by the population size. The vertical dashed line indicates the theoretical exposure risk score if plastic was uniformly distributed across all cells (15.3). Values in parentheses are the number of populations, followed by 1 if the species was tracked in breeding and non-breeding seasons or by 0.5 if only tracked in one season. Two-letter codes indicate the IUCN Red List assessment threat category (Least Concern (LC; *n* = 36), Near Threatened (NT; 9), Vulnerable (VU; 16), Endangered (EN; 10), Critically Endangered (CR; 6)). **b** The percentage of tracked petrel species within each IUCN threat category and the percentage of total exposure risk attributed to species in each category. Source data are provided as a Source Data file.

We ranked species by plastic exposure risk score (Fig. 2a), ranging from 0.003 to 549 (mean = 28.0; median = 4.9, interquartile range = 1.8–14.5). Of particular concern are the 19 species scoring over 15.3 (the score any species would receive if plastic was evenly distributed worldwide), indicating they mostly use areas with above-average plastic density. These species include the Critically Endangered Balearic shearwater *Puffinus mauretanicus* and Newell’s shearwater *Puffinus newelli*; the Endangered Hawaiian petrel *Pterodroma sandwichensis*; and the Vulnerable yelkouan shearwater *Puffinus yelkouan*, Cook’s petrel *Pterodroma cookii* and spectacled petrel *Procellaria conspicillata* (Fig. 2a). The proportion of total exposure risk within each IUCN Red List category differs from the proportion of tracked species within each category, with a greater percentage of the exposure risk shared among Threatened species, particularly Critically Endangered species (Fig. 2b). The 20 highest-scoring species had greatest plastic exposure risk in five areas, both in coastal regions (Mediterranean/Black Sea, northwest Pacific) and ocean gyres (northeast and northwest Pacific, South Atlantic, southwest Indian oceans; Figs. 1d, 2a). Plastic exposure risk was low in upwelling zones (Humboldt and Canary currents) and polar regions (Fig. 1d). For some species, scores differed greatly among populations (Fig. 2a). For example, European storm-petrels *Hydrobates pelagicus* breeding in the Mediterranean had much higher scores (306–534) than elsewhere (1.0–1.4; Supplementary Fig. 1). There was no long-term trend in exposure risk scores for populations tracked in the same months for more than three years (Supplementary Fig. 2). By using tracking data to estimate the relative density of regularised bird locations, instead of using only estimated presence or absence, we explicitly consider spatio-temporal variation in seabird distributions, thus providing more detail on global plastic exposure risk for a subset of species than an analysis based on range maps, which inferred different geographic hotspots of plastic exposure risk^14^.

### Breeding and non-breeding season exposure risk

**Fig. 3:**
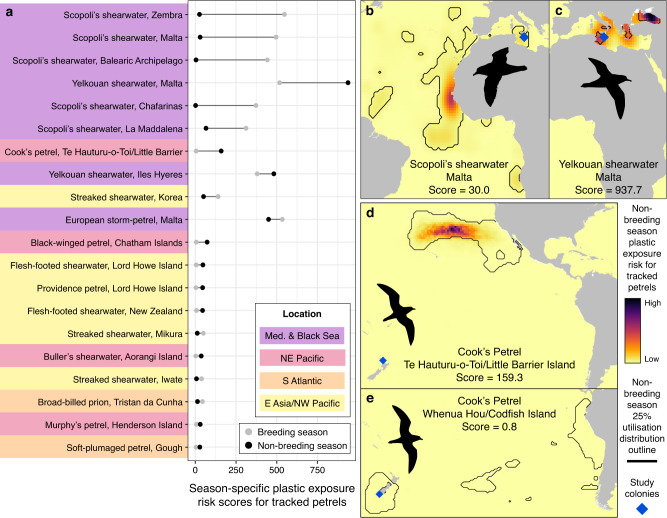
Season-specific plastic exposure risk scores. **a** Scores during breeding (grey circles) and non-breeding seasons (black circles) for the 20 populations with the greatest differences between seasons (grey lines). **b** Non-breeding season plastic exposure risk for Scopoli’s shearwaters (non-breeding score = 30.0, breeding season score = 496.24) and **c** yelkouan shearwaters (non-breeding = 937.7, breeding =517.5) for tracked from Malta, and for Cook’s petrels breeding either at **d** Te Hauturu-o-Toi/Little Barrier Island (non-breeding = 159.3, breeding = 5.5) or **e** Whenua Hou/Codfish Island (non-breeding = 0.8, breeding = 2.1). Black lines indicate the outline of the most used area in the non-breeding season (top 25% of the utilisation distribution). Land polygons from Natural Earth. Source data are provided as a Source Data file.

We calculated breeding and non-breeding plastic exposure risk scores for 107 populations of 60 species. The mean difference between seasons was 34.0, with little difference for most populations (median = 3.6), but substantial differences for some (maximum = 521.8; Fig. 3a). For example, Scopoli’s shearwaters *Calonectris diomedea* breed on Malta in the Mediterranean and migrate to the eastern Atlantic Ocean where they had a much lower plastic exposure risk score (30.0) than during the breeding season (496.2). In contrast, yelkouan shearwaters also breed on Malta (517.5), but had a higher score during non-breeding (937.7) when they disperse within the Mediterranean and migrate to the Black Sea (Fig. 3a–c). Seasonal contrasts also varied among populations of the same species. For example, scores for Cook’s petrels during non-breeding were much higher for birds breeding in northern New Zealand that migrate to the northeast Pacific (159.3), than those breeding in southern New Zealand that migrate to the Humboldt Current (0.8; Fig. 3a, d, e).

### Exposure risk and ingestion

Plastic exposure risk, as indicated by our scores, is necessary but not sufficient for ingestion to occur and there are not yet enough suitable samples to quantify this process for most species. The amount of ingested plastic detected in seabirds is affected by foraging style, body size, tendency to regurgitate, gut morphology, prey type, age and breeding stage^20,22,23,28^. Few ingestion studies have used standardised protocols to sample different populations of the same species^4^. Furthermore, ingestion data are influenced by whether samples came from pellets^26^ or regurgitates^18^, or necropsies of birds that were found dead at a colony^29^ or on beaches^35^, recovered after attraction to light pollution^36^, bycaught in fisheries^37^, or taken for research^28^ or human consumption^4^. Nonetheless, studies that compared ingestion for different populations of the same species using the same methods control for these factors, and so can be compared to our exposure risk scores. For example, flesh-footed shearwaters *Ardenna carneipes* sampled in eastern parts of their breeding range contained significantly more plastic^20^, consistent with our higher scores during the non-breeding season for populations migrating to the northwest Pacific (New Zealand = 44.9; Lord Howe = 47.1) compared with those migrating to the eastern Indian Ocean (Western Australia = 13.6). Additionally, the Ecological Quality Objective for part of the North Sea target of <10% of northern fulmars *Fulmarus glacialis* containing ≥0.1 g of plastic was exceeded more in the North Sea than Arctic Canada^38^, mirroring our exposure risk scores for those tracked from the UK (1.4) and Canada (0.25). There are clear examples of high ingested plastic loads in high exposure risk areas in the Mediterranean^37^, northeast Pacific^39^ and southwest Indian Ocean^36^. However, plastic loads are both low and high in areas with low exposure risk^40^, indicating that birds may still be at risk while foraging in marine areas with low estimated plastic densities. Plastic has been ingested even by the species with the lowest exposure risk score of 0.003 (4% of 27 sampled snow petrels *Pagodroma nivea*, which forage around Antarctica, contained plastic^40^), indicating that the ubiquitous availability of plastic is concerning across all oceans worldwide, not only in areas where plastic aggregates.

### Jurisdictions and policy

**Fig. 4:**
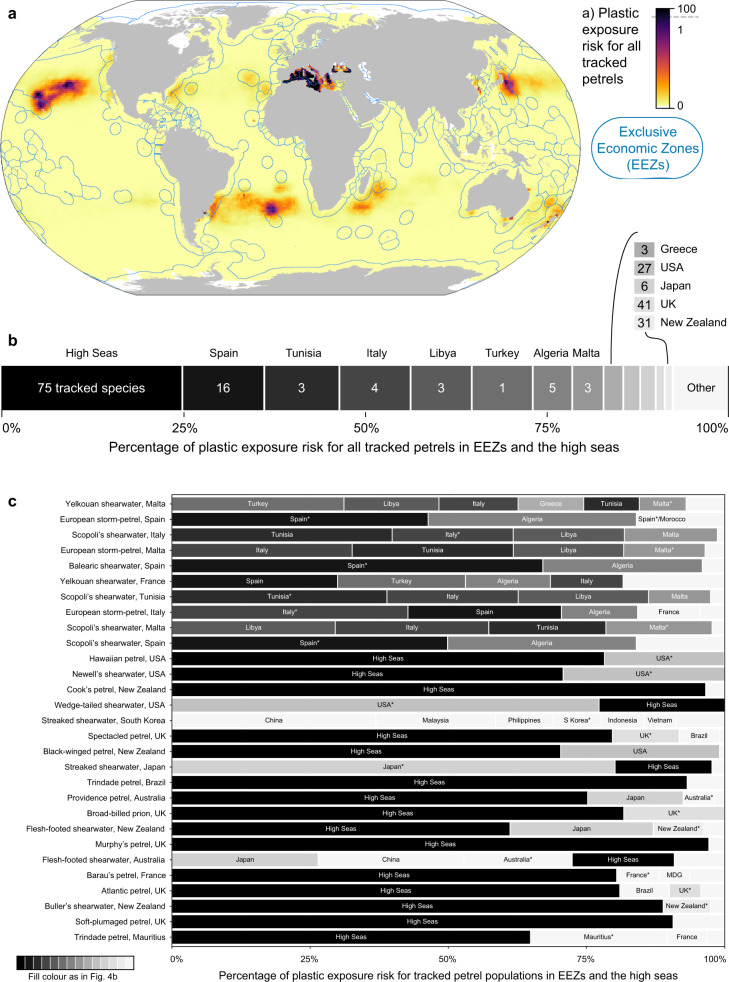
Plastic exposure risk for petrels in different jurisdictions. **a** Map of plastic exposure risk for 77 petrel species in the Exclusive Economic Zones (EEZs) of each country (including overseas territories) and the high seas (Areas Beyond National Jurisdiction). In the Mediterranean, theoretical EEZs are used. For visualisation only, the score is capped at 1% due to extreme values in the Mediterranean and Black Seas. **b** The percentage of plastic exposure risk score attributed to the high seas and each EEZ/theoretical EEZ accounting for >1% of total exposure risk, labelled with the number of tracked species using each area (values are provided in Supplementary Table 1). **c** For the 29 petrel populations by country with the highest exposure risk scores (ranked from high to low), bars show the proportion of the exposure risk score in each jurisdiction that accounts for over 5% of the total exposure risk, with unlabelled bars containing all others. Bars are coloured according to b. Overlapping territorial claims are shown as claim 1/claim 2. MDG = Madagascar. Asterisks(*) indicate that the EEZ matches the breeding country. Land polygons from Natural Earth. Source data are provided as a Source Data file.

Plastic exposure risk for tracked petrels occurred mostly in the Mediterranean and Black Seas (Fig. 4a, b), where breeding European storm-petrels and Scopoli’s, yelkouan and Balearic shearwaters are at risk, with high plastic loads recorded^37,41^. Elsewhere, the high seas are used by 75 of our 77 tracked species, and accounted for 25% of global plastics exposure risk, mainly within oceanic gyres. The US EEZ accounted for a high proportion of the exposure risk, noticeably northeast of Hawai’i, followed by the EEZs of Japan, and the UK, mainly around the Overseas Territories of Tristan da Cunha and Bermuda (Fig. 4a, b). The New Zealand EEZ ranked highly despite low plastic levels due to the exceptionally high petrel occurrence and diversity. Moderate plastic exposure risk scores (0.15–1.00% of total) occurred in the EEZs of France, Australia, Brazil, Portugal, Mauritius, China, Russia, Argentina, Madagascar, Bahamas, and Mexico (Fig. 4a).

Our results indicate that mitigating plastic pollution in the breeding country’s EEZ alone would not adequately protect most species throughout the annual cycle. We identified links between the countries within which each tracked petrel population breeds (including overseas territories) and the jurisdictions where those populations were exposed to plastic (Fig. 4c). Exposure risk primarily occurred outside the breeding country’s EEZ (theoretical EEZ in the Mediterranean because actual EEZs are not clearly defined), except for 7 of the 29 highest-scoring populations (e.g., wedge-tailed shearwaters *Ardenna pacifica* in the USA, and streaked shearwaters *Calonectris leucomelas* in Japan). Of the 29 highest-scoring populations, 25 were exposed to plastic in multiple EEZs. For example, streaked shearwaters breeding in South Korea were exposed in China, Malaysia, the Philippines, South Korea, Indonesia and Vietnam (Fig. 4c). Exposure risk was greatest in the high seas for 15 of the 29 highest-scoring populations, particularly those breeding in the USA, New Zealand, UK, Brazil, Australia, France, and Mauritius (Fig. 4b). For each petrel population, we provide the percentage of exposure risk occurring in each EEZ and the high seas to facilitate targeting mitigation and policy efforts towards key areas (Supplementary Data 1).

Marine vertebrates and plastic debris are globally distributed and highly mobile, and cross political boundaries within and beyond national jurisdictions^11^. Therefore, mitigating plastic pollution from marine and terrestrial sources will require efforts targeted across multiple jurisdictions and the high seas^42^. International cooperation, collaboration, resource mobilisation and information exchange are key to addressing marine plastic pollution^43^ by limiting still-increasing plastic waste production^13^, improving waste management, and cleaning up existing plastic. The International Convention for the Prevention of Pollution from Ships (MARPOL) Annex V prohibiting plastic waste discharge from vessels entered into force 31^st^ December 1988^44^, but plastics from marine sources still affect seabirds^26^ and account for at least 22% of ocean plastics^45^. Ghost fishing gear is a priority because it presents deadly entanglement risk^25^ and food web contamination after degradation at sea. Pollution from vessels could be reduced with more resources and incentives for monitoring and managing waste, and enforcing MARPOL and local regulations, particularly among developing countries^46^. A coordinated approach for plastic waste management could be achieved, for instance, through a global-scale treaty on plastics^43^, which could operate in synergy with MARPOL and other relevant bodies and frameworks, such as the Convention on Biological Diversity, Convention on the Conservation of Migratory Species, Agreement on the Conservation of Albatrosses and Petrels, Regional Seas Conventions and Action Plans.

### Research priorities

Greater use of standard methods for future ingestion studies would facilitate comparison and help identify the drivers of plastic ingestion^4,47^. The relationship between exposure risk, ingestion and impact could be examined by concurrently sampling ingested plastic and tracking movements^41,48^, and measuring physiological impacts. Interspecific differences could be clarified by systematically comparing plastic loads in species that have similar geographic ranges and exposure risk scores. Crucially, it is unclear for which species or populations plastic ingestion reduces survival or productivity and how much exposure they can tolerate; so, studies of population-level impacts and how to separate these from known causes of population declines will be vital^2,5^. Four species with high plastic exposure risk scores but no ingestion data in a recent review^1^ are key research priorities: Hawaiian petrel and streaked shearwater within the main high-exposure risk areas, and Bermuda petrel *Pterodroma cahow* and Desertas petrel *Pterodroma deserta* elsewhere. Comparable ingestion data from different tracked populations of the same species with contrasting migration patterns (e.g., Cook’s petrel; Fig. 3d, e) would be particularly valuable.

Our tracking data covered almost all of the world’s oceans and all ocean regions within the ranges of 70% of analysed species, broadly matching seabird biodiversity in general^10^, but also reflecting known spatial biases in research effort, notably towards the Atlantic Ocean and latitudes south of 40°S^32^ (see Supplementary Table 2 for spatial coverage gaps). Our study included tracking data for all four petrel species that breed in the Mediterranean, but we identified 14 species that occur in other high-exposure risk areas, making them priorities for tracking studies (Supplementary Table 3). Additionally, both petrel tracking and ingestion data are sparse in coastal waters around east and southeast Asia, where high plastic densities occur, and the South Pacific and North Atlantic gyres, where moderate plastic densities occur^10,32^ (Fig. 1). We identified priority species for future research in each of these regions (Supplementary Table 4). Sample sizes varied substantially among species, from 3 to 960 individuals (median = 35, mean = 93), so additional tracking for some species could be beneficial (Supplementary Data 2). Furthermore, tracking immature birds or adults when deferring breeding could reveal differences in exposure risk^33^. Our method could also be applied to global-scale, multi-species tracking datasets^12^ for other marine megafauna, such as turtles and marine mammals, for which plastic pollution is also a threat^1^.

Collecting more data on plastic density, identifying sources, and developing density models to provide better spatial coverage at a higher resolution would aid targeted mitigation strategies, and enable a better understanding of the effects of spatial scale on plastic exposure risk. The models that produced the plastic density estimates used in our analysis involved interpolating over wide areas, whereas observed plastic densities tend to be more patchy^49^. There were limited plastic data, particularly for southeast Asia^6^, where a recent survey recorded high plastic levels^50^. The South Pacific has a high petrel species richness, but few samples were used to inform the plastic density models^6^. The plastic density model estimates covered most of the Arctic and Antarctic oceans, but had more missing values near the poles than in other regions (Fig. 1b), although the Southern Ocean is not thought to contain much plastic^6^. However, plastic accumulates around Svalbard in the Arctic^51^, which although only important for northern fulmars among petrels, could affect other taxa. Marine species also feed at different depths and so it would be valuable to examine how plastic varies vertically^52^. Repeated plastic sampling across longer timescales would improve temporal matching between plastic and seabird data and allow investigations into long-term changes in plastic exposure risk^53^. We provide example versions of the code used to produce our results to facilitate future research on different tracking or plastics datasets^54^.

## Methods

In brief, we collated tracking data for petrels and computed gridded utilisation distributions (UDs) at a monthly scale. We then combined gridded distributions of marine plastic density and multiplied them by the petrel UDs to map estimated exposure risk. For each map, we summed the plastic exposure risk values in all cells to provide a score representing relative estimated exposure risk. We combined maps and scores to investigate variation in exposure risk between breeding and non-breeding seasons, among populations and species, and across Exclusive Economic Zones (EEZs) and the high seas. Steps for processing and analysing the data are described in detail below and represented graphically in Supplementary Fig. 3. All data handling was carried out in R^55^ and R scripts are provided, along with example data and templates^54^.

### Petrel tracking data collation and processing

We collated tracking data that were collected using Global Positioning System (GPS) loggers, Platform Terminal Transmitters (PTTs) and Global Location Sensor (GLS) loggers deployed on adult petrels (Table S1; Oceanitidae, Hydrobatidae and Procellariidae). We searched for published and unpublished tracking data for all petrel species between March and August 2020, excluding the two giant petrel species *Macronectes giganteus* and *M. halli* because our analyses focused on marine areas and they regularly feed on land^56^. We obtained data for 77 species (64% of the 121 target species) from the Seabird Tracking Database (www.seabirdtracking.org), ZoaTrack (www.zoatrack.org)^57^, Movebank (www.movebank.org)^58^, and individual researchers (represented by authors of this study or detailed in the Supplementary Acknowledgements). We collated 1,736,880 tracked locations for 7137 individuals tracked from 27 countries and Antarctica. Datasets varied in terms of number of colonies per species, and numbers of individuals, years, and months tracked per population (Supplementary Data 2) and species (Supplementary Data 3).

We standardised tracking datasets to contain the following fields in the same format: latitude, longitude, datetime, species, colony name, colony latitude, colony longitude and device type. For GLS, we removed locations around the equinoxes (March equinox: −21, +7 days; September equinox: −7, +21 days) as they are unreliable^59^, unless latitudes were estimated using additional information such as sea surface temperature prior to our analysis. For GPS and PTT data, we filtered locations for unrealistic speeds (>90 km/h), and visually checked maps and removed locations that were clear outliers. We removed locations within 5 km of the colony for GPS data or within 15 km of the colony for PTT data, but not for GLS locations due to large location error for these devices. We linearly interpolated and resampled GPS and PTT datasets to the sampling frequency for GLS of two locations per day.

We grouped data for each species into 148 breeding populations determined according to jurisdiction, the distance between colonies, and overlap in at-sea distributions based on the tracking data, i.e., if distributions overlapped substantially (at a 1 × 1° scale) and colonies are in close geographical proximity and in the same country, we considered colonies to belong to the same population.

### Density of tracked petrel locations

For each population, we pooled all locations for all individuals across all years by month, and then removed months with fewer than five locations. For each month, we reprojected tracked locations onto a Lambert azimuthal equal area projection centred around the geometric mean of all locations. We estimated kernel densities of tracked locations to compute a 95% UD, a common home-range metric, which, because the sampling frequency was standardised, represented the estimated time spent by all tracked petrels in that population within that month. We used the adehabitatHR R package^60^, using a cell size of 10 km^2^ and a smoothing factor of 200 km (based on the magnitude of error in estimating locations from GLS^33^). We trimmed all cells that fell over land (Natural Earth land 1:10 m polygons version 5.1.1 downloaded from www.naturalearthdata.com/) because these species do not forage in terrestrial environments and it is extremely rare for them to travel over land, so any locations are most likely due to device error^33^. We then reprojected the resulting rasters back to a latitude and longitude projection (WGS84).

Of the 148 tracked populations, 108 (61 species) were tracked both in the breeding and non-breeding seasons. For these populations, we collated published information on the timing of breeding at a monthly scale (Supplementary Data 4) for each species or, where possible, each population. We also labelled months as breeding or non-breeding based on the tracking data. Locations were not always available for all months, with March and September often excluded from GLS datasets due to the uncertainty in light-based geolocation around equinoxes. We first calculated the distance between each location at sea and the breeding colony. For each population, we calculated the mean distance from the colony for each month, and a mean of those monthly means. If the mean for a month was greater than the population-specific mean across all months or if no individuals travelled within 200 km (chosen due to the approximate 200 km error common when using GLS devices) of the colony, this month was classified as non-breeding. To ensure there was only one breeding and one non-breeding season, if the classification of one month differed from the previous and following months, it was re-classified. We used published values except in cases when a month was labelled as breeding, but the tracking data showed that the subset of tracked birds did not attend the colony during that month, in which case, we used the label identified by the distance-to-colony method. Breeding and non-breeding months, therefore, do not necessarily represent the general phenology of the species, but instead reflect the behaviour (distance from the colony) of the majority of tracked individuals in that month. A sensitivity analysis showed that plastic exposure risk scores calculated using published breeding schedules were highly correlated with those estimated using the tracking data, Kendall’s tau = 0.98 (*z* = 13.879, *p* < 0.001) for the breeding season, and tau = 0.97 (*z* = 10.810, *p* < 0.001) for the non-breeding season.

### Plastic density distribution

We used estimated global marine plastic density (count per km^2^) in 1 × 1° grid cells, from publicly available outputs from three published Lagrangian particle tracking models (Maximenko^34^, Lebreton^9^, and van Sebille^6^). The model estimates combined floating micro and macro-plastics from ~0.333 mm to 40 cm, with different size classes having similar estimated distributions^7^. Although petrels can ingest plastic flexible plastic pieces 40–60 cm long, they generally consume smaller pieces^61^. The three models estimated plastic density using records from ~12,000 surface trawls. They provided particularly good spatial coverage in the northeast Pacific, northwest Atlantic and Australian waters, but particularly poor coverage at the poles, the waters around Southeast Asia, the northwest Indian Ocean, and the South Pacific^6^. The models simulate the movement of plastic particles through multiple years and then create a static probability grid for a single time point (2014) based on where particles spent most time up until 2014 (equivalent to a utilisation distribution). We do not expect interannual variation in plastic distribution to be substantial in comparison to the spatial scale of between-season seabird movement because plastics travel passively, take decades to break down, and have been released throughout the study period. Each model uses the trawl data along with weather conditions, ocean circulation models, and plastic sources and sinks to inform the movement of plastic particles and predict the number of particles in each sampled and unsampled 1 × 1° grid cell. The Maximenko model assumes particles can wash ashore and originate from a uniform input across the ocean surface^34^, the van Sebille model assumes no sinks for plastic and plastics originate at the coast^6^, and the Lebreton model assumes no sinks for plastic and plastics are sourced from river mouths^9^. None of the models incorporate sinking through the water column^52^, ingestion by marine organisms^1^, or fragmentation processes. For each ocean basin and model, a prediction value was compared to observed plastic counts, providing regression coefficients used to scale the model plastics distribution and predict plastic concentrations within all cells^6^. Each model represents observed ocean plastic concentrations well^6^, with observations generally falling within 1–2 orders of magnitude around the model estimate. Further details on the methods used to model plastic density, including on how regression coefficients were used and validated, are provided in Maximenko et al.^34^, Lebreton et al.^9^, and van Sebille et al.^6^. Despite the variation in sampling effort, the model outputs generally agree with subsequent surveys in the Mediterranean^62^, southeast Pacific^63^ and southeast Asia^50^.

We took the geometric mean (as opposed to the arithmetic mean) of the Maximenko^34^, Lebreton^9^, and van Sebille^6^ models to avoid bias in our plastic density layer toward the highest estimate from any individual model because the models have log scale variability between their estimates. Additionally, because the ocean is in constant flux, concentrations at any given location are constantly changing^53^, assuming a lognormal distribution of concentrations through time, the geometric mean will be a better estimate of the central tendency and closer to the median concentration than the arithmetic mean^64^. The model outputs varied in spatial coverage in coastal and polar regions (Supplementary Fig. 4), and when one of the models did not have an estimate within a cell, we used the geometric mean of the other models, or the estimate from the only available model. If there was no estimate from any model, this was marked as NA, which occurred mostly in the Arctic and the Antarctic, and in some coastal areas where the marine area was less than the 1 × 1° grid size. The model outputs were centred around 180°E. Values in cells at 0–1°W were incorrectly estimated so these were imputed from the mean values in the three adjacent cells east and west (177–180°E and 1–4°W).

### Plastic exposure risk scores

We aggregated the monthly 10 × 10 km petrel 95% UDs for each population^33^ onto the same 1 × 1° global grid of the plastic density data. All petrel UDs and the plastic density grid were divided by the respective cumulative sum for each grid so that the values of each entire raster grid summed to one. We estimated exposure risk as the mathematical product of the petrel and the plastic values in each grid cell^14^. This gives equal weight to the number of plastic pieces in each cell and the density estimate for bird tracking locations in each cell. We assume that estimated density of bird tracking locations at equal time intervals is strongly related to the time spent at risk of exposure to plastic debris, because areas where seabirds spend more time are very likely to be where foraging is concentrated^65^, as a result of area-restricted searching behaviour^66–68^. We then summed all cell values and multiplied all scores by 1,000,000 to reduce the number of decimal places to produce a single score for that month (ranging from 0.0007 to 1091). For comparison, we calculated a theoretical score of 15.3, which represents what the exposure risk score would be for any species if all global grid cells contained the mean plastic density (i.e., assuming that plastic was evenly distributed across the world’s oceans). We combined monthly grids to produce grids for each population, breeding or non-breeding season (if data were available for non-breeding months) and species. Scores for each population are the mean of all tracked months, and scores for each season are the mean of all months in that season (Supplementary Data 5). We used the mean to allow comparison between species with different numbers of tracked months. Maps for most populations are in Supplementary Fig. 5. For the 33 species for which we had multiple tracked populations, we searched for published population estimates (Supplementary Data 6). We calculated species-level scores as the mean of scores for each population weighted by the population size and multiplied by 0.5 if the population was only tracked during the breeding season (Supplementary Data 7).

We tested how robust our results were in relation to population size estimates, sampling frequency and tracking year. Population estimates for some species have large uncertainty, so we tested the correlation between species-level scores calculated with and without weighting by population size using Kendall’s tau because scores are not normally distributed. They were highly correlated (tau = 0.83; *T* = 483, *p* < 0.001), so our results are unlikely to be affected by uncertainty in population size estimates.

To investigate possible effects of sampling frequency, we reprocessed the tracking data without subsampling all datasets to 12-hourly intervals. We identified 44 populations for which all data were derived from GPS or PTT devices. For each track, we calculated the median interval between successive locations and recorded the maximum median for each population, and if this was less than 6 h, we regularised tracking locations at that frequency (intervals ranging from 1 min to 5 h, median = 1 h, mean = 82 min). We performed kernel density estimation with the higher-frequency datasets using a smaller 50 km smoothing factor^33^ for the remaining 39 populations and used them to calculate exposure risk scores for each population. The scores estimated using the higher and lower resolution data were highly correlated (tau = 0.90, *T* = 703, *p* < 0.001), so we conclude that 12-hour sampling intervals and 200 km smoothing parameter are sufficient for a study of this scale.

Birds were tracked between 1995 and 2020 with a mean tracking year of 2012. Among the 148 populations, 139 (94%) were tracked within 5 years of 2014 (2009–2019), the year for which plastic density was estimated. Given petrels are long-lived and generally faithful to breeding sites^69^ and foraging areas during both breeding and non-breeding seasons^70–73^, we assumed that distributions were unlikely to vary substantially across the study period. Data on long-term trends in plastic ingestion by seabirds have not shown substantial increases during the study period^27,74,75^. A subset of 13 populations had been tracked with geolocators for the same set of months across more than three years (Supplementary Fig. 2). For these, we calculated an exposure risk score for each year and then tested the effect of population and year using a generalised linear model with a Gamma distribution (due to positive continuous right-skewed response variable). We checked model fit by simulating residuals using the DHARMa R package^76^.

We recorded the most recent IUCN Red List assessment threat category^77^, where 36 species were Least Concern (LC), 9 Near Threatened (NT), 16 Vulnerable (VU), 10 Endangered (EN) and 6 Critically Endangered (CR). Red List status categories from the year each species was first tracked remained the same for 71 of the 77 species, and we used the most recent assessment for the 6 species for which changes have occurred. Three were genuine changes relating to altered threats or conservation action (Westland petrel *Procellaria westlandica* from VU in 2016 to EN in 2017; Chatham petrel from CR in 2008 to EN in 2009 to VU in 2015; yelkouan shearwater from LC in 2004 to NT in 2008 to VU in 2012), while three were not genuine changes because they related to improved evidence for assessment (flesh-footed shearwater from LC in 2012 to NT in 2016; streaked shearwater from LC in 2012 to NT in 2015; spectacled petrel from CR in 2005 to VU in 2007)^77,78^. We calculated the proportion of the total of all exposure risk scores attributed to species in each threat category.

### Spatial patterns in plastic exposure risk

We used the ranked species scores to identify global-scale high -exposure risk areas by recording the region in which each species had the highest scores. We created an all-species map by summing results for each species, with those tracked in both breeding and non-breeding seasons given a weight of 1, while the 16 species that were tracked only in the breeding season were given a weight of 0.5 to avoid undue bias towards breeding colonies. We also divided the all-species distribution grid by the cumulative sum so that all values sum to one and multiplied this by the plastic density grid to produce an exposure risk map. We then overlapped this all-species map with EEZs and the high seas, obtained as an open-source polygon layer^79^. Because national jurisdictions in the Mediterranean are not yet clearly defined or are subject to dispute, we used theoretical EEZs, which are defined as 200 nautical miles from the coastline or the median point between two coastlines unless treaties and agreements have been submitted to the UN^80^. We calculated the proportion of the global risk of exposure to plastic for all petrels in each EEZ/theoretical EEZ and in the high seas. For joint regimes and overlapping claims, the score was divided evenly between the involved sovereigns. To record the links between the breeding country and the jurisdictions of plastic exposure risk, we calculated the proportion of plastic exposure risk for each population by country in each EEZ/theoretical EEZ and in the high seas^11^ (Supplementary Data 1).

### Spatial coverage and research priorities

To assess spatial coverage and identify research priorities for tracked species, we compared the distribution of the tracking data for each species with the estimated range maps^77^. We assessed whether major populations (>1% of the global population or 200 pairs) of each tracked petrel species were missing from any of 10 major ocean areas (NW/NE/SW/SE Atlantic, NW/NE/SW/SE Pacific, Indian or Southern Oceans) according to the SeaVoX Salt and Fresh Water Body Gazetteer (https://www.marineregions.org/). Our tracking data covered all ocean regions within the published estimated ranges of 54 of the 77 species considered (70%). Our data compilation also revealed the main gaps in coverage for the remaining 23 species (Supplementary Table 2).

To identify research priorities for high exposure risk areas identified in this study, we used range maps to identify species or populations for which tracking data were not included in this study, but range maps indicated they may overlap (Supplementary Table 3). We recorded ingestion frequency of occurrence as the percentage of individuals found to contain plastic and the number examined as reported in Kühn & van Franeker^1^. We also carried out this process for areas for which plastic density is high and range maps showed that petrel species may use these areas, but no tracking data were available for our study (Supplementary Table 4).

### Reporting summary

Further information on research design is available in the Nature Portfolio Reporting Summary linked to this article.

## Supplementary information

Supplementary Information

Peer Review File

Description of Additional Supplementary Files

Supplementary Dataset 1

Supplementary Dataset 2

Supplementary Dataset 3

Supplementary Dataset 4

Supplementary Dataset 5

Supplementary Dataset 6

Supplementary Dataset 7

Reporting Summary

## Data Availability

The plastic exposure risk data generated in this study and the plastic density data used in this study are provided in the Supplementary Data files, available at https://github.com/BirdLifeInternational/petrels-plastics and have been deposited in the Zenodo database at 10.5281/zenodo.7852143. The seabird tracking data are available under restricted access because the data were collected for other purposes that vary between datasets and revealing the exact locations of sensitive species may put them at risk. Access can be obtained by making a request to the owners of each dataset using the mechanisms provided by each database. Zoatrack (https://zoatrack.org/) dataset IDs: 57, 93, 102–112, 159, 253, 254, 762, 817. Movebank (https://www.movebank.org/) dataset IDs: 944960474, 200628745, 241140274. SEATRACK (https://seapop.no/en/seatrack/) for relevant northern fulmar data. U.S. Geological Survey data release: 10.5066/P9NTEXM6. Seabird Tracking Database (https://www.seabirdtracking.org/) dataset IDs: 434, 438, 439, 448, 466, 467, 506–511, 517, 518, 554, 555, 561, 571, 607, 609, 610, 627, 628, 634, 635, 637, 639, 658, 659, 662, 663, 667, 668, 670, 672–678, 683, 684, 686, 694–696, 704–706, 708–715, 736, 741, 783–786, 788, 789, 826, 827, 829–831, 836–842, 844, 854, 858–872, 879, 883–886, 888–893, 900, 945, 946, 949, 951–954, 959–963, 966, 967, 970–983, 986–998, 1004, 1028, 1029, 1031–1033, 1055–1061, 1081, 1083, 1084, 1086–1091, 1120, 1121, 1140–1142, 1233–1236, 1238, 1239, 1258, 1259, 1279, 1280, 1282, 1285–1289, 1298, 1314, 1317, 1326, 1343–1347, 1360–1362, 1375, 1386, 1401, 1404, 1409, 1410, 1413–1415, 1422–1425, 1440, 1443, 1449, 1452, 1453, 1460, 1461, 1463, 1481, 1482, 1485–1488, 1494, 1497–1500, 1520–1523, 1541, 1544, 1546, 1549–1551, 1553–1558, 1562–1570, 1574–1577, 1579–1582, 1585–1592, 1594–1600, 1602, 1603, 1606–1608, 1610, 1618, 1619, 1621–1625, 1630, 1665, 1668–1672, 1690, 1711–1717, 1738, 1908–1923, 2036–2038, 2042, 2044–2046–2049, 2051–2056, 2059, 2060, 2063–2066. Source data are provided with this paper.

## Source data

Source Data

## Code availability

R code used to produce the analysis can be accessed at https://github.com/BirdLifeInternational/petrels-plastics with the version on the date of publication archived at https://zenodo.org/record/8033861^54^.
