# Supplementary material for: Global assessment of marine plastic exposure risk for oceanic birds
Source: Nat Commun. 2023 Jul 4;14:3665. doi: 10.1038/s41467-023-38900-z (PMC10319801; doi:10.1038/s41467-023-38900-z)
Supplement: Supplementary file 1 — Supplementary Information [file 41467_2023_38900_MOESM1_ESM.pdf]

Supplementary Information

Global assessment of marine plastic exposure risk for oceanic birds

Clark *et al.*

Contents **Page**

- Supplementary Fig. 1. Plastic exposure risk for different European storm-petrel populations 2
- Supplementary Fig. 2. Exposure risk scores per year 2
- Supplementary Fig. 3. Graphical representation of the method for calculating marine plastic exposure risk scores from tracking data 3
- Supplementary Table 1. Plastic exposure risk in each EEZ and the high seas 4
- Supplementary Table 2. Gaps in the spatial coverage of tracking data among species included in this study 4
- Supplementary Table 3. Research priorities for tracking and ingestion studies in high exposure risk areas 5
- Supplementary Table 4. Research priorities for tracking and ingestion studies in areas with potential high or moderate exposure risk 6
- Supplementary Fig. 4. Spatial coverage of plastic density estimates 6
- Supplementary acknowledgements 7
- Supplementary Fig. 5. Plastic exposure maps for the breeding and non-breeding seasons for each population 13

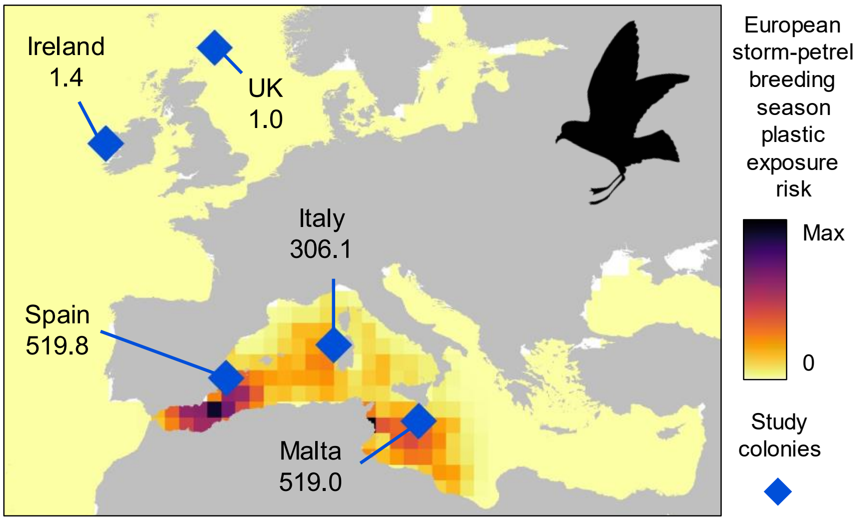

Supplementary Fig. 1 | Variation in plastic exposure risk for different European storm-petrel populations. Individuals were tracked during the breeding season from colonies in the UK (breeding season plastic exposure = 1.0), Ireland (1.4), Italy (306.1), Malta (534.2) and Spain (519.8). Land polygons from Natural Earth.

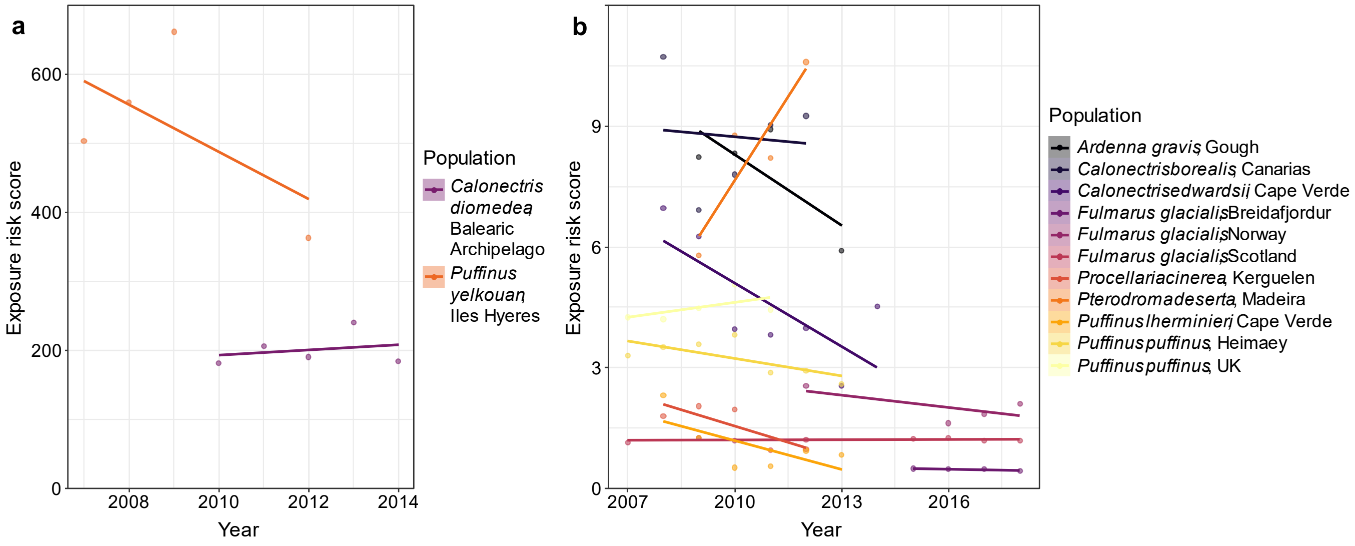

Supplementary Fig. 2 | Exposure risk scores per year. Exposure risk scores by year for the populations GLS tracked in the same set of months for more than three years for **a**, all populations and **b**, excluding the two highest scoring populations. A generalised linear model with a Gamma distribution showed that, across all populations, there was no effect of year ($X^2_1 = 0.7$, $p = 0.391$; Estimate = 0.0001 ± 0.0001), while controlling for the strong effect of population ($X^2_{12} = 3993.5$, $p < 0.001$). Hypothesis testing was two-sided. No adjustments were made for multiple comparisons because all populations were fitted in a single model. Trend lines for each population are linear smooths for illustration only. Source data are provided as a Source Data file.

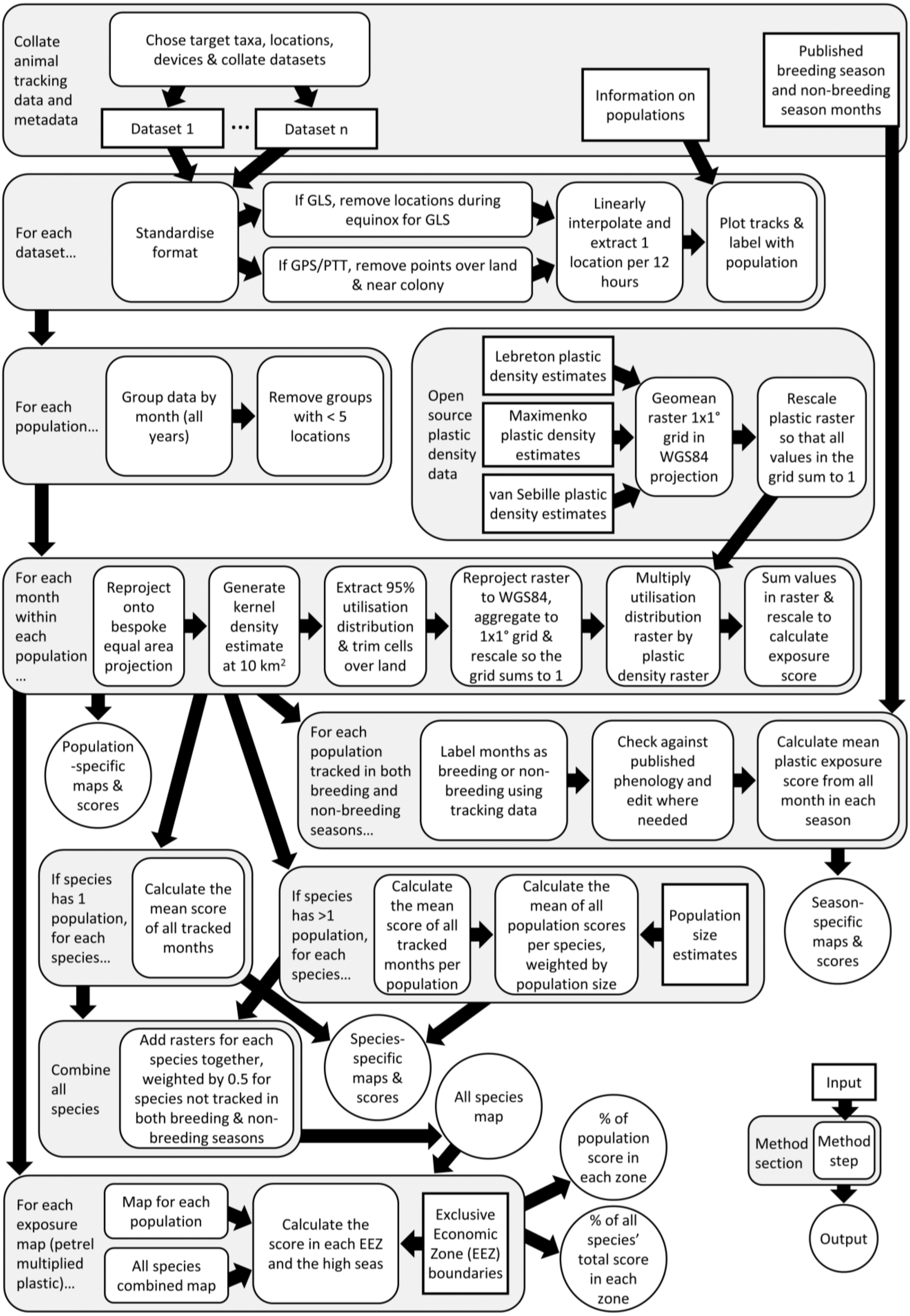

Supplementary Fig. 3 | Graphical representation of the method for calculating marine plastic exposure risk scores from tracking data. Input datasets are represented by rectangles, method steps are represented by white rounded rectangles, sections of related method steps are represented by grey rounded rectangles, and outputs are represented by circles.

Supplementary Table 1 | Plastic exposure risk in each EEZ and the high seas. Percentage of the plastic exposure risk for all tracked petrels occurring in each Exclusive Economic Zone (EEZ; or theoretical EEZ in the Mediterranean) and in the high seas, ordered from highest to lowest. The n species value is the number of the 77 tracked petrel species that had their 95% utilisations distributions overlapping with that EEZ. In total, the EEZs of 143 countries were used by tracked petrels. *List of EEZs for which any of petrel species' 95% utilisation distribution overlapped but accounted for <0.02% of the total plastic exposure risk in order of % or exposure.

EEZ	%	n species	EEZ	%	n species	EEZ	%	n species
High seas	24.72	75	Portugal	0.59	18	Chile	0.07	20
Spain	11.29	16	Republic of Mauritius	0.26	5	Japan/Russia	0.06	5
Tunisia	10.35	3	Russia	0.24	9	Seychelles	0.06	6
Italy	9.79	4	China	0.24	2	Ireland	0.06	12
Libya	8.43	3	Spain/Morocco	0.23	8	Monaco	0.06	4
Turkey	8.40	1	Ukraine	0.14	1	South Korea	0.05	4
Algeria	5.43	5	Argentina	0.21	14	Ecuador	0.04	13
Malta	4.32	3	Madagascar	0.20	9	Colombia	0.04	9
Greece	2.72	3	Bahamas	0.18	7	France/Madagascar/Mauritius	0.03	7
United States	2.33	27	Mexico	0.16	8	Cyprus	0.03	1
Japan	2.14	6	Uruguay	0.14	12	Cape Verde	0.03	15
United Kingdom	1.25	41	Bulgaria	0.13	1	Albania	0.03	3
New Zealand	1.09	31	South Africa	0.12	18	Dominican Republic	0.02	5
Georgia	0.95	1	Canada	0.11	16	Indonesia	0.02	4
France	0.92	49	Egypt	0.10	1	India	0.02	4
Australia	0.91	35	Romania	0.10	1			
Brazil	0.66	18	Morocco	0.09	13			

*Israel, Montenegro, Norway, Micronesia, Syria, Malaysia, Namibia, Palestine, Denmark, Peru, Western Sahara, North Korea, Mozambique, Maldives, Haiti, Philippines, Lebanon, Panama, Vanuatu, Palau, Sri Lanka, Costa Rica, Jamaica, France/Vanuatu, Netherlands, Iceland, Vietnam, Angola, Federal Republic of Somalia, Brunei, Taiwan, Cuba, Comores, Tanzania, Germany, Pakistan, Singapore, Taiwan/Japan/China, Mauritius, Saudi Arabia, Papua New Guinea, Japan/South Korea, Madagascar/France, Haiti/United States/Jamaica, Guinea-Bissau, Tonga, France/Comores, Fiji, Oman, Kenya, Senegal, Yemen, South China Sea, Gabon, Myanmar, Republic of the Congo, Sudan, Democratic Republic of the Congo, Venezuela, Antarctica, Guinea, Mauritania, Solomon Islands, Colombia/Dominican Republic/Venezuela, Kenya/Federal Republic of Somalia, Sudan/Egypt, United States/Dominican Republic, Antigua and Barbuda, Honduras, Venezuela/Netherlands/Dominican Republic, Djibouti, Bangladesh, Marshall Islands, Gambia, Equatorial Guinea, Sierra Leone, Eritrea, Ghana, Nicaragua, Ivory Coast, Thailand, Eritrea/Djibouti, Iran, Kiribati, Nigeria, Liberia, Barbados, Guyana, Suriname, Sweden, Guatemala, Trinidad and Tobago, Belize, Guyana/Trinidad and Tobago/Venezuela, Dominica, Sao Tome and Principe, Nauru, Tuvalu, East Timor, El Salvador, Saint Kitts and Nevis, Saint Vincent and the Grenadines, Samoa, Saint Lucia, Grenada, Benin, Togo, Finland, Poland, Cambodia, Estonia.

Supplementary Table 2. | Gaps in the spatial coverage of tracking data among species included in this study. Estimated range maps available at datazone.birdlife.org showed major populations (>1% of global population) of 23 tracked petrel species were missing from any ocean areas (NW/NE/SW/SE Atlantic, NW/NE/SW/SE Pacific, Indian or Southern Oceans) according to the SeaVoX Salt and Fresh Water Body Gazetteer*. Suggested new populations do not account for feasibility.

Common Name	Scientific name	Tracking data gap (ocean)	Suggested population locations for new tracking studies	Priority data for tracked populations
Flesh-footed Shearwater	*Ardenna carneipes*	NE Pacific, Indian	-	Australia
Bulwer's Petrel	*Bulweria bulwerii*	N Pacific, Indian	China, Bonin Is, Hawaii, Marquesas Is	-
Northern Fulmar	*Fulmaris glacialis*	N Pacific	Russia, Japan, Alaska	-
Band-rumped Storm-petrel	*Hydrobates castro*	N Pacific	Japan, Hawaii, Galapagos	Non-breeding Azores, St Helena
Cape Verde Storm-petrel	*Hydrobates jabejabe*	NW Atlantic	-	Non-breeding
Leach's Storm-petrel	*Hydrobates leucorhous*	NW Pacific, NW Atlantic	Japan	Non-breeding in Atlantic
European Storm-petrel	*Hydrobates pelagicus*	NW/S Atlantic	Faroe Islands, Canary Islands	Non-breeding in NE Atlantic
Wilson's Storm-petrel	*Oceanites oceanicus*	N/SE Atlantic, S Pacific, Indian	Cape Horn, Kerguelen, Antarctica	Non-breeding
South Georgia Diving-petrel	*Pelecanoides georgicus*	Indian, Southern	Prince Edward Is, Crozet, Kerguelen, Heard Is	South Georgia
Common Diving-petrel	*Pelecanoides urinatrix*	Indian	Crozet, Heard Is, Kerguelen	-
Fairy Prion	*Pachyptila turtur*	S Atlantic, Indian	Falklands, Crozet, South Georgia	-
Broad-billed Prion	*Pachyptila vittata*	S Pacific, Indian	New Zealand	-
White-faced Storm-petrel	*Pelagodroma marina*	S Atlantic, S Pacific, Indian	Tristan da Cuhna, Australia, New Zealand	Non-breeding in Atlantic
Tahiti Petrel	*Pseudobulweria rostrata*	S Pacific	Marquesas, Society & Gambier Is, French Polynesia, Fiji, American Samoa	Non-breeding
White-necked Petrel	*Pterodroma cervicalis*	N Pacific	Kermadec Is	Non-breeding
White-headed Petrel	*Pterodroma lessonii*	S Atlantic, SE Pacific	Macquarie, Antipodes, Crozet, Kerguelen	-
Great-winged Petrel	*Pterodroma macroptera*	S Atlantic, Indian	Australia, Tristan da Cuhna, Gough, Crozet, Kerguelen	Non-breeding
Kermadec Petrel	*Pterodroma neglecta*	Pacific	Lord Howe, Kermadec Is, San Ambrosio	Non-breeding
Providence Petrel	*Pterodroma solandri*	NE Pacific	-	Australia
Little Shearwater	*Puffinus assimilis*	Indian	Australia	-
Tropical Shearwater	*Puffinus bailloni*	W Pacific	Line & Pheonix Islands	-
Hutton's Shearwater	*Puffinus huttoni*	Indian	-	Non-breeding
Audubon's Shearwater	*Puffinus lherminieri*	NW Atlantic	Caribbean	-

*Available online at https://www.marineregions.org/. Consulted on 2022-02-11

Supplementary Table 3. | Research priorities for tracking and ingestion studies in high exposure risk areas. Ordered by frequency of occurrence of ingested plastic across all studies from Kuhn & van Franeker, 2020[1] (>20%, no data, ≤20%) and IUCN red list category*. A total of 19 species or populations for which tracking data were not included in this study in the five main high exposure risk areas, but range maps indicated they may overlap with high plastic exposure risk areas. Ingestion frequency of occurrence shows the percent of individuals found to contain plastic and the number examined.

High exposure risk area	Common Name	Scientific name	Red List	Population trend	Ingested plastic frequency of occurrence	Priority tracking data gap (range also occurs in other priority areas)
**Mediterranean and Black Seas** None – all 4 species tracked						
**Northeast Pacific** - 9 species	Tristram's storm-petrel	*Hydrobates tristrami*	LC	Stable	85% of 150	Entire range (NW Pacific)
	Stejneger's petrel	*Pterodroma longirostris*	VU	Decreasing	67% of 72	Entire range (NW & S Pacific)
	Bonin petrel	*Pterodroma hypoleuca*	LC	Decreasing	40% of 72	Entire range (SE Asia)
	Bannerman's shearwater	Puffinus bannermani	EN	Decreasing	No data	Entire range (NW Pacific/E Asia)
	Heinroth's shearwater	*Puffinus heinrothi*	VU	Stable	No data	Entire range
	Christmas shearwater	*Puffinus nativitatis*	LC	Stable	20% of 44	Entire range (S Pacific)
	Band-rumped storm-petrel	*Hydrobates castro*	LC	Decreasing	0% of 7	Pacific
	Kermadec petrel	*Pterodroma neglecta*	LC	Decreasing	0% of 19	Non-breeding season (NW, S Pacific)
	Juan Fernandez petrel	*Pterodroma externa*	VU	Stable	1% of 287	Entire range (S Pacific)
**South Atlantic** - 7 species	Wilson's storm-petrel	*Oceanites oceanicus*	LC	Stable	34% of 244	Non-breeding season (SW Indian Ocean, SE Asia, S Pacific, N Atlantic)
	White-bellied storm-petrel	*Fregetta grallaria*	LC	Decreasing	32% of 37†	Entire range (SW Indian Ocean, S Pacific)
	Grey-backed storm-petrel	*Garrodia nereis*	LC	Decreasing	27% of 11	Entire range
	Cape petrel	*Daption capense*	LC	Stable	19% of 282	Atlantic (SW Indian Ocean, S Pacific)
	Subantarctic shearwater	*Puffinus elegans*	LC	Decreasing	7% of 15‡	Entire range
	Kerguelen petrel	*Aphrodroma brevirostris*	LC	Decreasing	7% of 70	Entire range (SW Indian Ocean)
	Black-bellied storm-petrel	*Fregetta tropica*	LC	Decreasing	0% of 7	Entire range (SW Indian Ocean, S Pacific)
**Northwest Pacific/ East Asia** - 7 species	Tristram's storm-petrel	*Hydrobates tristrami*	LC	Stable	85% of 150	Entire range (NE Pacific)
	Stejneger's petrel	*Pterodroma longirostris*	VU	Decreasing	67% of 72	Entire range (NE & S Pacific)
	Bryan's shearwater	*Puffinus bryani*	CR	Decreasing	No data	Entire range
	Bannerman's shearwater	*Puffinus bannermani*	EN	Decreasing	No data	Entire range (NE Pacific)
	Swinhoe's storm-petrel	*Hydrobates monorhis*	NT	Stable	No data	Entire range (SE Asia)
	White-necked petrel	*Pterodroma cervicalis*	VU	Increasing	8% of 24	Non-breeding season
	Kermadec petrel	*Pterodroma neglecta*	LC	Decreasing	0% of 19	Non-breeding season (NE, S Pacific)
**Southwest Indian Ocean** - 5 species	Wilson's storm-petrel	*Oceanites oceanicus*	LC	Stable	34% of 244	Non-breeding season (N, S Atlantic, SE Asia, S Pacific)
	White-bellied storm-petrel	*Fregetta grallaria*	LC	Decreasing	32% of 37†	Entire range (S Atlantic, S Pacific)
	Cape petrel	*Daption capense*	LC	Stable	19% of 282	SW Indian Ocean (S Atlantic, S Pacific)
	Kerguelen petrel	*Aphrodroma brevirostris*	LC	Decreasing	7% of 70	Entire range (S Atlantic)
	Black-bellied storm-petrel	*Fregetta tropica*	LC	Decreasing	0% of 7	Entire range (S Atlantic, S Pacific)

*IUCN red list categories: LC = Least Concern, NT = Near Threatened, VU = Vulnerable, EN = Endangered. †Individuals recorded as *Fregetta grallaria* on Tristan du Cunha are now known to be a mix of *Fregetta tropica* and *Fregetta grallaria.* ‡Labelled as Little Shearwater in Ryan 1987.

Supplementary Table 4. | Research priorities for tracking and ingestion studies in areas with potential high or moderate exposure risk. Ordered by frequency of occurrence of ingested plastic across all studies from Kuhn & van Franeker, 2020[1] (>20%, no data, ≤20%) and IUCN red list category*. A total of 31 species, populations or non-breeding seasons that may overlap with areas identified as potentially having a high or moderate exposure risk for petrels but poor data coverage. Ingestion frequency of occurrence shows the percent of individuals found to contain plastic and the number examined.

Area	Common Name	Scientific name	Red List	Population trend	Ingested plastic frequency of occurrence	Priority tracking data gap (range also occurs in other priority areas)
**Southeast Asia**	Bonin petrel	*Pterodroma hypoleuca*	LC	Decreasing	40% of 72	Entire range (NE Pacific)
	Wilson's storm-petrel	*Oceanites oceanicus*	LC	Stable	34% of 244	Non-breeding season (S Atlantic, SW Indian Ocean, S Pacific, N Atlantic)
	Matsudaira's storm-petrel	*Hydrobates matsudairae*	VU	Unknown	No data	Entire range
- 7 species	Streaked shearwater	*Calonectris leucomelas*	NT	Decreasing	No data	SW part of their range
	Swinhoe's storm-petrel	*Hydrobates monorhis*	NT	Stable	No data	Entire range (also NW Pacific)
	Tropical shearwater	*Puffinus bailloni*	LC	Stable	No data	Pacific
	Bulwer's petrel	*Bulweria bulwerii*	LC	Stable	2% of 98	Pacific
**South Pacific**	White-faced storm-petrel	*Pelagodroma marina*	LC	Decreasing	79% of 34	Pacific
	Stejneger's petrel	*Pterodroma longirostris*	VU	Decreasing	67% of 72	Entire range (NE & NW Pacific)
	Collared petrel	*Pterodroma brevipes*	VU	Decreasing	67% of 3	Entire range
- 21 species	White-bellied storm-petrel	*Fregetta grallaria*	LC	Decreasing	32% of 37†	Entire range (S Atlantic, SW Indian Ocean)
	Wilson's storm-petrel	*Oceanites oceanicus*	LC	Stable	34% of 244	Non-breeding season (S Atlantic, SW Indian Ocean, SE Asia, N Atlantic)
	Rapa shearwater	*Puffinus myrtae*	CR	Decreasing	No data	Entire range
	Beck's petrel	*Pseudobulweria becki*	CR	Decreasing	No data	Entire range
	Fiji petrel	*Pseudobulweria macgillivrayi*	CR	Decreasing	No data	Entire range
	Henderson petrel	*Pterodroma atrata*	EN	Decreasing	No data	Entire range
	Masatierra petrel	*Pterodroma defilippiana*	VU	Stable	No data	Entire range
	Christmas Shearwater	*Puffinus nativitatis*	LC	Stable	20% of 44	Entire range (NE Pacific)
	Cape Petrel	*Daption capense*	LC	Stable	19% of 282	S Pacific (S Atlantic, SW Indian Ocean)
	Polynesian Storm-petrel	*Nesofregetta fuliginosa*	EN	Decreasing	0% of 14	Entire range
	Phoenix Petrel	*Pterodroma alba*	EN	Decreasing	0% of 30	Entire range
	Black-bellied Storm-petrel	*Fregetta tropica*	LC	Decreasing	0% of 7	Entire range (S Atlantic, SW Indian Ocean)
	Herald Petrel	*Pterodroma heraldica*	LC	Decreasing	0% of 12	Entire range
	Kermadec Petrel	*Pterodroma neglecta*	LC	Decreasing	0% of 19	Non-breeding season (NW, NE Pacific)
	Markham's Storm-petrel	*Hydrobates markhami*	NT	Decreasing	4% of 107	Entire range
	Tahiti Petrel	*Pseudobulweria rostrata*	NT	Decreasing	1% of 190	Non-breeding season
	Juan Fernandez Petrel	*Pterodroma externa*	VU	Stable	1% of 287	Entire range (S Pacific)
	Wedge-rumped Storm-petrel	*Hydrobates tethys*	LC	Decreasing	1% of 472	Entire range
**North West Atlantic**	Wilson's Storm-petrel	*Oceanites oceanicus*	LC	Stable	34% of 244	Non-breeding season (S Atlantic, SW Indian Ocean, SE Asia, S Pacific)
	Audubon's Shearwater	*Puffinus lherminieri*	LC	Decreasing	29% of 175	W Atlantic
- 5 species	Leach's Storm-petrel	*Hydrobates leucorhous*	VU	Decreasing	26% of 470	Non-breeding season
	Jamaican Petrel	*Pterodroma caribbaea*	CR	Unknown	No data	Entire range
	Cape Verde Storm-petrel	*Hydrobates jabejabe*	LC	Decreasing	No data	W Atlantic

*IUCN red list categories: LC = Least Concern, NT = Near Threatened, VU = Vulnerable, EN = Endangered. †Individuals recorded as *Fregetta grallaria* on Tristan du Cunha are now known to be a mix of *Fregetta tropica* and *Fregetta grallaria.*

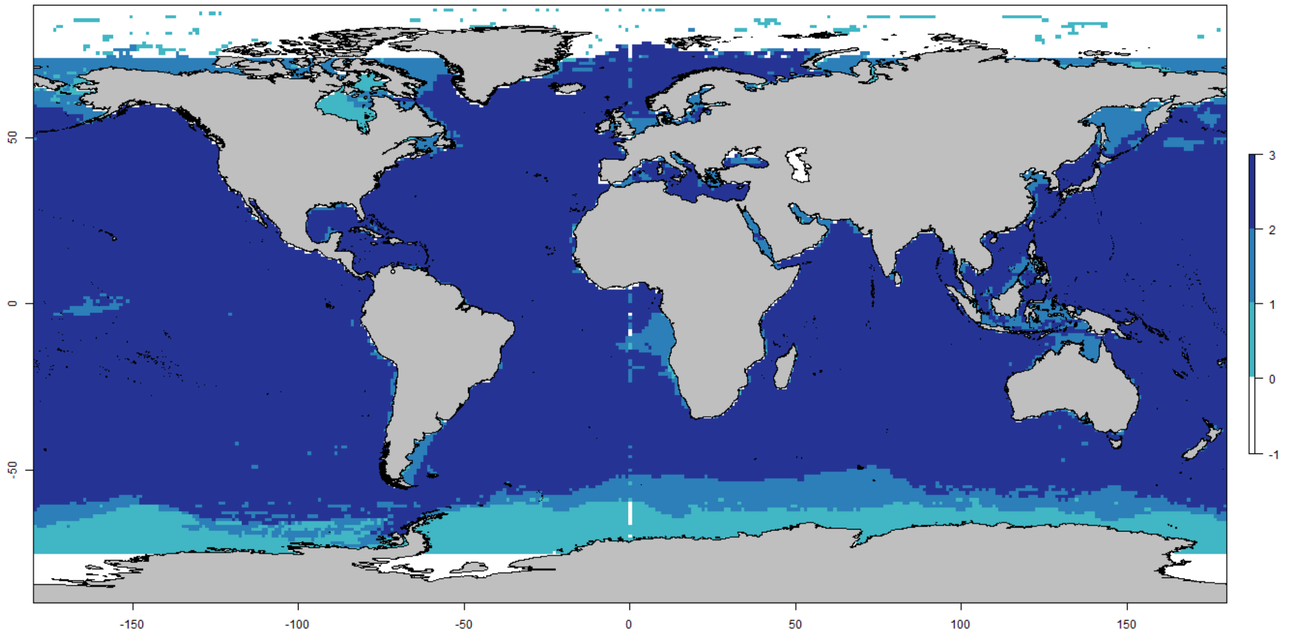

Supplementary Fig. 4 | Spatial coverage of plastic density estimates. Map of the number of plastic density model outputs (van Sebille, Lebreton and Maximenko) used to calculate the value for each cell. Land polygons from Natural Earth.

Supplementary Acknowledgements

We thank the following people and organisations in no particular order and all those who have helped with the collection and management of petrel tracking data. Animal ethics were managed by each organisation as appropriate.

Marie-Morgane Rouyer has received funding from the European Union’s Horizon 2020 research and innovation programme under the Marie Skłodowska-Curie grant agreement No 766417. This communication reflects only the authors’ view and the Research Executive Agency of the European Union is not responsible for any use that may be made of the information it contains.

Win Cowger was funded in part by the National Science Foundation Graduate Research Fellowship.

Tammy Davies, Jonathan Handley and Martin Beal for advice on data collation and presentation.

David Pascall for advice on data analysis.

Norman Ratcliffe of British Antarctic Survey for assistance with the Round Island petrel tracking project.

Graeme Loh for assistance with tracking Broad-billed Prions on Rangatira and Fairy Prions at Dunedin.

Yasuko Suzuki for translation of streaked shearwater populations estimates in Japan.

Trevor Glass for assistance with Great Shearwater tracking (Tristan da Cunha).

Mandy Shailer for help with Bermuda Petrel tracking.

Linda Wilson for assistance accessing data via the Royal Society for the Protection of Birds.

The Australian Antarctic Division for logistics and funding through Australian Antarctic Science project #4087 to Louise Emmerson, the Flemish Science Foundation (FWO 12Q6915N, V458215N and V416817N) and the University of Antwerp (BOF/KP 2015 #31032) to Nina Dehnhard.

The Madeira Institute for Forest and Nature Conservation for permission to visit Cima Islet and Selvagem Grande, help with boat trips and accommodation. Financial support for the fieldwork on Cima Islet was provided by the EU project LIFE09NAT/PT/000041. Fieldwork on Selvagem Grande was partially funded by a British Ornithologists’ Union (BOU) small grant from 2011.

The New Zealand Department of Conservation, Ministry for Primary Industries and Ngāti Rehua Ngāti Wai ki Aotea for permission to release the Black Petrel data.

Tracking funding/support from my contributions include: BirdLife, US Fish and Wildlife Service, Killam Trust of Dalhousie University, NSERC, Environment and Climate Change Canada.

SEATRACK (https://seapop.no/en/seatrack) for aiding with Northern Fulmar tracking data.

For Barau’s Petrel, Mascarene petrel and Wedge-tailed Shearwater data from Réunion, Mauritius and Seychelles, the Pew Environment Group (Pew Fellowship Award in Marine Conservation of M. Le Corre and the European Commission (LIFE+Petrel Project Grant n° LIFE13 BIO/FR/000075).

Paul Sagar for assistance with GLS tracking White-chinned Petrels on the Antipodes Islands.

The tracking data for White-chinned Petrel, Grey Petrel, Thin-billed Prion, Antarctic Prion, Blue Petrel, Cape Petrel, Southern Fulmar and Snow Petrel were collected with logistical support and funding from the French Polar Institute (IPEV) and with funding from Fondation Prince Albert II de Monaco. We also thank Terres Australes et Antarctiques Françaises.

For seabirds from the Terres Australes et Antarctiques Françaises, the work was supported financially and logistically by the French Polar Institute IPEV (program N°109, Christophe Barbraud).

L.R.H. was supported with an Endeavour Postgraduate Leadership Award by the Australian government and with grants awarded by the Holsworth Wildlife Research Endowment Equity Trustees Charitable Foundation and the Ecological Society of Australia, the Birdlife Australia Stuart Leslie Bird Research Award.

Direção Regional do Ambiente, Região Autónoma dos Açores for granting access to Praia Islet (HFRH licence number: 40/2019/DRA, SAI-DRA/2019/1821 Proc. 116.14.03/43 and 83/2020/DRA - SAI-DRA/2020/3569 Proc. 116.14.03/43) and also Graciosa Natural Park Environmental Service for helping us access and stay on Praia Islet. Many thanks to fieldwork assistants, Kirsty Jones, Jonathon M. Biddle, Sarah E. Long, Dimitri Dujardin, Jodie M. Henderson, Alice J. Edney and Max A. Levy.

The GPS tracking data for Scopoli's Shearwaters breeding in La Maddalena Archipelago in 2018 and 2019 were collected with funding from Parco Nazionale dell'Arcipelago di La Maddalena. The GPS tracking of European storm petrels breeding in Porto Conte was partially funded the Ministero Italiano per la Transizione Ecologica.

Direção Regional do Ambiente, Região Autónoma dos Açores, for granting access to Vila Islet (JB licence numbers 2/2006/DRA, 25/2008/DRA, 20/2011/DRA, 19/2012/DRA), and the Clube Naval from Santa Maria for transportation to Vila islet. Cory's shearwater PTT data at Vila was collected under Dr Keith Hamer's supervision.

Projects: LIFE Spanish marine IBAs (2004-2009); LIFE+ INDEMARES (2009-2014); Interreg FAME (2010-2013)

The St Helena Nature Conservation Group (SNCG) and the St Helena National Trust for their help during fieldwork. Thanks also go to the Terrestrial Conservation Section of St Helena Government for continued support on the Egg Island Restoration Programme. We appreciate the financial support given though the Darwin Plus Initiative (DPLUS070) project Oceanographic influences on the St Helena pelagic ecosystem and the Department for Environment, Food and Rural Affairs (DEFRA). Analysis was facilitated by the Royal Society for the Protection of Birds (RSPB) and British Antarctic Survey (BAS).

The Mauritian Wildlife Foundation, Mauritius Government National Parks and Conservation Service for supporting the collection of Round Island Petrel data, and to our funders, the Natural Environment Research Council.

Brazilian National Research Council - CNPq; CAPES Foundation; American Bird Conservancy (funders); Gustavo Leal, Gabriela Oliveira for field work support.

The GPS tracking data for the Bermuda petrels were collected with funding from the Mohamed Bin Zayed Species Conservation Fund (project number 182520049), the DENR Government of Bermuda and ECC Canada.

The Korean Streaked Shearwater tracking program was supported by the Korean National Park Service.

This study had the support of national funds through Fundação para a Ciência e Tecnologia, I. P (FCT), under the projects UIDB/04292/2020, UIDP/04292/2020, granted to MARE, and LA/P/0069/2020, granted to the Associate Laboratory ARNET. FCT also funded the transitory norm contract DL57/2016 to FRC (DL/2016/CP1370/CT90) and the CEEC contract to VHP (2021.01812.CEECIND).

Fundação para a Ciência e Tecnologia/MCTES provided financial support to CESAM (UIDP/50017/2020 and UIDB/50017/2020), through national funds. T.C. was supported by FCT through contract IF/00694/2015.

The Contribution of the Government of Mauritius though the National Parks and Conservation.

The GPS tracking data for Pink-footed Shearwaters were collected with assistance from Verónica López and funding from the National Fish and Wildlife Foundation and additional support from the US Geological Survey, Western Ecological Research Center, Ecosystems Mission Area.

ZD was supported by a NERC GW4+ Doctoral Training Partnership studentship [NE/L002434/1] and a grant from the Seabird Group.

This monitoring program was supported financially and logistically by the French Polar Institute IPEV (program 109, resp. Henri Weimerskirch), the Zone Atelier Antarctique (CNRS-INEE), Terres Australes et Antarctiques Françaises. All work was carried out in accordance with the IPEV ethics committee.

BJD acknowledges mana whenua iwi for their support of tracking common diving petrels.

The Wildlife Trust for South and West Wales and the Skomer & Skokholm Islands Advisory Council, the Copeland Bird Observatory, the RSPB, Scottish National Heritage and the National Trust for Scotland, and the Landmark Trust, for permission to work on Skomer, Copeland, Ramsey, Rum and Lundy Islands, respectively. This work was supported by Microsoft Research Cambridge, the Northern Ireland Environment Agency, the RSPB, Scottish Natural Heritage, the Birds of Lundy Fund and the University of Oxford, and funding from the Mary Griffiths Award.

JHF, GAT, and HUW thank Kāi Tahu and the Whenua Hou Komiti for granting data collection and permitting data release.

Karry-Jayne Wilson for assistance to Amanda Freeman.

The 2008 GPS tracking data for Scopoli's shearwaters breeding in Linosa were collected with funding from the Ministero italiano per la Transizione Ecologica. LIPU-UK funded the GPS tracking of Scopoli's shearwaters carried out in Tremiti Archipelago (2009-2010), in Linosa (2009, 2012), in Tuscany Archipelago (2010-2011) and in La Maddalena Archipelago (2011 and 2013). The Scopoli's shearwater GPS tracking activity carried out in Tuscany Archipelago was conducted within the framework of Progetto Co.R.E.M.-Programma di Cooperazione transfrontaliera Italia/Francia"Marittimo" 2007-2013 funded by European Commission, with the support of Parco Nazionale dell'Arcipelago Toscano and Centro Ornitologico Toscano (COT).

Veiðikortasjóður funded our research in Iceland.

The European Union for funds through the Seventh Framework Programme (Research Executive Agency of the European Commission, 618841, FP7-PEOPLE-2013-CIG).

Spanish funds through the Ministerio de Ciencia y Tecnología (REN2002-01164/GLO), Ministerio de Educación y Ciencia (CGL2006-01315/BOS), Ministerio de Ciencia e Innovación (CGL2009-11278/BOS), and Ministerio de Economía y Competitividad (CGL2013-42585-P); Ministerio de Economia, Industria y Competitividad (CGL2016-78530-R).

Catalan funds through the Generalitat de Catalunya (2001SGR00091; 2014 SGR 1155; 2017SGR1611); Programa ICREA (2019-2023).

Additional funding from SEO/BirdLife (programa Migra & proyecto LIFE+ Indemares), Fundación Banco Bilbao Vizcaya Argentaria (BIOCON04/099) and Fundación Biodiversidad (18PCA4328; 2019/1423; Pleamar 2017; Pleamar 2019); MAVA Foundation (MAVA17022; 4880; 20210/20033).

Raül Ramos was supported by postdoctoral contracts by Beatriu de Pinós (2010-BP_A-00173), Juan de la Cierva (JCI-2009-05426), PLEAMAR (2017/2349), and funding for a postdoctoral Ramón y Cajal contract of the Spanish Ministry of Economy, Industry and Competitiveness (RYC-2017-22055).

Leia Navarro-Herrero was supported by predoctoral contract BES-2017-079874 of the Spanish Ministerio de Industria, Economía y Competitividad.

Fernando Medrano was supported by a predoctoral grant DoctoradoBecasChile/2019-72200117 of the Agencia Nacional de Investigación y Desarrollo of Chile (ANID).

Diego Vicente-Sastre was supported by Predoctoral contract BDNS 481561 (file number 2020 FISDU 463) of the Agència de Gestió d'Ajuts Universitaris i de Recerca (AGAUR, Generalitat de Catalunya).

FCT/MCTES for the financial support to CESAM (UIDP/50017/2020+UIDB/50017/2020), through national funds.

The work was supported financially and logistically by the French Biodiversity Agency, the Parc National de Port Cros et Porquerolles, and by the Region Nouvelle Aquitaine through the Excellence Chair ECOMM administred by David Grémillet.

Sooty Shearwaters: Helen Otley, Alastair Baylis, Robin Snape, Anton Wolfaardt, Sally Poncet, Nick Rendell and Falklands Conservation for field and logistic support, and Dave Fifield and Paul Regular for analytical support. Project funding through an NSERC Discovery Grant and the Government of Canada’s Program for International Polar Year to W.A. Montevecchi.

Leach's Storm-Petrels: Chantelle Burke, Marina Montevecchi, Michelle Valliant and Dave Fifield for field support. Project funding through Environment and Climate Change Canada.

The Direção Regional do Ambiente, Região Autónoma dos Açores for granting access to Praia Islet (HFRH licence number: 40/2019/DRA, SAI-DRA/2019/1821 Proc. 116.14.03/43 and 83/2020/DRA - SAI-DRA/2020/3569 Proc. 116.14.03/43) and also Graciosa Natural Park Environmental Service for helping us access and stay on Praia Islet. Many thanks to fieldwork assistants: Sarah E. Long, Jodie M. Henderson, Alice J. Edney and Max A. Levy.

All fieldworkers for data collection, in particular Saskia Wischnewski and Ashley Bennison. GPS tracking data for Manx Shearwaters and European Storm-petrels (GOIPD/2015/81) in Ireland was funded by the Irish Research Council and Petroleum Infrastructure Programme.

L.K. is supported by the Marine Protected Areas program of the Instituto Antártico Chileno.

Data was collected for Maltese seabirds (2013-2018) under BirdLife Malta’s EU-LIFE + Malta Seabird Project (LIFE10NAT/MT/090) and EU-LIFE Arċipelagu Garnija (LIFE14NAT/MT/000991), both co-funded by the LIFE programme of the European Commission and the Maltese Ministry for the Environment, Sustainable Development and Climate Change, in partnership with the Royal Society for the Protection of Birds, the Portuguese Society for the Study of Birds and Transport Malta.

Detached Cultural Organization funding to Jennifer Lavers.

Fieldwork at New Island was supported by the New Island Conservation Trust with assistance.

from Ian, Maria and Georgina Strange. The work was approved by the Falkland Islands Government (Environmental Planning Office) and funded by grants provided by the German Science Foundation DFG (Qu 148/5).

The PhD of FM is granted by the Agencia Nacional de Investigación y Desarrollo of Chile (ANID)/DoctoradoBecasChile/2019-72200117

M Tarita da Silva, T Chateaugirond, J Tayor-Bruce, M Austad, T Karwinkel, A Sargent, D Dujardin, L Schröder, M Cuesta and K Jones for help with fieldwork. We also thank the director and team of Graciosa Natural Park for access to the islet and all the logistical support. Fieldwork was conducted under permits from the Regional Directorate for the Environment, Azores Government (Licences #67/2018/DRA, #37/2019/DRA and #83/2020/DRA).

The Round Island petrel tracking programme has been conducted by numerous staff and volunteers from the Mauritian Wildlife Foundation (MWF) and the National Parks and Conservation Service (NPCS) over the last 12 years. Particular thanks go to Vimul Nundlaul, Richard Baxter, Pat Banville, Lucy Rouse, Helen Gath, Nik Cole and Johannes Chambon. The petrel tracking programme was supported by the Natural Environmental Research Council (UK) (Grant NE/H5081500) and Research England, with in-situ support from MWF & NPCS.

Pitcairn Island Conservation Department for granting access and permission to track Murphy's Petrels.

All fieldworkers who collected data as part of the RSPB FAME/STAR tracking projects: C. Bell, M. Chimienti, D. Fox, R. Hughes, J. Lamb, R. Langton, T. Newman, M. Nydegger, Y. Satgé, Sturgeon, C. Taylor, J. Taylor and others. Funding was provided by the European Regional Development Fund through its Atlantic Area Programme, Marine Scotland, Scottish Natural Heritage, Natural England, the Natural Environment Research Council (NE/M017990/1), the Joint Nature Conservation Committee, Natural Resources Wales, Environment Wales, Argyll Bird Club, Fair Isle Bird Observatory Trust, and the RSPB.

Work on Wilson's storm-petrels was funded by the German Ornithologist`s Society (Deutsche Ornithologen-Gesellschaft - DO-G) and the DFG grant Qu148/18 in the Priority Programme 1158 "Antarctic Research with Comparative Studies in Arctic Ice Regions". The Alfred Wegener Institute, Germany and the Instituto Antártico Argentino (IAA) and the Argentinian Dirección Nacional del Antártico (DNA), funded transport and accommodation and provided infrastructure in Antarctica.

For Bon Portage Island GLS and GPS data, Lee Adams, Zoe Crysler, Danielle Fife, Rielle Hoeg, and Erika Holland. For Country Island GLS data, Sarah N.P. Wong and Environment and Climate Change Canada field crews. For Eastern Shore Island GLS data, Ellen Boyd, and Molly D. Tomlik. Funding was provided through a NSERC post-graduate scholarship, NSERC Collaborative Research and Development grant, and Nova Scotia Habitat Conservation Fund.

Steve Stansfield, Mark Carter, Ephraim Perfect and Emma Stansfield for their invaluable help in the field in carrying out GPS tracking work on Manx shearwaters on Bardsey Island, and also Bardsey Island Trust and Natural Resources Wales for the permission to carry out this work on the island.

We thank the Island Conservation Society, Fondation Total, James Cadbury Foundation, and Miguel Torres wineries for funding the Shearwater Research Programme on Aride Island Nature Reserve, and its staff and volunteers for their help in the field.

For Seychelles Wedge-tailed and Tropical shearwater data, the ICS (Island Conservation Society). We thank Melinda Curran (ICS Assistant Conservation Officer), ICS Conservation Rangers and volunteers

from Aride Island for their assistance in the field; Fondation TOTAL, James Cadbury, Fundació MIGUEL TORRES and CNRS for providing funds; Pierre-André Adam and the ICS Head Office Management Team for their support in coordinating and facilitating the administration procedures associated with this research.

Movebank for aiding with the Galapagos petrel tracking.

Support from LIFE NAT B-4/3200/97/246.

Serra Gelada Natural Park and Fundación Balearia. Funds were provided by the project CNME19/0301/39 from the Generalitat Valenciana and the project CGL2017-85210-P from the Spanish Minister of Science, Innovation and Universities. ASA was supported by a “ramon y Cajal” fellowship (RyC-2017-22796).

Funding provided by the Neotropical Bird Club, the South Carolina Cooperative Fish and Wildlife Research Unit, and Grupo Jaragua. Field assistance was provided by Pirrín Jairo Matos, Gerson Feliz, José Luis Castillo and Ivan Terrero. We are grateful to Voltaic Systems and Jeff Crystal for their financial and technical support and for providing refurbished solar panels for the base-stations, and to Brenna Byler and Miguel Abreu for managing international orders.

Data collection for Sooty Shearwaters was partially supported by the Tagging of Pacific Predators (TOPP) program.

MCSilva received funding from Fundação para a Ciência e a Tecnologia (FCT) through the cE3c Unit UIDB/BIA/00329/2020 and a contract under ns. 4–6 of article 23 of DL 57/2016, changed by Law 57/2017.

The Houtman Abrolhos community in Australia for their support.

The Lusk and Woodward families for permission to use their seabird islands as study sites and for accommodation in New Zealand.

The Orkney Islands Council for access to Eynhallow.

The European Union BEST 2.0 project “Biopelagos” (grant number 1070–2015 to SPC), the Province Nord (grant number 15C493), OFB (French Biodiversity Office), New Caledonia Gouvernment, Ecole Doctorale du Pacifique and Université de la Nouvelle-Calédonie

Institut Polaire Français IPEV.

The work at Awashima Island was approved by the Animal Experimental Committee of Nagoya University and the Ministry of the Environment Government of Japan. Funding from the Grants-in-Aid for Scientific Research from the Japan Society of the Promotion of Science (19H01157, 16H06541, 21H05294, 24681006).

The Pacific Seabird Group - Craig S. Harrison Conservation Fund 2011.

JA and JJF were supported in part by the US Geological Survey Ecosystems Mission Area. We thank the USGS reviewer for providing review. The use of trade, product, or firm names in this publication is for descriptive purposes only and does not imply endorsement by the US Government.

Supplementary Fig. 5 | Plastic exposure maps for the breeding and non-breeding (if available) seasons for each population. Pale yellow shows the marine area covered by the plastic density models and darker colours indicate higher exposure to marine plastic pollution. The exposure score is calculated by summing the exposure in all cells in the presented map. Blue diamonds show the study colonies for the tracked petrels. Species are in alphabetical order by scientific name.

Flesh-footed Shearwater Ardenna carneipes, New Zealand
Breeding season plastic exposure score = 51.62

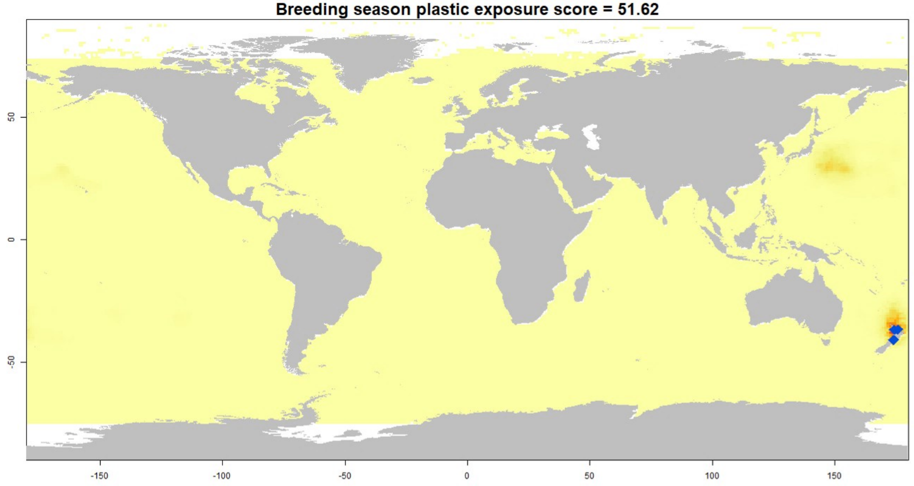

Pink-footed Shearwater Ardenna creatopus, Isla Mocha and Juan Fernandez
Breeding season plastic exposure score = 3.45

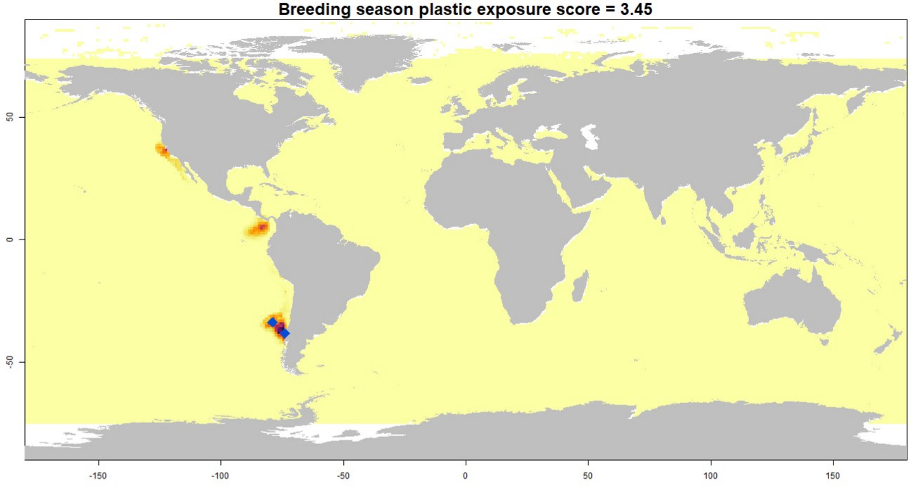

Flesh-footed Shearwater Ardenna carneipes, Lord Howe Island
Non-breeding season plastic exposure score = 35.03

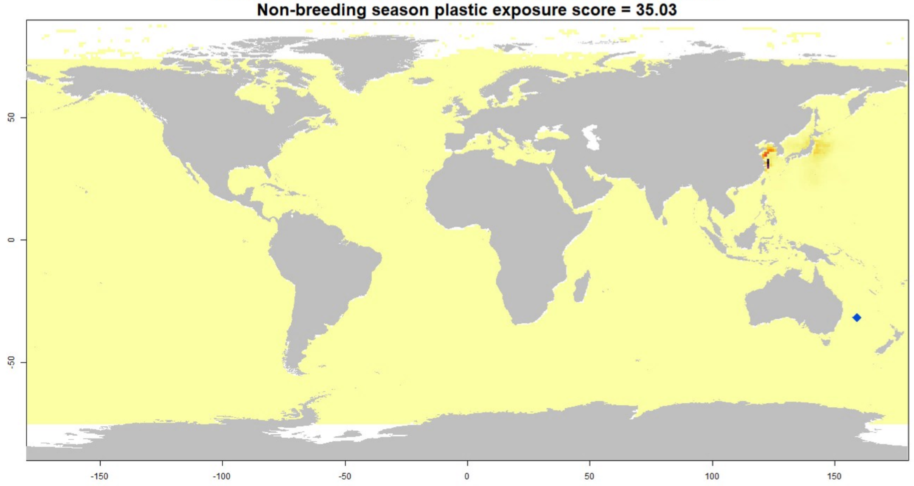

Flesh-footed Shearwater Ardenna carneipes, New Zealand
Non-breeding season plastic exposure score = 51.62

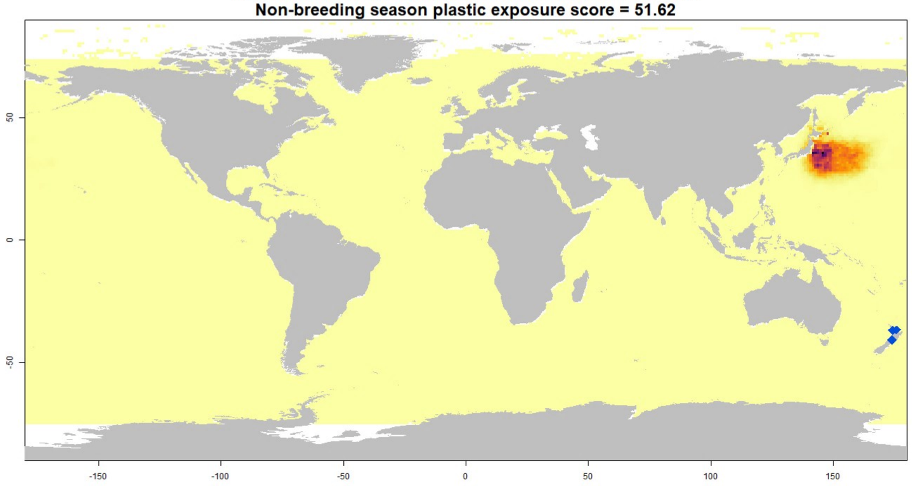

Great Shearwater Ardenna gravis, Gough
Breeding season plastic exposure score = 95.96

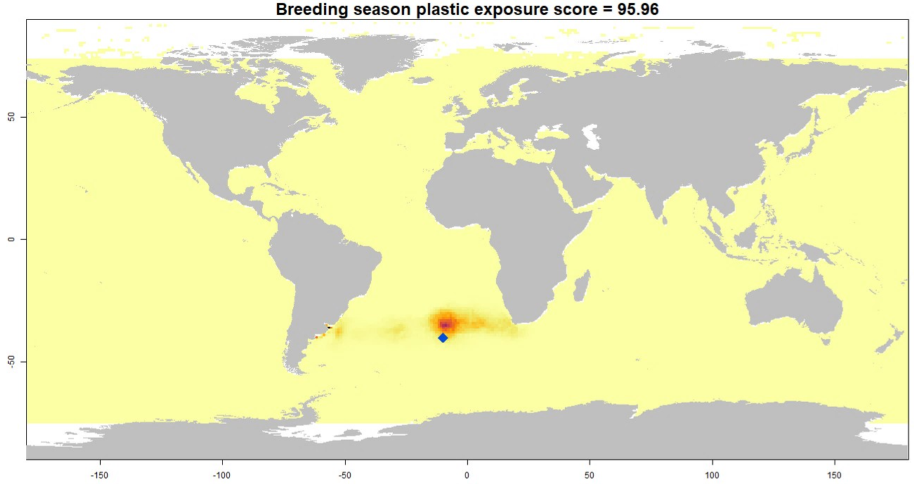

Great Shearwater Ardenna gravis, Tristan da Cunha
Breeding season plastic exposure score = 33.41

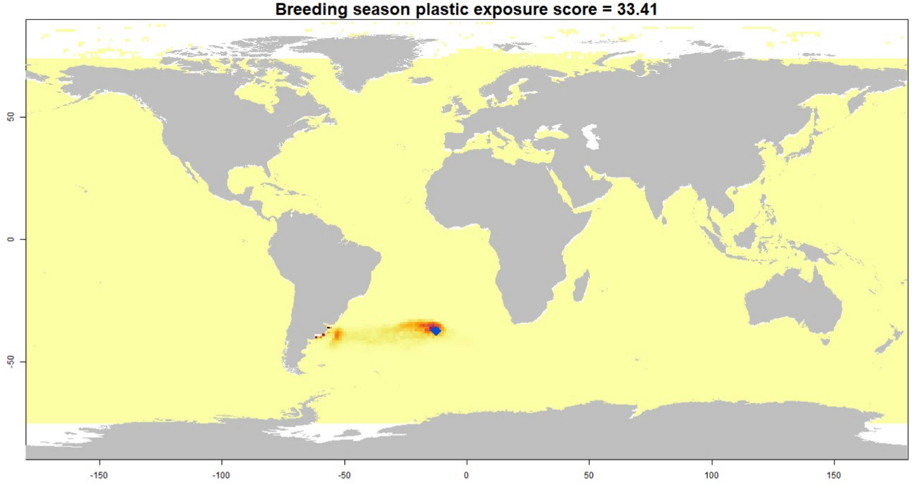

Pink-footed Shearwater Ardenna creatopus, Isla Mocha and Juan Fernandez
Non-breeding season plastic exposure score = 3.45

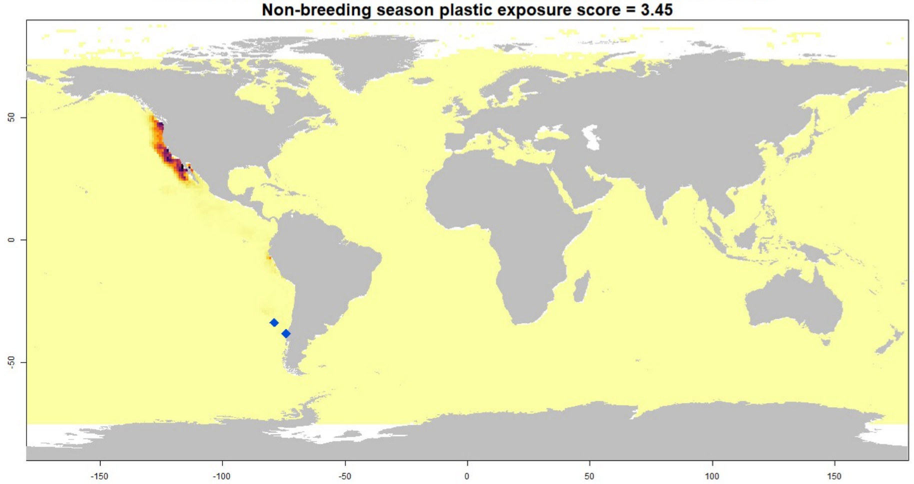

Great Shearwater Ardenna gravis, Gough
Non-breeding season plastic exposure score = 95.96

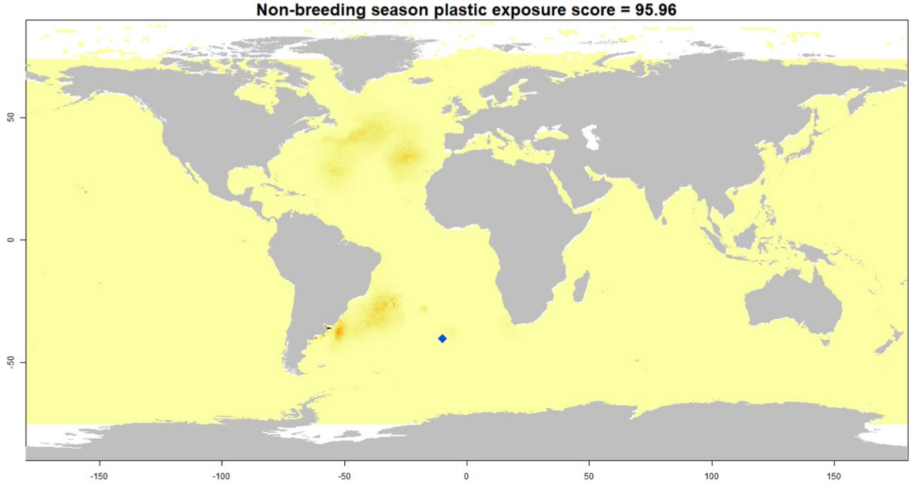

Sooty Shearwater Ardenna grisea, Codfish Island
Breeding season plastic exposure score = 35.12

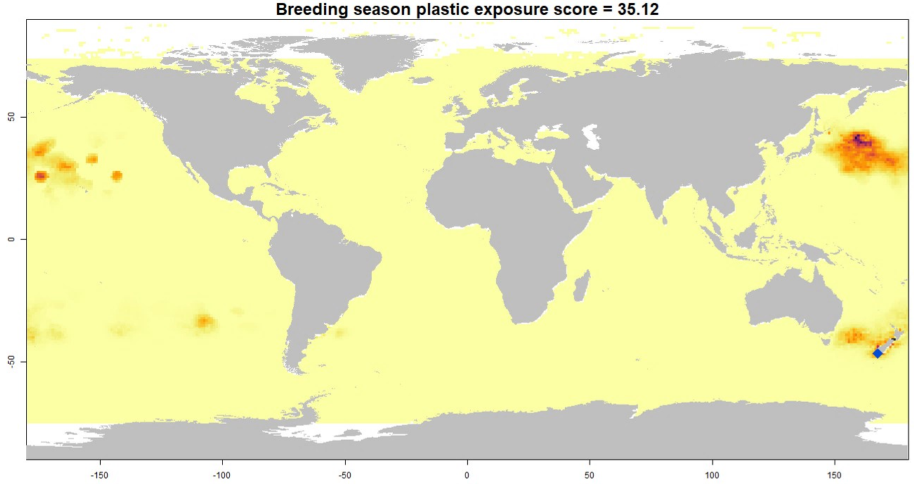

Sooty Shearwater Ardenna grisea, Falkland Islands
Breeding season plastic exposure score = 5.69

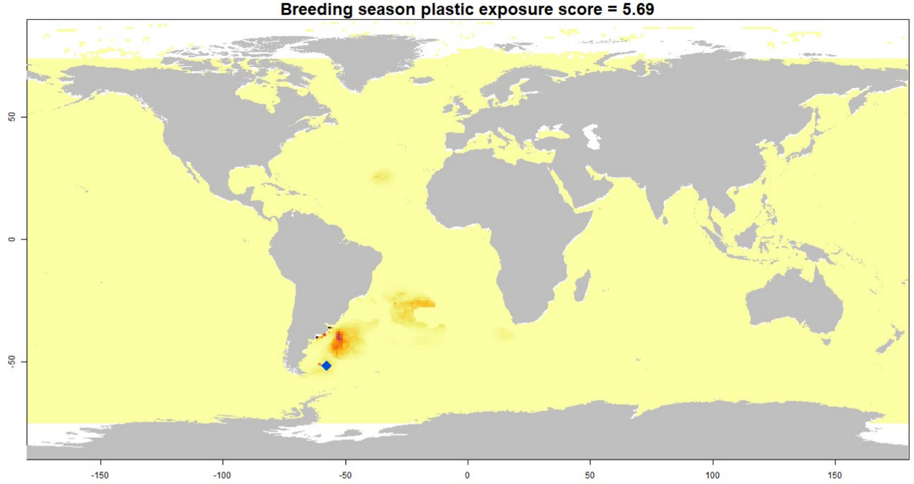

Great Shearwater Ardenna gravis, Tristan da Cunha
Non-breeding season plastic exposure score = 33.41

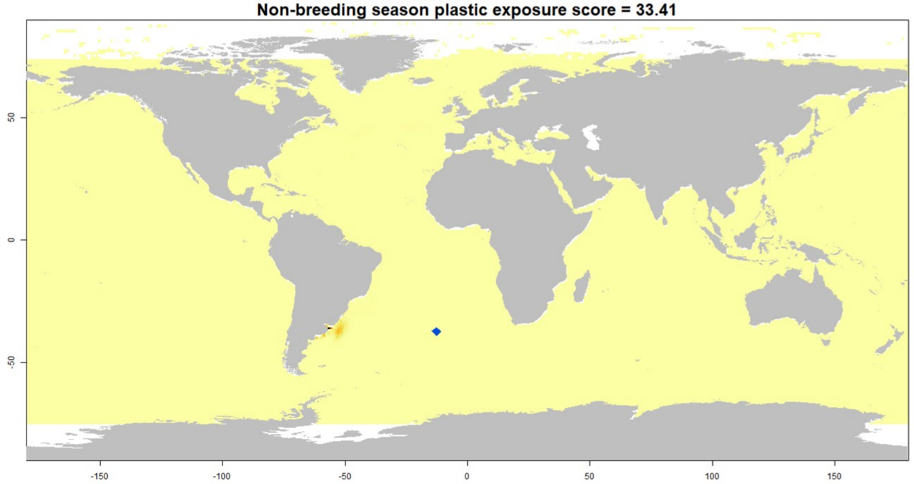

Sooty Shearwater Ardenna grisea, Codfish Island
Non-breeding season plastic exposure score = 35.12

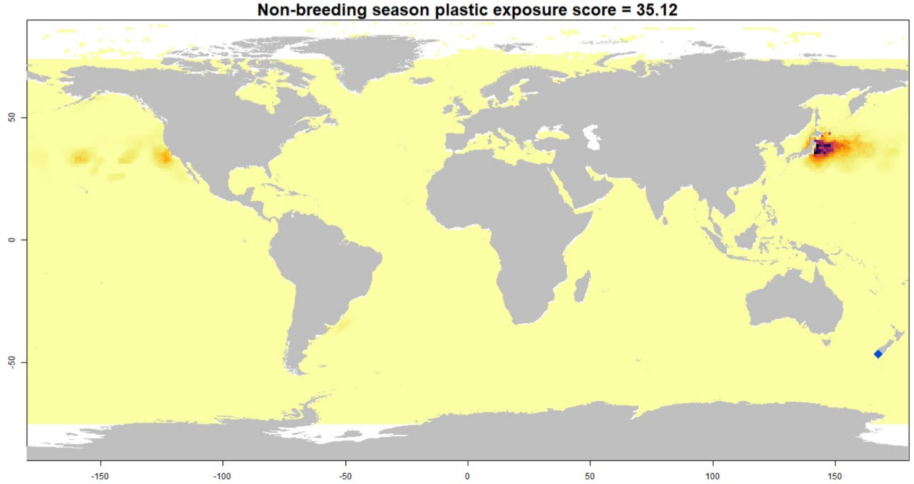

Sooty Shearwater Ardenna grisea, North Island
Breeding season plastic exposure score = 35.77

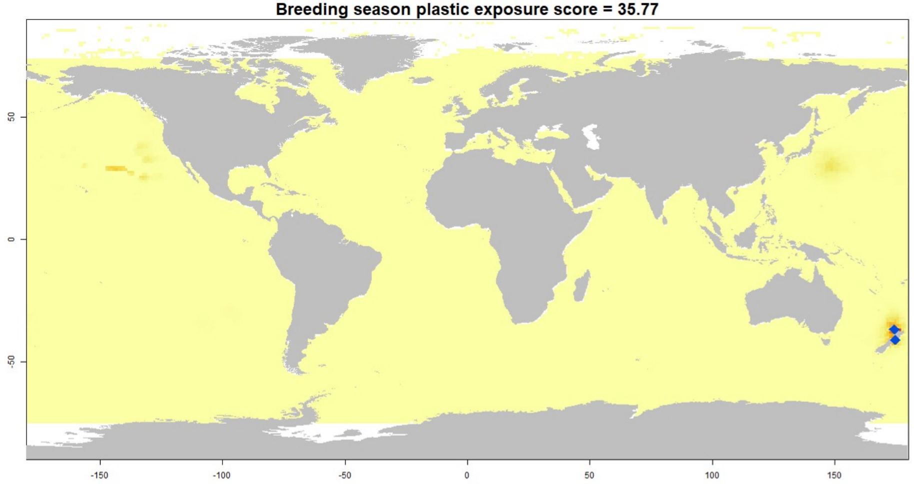

Wedge-tailed Shearwater Ardenna pacifica, Hawaii
Breeding season plastic exposure score = 220.28

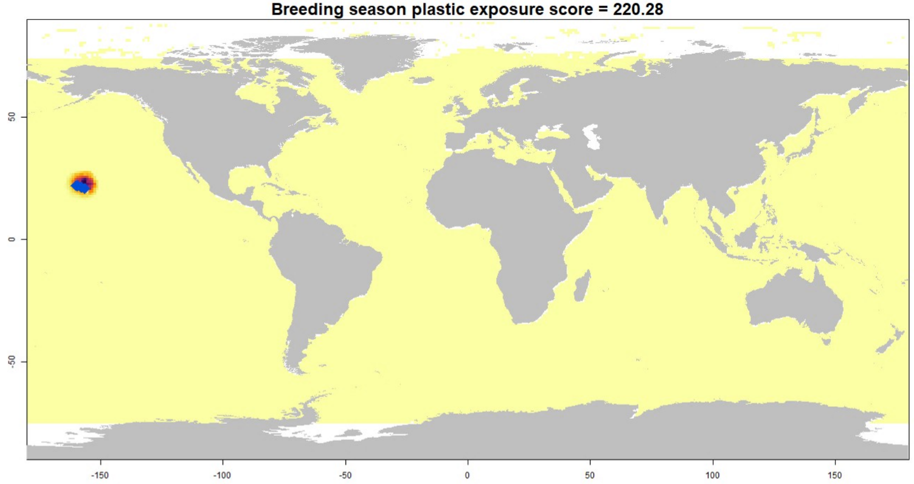

Sooty Shearwater Ardenna grisea, Falkland Islands
Non-breeding season plastic exposure score = 5.69

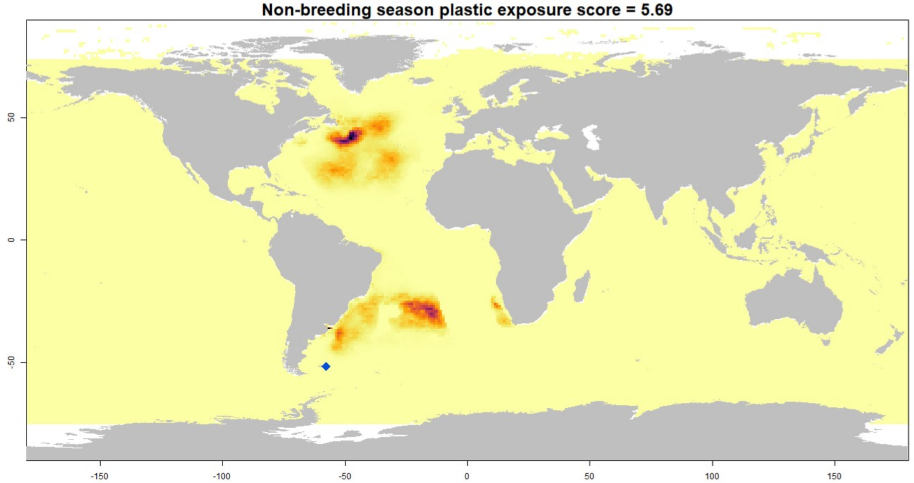

Sooty Shearwater Ardenna grisea, North Island
Non-breeding season plastic exposure score = 35.77

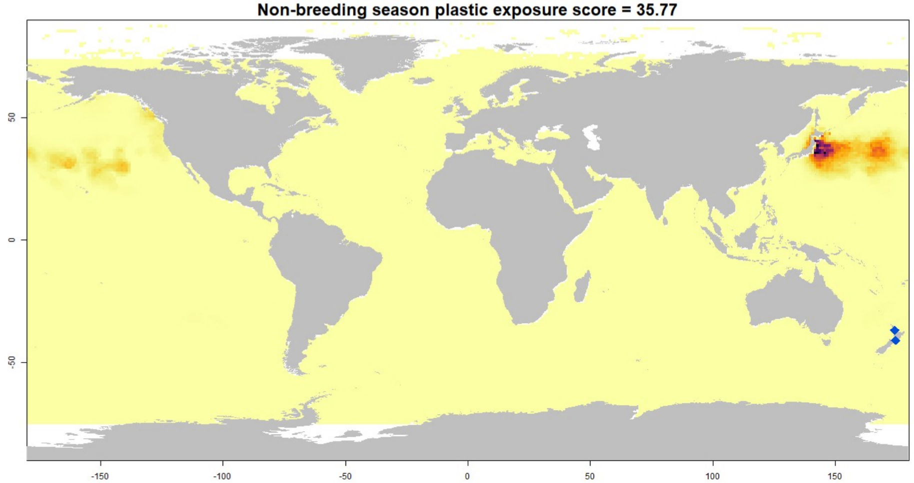

Wedge-tailed Shearwater Ardenna pacifica, Lowendal & Houtman Abrolhoss
Breeding season plastic exposure score = 16.61

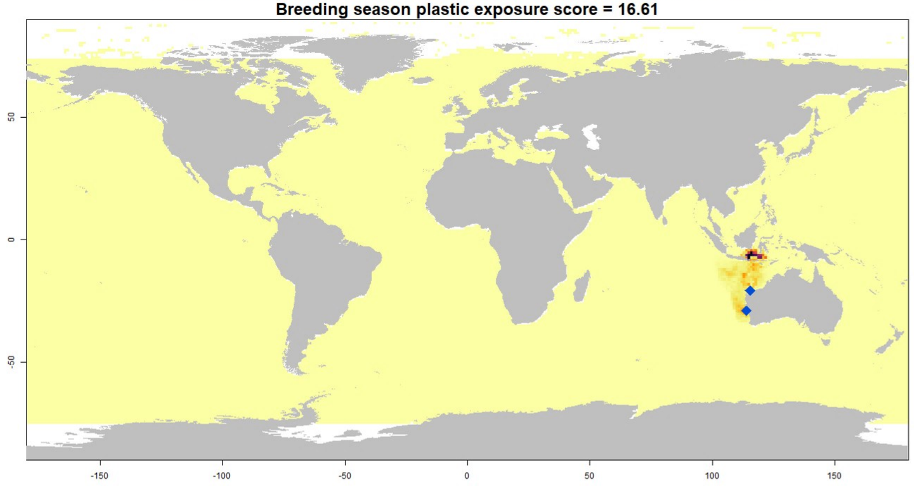

Wedge-tailed Shearwater Ardenna pacifica, New Calendonia
Breeding season plastic exposure score = 3

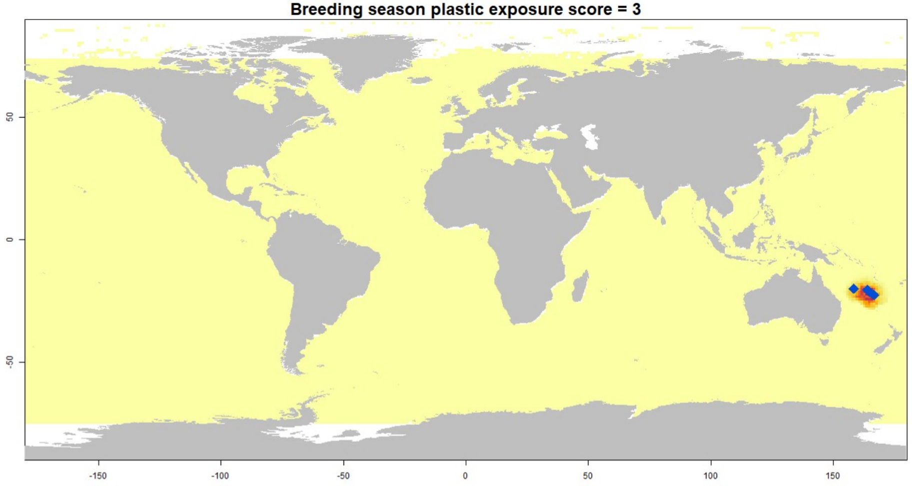

Wedge-tailed Shearwater Ardenna pacifica, Heron Island
Breeding season plastic exposure score = 45.23

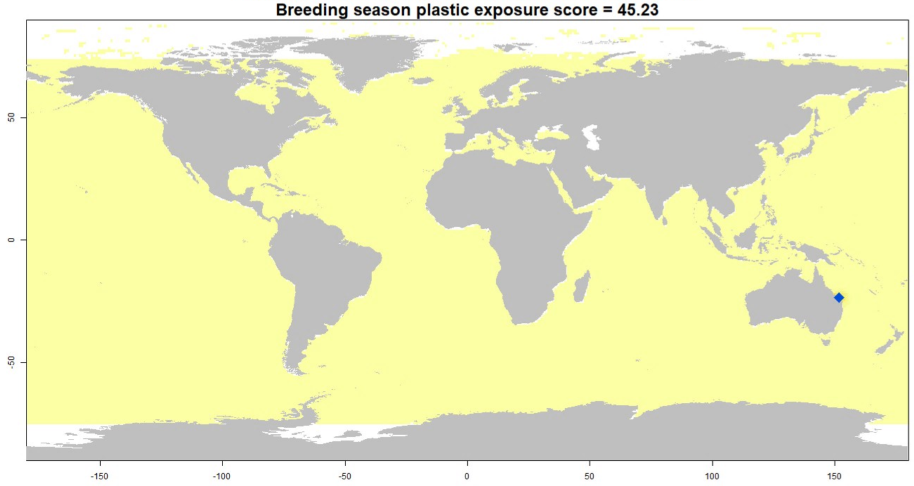

Wedge-tailed Shearwater Ardenna pacifica, Lowendal & Houtman Abrolhoss
Non-breeding season plastic exposure score = 16.61

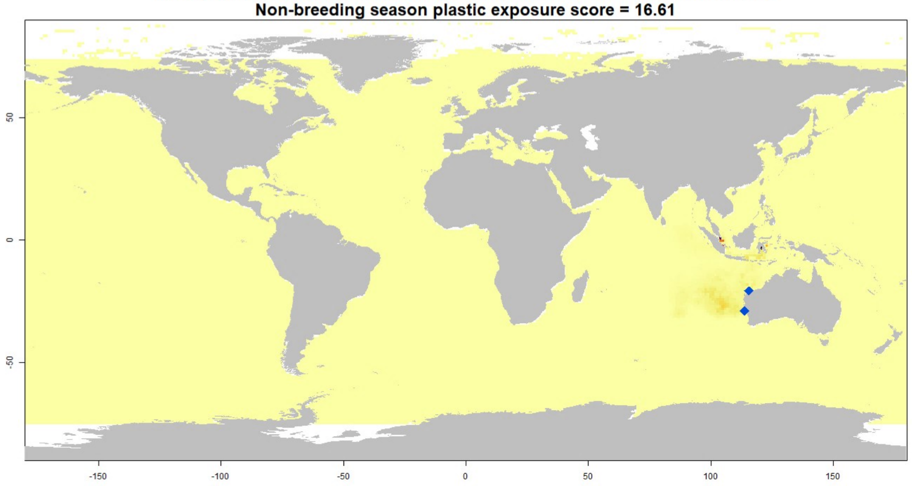

Short-tailed Shearwater Ardenna tenuirostris, SE Australia
Non-breeding season plastic exposure score = 15.26

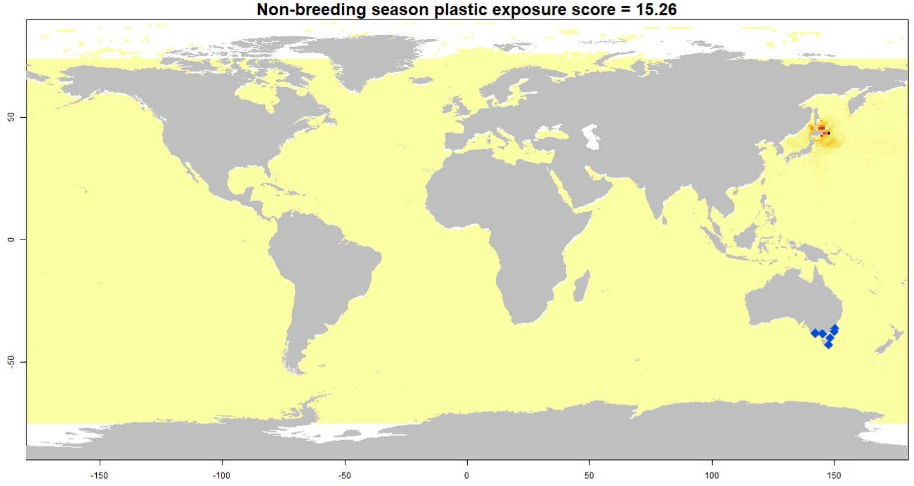

Bulwer's Petrel Bulweria bulwerii, Azores
Non-breeding season plastic exposure score = 52.73

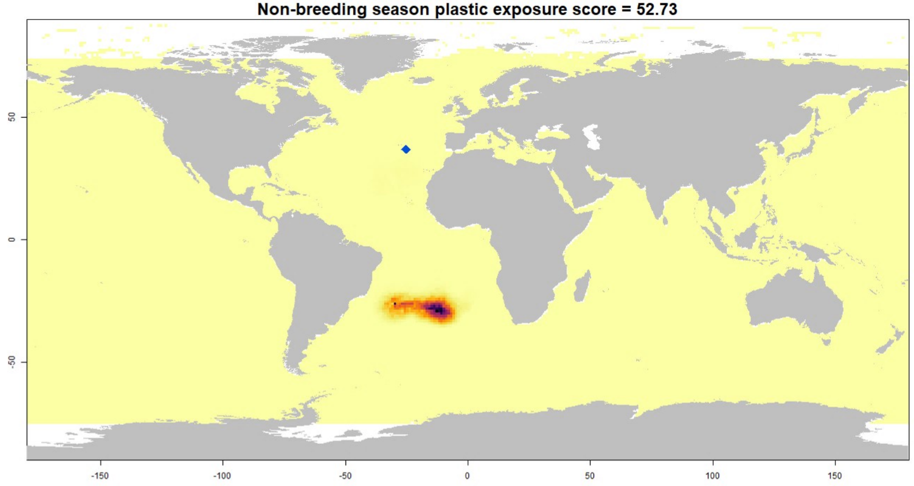

Short-tailed Shearwater Ardenna tenuirostris, SE Australia
Breeding season plastic exposure score = 15.26

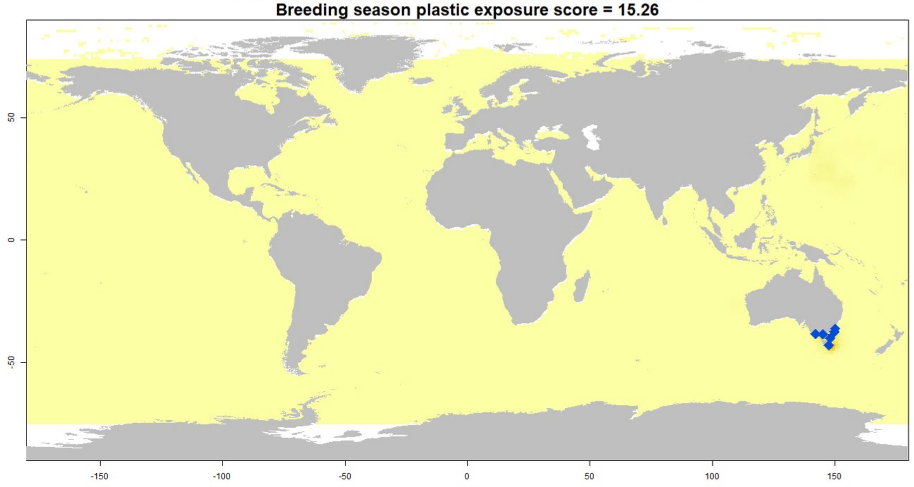

Bulwer's Petrel Bulweria bulwerii, Azores
Breeding season plastic exposure score = 52.73

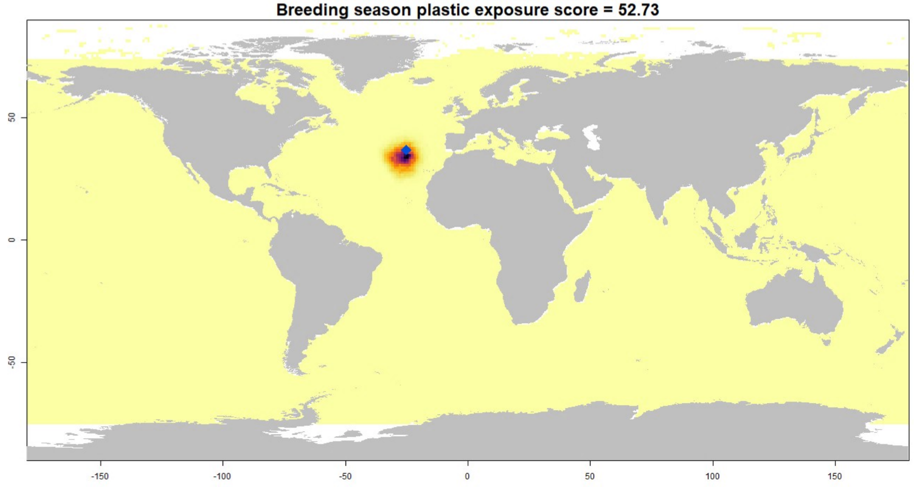

Bulwer's Petrel Bulweria bulwerii, Canarias
Non-breeding season plastic exposure score = 25.05

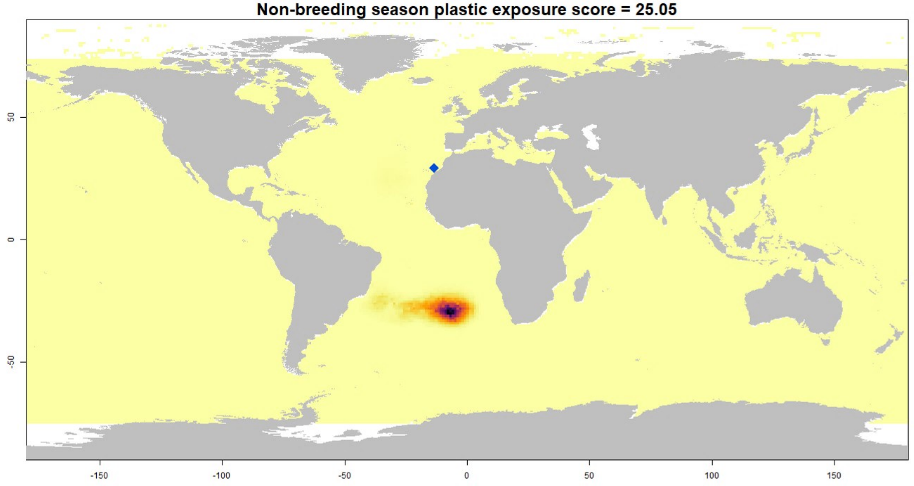

Bulwer's Petrel Bulweria bulwerii, Cape Verde
Non-breeding season plastic exposure score = 1.83

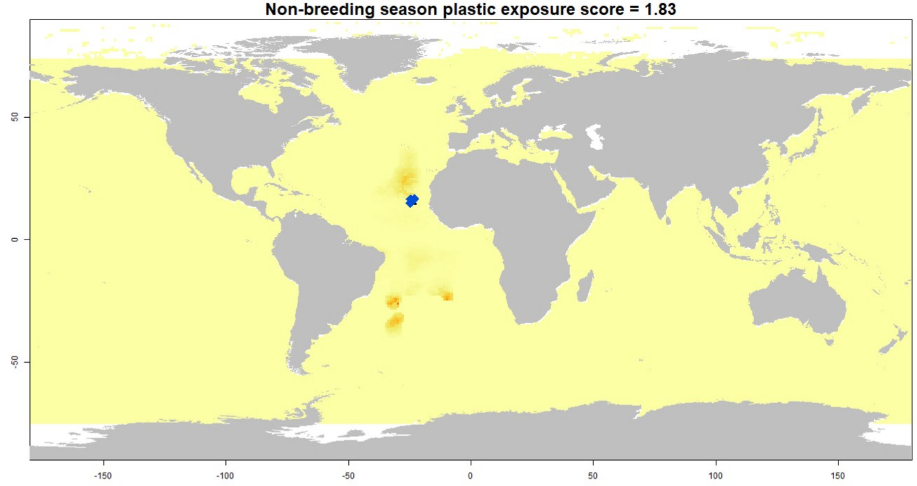

Bulwer's Petrel Bulweria bulwerii, Canarias
Breeding season plastic exposure score = 25.05

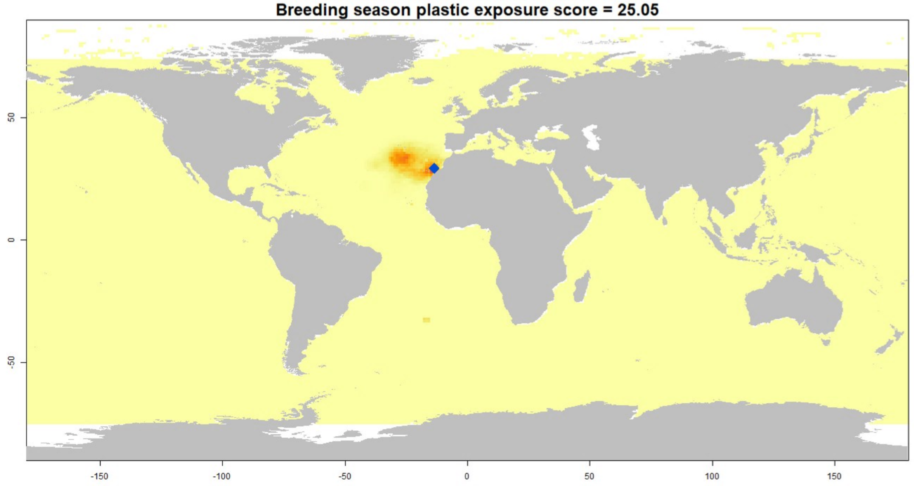

Bulwer's Petrel Bulweria bulwerii, Cape Verde
Breeding season plastic exposure score = 1.83

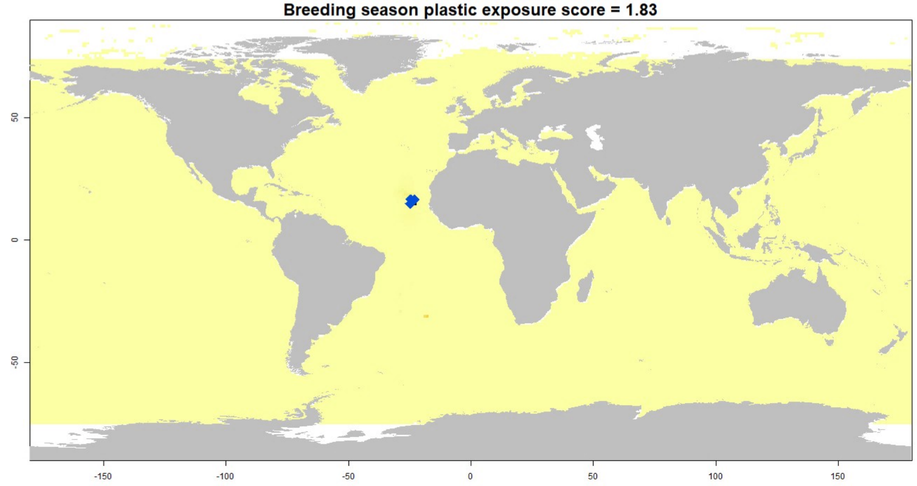

Bulwer's Petrel Bulweria bulwerii, Madeira
Non-breeding season plastic exposure score = 37.2

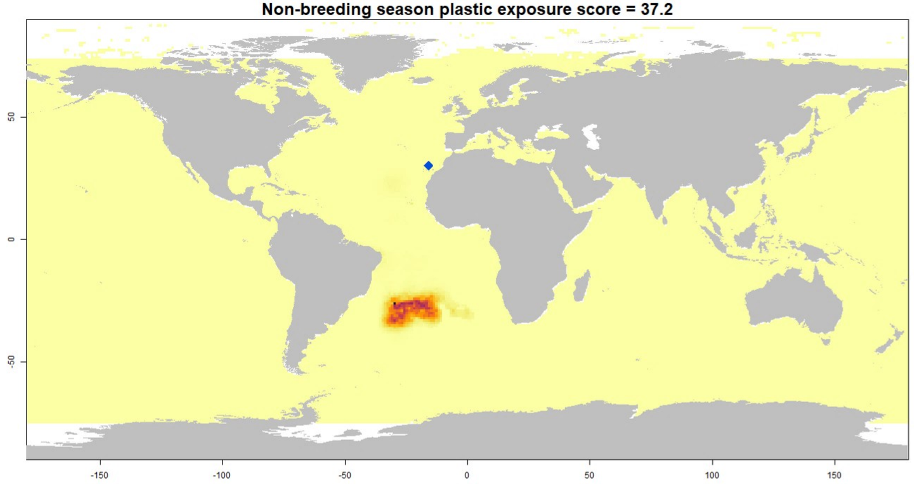

Cory's Shearwater Calonectris borealis, Azores
Non-breeding season plastic exposure score = 54.15

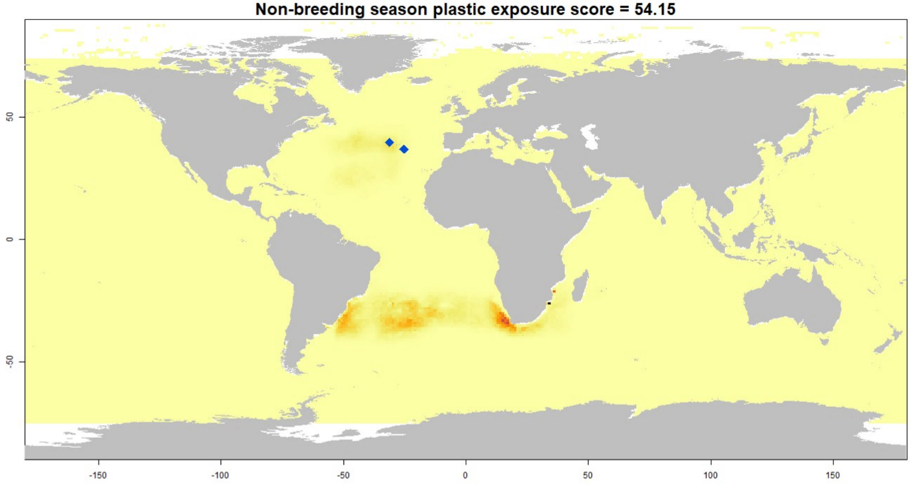

Bulwer's Petrel Bulweria bulwerii, Madeira
Breeding season plastic exposure score = 37.2

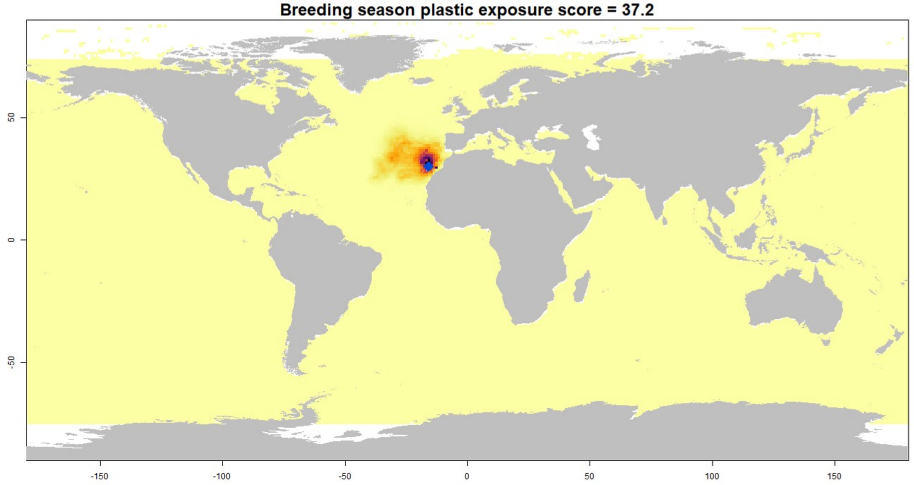

Cory's Shearwater Calonectris borealis, Azores
Breeding season plastic exposure score = 54.15

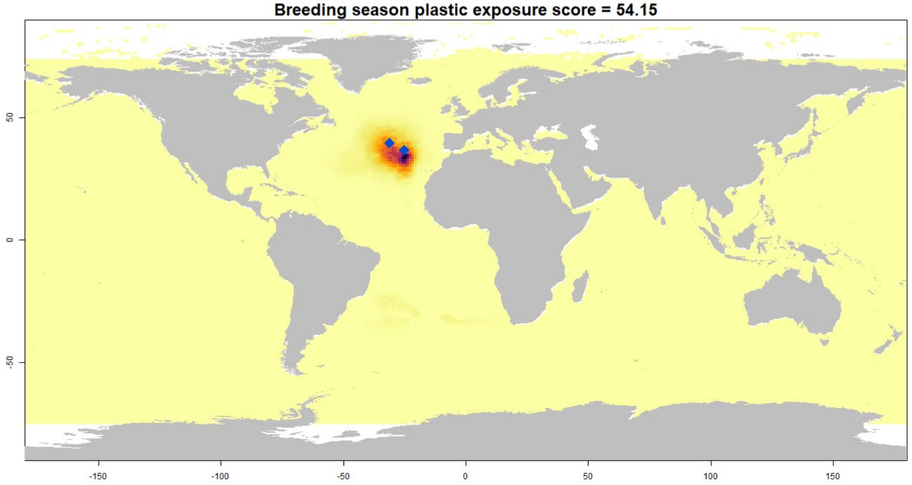

Cory's Shearwater Calonectris borealis, Berlengas
Non-breeding season plastic exposure score = 26.97

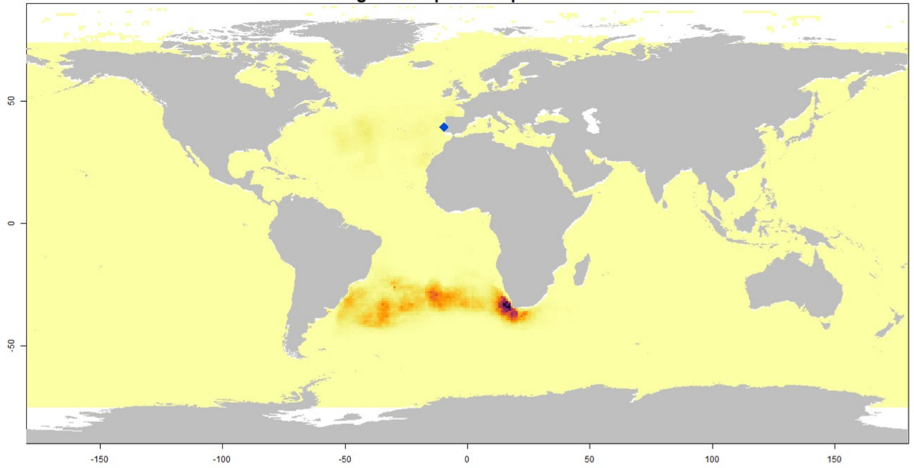

Cory's Shearwater Calonectris borealis, Canarias
Non-breeding season plastic exposure score = 17

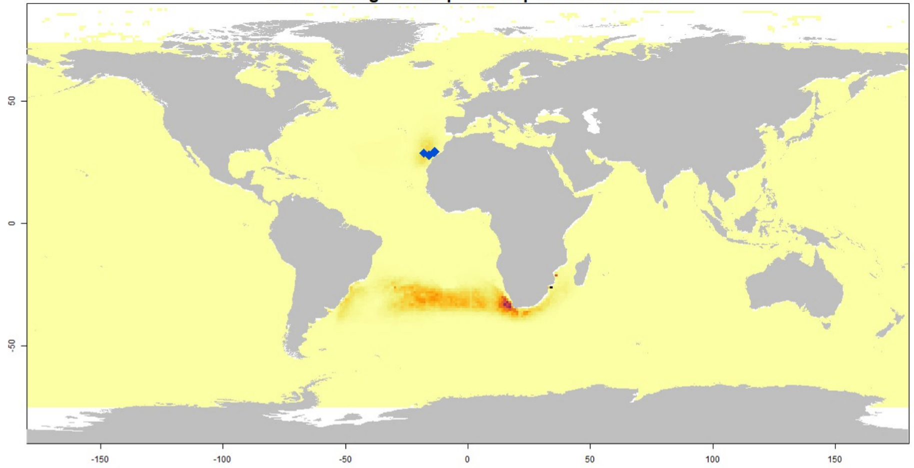

Cory's Shearwater Calonectris borealis, Berlengas
Breeding season plastic exposure score = 26.97

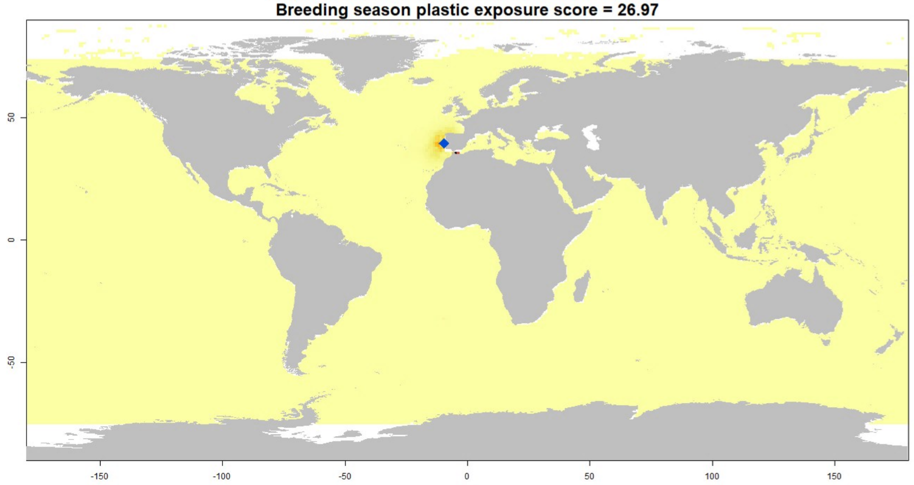

Cory's Shearwater Calonectris borealis, Canarias
Breeding season plastic exposure score = 17

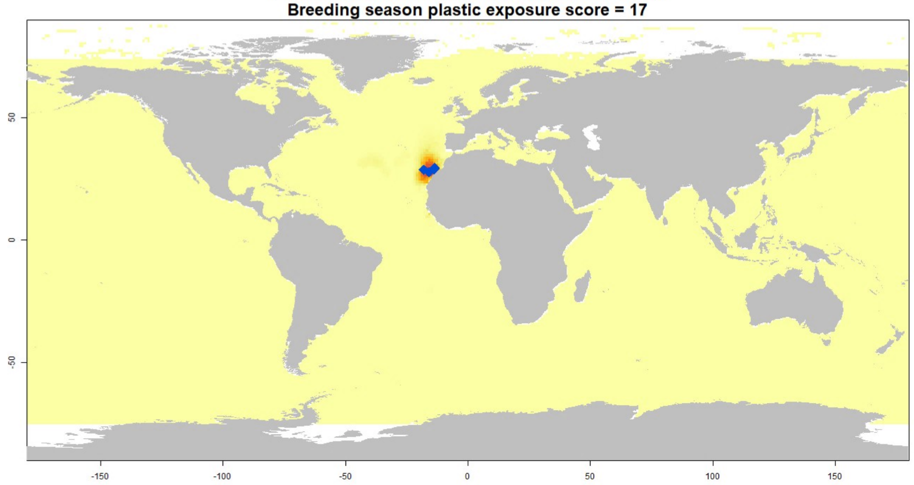

Cory's Shearwater Calonectris borealis, Madeira
Non-breeding season plastic exposure score = 27.75

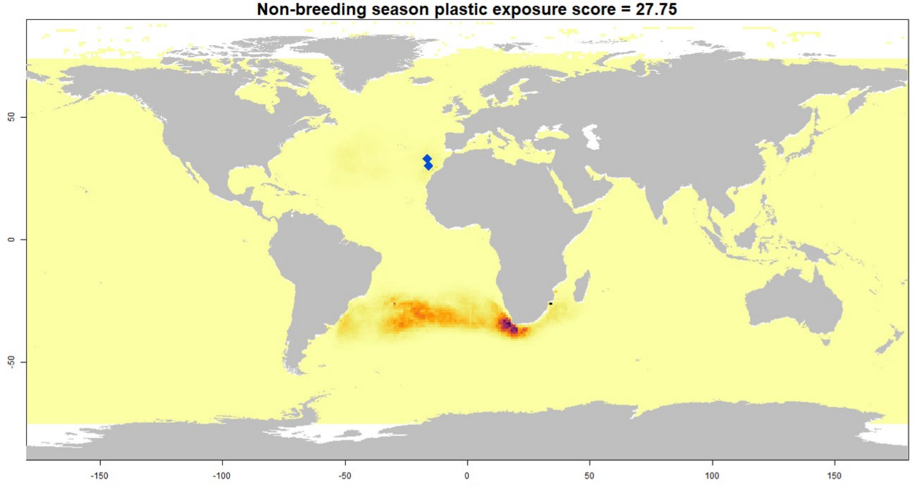

Scopoli's Shearwater Calonectris diomedea, Balearic Archipelago
Non-breeding season plastic exposure score = 3103.83

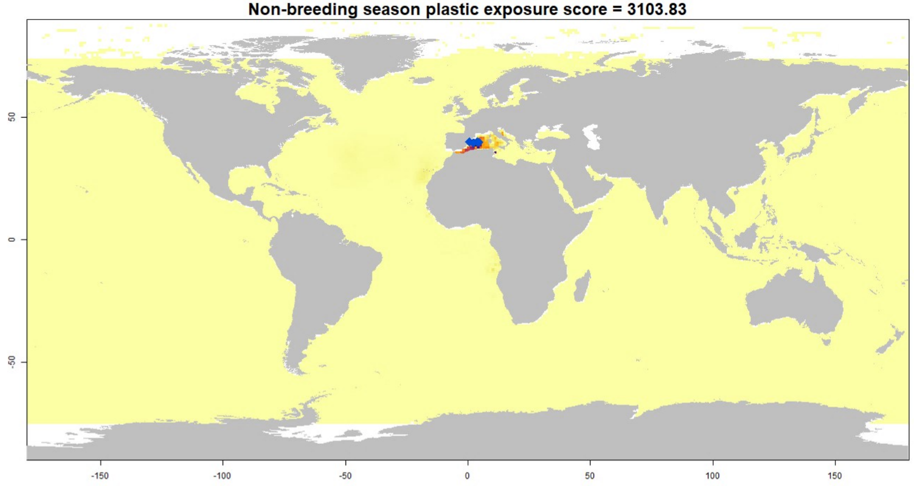

Cory's Shearwater Calonectris borealis, Madeira
Breeding season plastic exposure score = 27.75

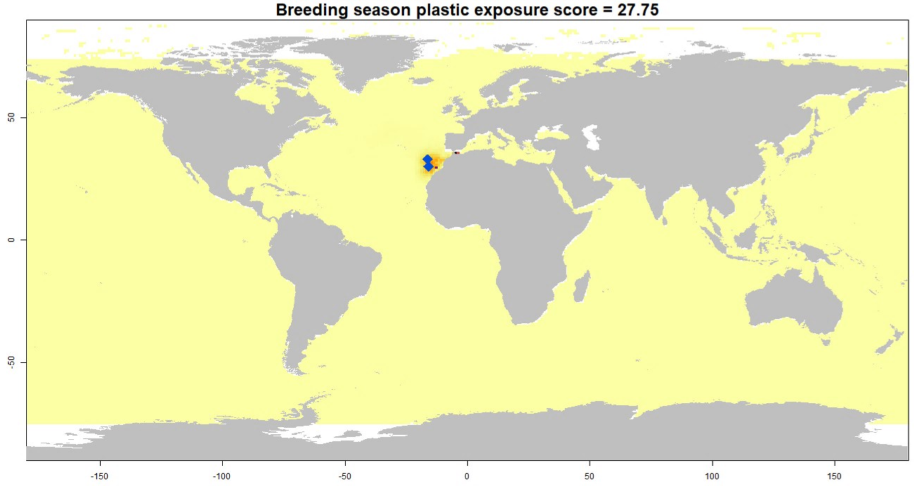

Scopoli's Shearwater Calonectris diomedea, Balearic Archipelago
Breeding season plastic exposure score = 3103.83

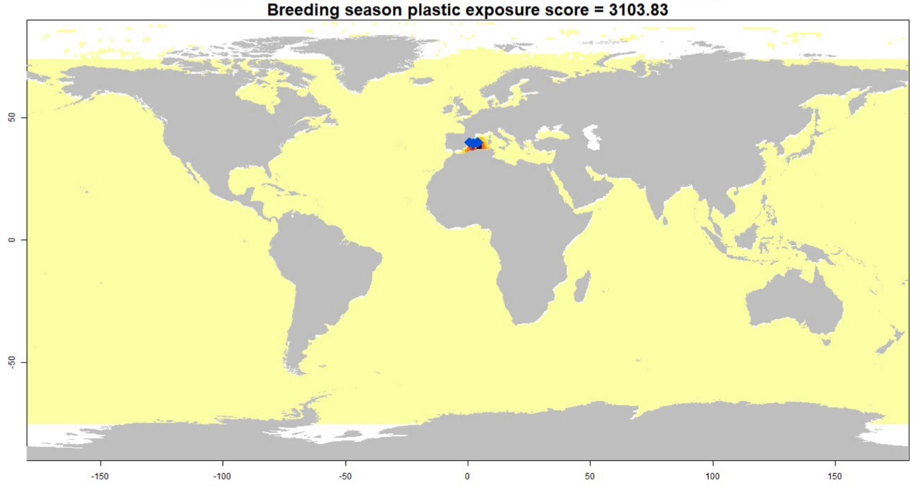

Scopoli's Shearwater Calonectris diomedea, Chafarinas
Non-breeding season plastic exposure score = 2612.55

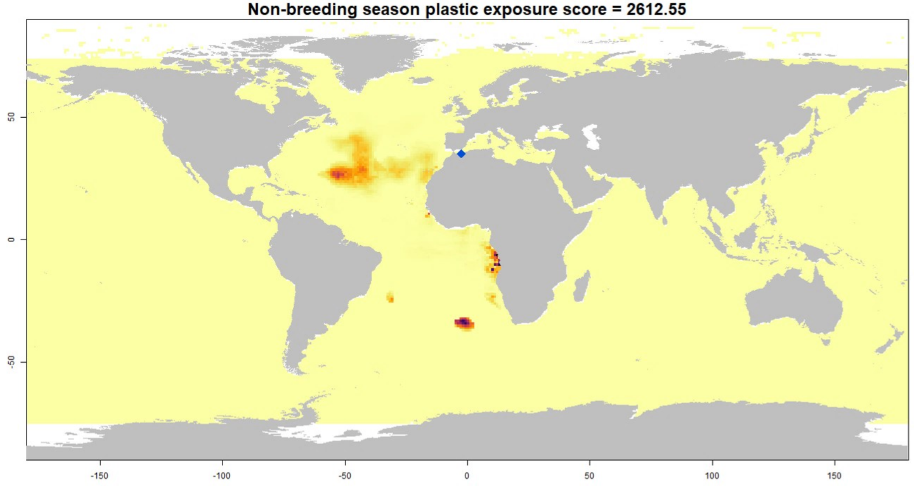

Scopoli's Shearwater Calonectris diomedea, La Maddalena Archipelago
Non-breeding season plastic exposure score = 2487.84

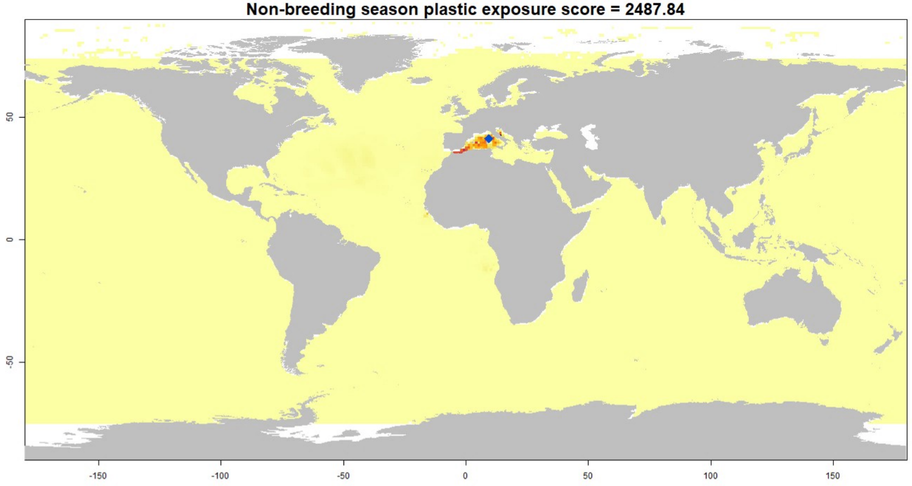

Scopoli's Shearwater Calonectris diomedea, Chafarinas
Breeding season plastic exposure score = 2612.55

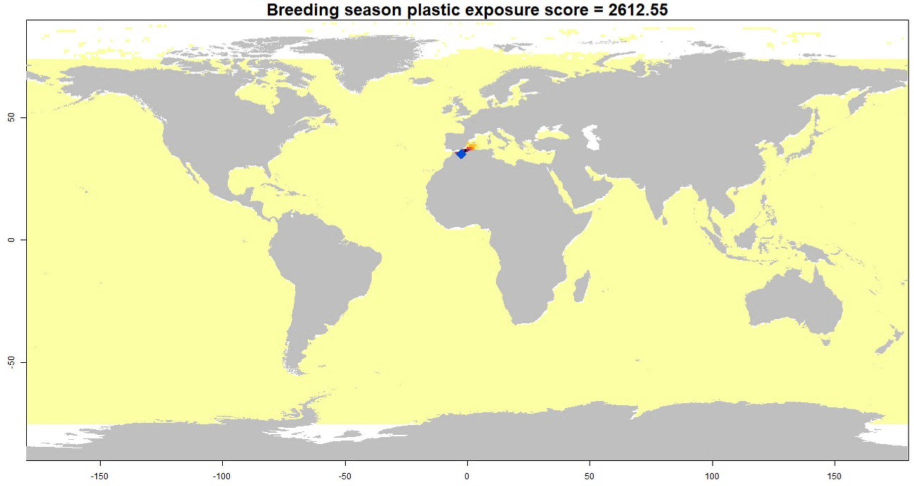

Scopoli's Shearwater Calonectris diomedea, La Maddalena Archipelago
Breeding season plastic exposure score = 2487.84

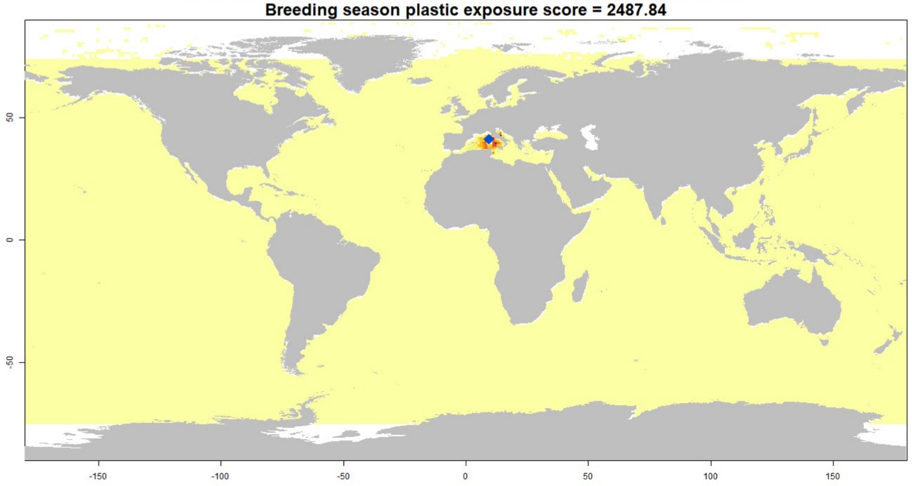

Scopoli's Shearwater Calonectris diomedea, Malta
Breeding season plastic exposure score = 3473.88

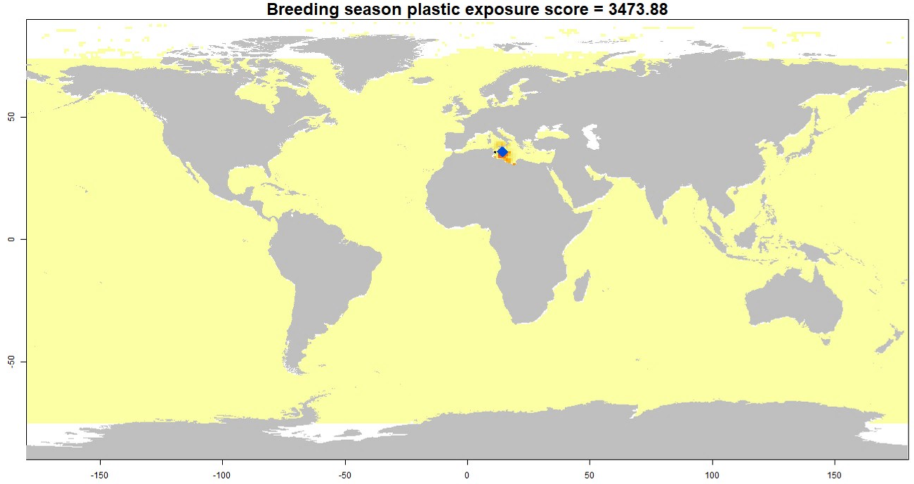

Scopoli's Shearwater Calonectris diomedea, Tremiti Archipelago
Breeding season plastic exposure score = 785.15

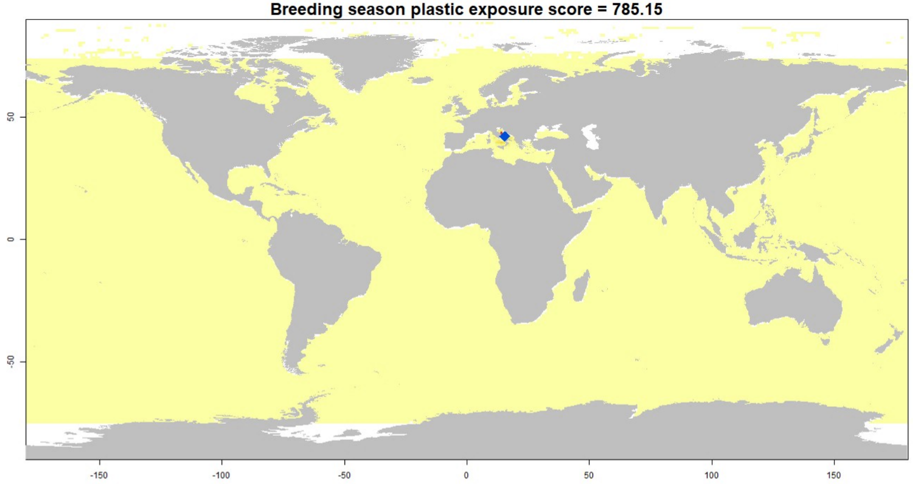

Scopoli's Shearwater Calonectris diomedea, Linosa
Breeding season plastic exposure score = 2263.75

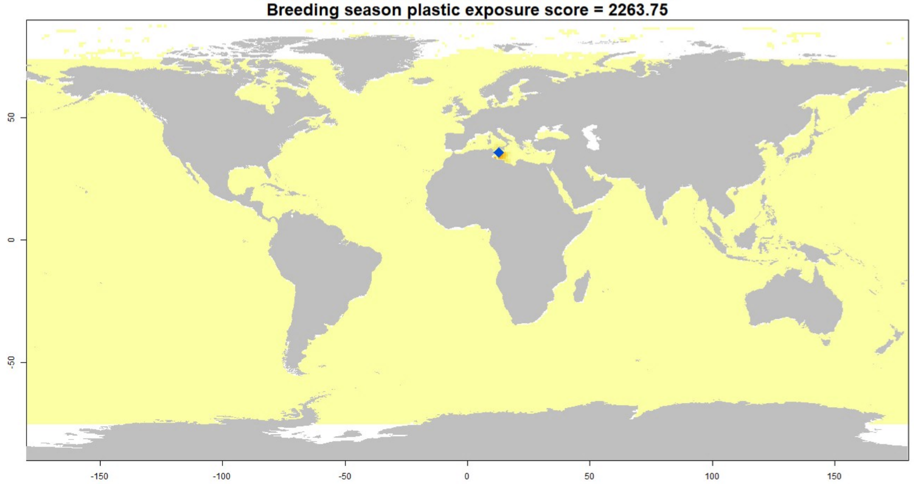

Scopoli's Shearwater Calonectris diomedea, Malta
Non-breeding season plastic exposure score = 3473.88

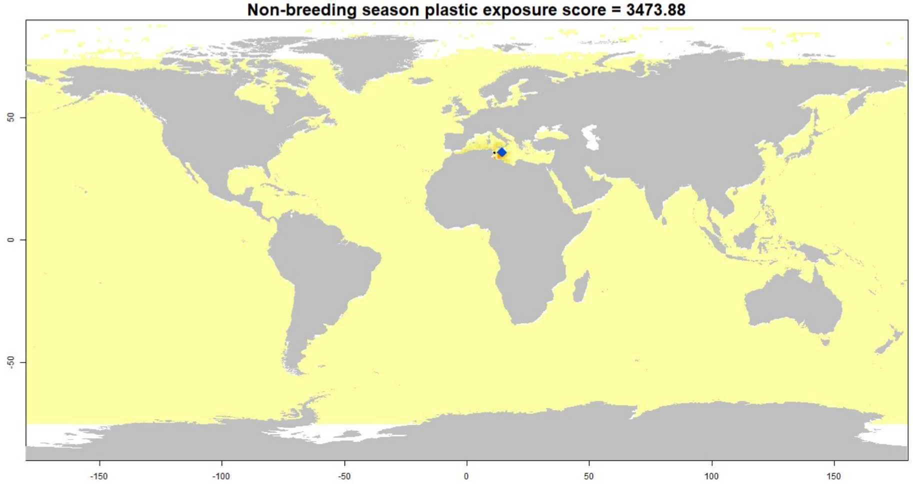

Scopoli's Shearwater Calonectris diomedea, Zembra
Breeding season plastic exposure score = 3834.54

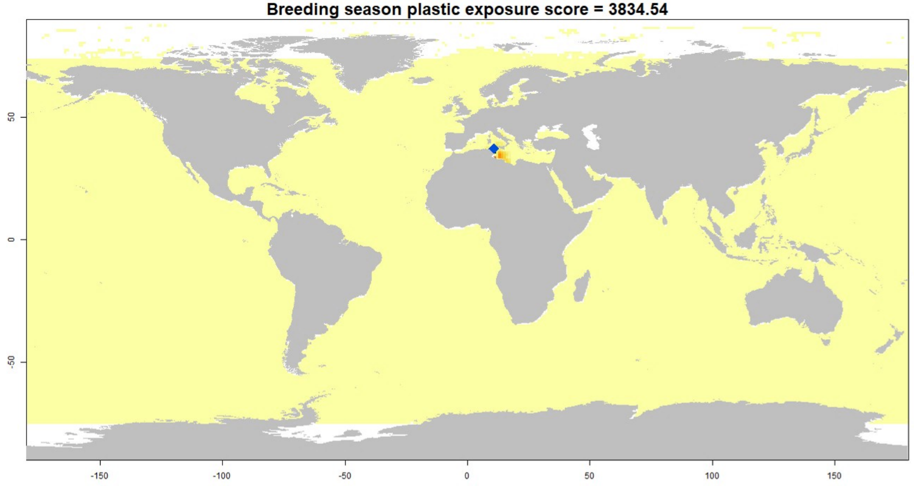

Cape Verde Shearwater Calonectris edwardsii, Cape Verde
Breeding season plastic exposure score = 4.06

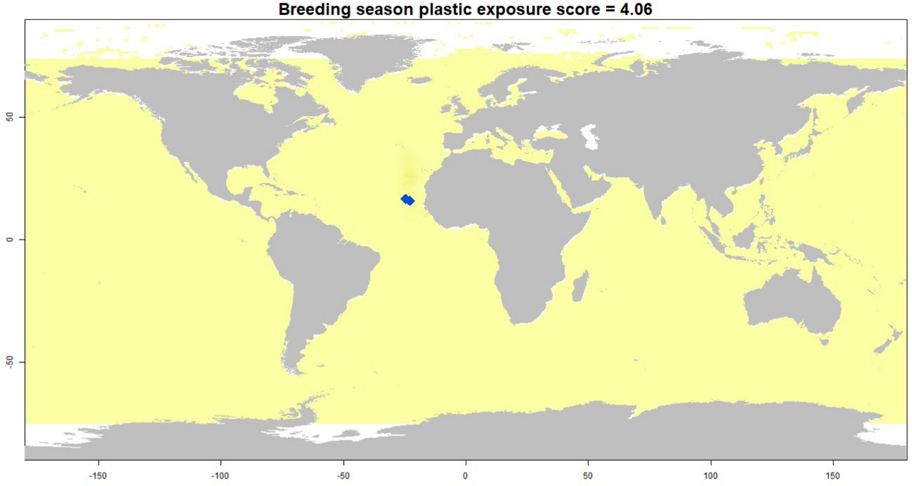

Scopoli's Shearwater Calonectris diomedea, Tuscany Archipelago
Breeding season plastic exposure score = 486.37

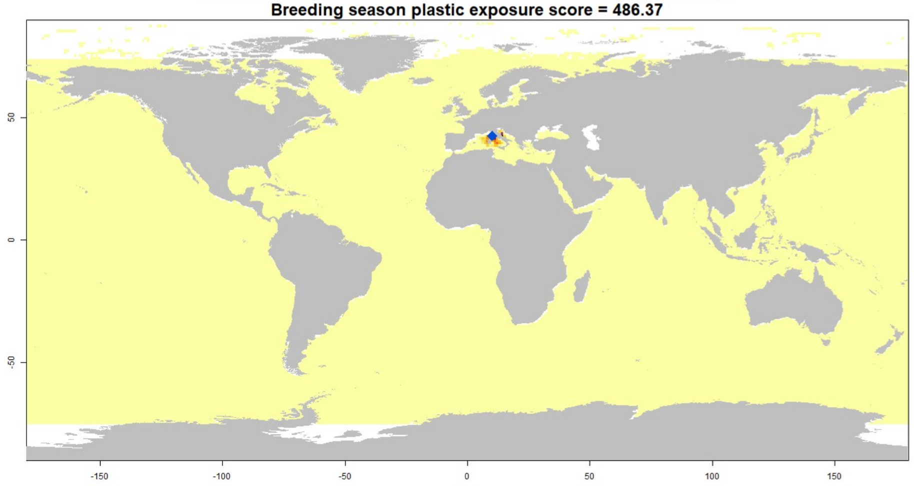

Scopoli's Shearwater Calonectris diomedea, Zembra
Non-breeding season plastic exposure score = 3834.54

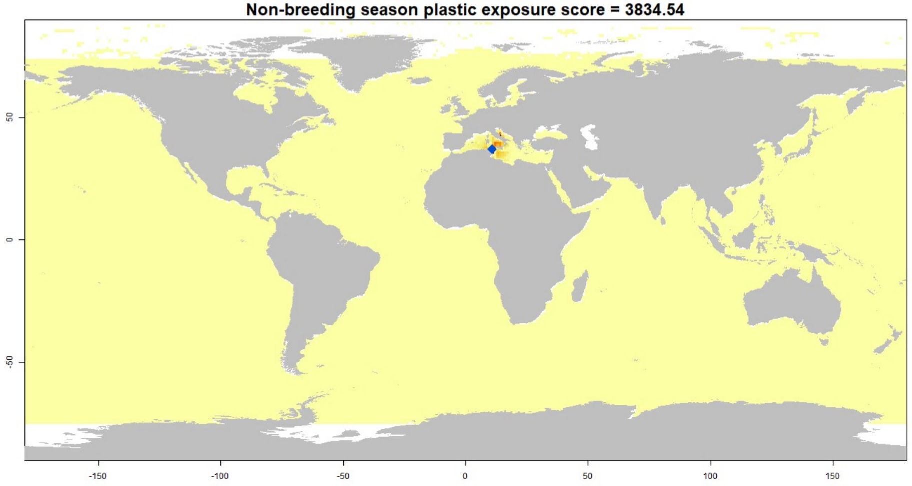

Streaked Shearwater Calonectris leucomelas, Iwate
Breeding season plastic exposure score = 312.3

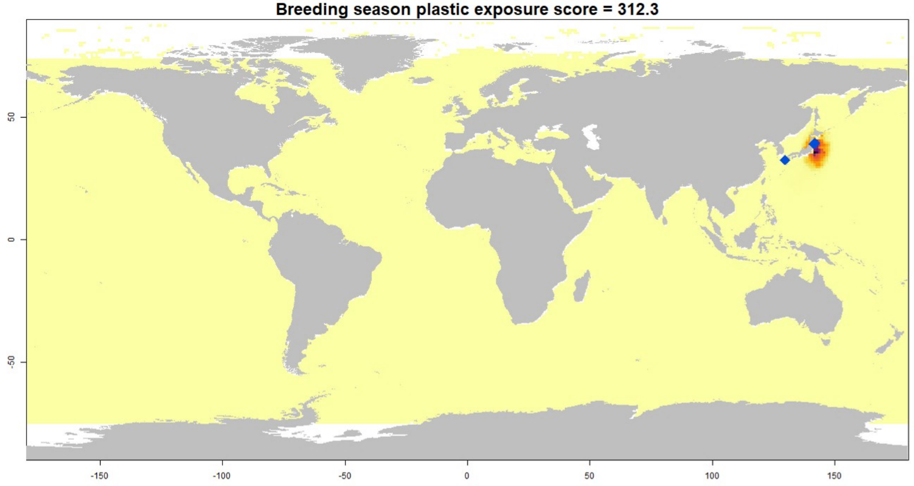

Streaked Shearwater Calonectris leucomelas, Korea
Breeding season plastic exposure score = 281.33

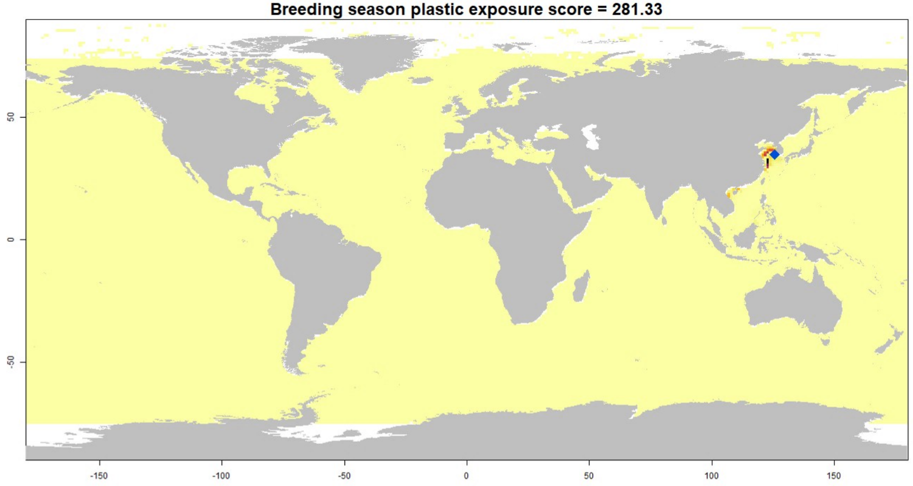

Cape Verde Shearwater Calonectris edwardsii, Cape Verde
Non-breeding season plastic exposure score = 4.06

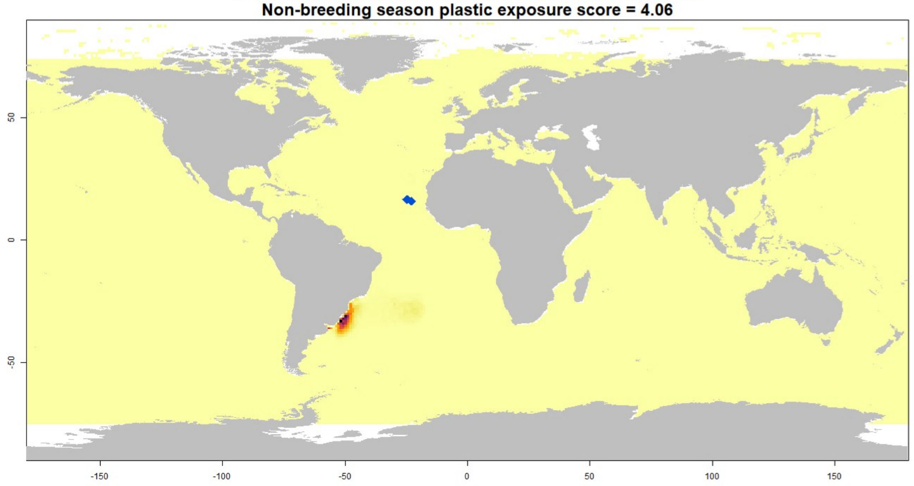

Streaked Shearwater Calonectris leucomelas, Iwate
Non-breeding season plastic exposure score = 312.3

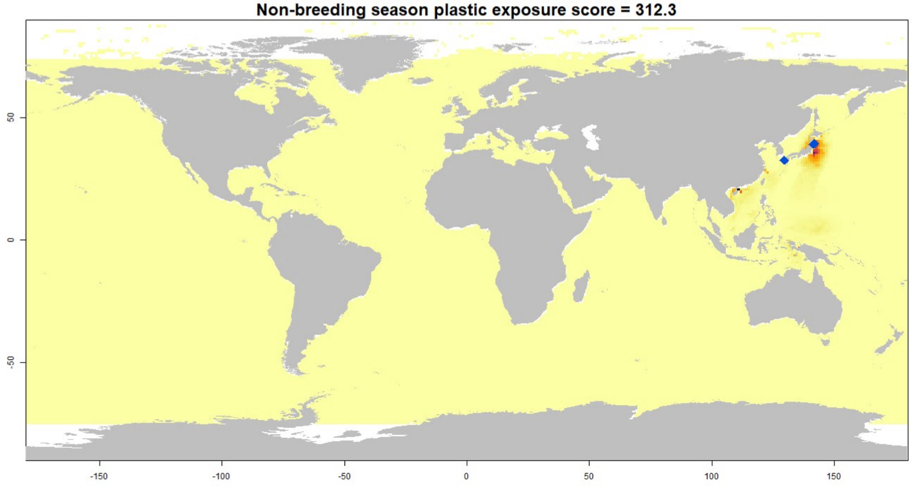

Streaked Shearwater Calonectris leucomelas, Mikura
Breeding season plastic exposure score = 348.16

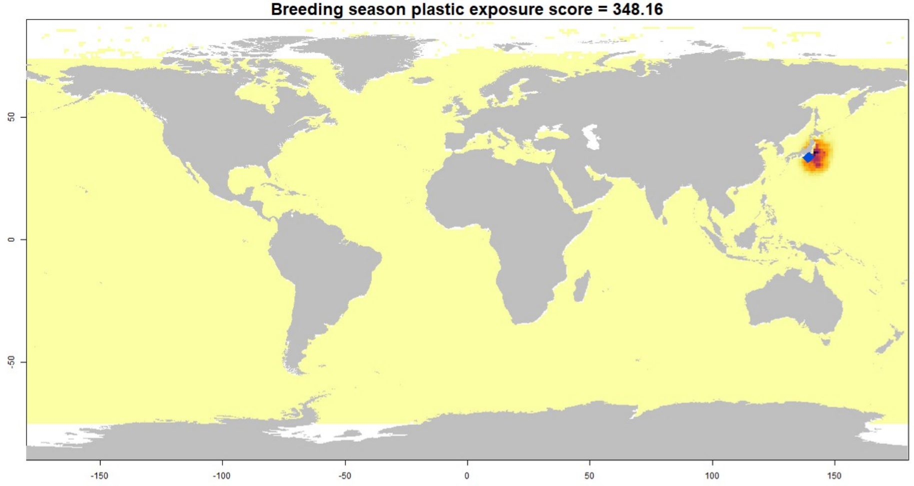

Streaked Shearwater Calonectris leucomelas, Niigata
Breeding season plastic exposure score = 89.64

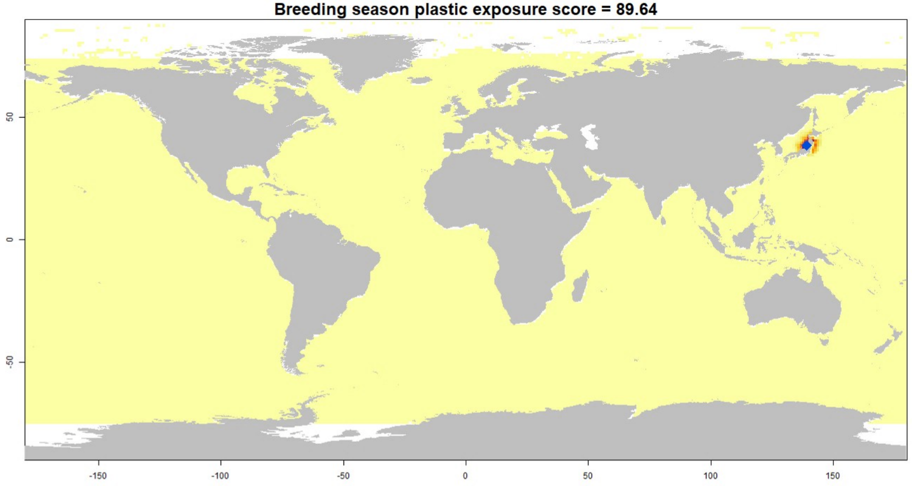

Streaked Shearwater Calonectris leucomelas, Korea
Non-breeding season plastic exposure score = 281.33

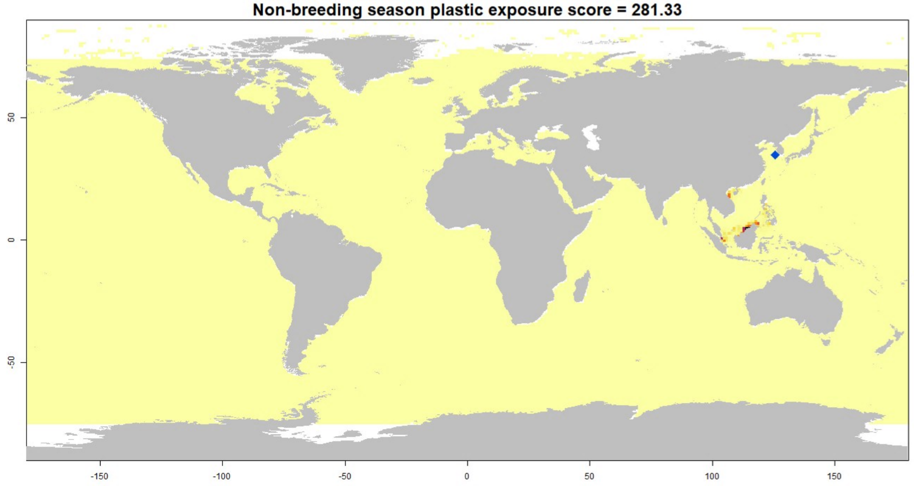

Streaked Shearwater Calonectris leucomelas, Mikura
Non-breeding season plastic exposure score = 348.16

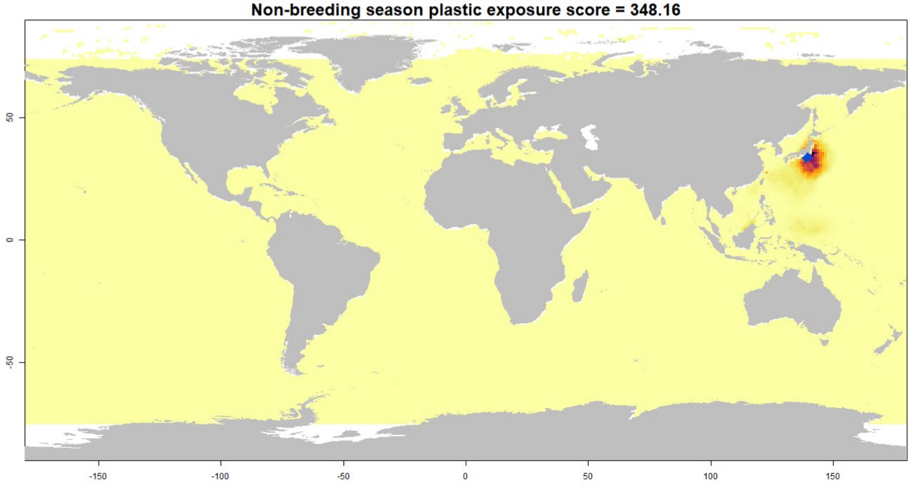

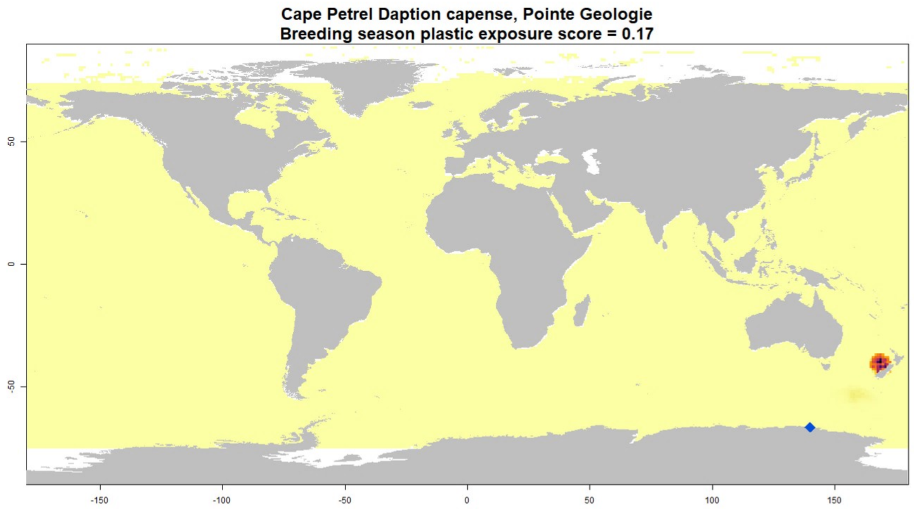
Cape Petrel Daption capense, Pointe Geologie
Breeding season plastic exposure score = 0.17
50
0
-50
-150
-100
-50
0
50
100
150

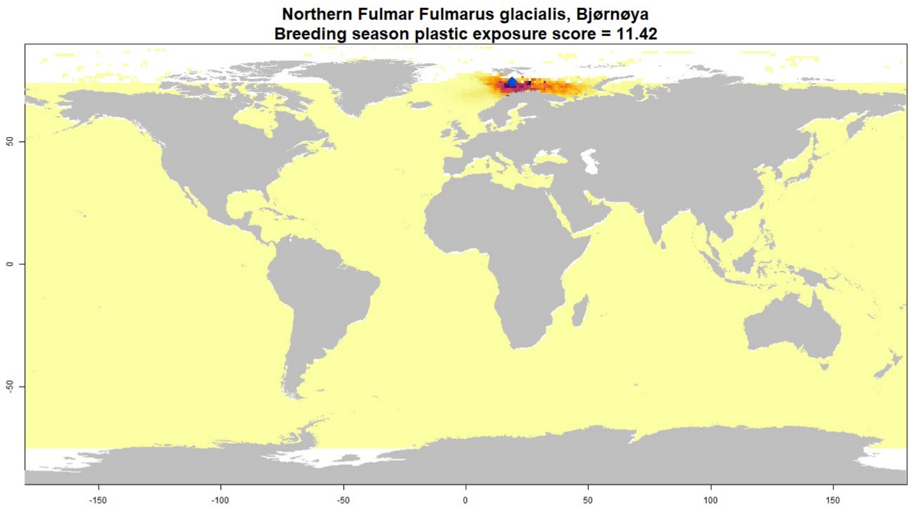
Northern Fulmar Fulmarus glacialis, Bjørnøya
Breeding season plastic exposure score = 11.42
50
0
-50
-150
-100
-50
0
50
100
150

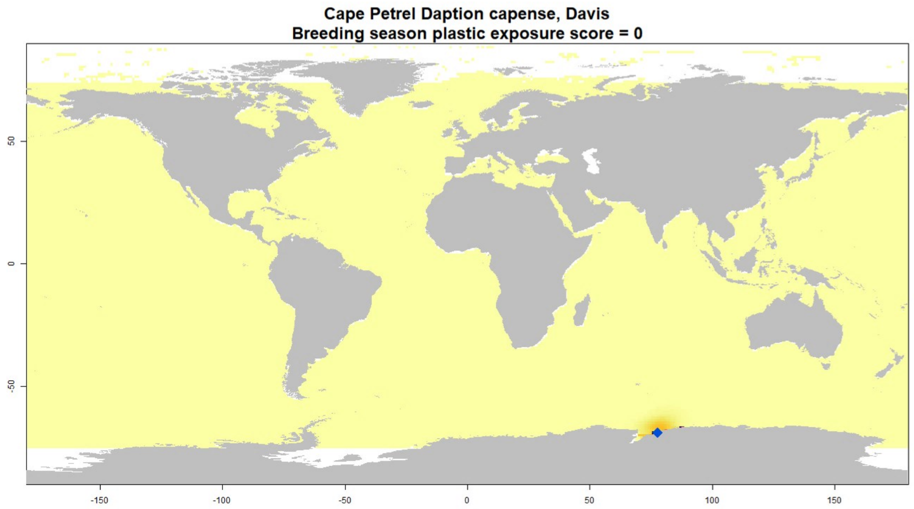
Cape Petrel Daption capense, Davis
Breeding season plastic exposure score = 0
50
0
-50
-150
-100
-50
0
50
100
150

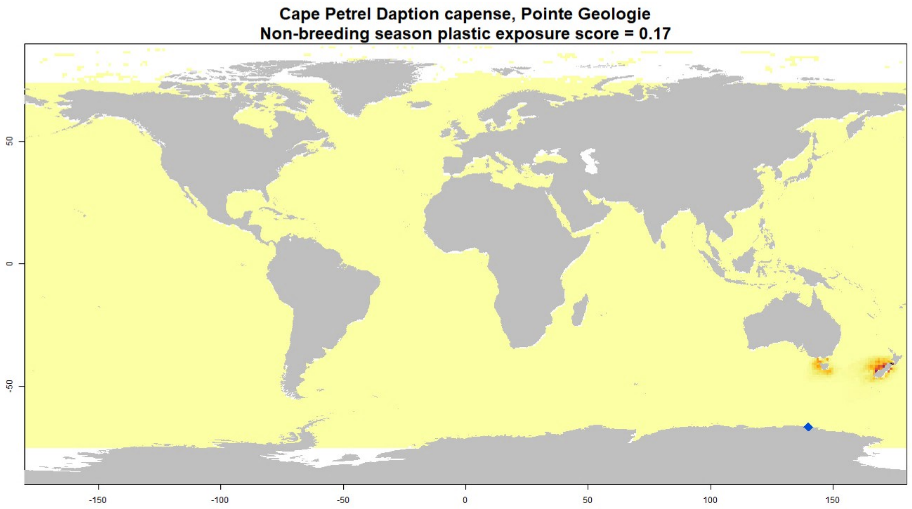
Cape Petrel Daption capense, Pointe Geologie
Non-breeding season plastic exposure score = 0.17
50
0
-50
-150
-100
-50
0
50
100
150

Northern Fulmar Fulmarus glacialis, Breidafjordur
Breeding season plastic exposure score = 1.93

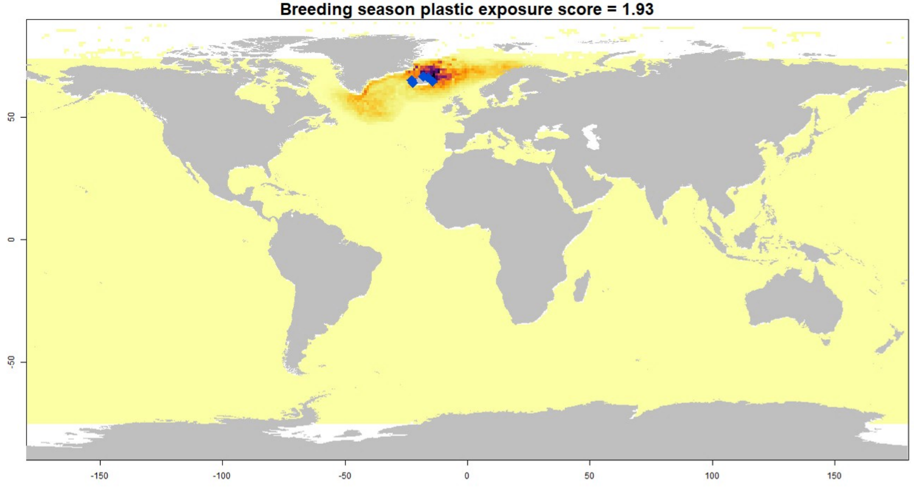

Northern Fulmar Fulmarus glacialis, Cape Vera
Breeding season plastic exposure score = 0.13

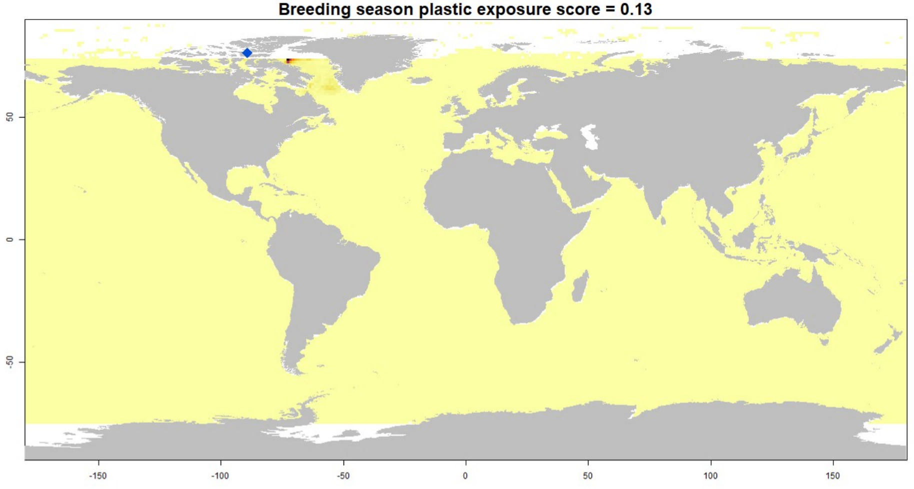

Northern Fulmar Fulmarus glacialis, Bjørnøya
Non-breeding season plastic exposure score = 11.42

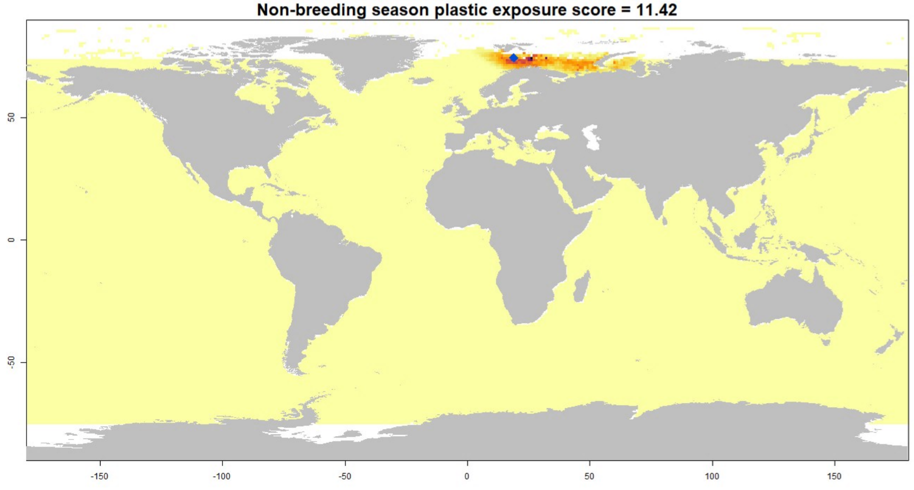

Northern Fulmar Fulmarus glacialis, Breidafjordur
Non-breeding season plastic exposure score = 1.93

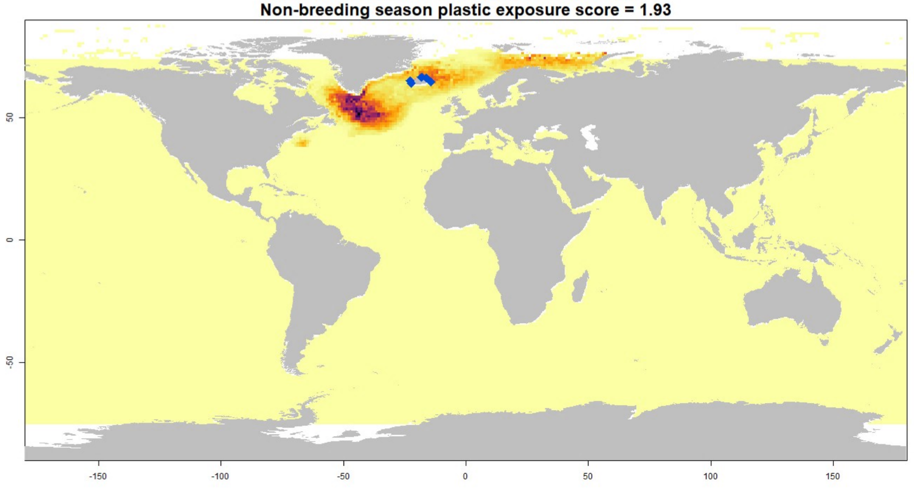

Northern Fulmar Fulmarus glacialis, Faroe Islands
Breeding season plastic exposure score = 5.78

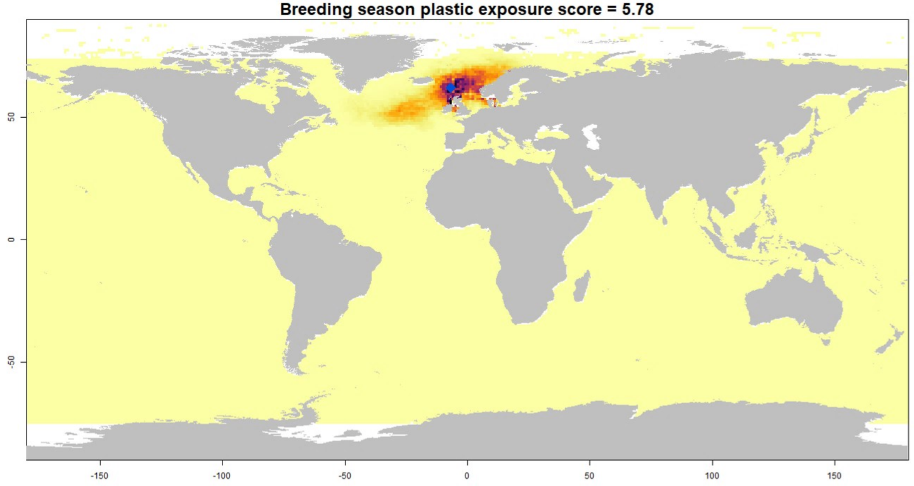

Northern Fulmar Fulmarus glacialis, Jan Mayen
Breeding season plastic exposure score = 4.31

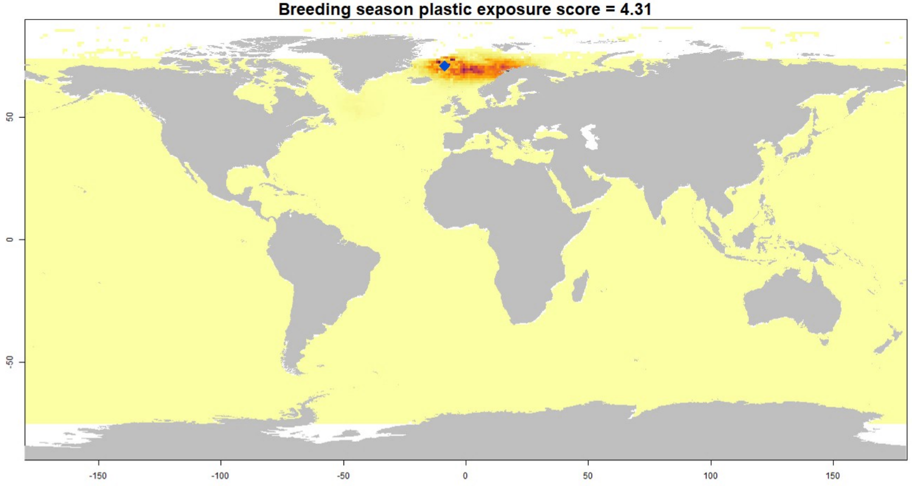

Northern Fulmar Fulmarus glacialis, Cape Vera
Non-breeding season plastic exposure score = 0.13

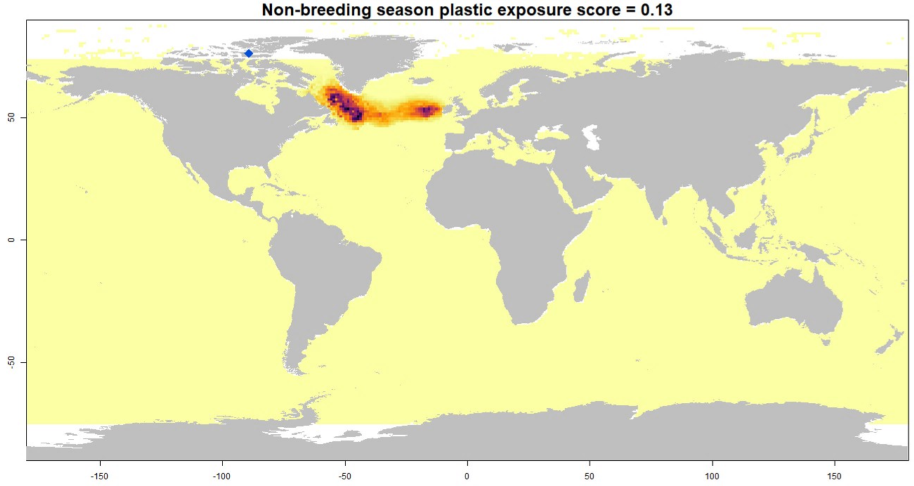

Northern Fulmar Fulmarus glacialis, Faroe Islands
Non-breeding season plastic exposure score = 5.78

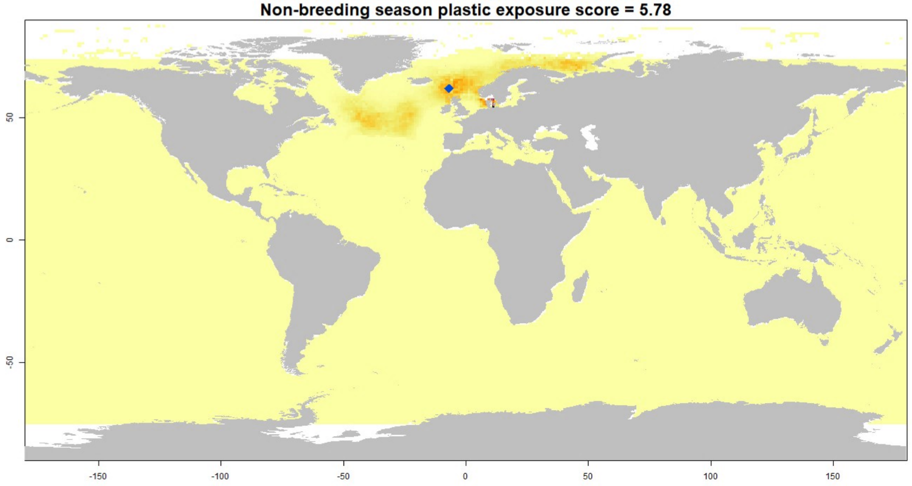

Northern Fulmar Fulmarus glacialis, Norway
Breeding season plastic exposure score = 15.17

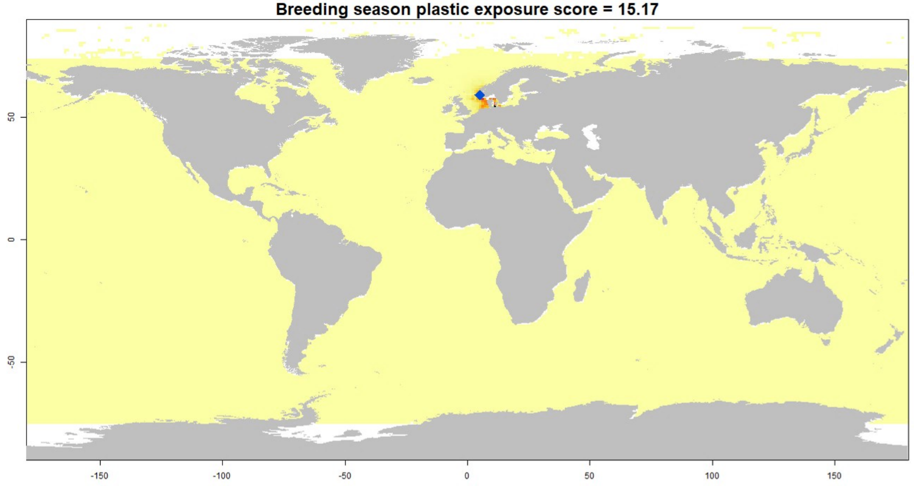

Northern Fulmar Fulmarus glacialis, Scotland
Breeding season plastic exposure score = 11.34

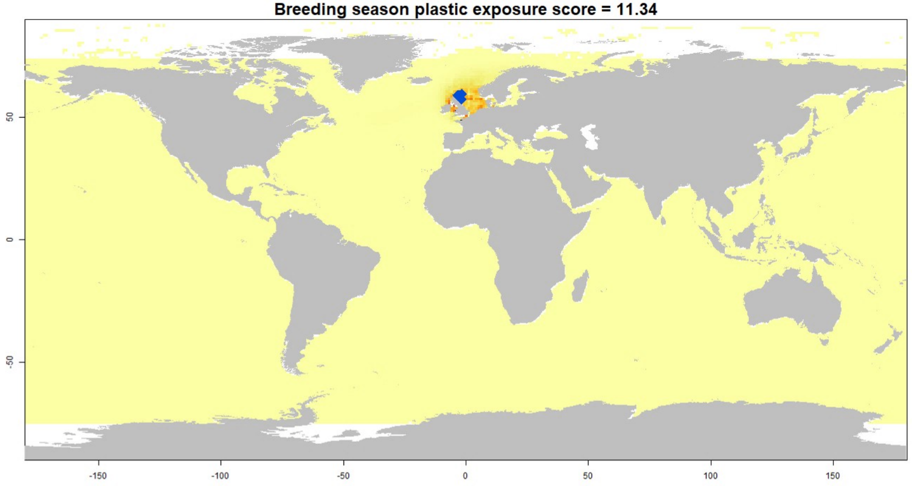

Northern Fulmar Fulmarus glacialis, Jan Mayen
Non-breeding season plastic exposure score = 4.31

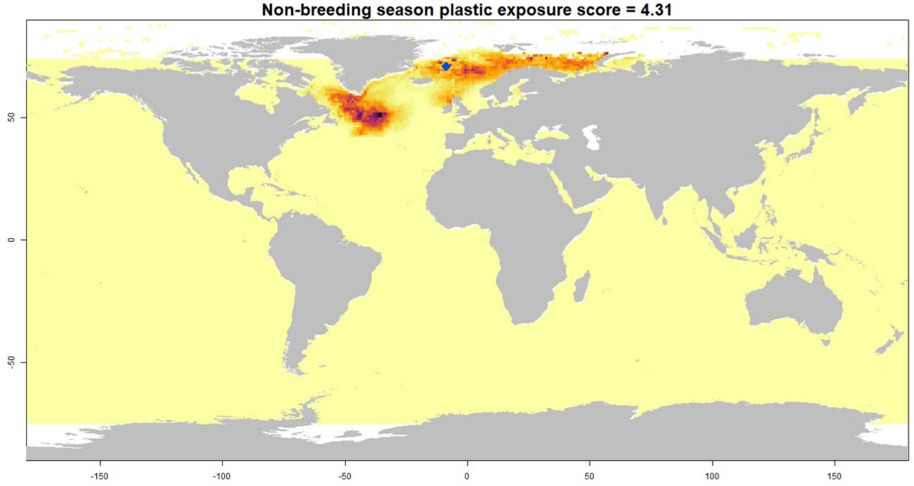

Northern Fulmar Fulmarus glacialis, Norway
Non-breeding season plastic exposure score = 15.17

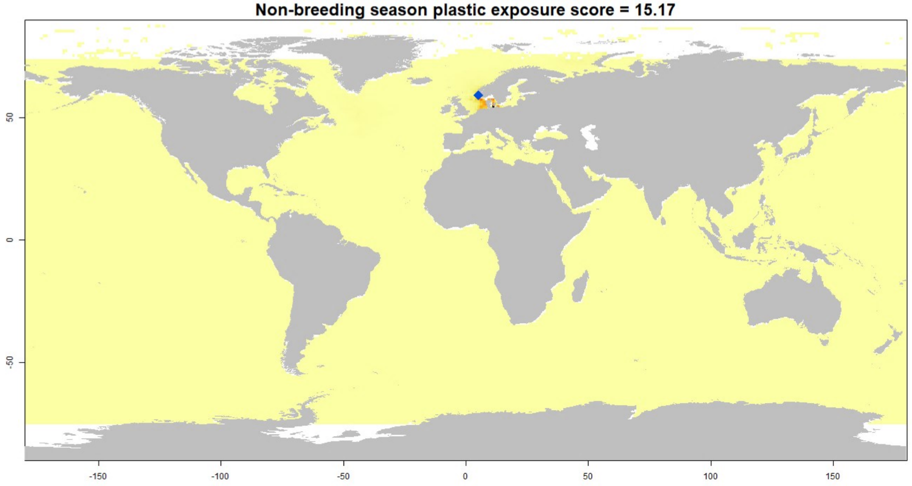

Northern Fulmar Fulmarus glacialis, Svalbard
Breeding season plastic exposure score = 4.99

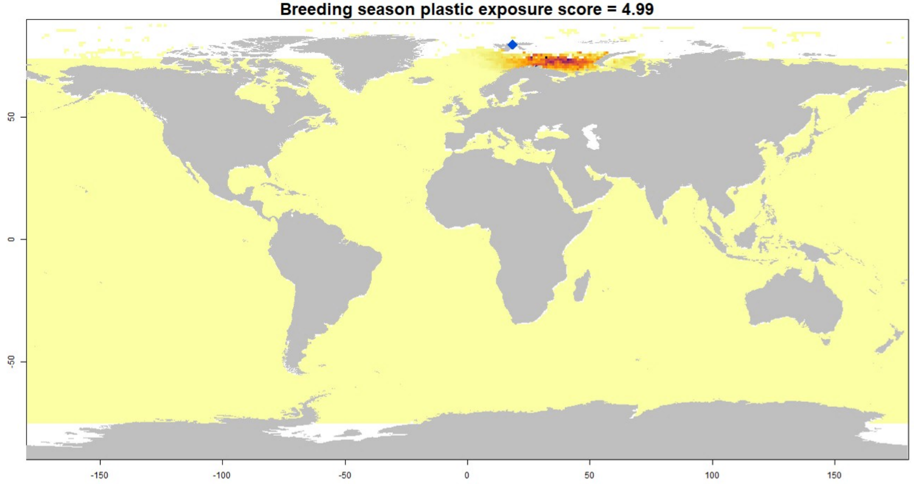

Southern Fulmar Fulmarus glacialoides, Davis
Breeding season plastic exposure score = 0

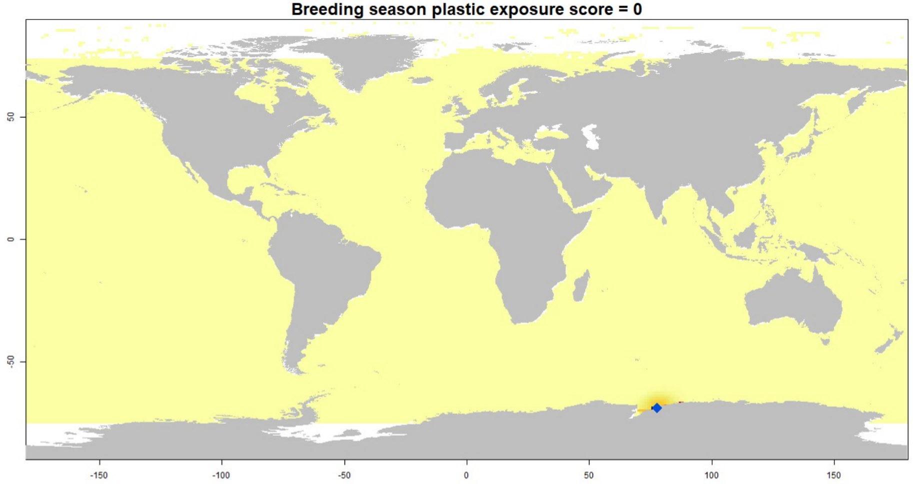

Northern Fulmar Fulmarus glacialis, Scotland
Non-breeding season plastic exposure score = 11.34

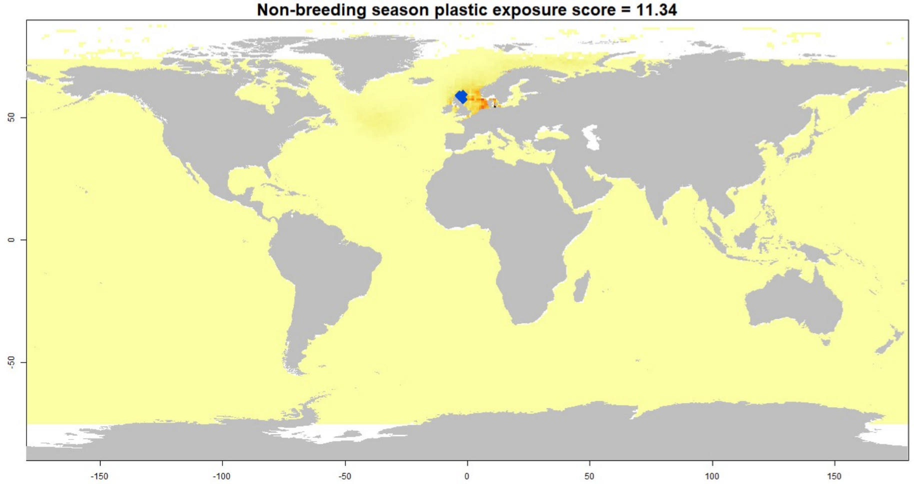

Northern Fulmar Fulmarus glacialis, Svalbard
Non-breeding season plastic exposure score = 4.99

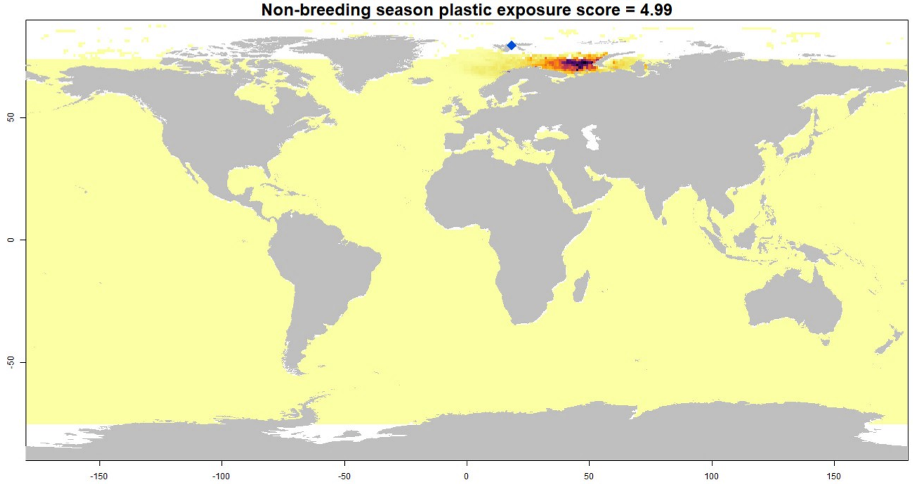

Southern Fulmar Fulmarus glacialoides, Pointe Geologie
Non-breeding season plastic exposure score = 0

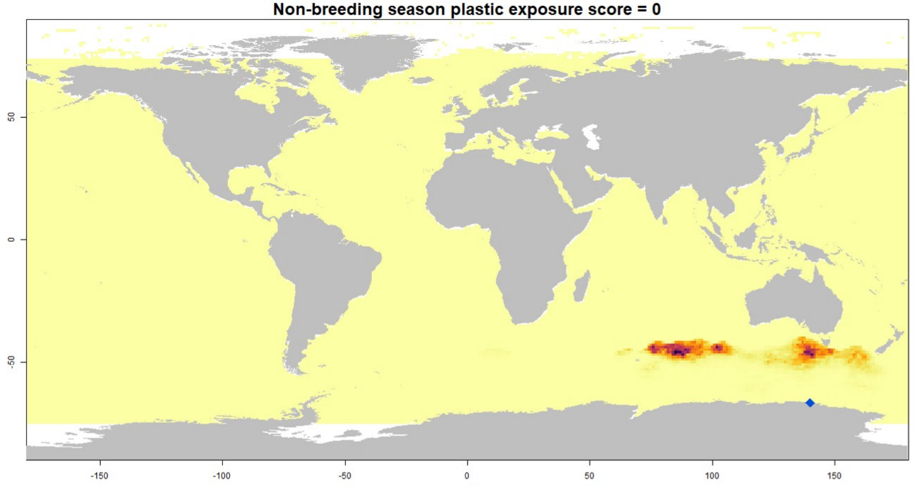

Blue Petrel Halobaena caerulea, Kerguelen
Non-breeding season plastic exposure score = 2.69

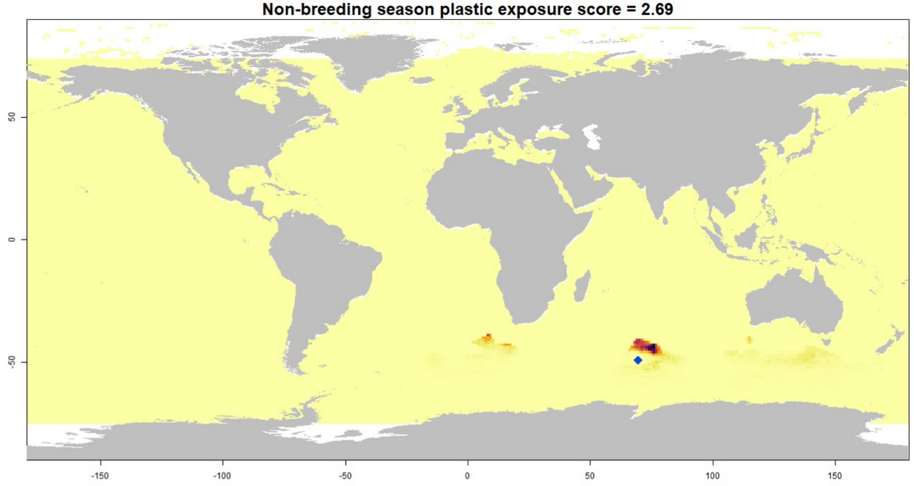

Southern Fulmar Fulmarus glacialoides, Pointe Geologie
Breeding season plastic exposure score = 0

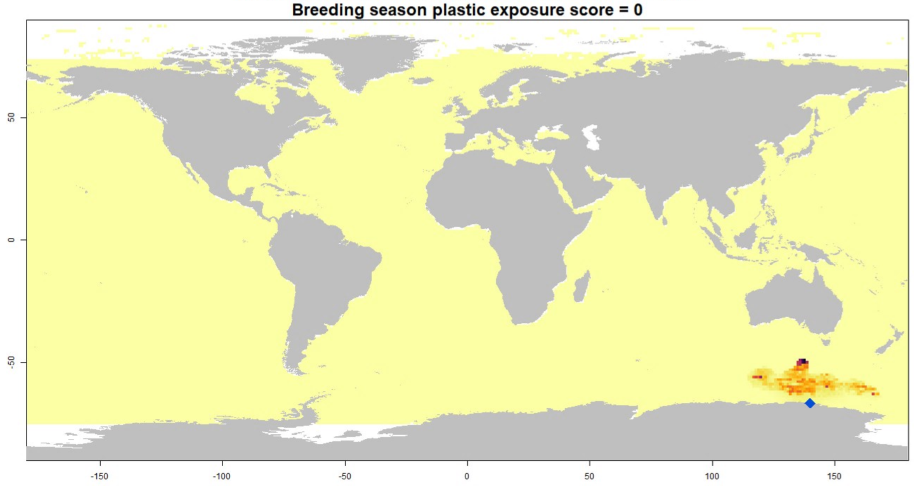

Blue Petrel Halobaena caerulea, Kerguelen
Breeding season plastic exposure score = 2.69

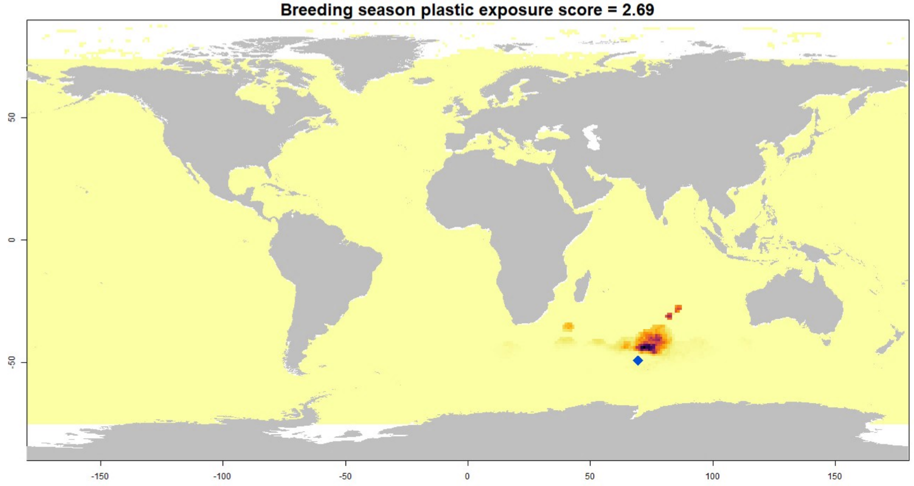

Blue Petrel Halobaena caerulea, South Georgia
Non-breeding season plastic exposure score = 0.36

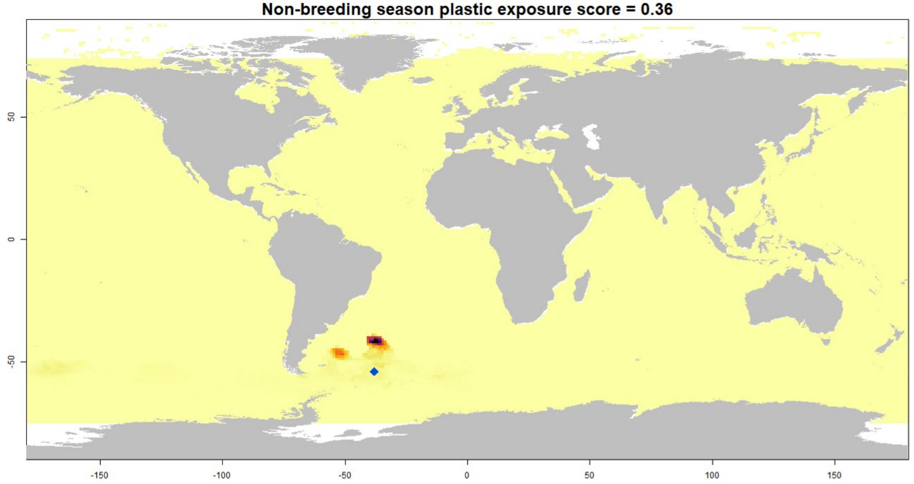

Band-rumped Storm-petrel Hydrobates castro, Berlengas
Breeding season plastic exposure score = 31

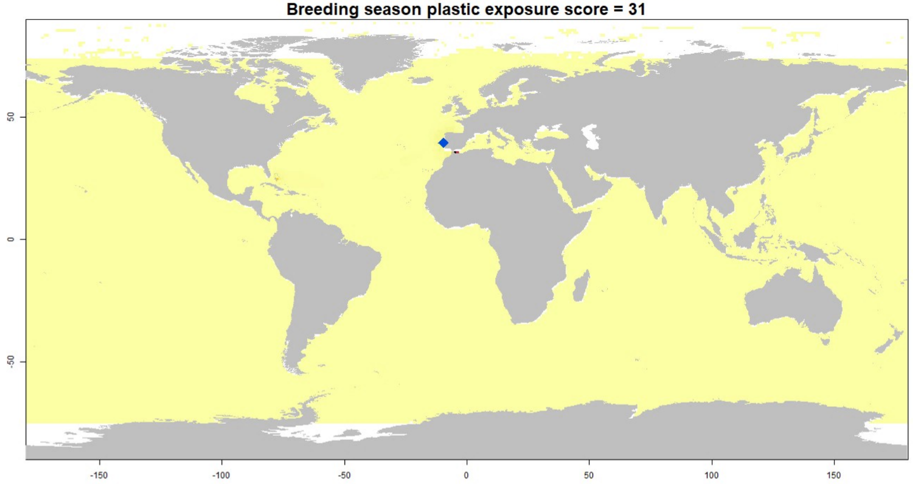

Blue Petrel Halobaena caerulea, South Georgia
Breeding season plastic exposure score = 0.36

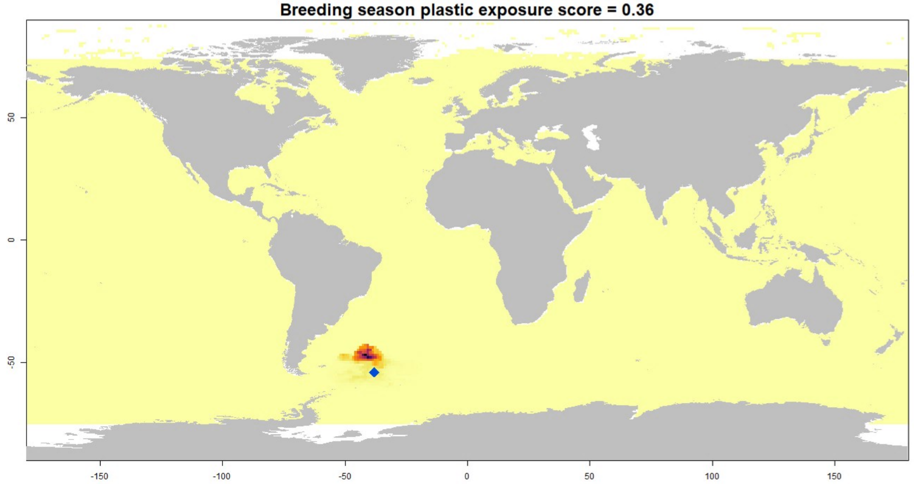

Band-rumped Storm-petrel Hydrobates castro, Azores
Breeding season plastic exposure score = 18.83

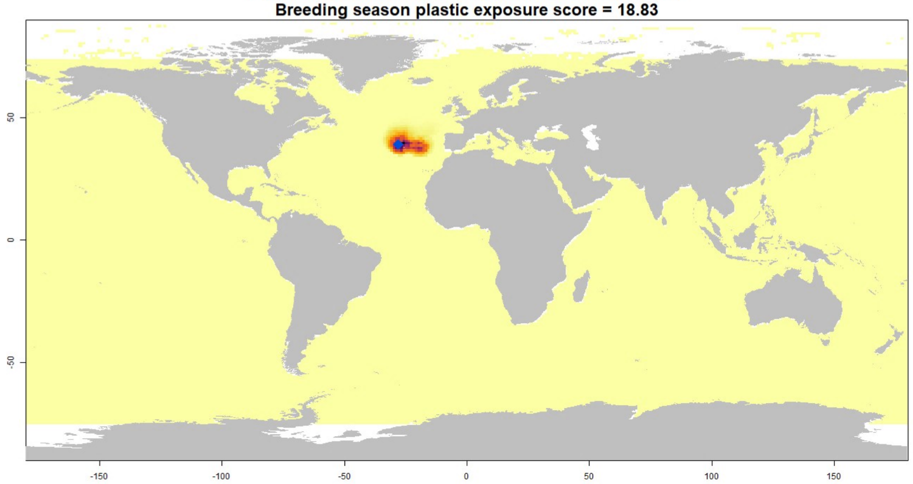

Band-rumped Storm-petrel Hydrobates castro, St Helena
Breeding season plastic exposure score = 28.94

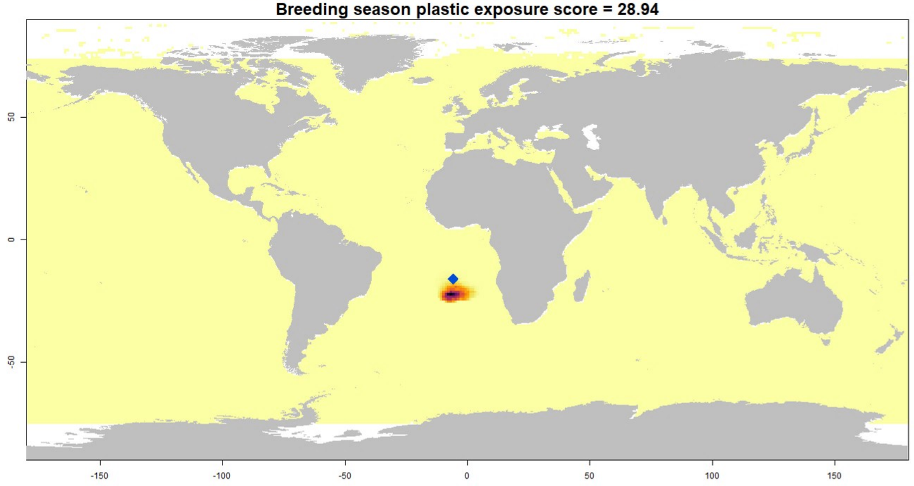

Fork-tailed Storm-petrel Hydrobates furcatus, Gillam Island
Non-breeding season plastic exposure score = 38.41

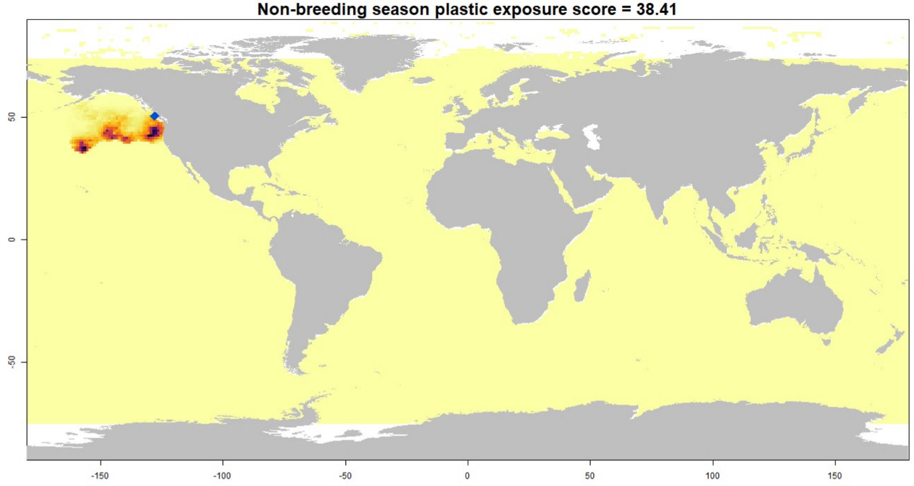

Band-rumped Storm-petrel Hydrobates castro, Berlengas
Non-breeding season plastic exposure score = 31

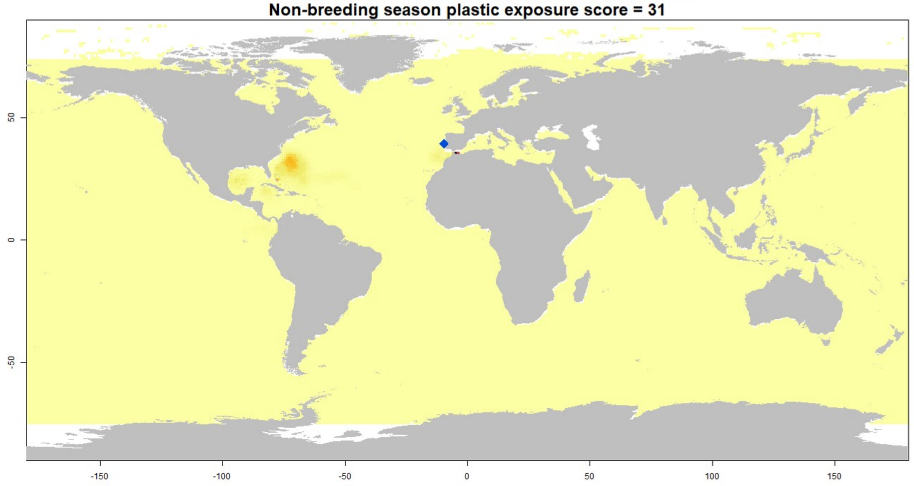

Fork-tailed Storm-petrel Hydrobates furcatus, Gillam Island
Breeding season plastic exposure score = 38.41

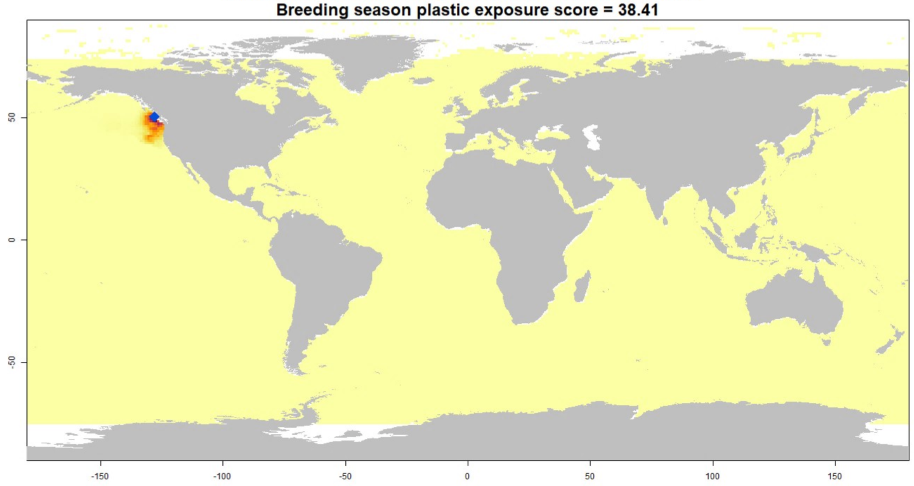

Leach's Storm-petrel Hydrobates leucorhous, Gillam Island
Breeding season plastic exposure score = 49.84

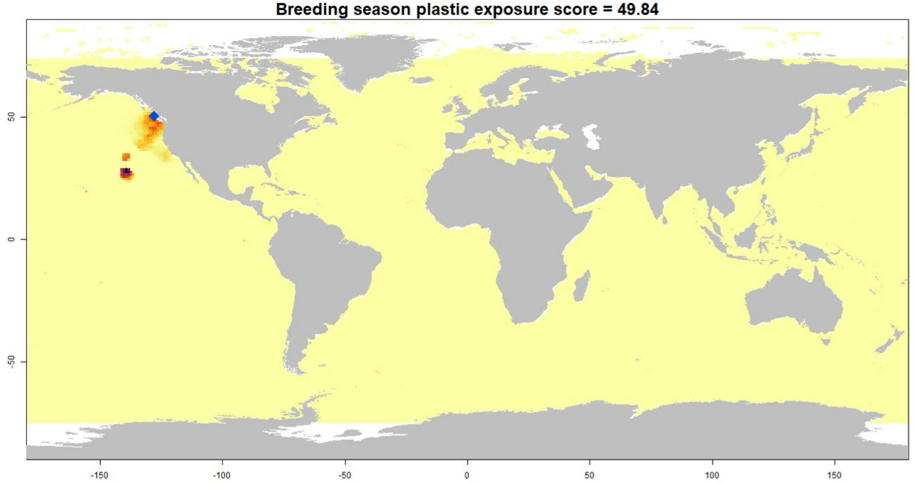

Leach's Storm-petrel Hydrobates leucorhous, Newfoundland
Breeding season plastic exposure score = 2.93

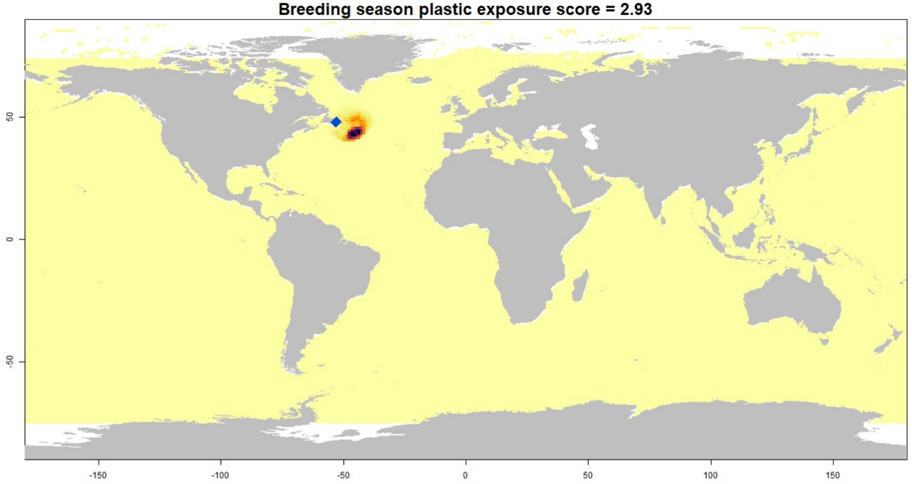

Cape Verde Storm-petrel Hydrobates jabejabe, Cape Verde
Breeding season plastic exposure score = 2.65

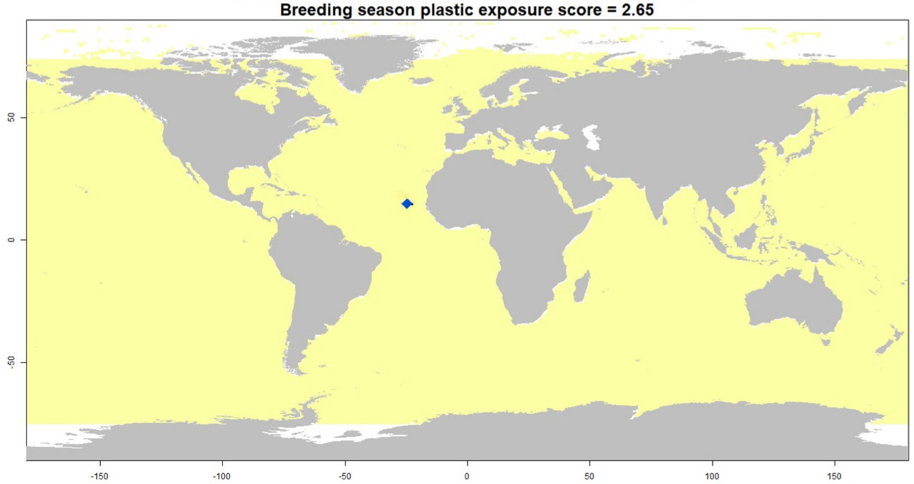

Leach's Storm-petrel Hydrobates leucorhous, Gillam Island
Non-breeding season plastic exposure score = 49.84

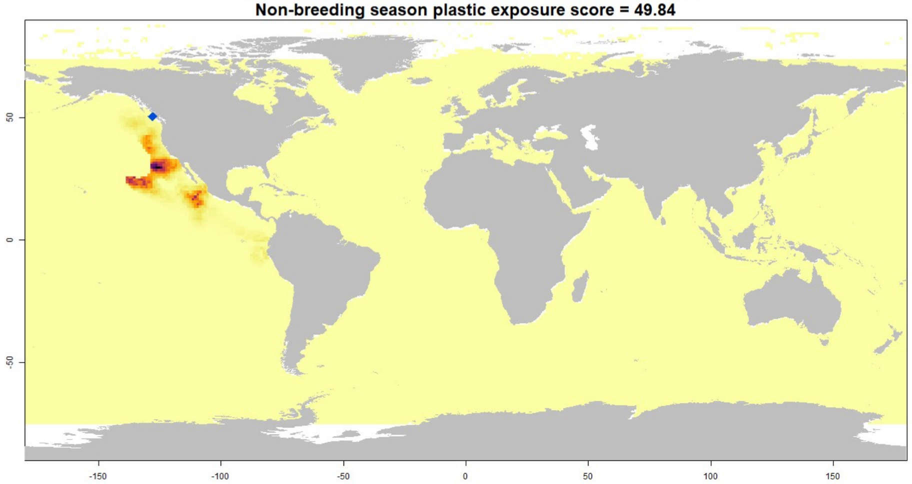

Monteiro's Storm-petrel Hydrobates monteiroi, Azores
Breeding season plastic exposure score = 15.91

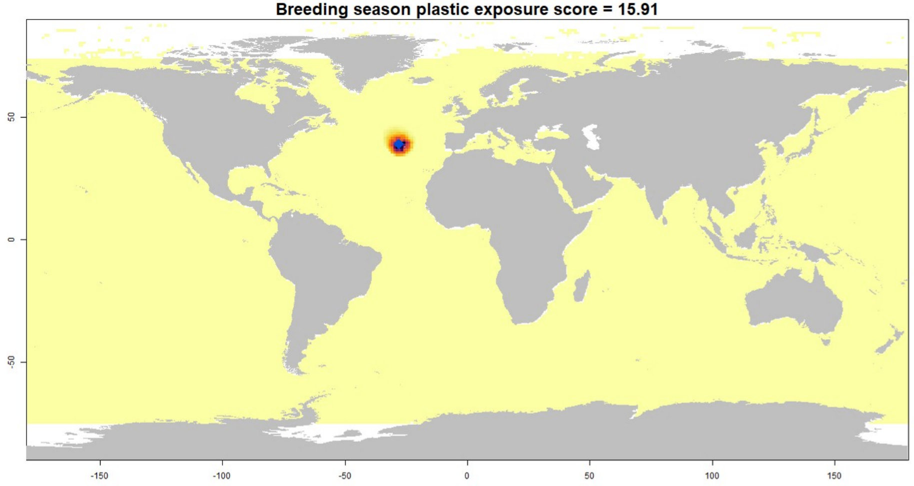

European Storm-petrel Hydrobates pelagicus, Capo caccia
Breeding season plastic exposure score = 306.12

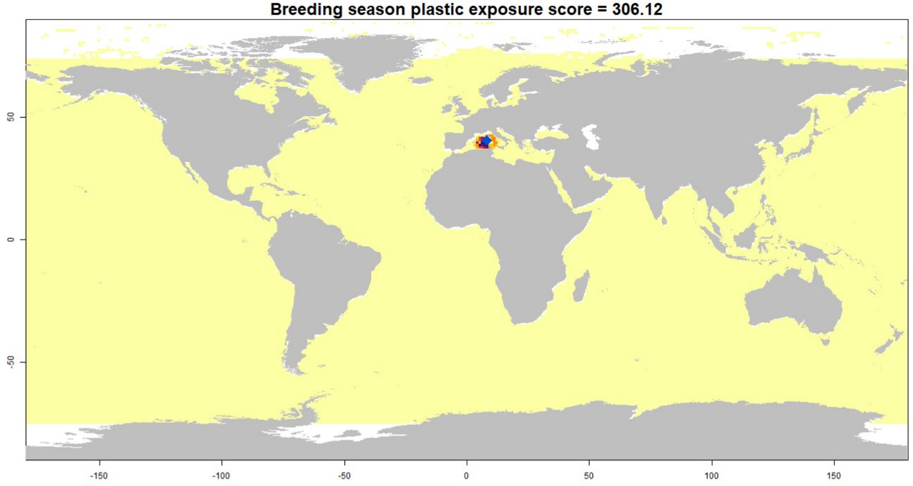

Leach's Storm-petrel Hydrobates leucorhous, Nova Scotia
Breeding season plastic exposure score = 10.51

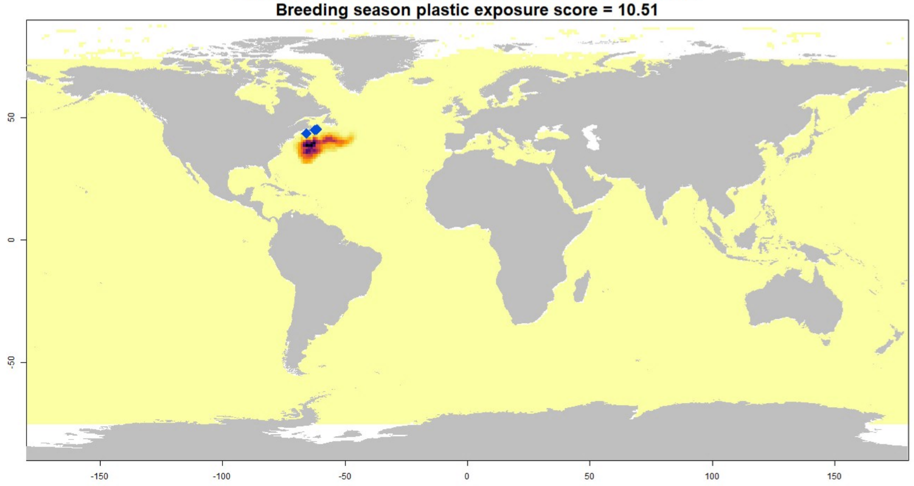

European Storm-petrel Hydrobates pelagicus, Benidorm Island
Breeding season plastic exposure score = 1039.67

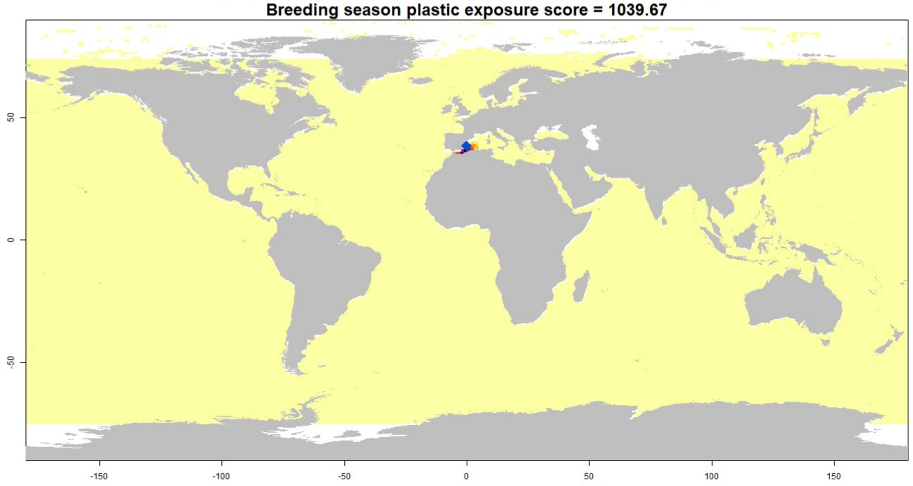

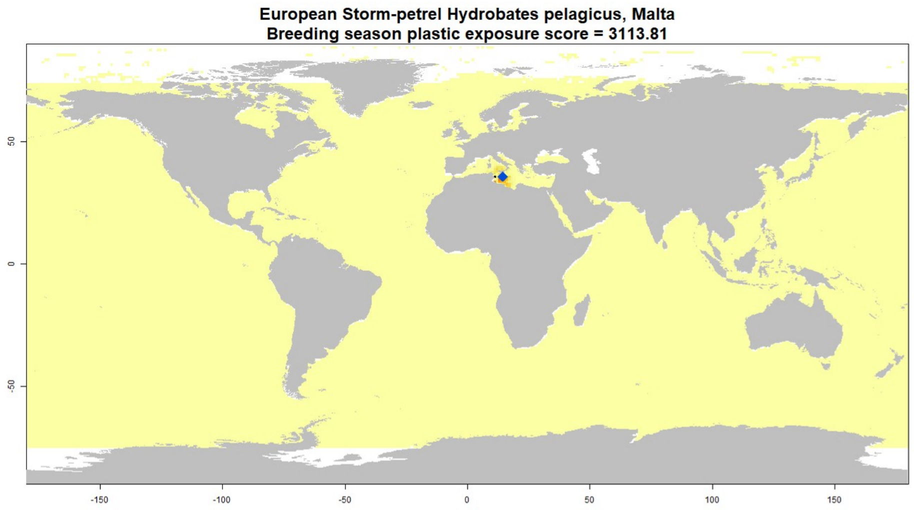
European Storm-petrel Hydrobates pelagicus, Malta
Breeding season plastic exposure score = 3113.81
50
0
-50
-150
-100
-50
0
50
100
150

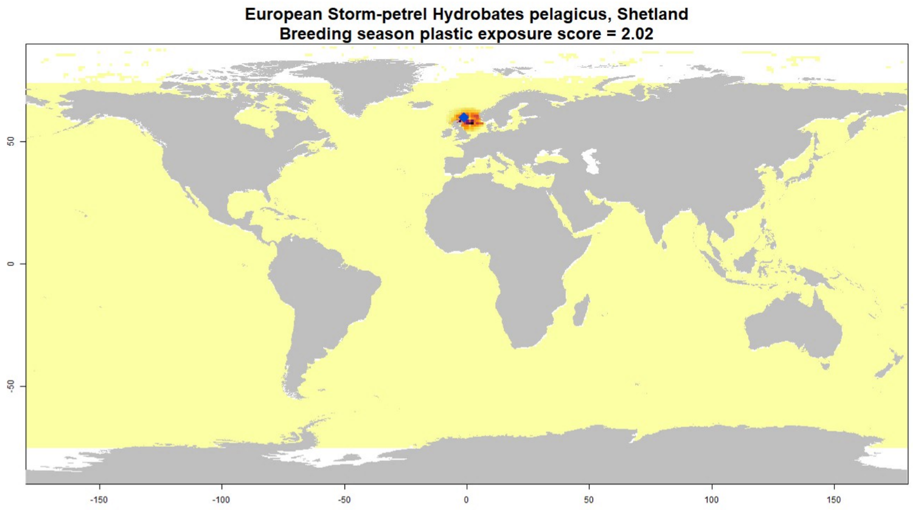
European Storm-petrel Hydrobates pelagicus, Shetland
Breeding season plastic exposure score = 2.02
50
0
-50
-150
-100
-50
0
50
100
150

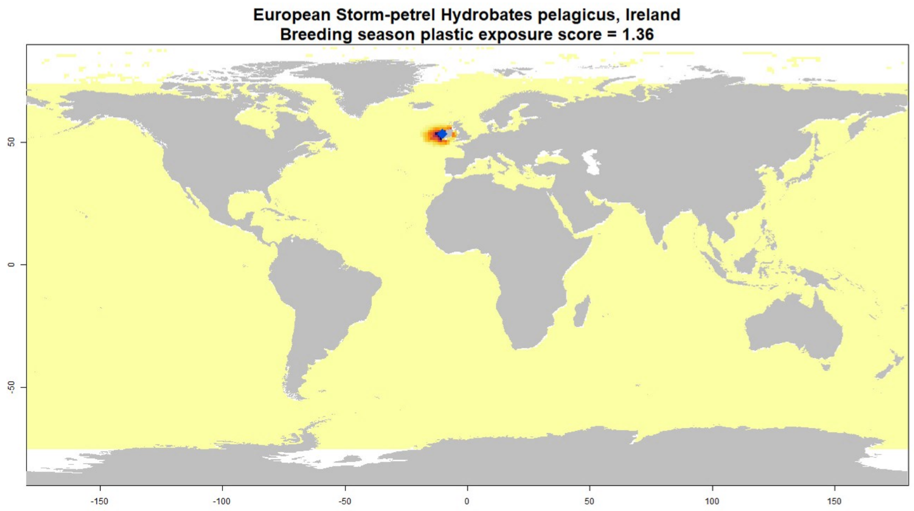
European Storm-petrel Hydrobates pelagicus, Ireland
Breeding season plastic exposure score = 1.36
50
0
-50
-150
-100
-50
0
50
100
150

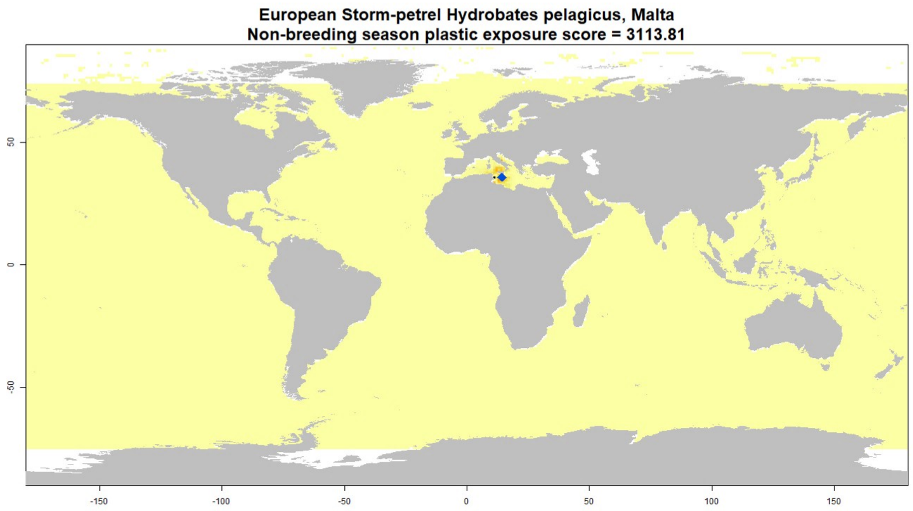
European Storm-petrel Hydrobates pelagicus, Malta
Non-breeding season plastic exposure score = 3113.81
50
0
-50
-150
-100
-50
0
50
100
150

Slender-billed Prion Pachyptila belcheri, Kerguelen
Breeding season plastic exposure score = 2.53

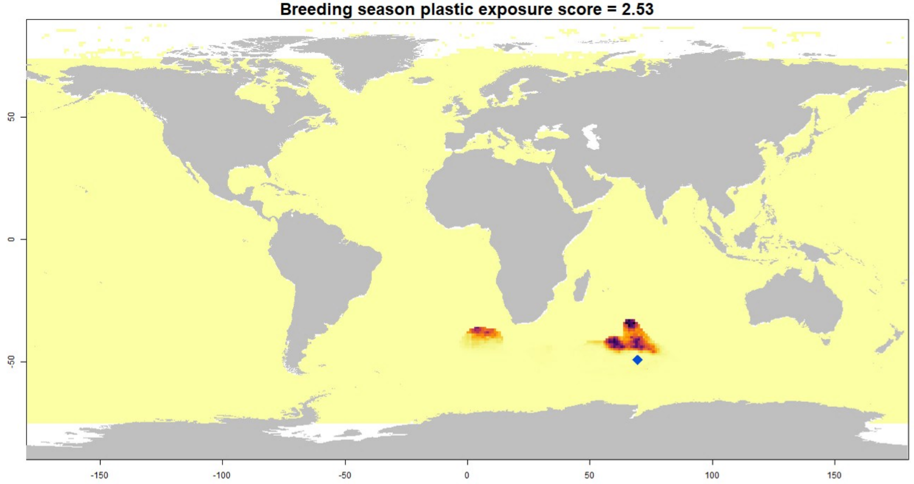

Slender-billed Prion Pachyptila belcheri, New Island
Breeding season plastic exposure score = 2.23

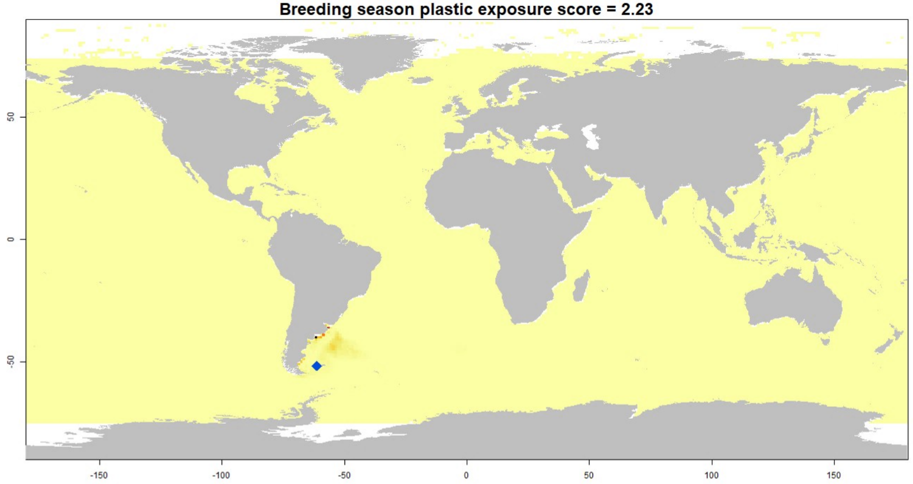

Wilson's Storm-petrel Oceanites oceanicus, South Shetland Islands
Breeding season plastic exposure score = 0.01

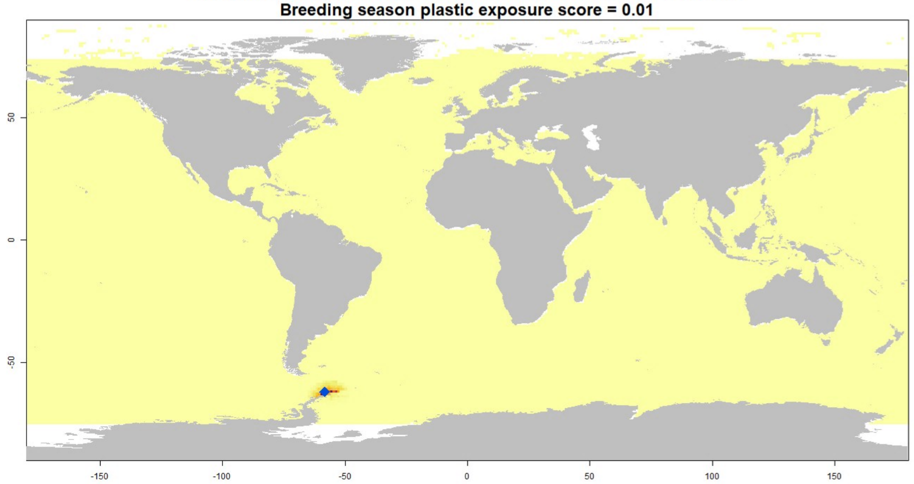

Slender-billed Prion Pachyptila belcheri, Kerguelen
Non-breeding season plastic exposure score = 2.53

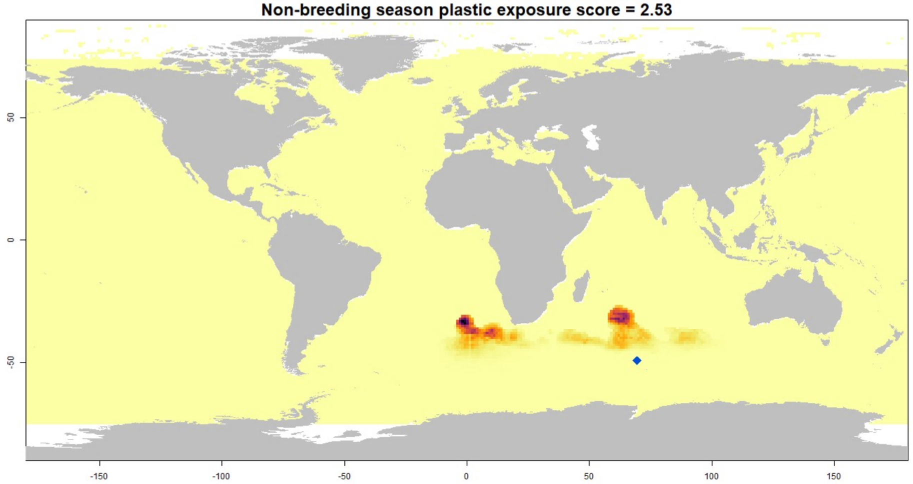

Antarctic Prion Pachyptila desolata, Kerguelen
Breeding season plastic exposure score = 14.46

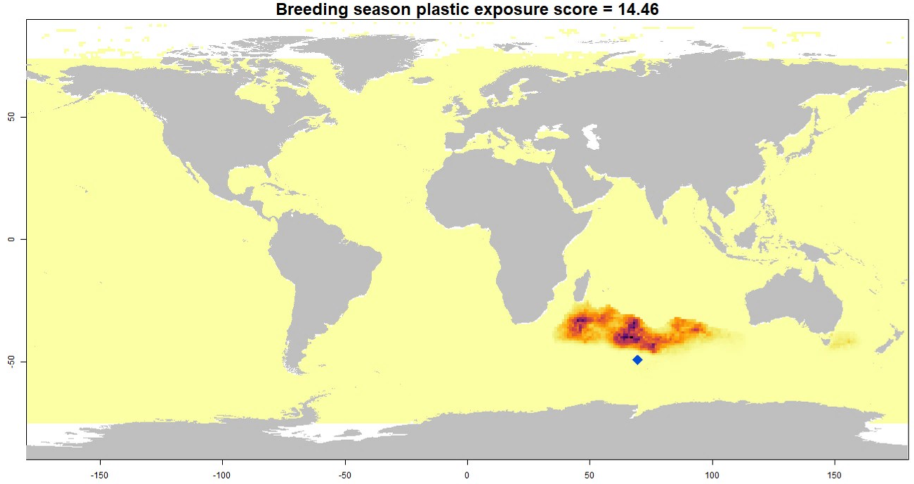

Antarctic Prion Pachyptila desolata, South Georgia
Breeding season plastic exposure score = 1.75

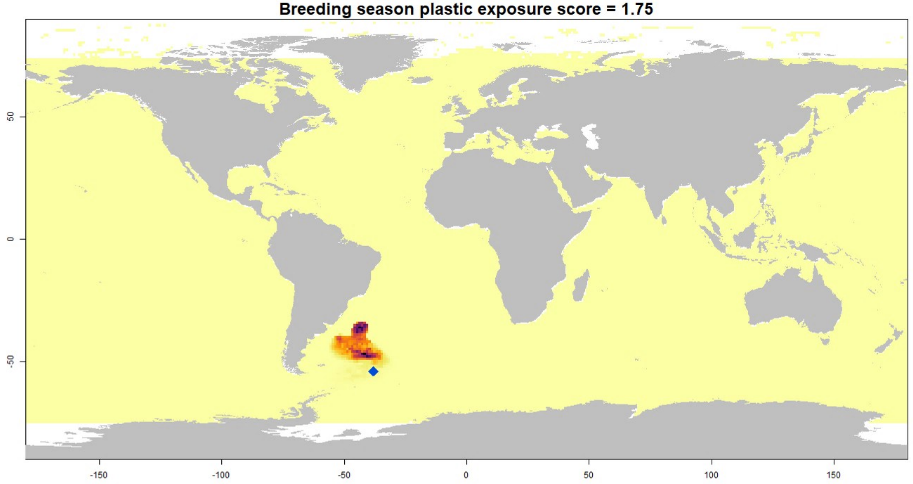

Slender-billed Prion Pachyptila belcheri, New Island
Non-breeding season plastic exposure score = 2.23

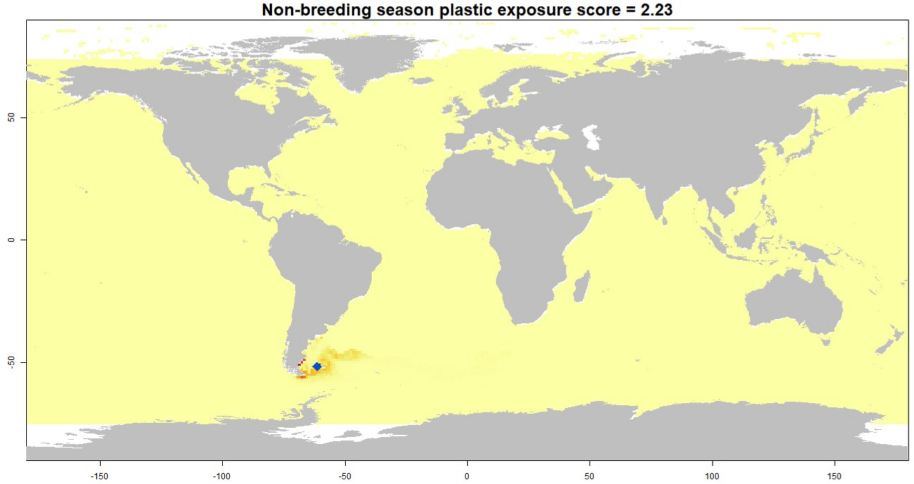

Antarctic Prion Pachyptila desolata, Kerguelen
Non-breeding season plastic exposure score = 14.46

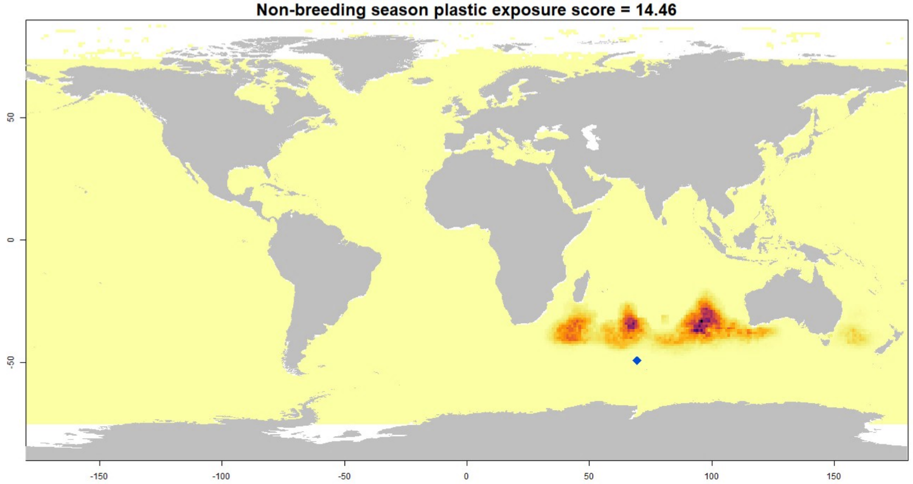

MacGillivray's Prion Pachyptila macgillivrayi, Gough
Breeding season plastic exposure score = 70.22

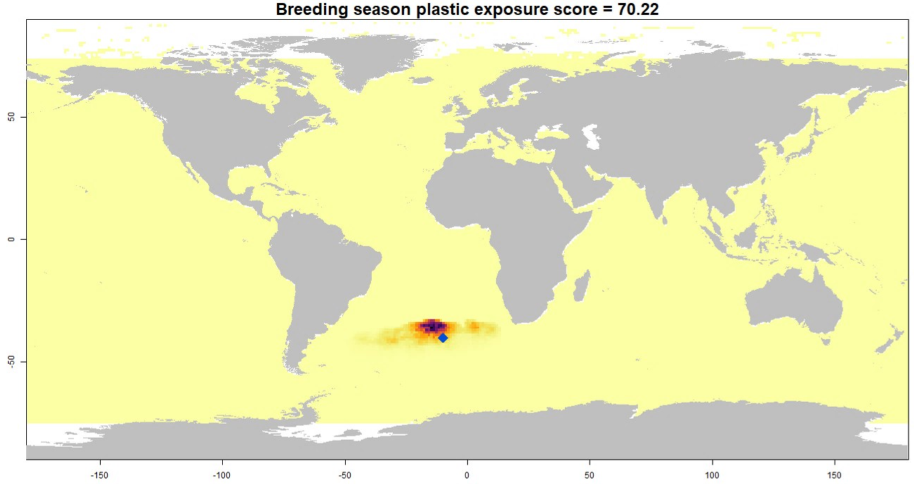

Fairy Prion Pachyptila turtur, Dunedin
Breeding season plastic exposure score = 13.51

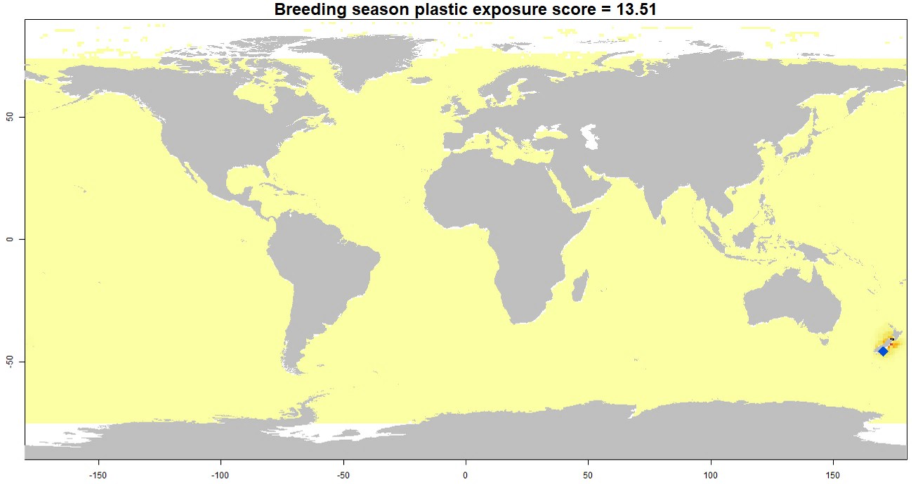

Antarctic Prion Pachyptila desolata, South Georgia
Non-breeding season plastic exposure score = 1.75

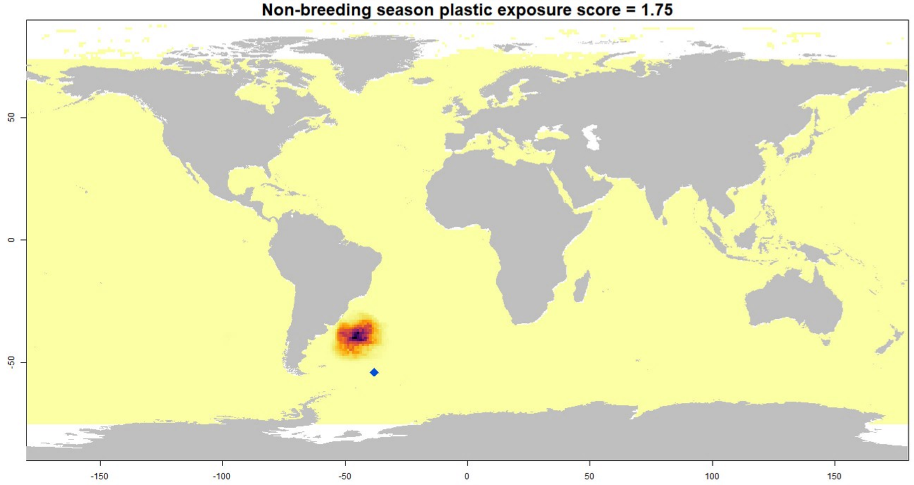

MacGillivray's Prion Pachyptila macgillivrayi, Gough
Non-breeding season plastic exposure score = 70.22

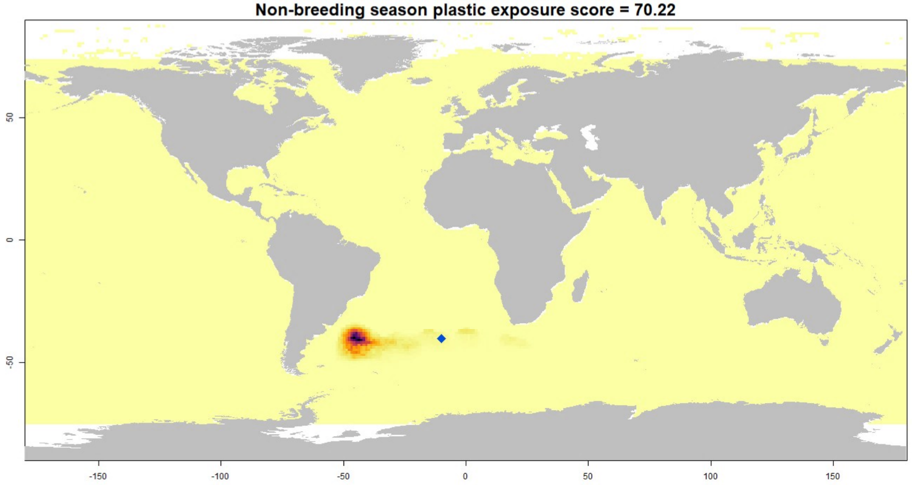

Fairy Prion Pachyptila turtur, Kanowna Island
Breeding season plastic exposure score = 23.61

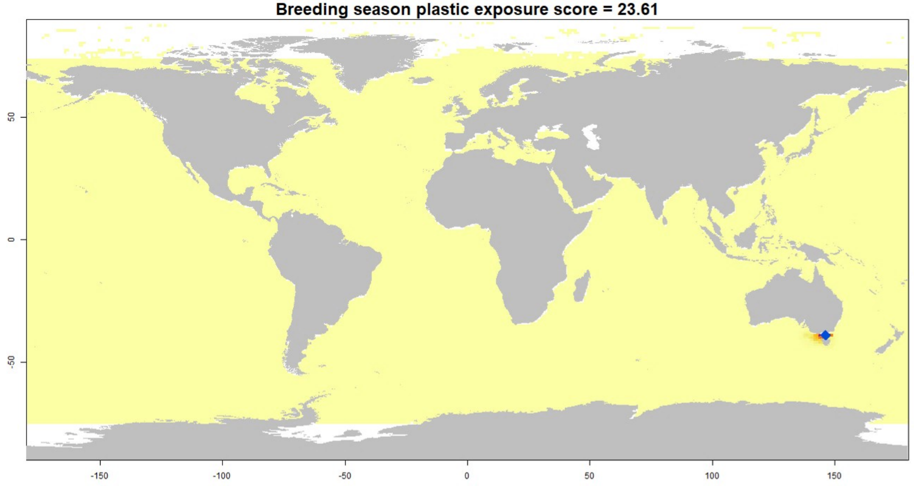

Broad-billed Prion Pachyptila vittata, Gough
Non-breeding season plastic exposure score = 203.85

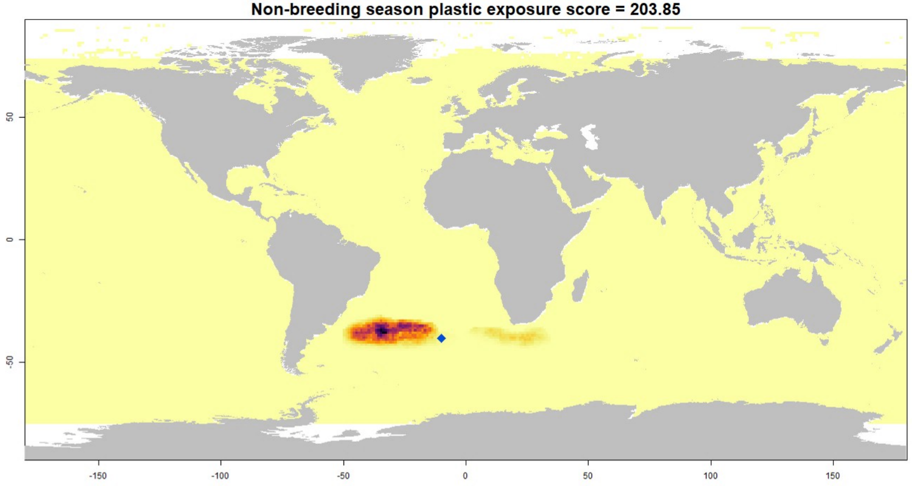

Fairy Prion Pachyptila turtur, Dunedin
Non-breeding season plastic exposure score = 13.51

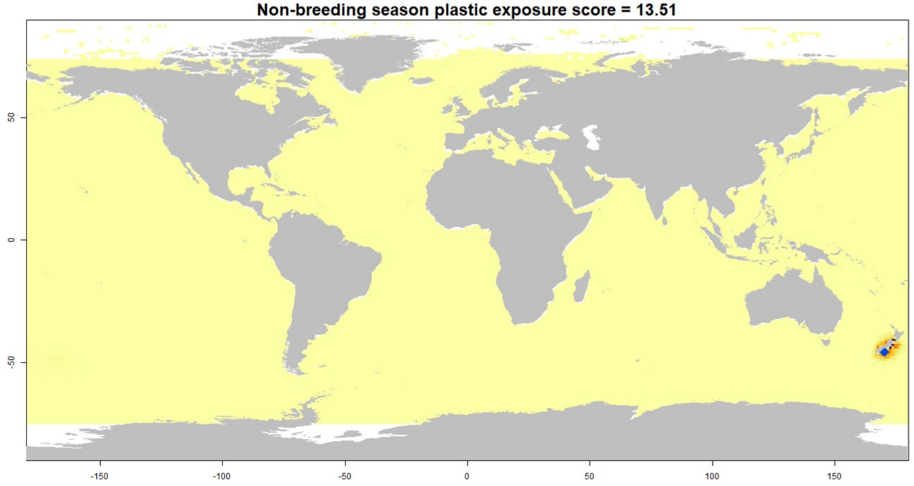

Broad-billed Prion Pachyptila vittata, Gough
Breeding season plastic exposure score = 203.85

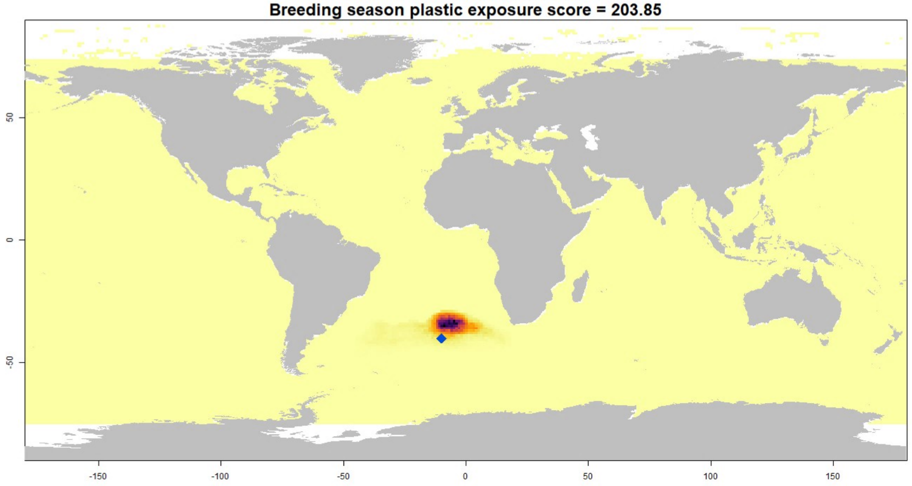

Broad-billed Prion Pachyptila vittata, Tristan da Cunha
Non-breeding season plastic exposure score = 231.54

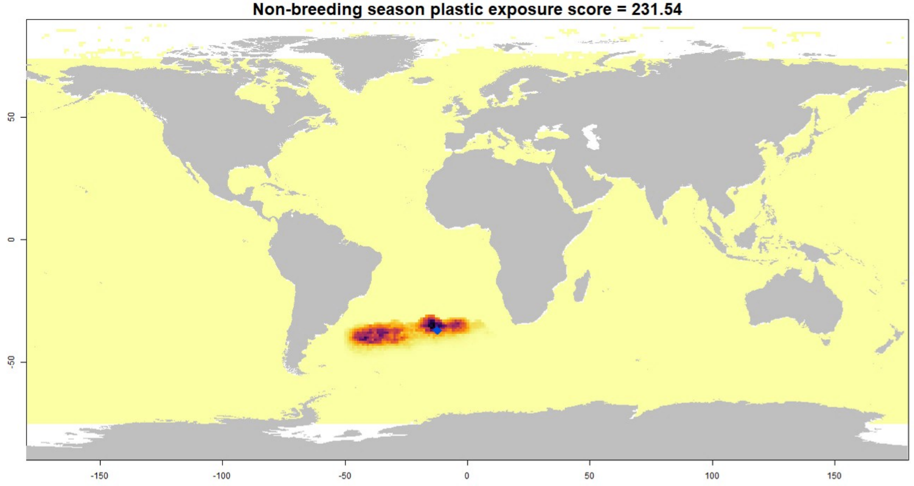

Snow Petrel Pagodroma nivea, Pointe Geologie
Non-breeding season plastic exposure score = 0

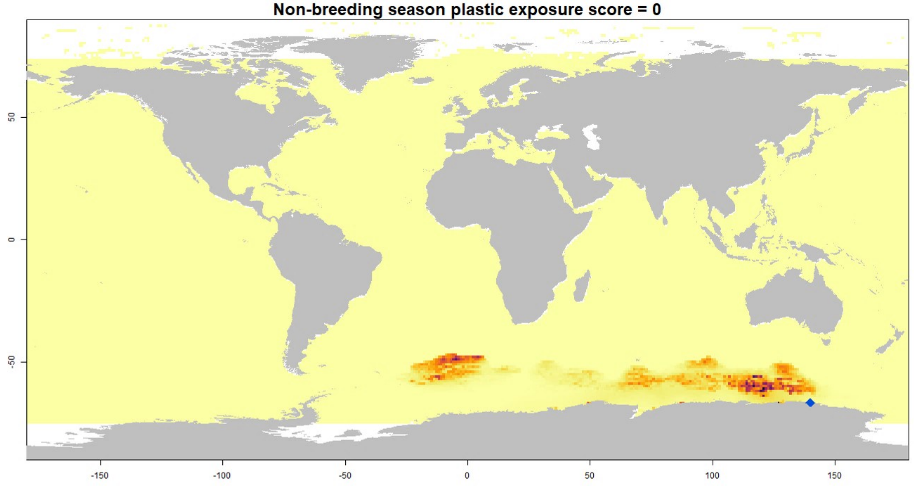

Broad-billed Prion Pachyptila vittata, Tristan da Cunha
Breeding season plastic exposure score = 231.54

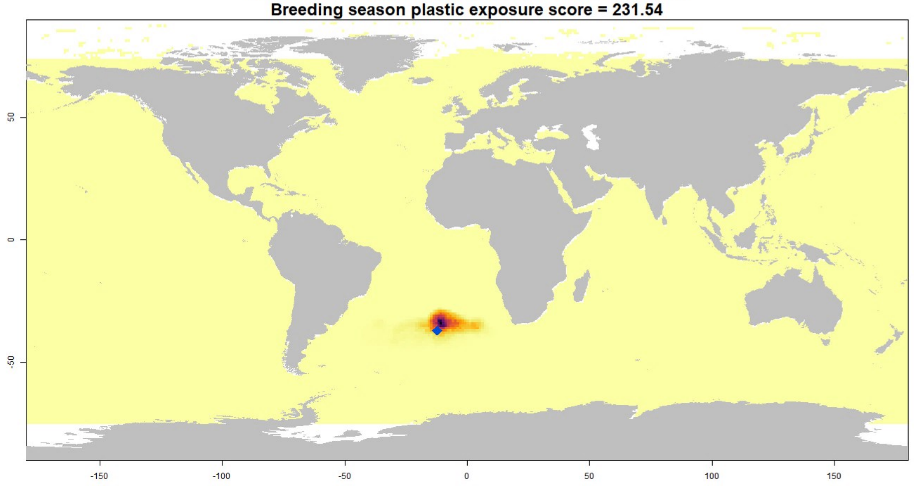

Snow Petrel Pagodroma nivea, Pointe Geologie
Breeding season plastic exposure score = 0

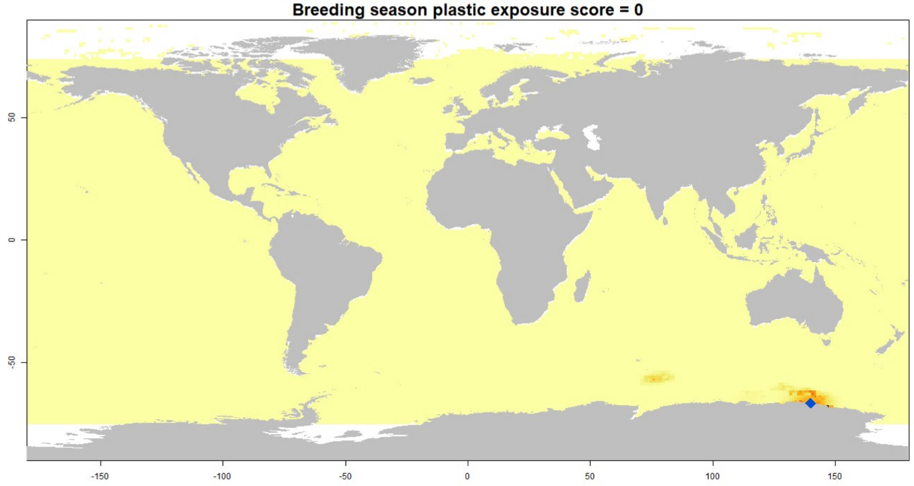

White-faced Storm-petrel Pelagodroma marina, Madeira
Breeding season plastic exposure score = 14.37

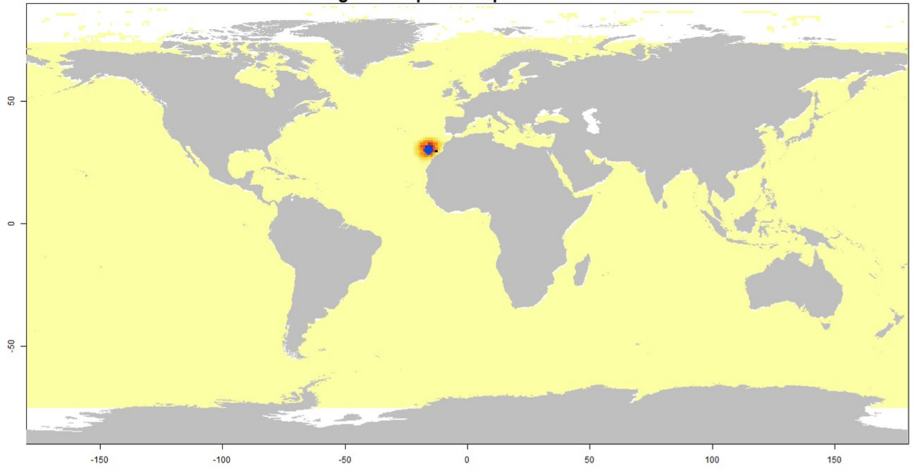

South Georgia Diving-petrel Pelecanoides georgicus, South Georgia
Breeding season plastic exposure score = 1.88

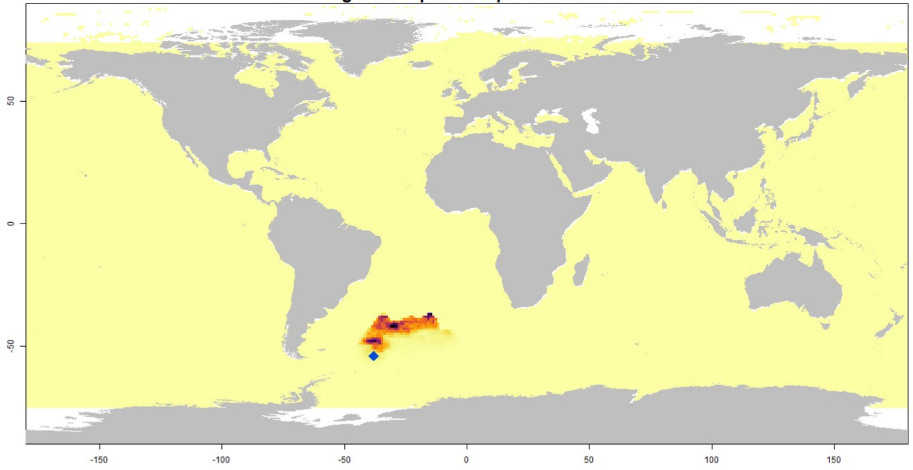

White-faced Storm-petrel Pelagodroma marina, Cape Verde
Breeding season plastic exposure score = 0.7

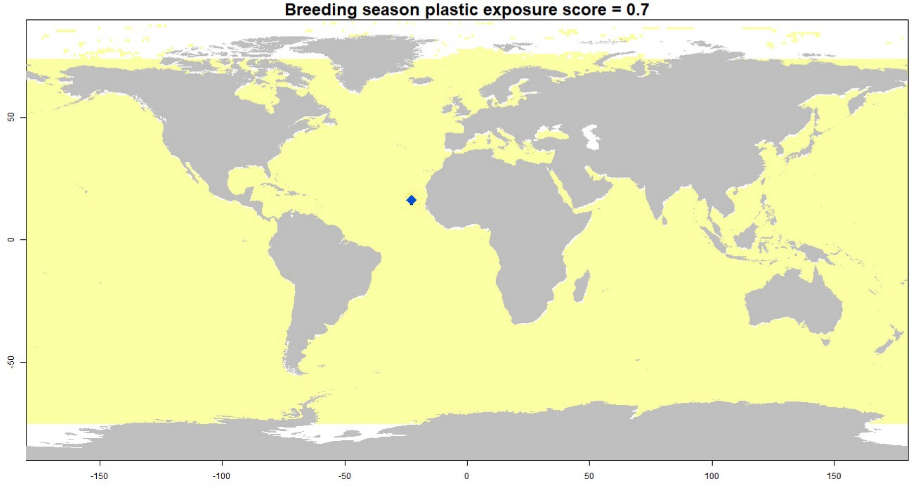

Peruvian Diving-petrel Pelecanoides garnotii, Paracas
Breeding season plastic exposure score = 0.18

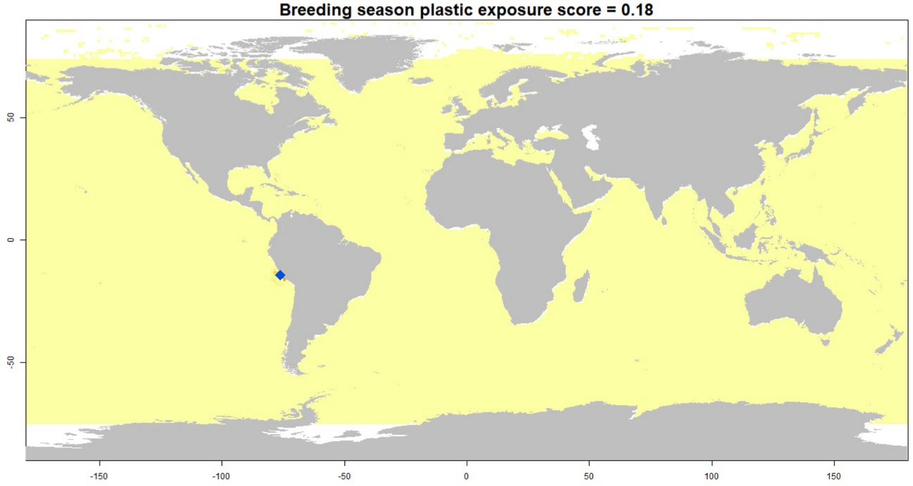

Common Diving-petrel Pelecanoides urinatrix, Kanowna Island
Breeding season plastic exposure score = 38.88

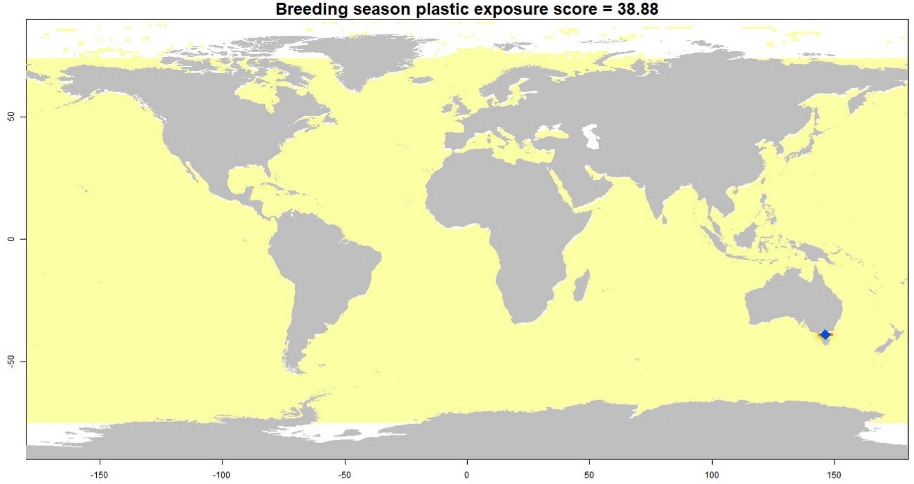

Common Diving-petrel Pelecanoides urinatrix, North Island
Non-breeding season plastic exposure score = 40.02

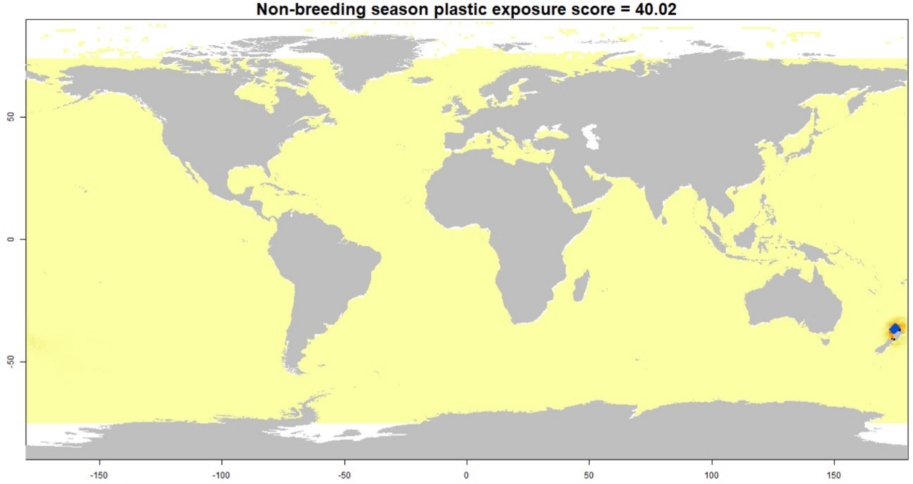

South Georgia Diving-petrel Pelecanoides georgicus, South Georgia
Non-breeding season plastic exposure score = 1.88

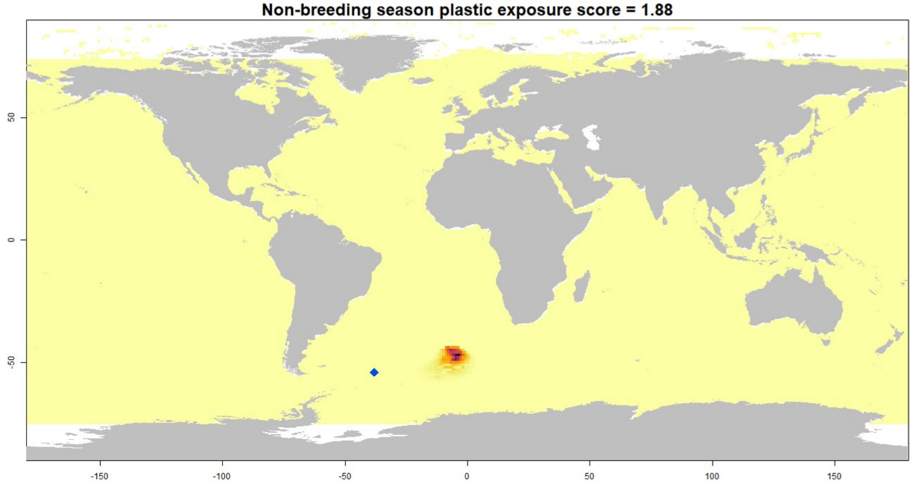

Common Diving-petrel Pelecanoides urinatrix, North Island
Breeding season plastic exposure score = 40.02

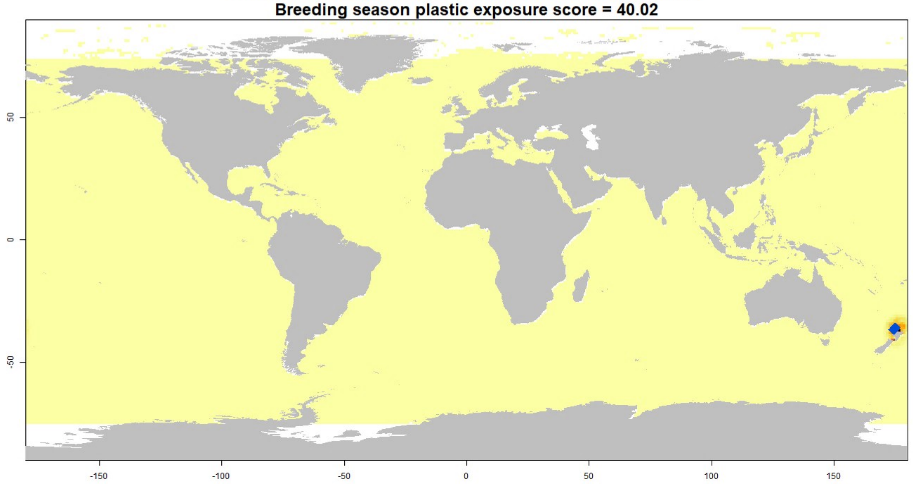

Common Diving-petrel Pelecanoides urinatrix, South Georgia
Non-breeding season plastic exposure score = 0.37

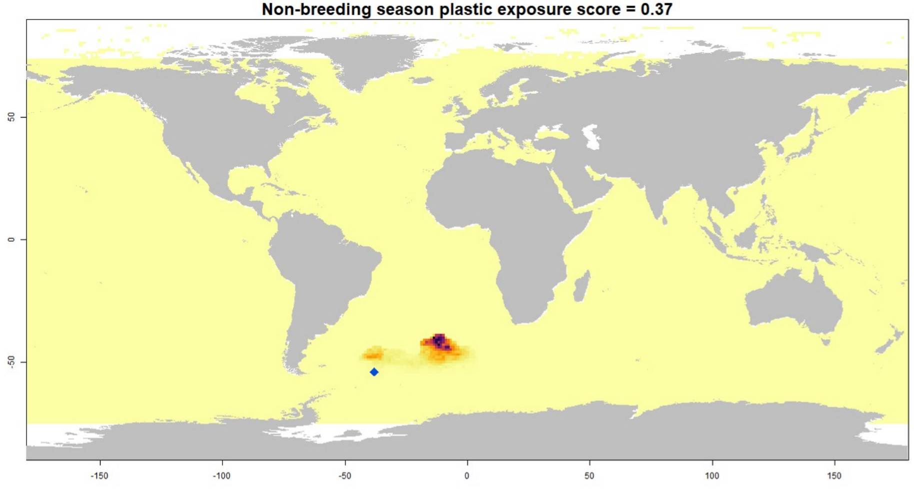

Whenua Hou Diving-petrel Pelecanoides whenuahouensis, Codfish Island
Non-breeding season plastic exposure score = 3.44

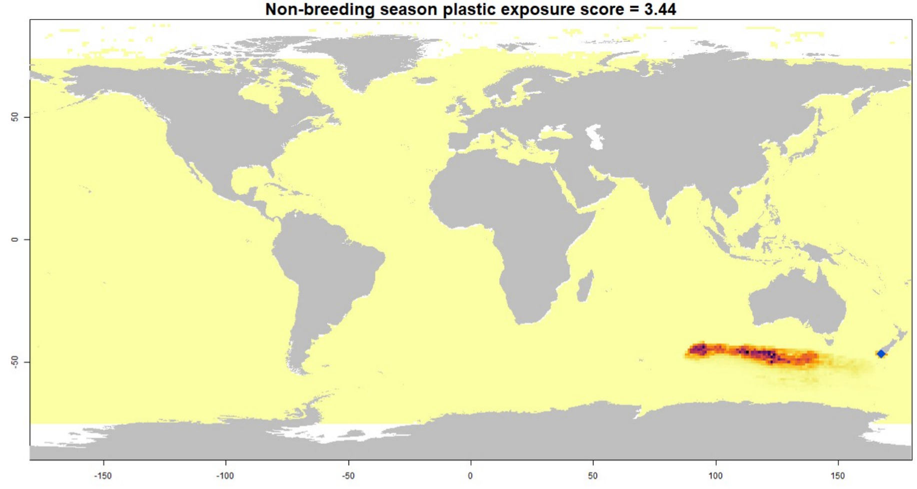

Common Diving-petrel Pelecanoides urinatrix, South Georgia
Breeding season plastic exposure score = 0.37

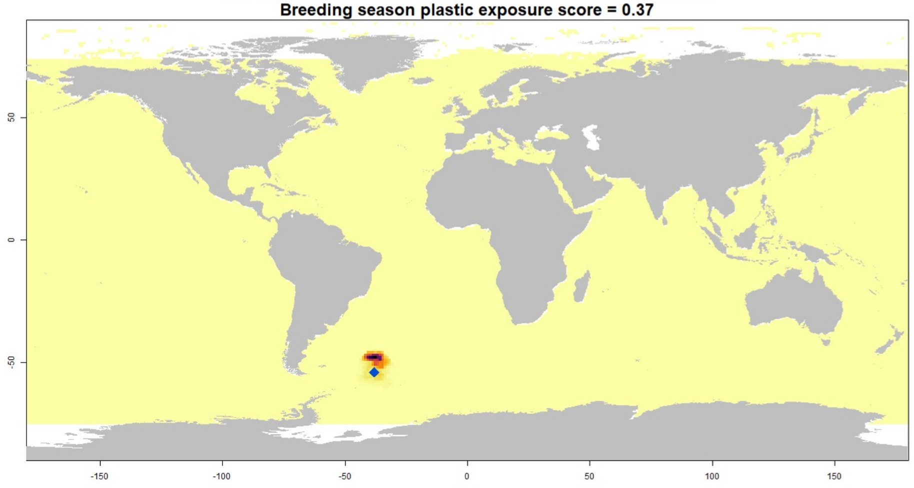

Whenua Hou Diving-petrel Pelecanoides whenuahouensis, Codfish Island
Breeding season plastic exposure score = 3.44

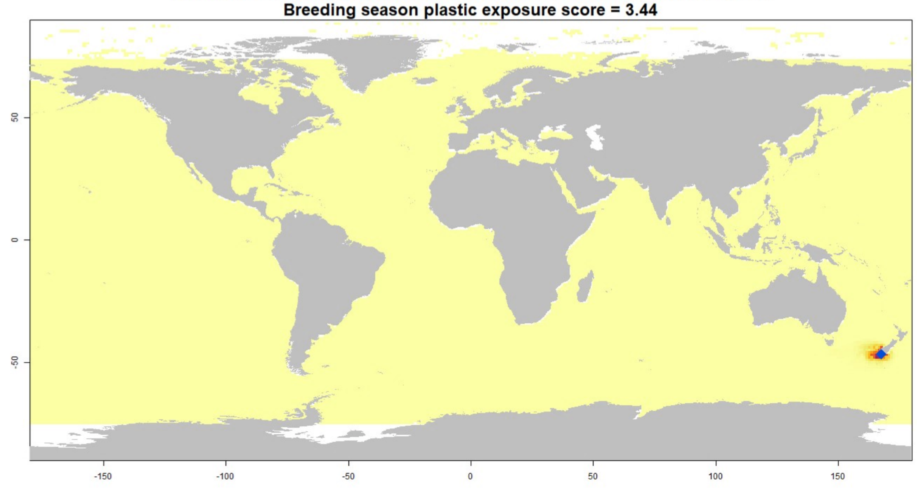

White-chinned Petrel Procellaria aequinoctialis, Antipodes Islands
Non-breeding season plastic exposure score = 12.51

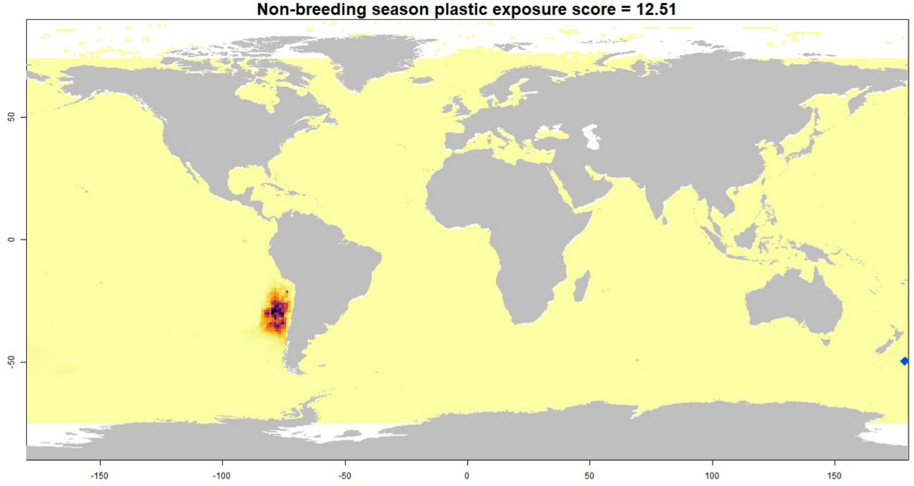

White-chinned Petrel Procellaria aequinoctialis, Crozet
Non-breeding season plastic exposure score = 52.55

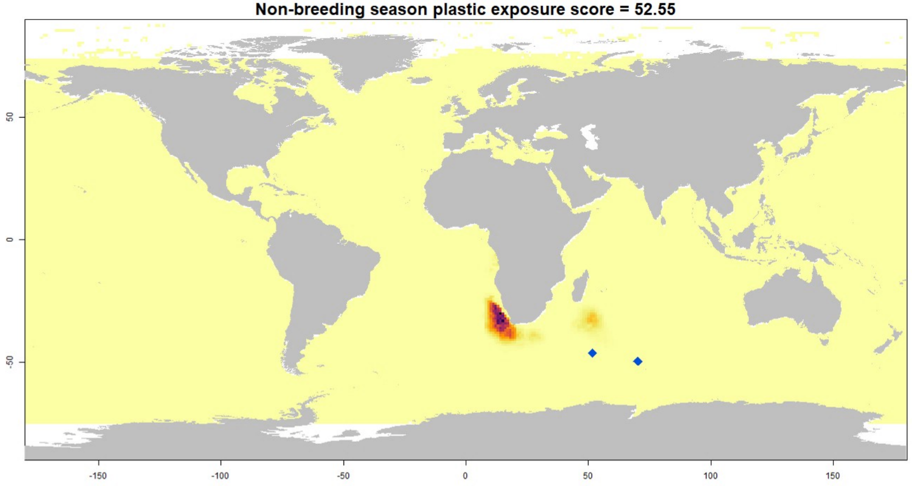

White-chinned Petrel Procellaria aequinoctialis, Antipodes Islands
Breeding season plastic exposure score = 12.51

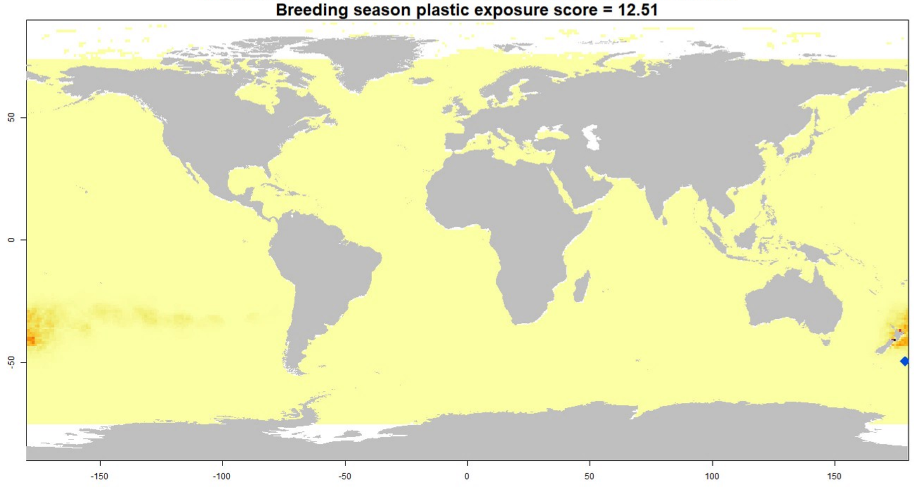

White-chinned Petrel Procellaria aequinoctialis, Crozet
Breeding season plastic exposure score = 52.55

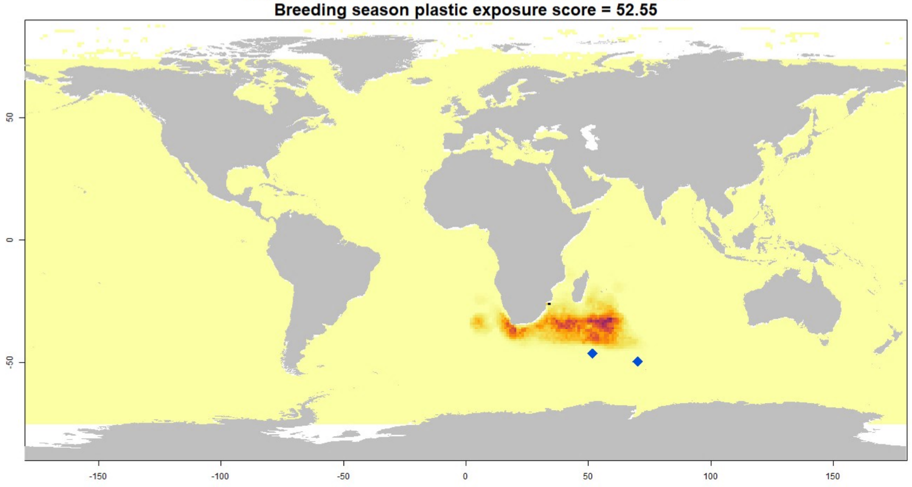

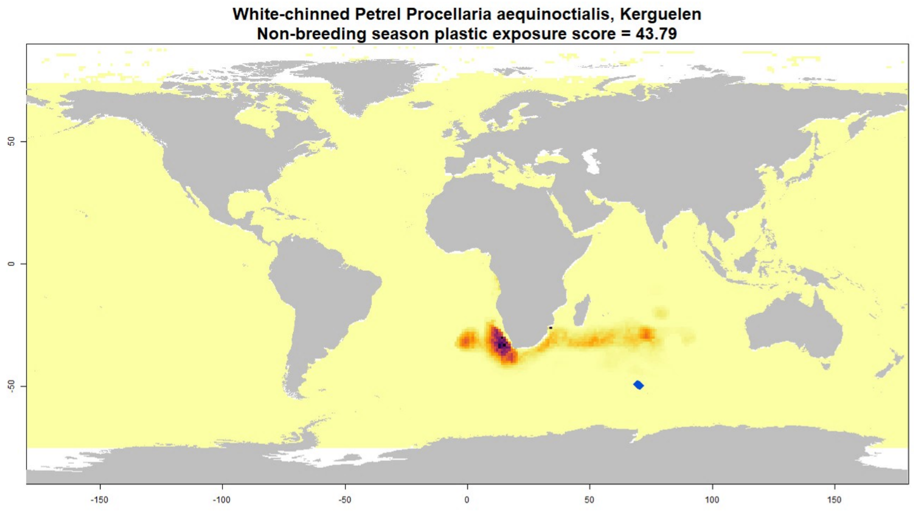
White-chinned Petrel Procellaria aequinoctialis, Kerguelen
Non-breeding season plastic exposure score = 43.79
50
0
-50
-150
-100
-50
0
50
100
150

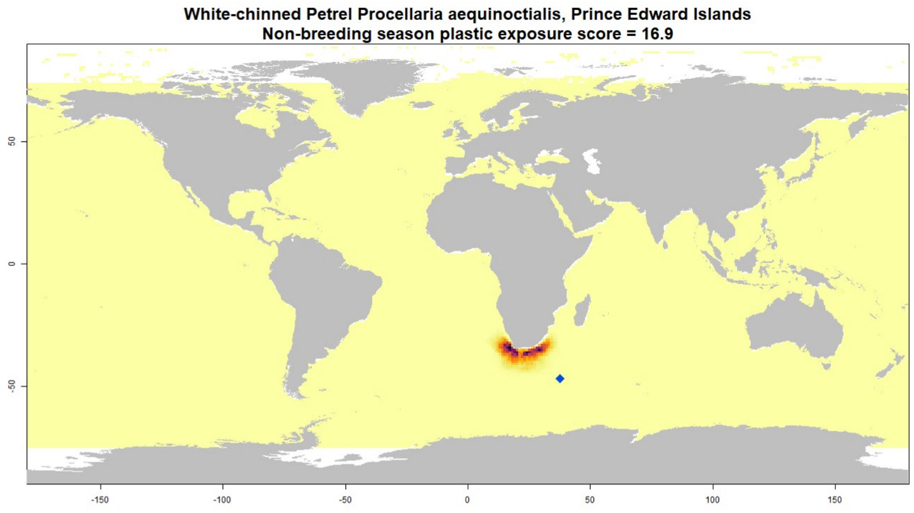
White-chinned Petrel Procellaria aequinoctialis, Prince Edward Islands
Non-breeding season plastic exposure score = 16.9
50
0
-50
-150
-100
-50
0
50
100
150

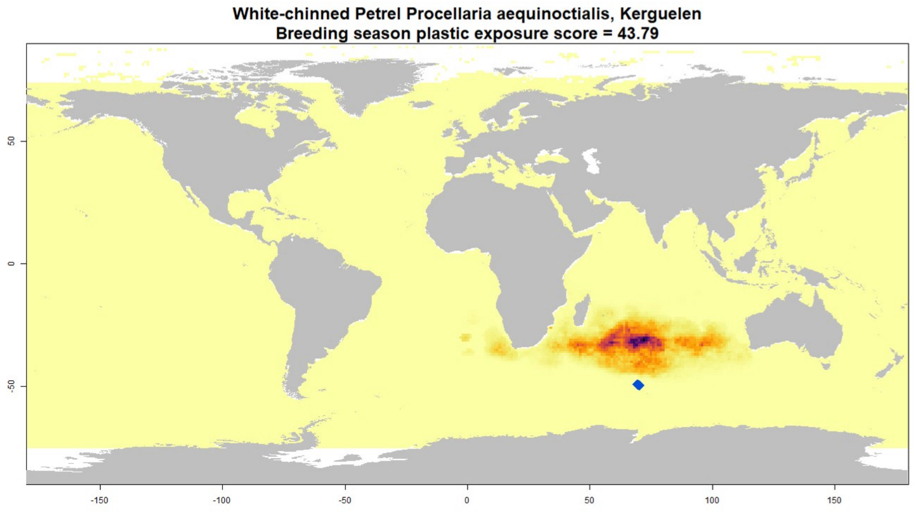
White-chinned Petrel Procellaria aequinoctialis, Kerguelen
Breeding season plastic exposure score = 43.79
50
0
-50
-150
-100
-50
0
50
100
150

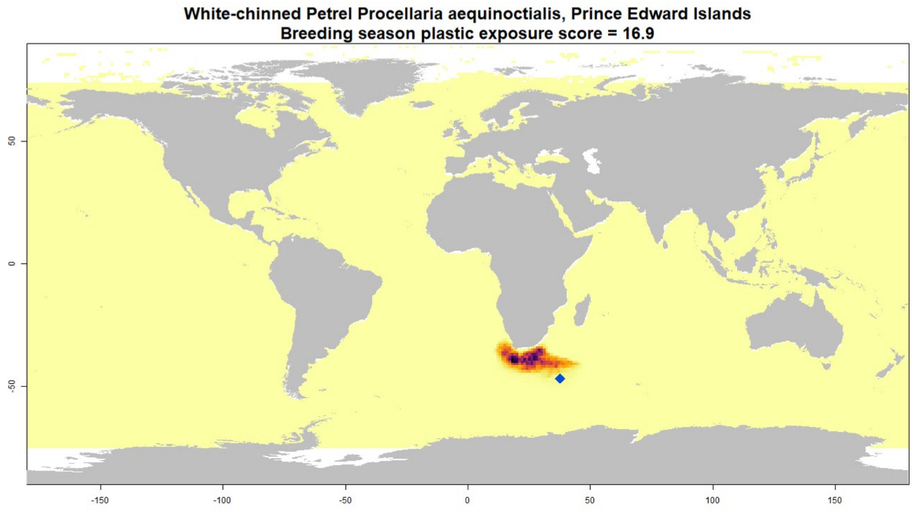
White-chinned Petrel Procellaria aequinoctialis, Prince Edward Islands
Breeding season plastic exposure score = 16.9
50
0
-50
-150
-100
-50
0
50
100
150

White-chinned Petrel Procellaria aequinoctialis, South Georgia
Non-breeding season plastic exposure score = 5.53

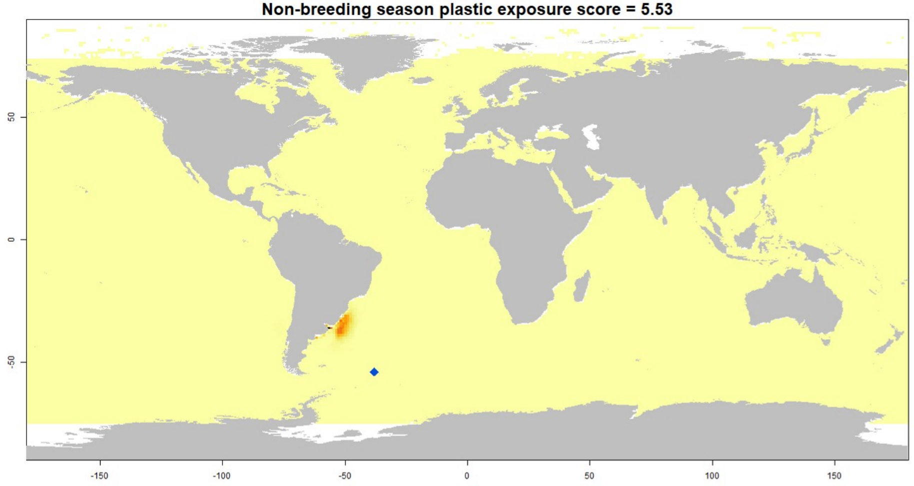

Grey Petrel Procellaria cinerea, Antipodes Islands
Non-breeding season plastic exposure score = 3.65

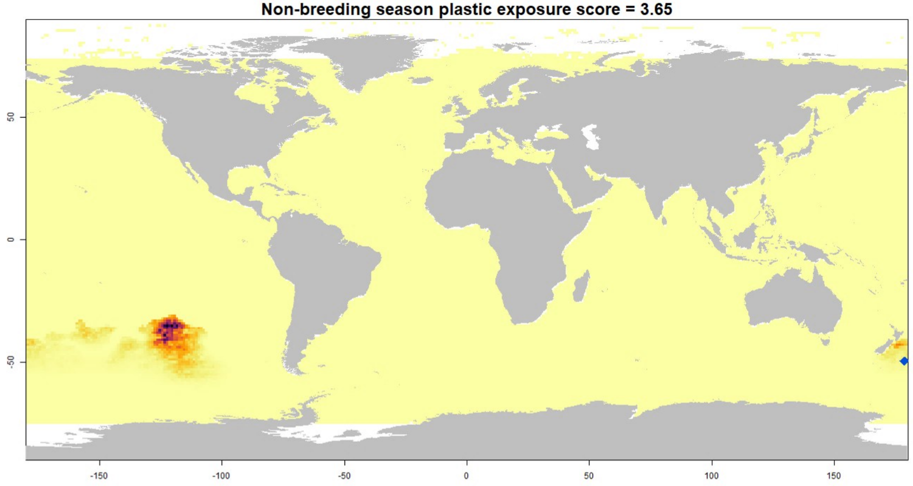

White-chinned Petrel Procellaria aequinoctialis, South Georgia
Breeding season plastic exposure score = 5.53

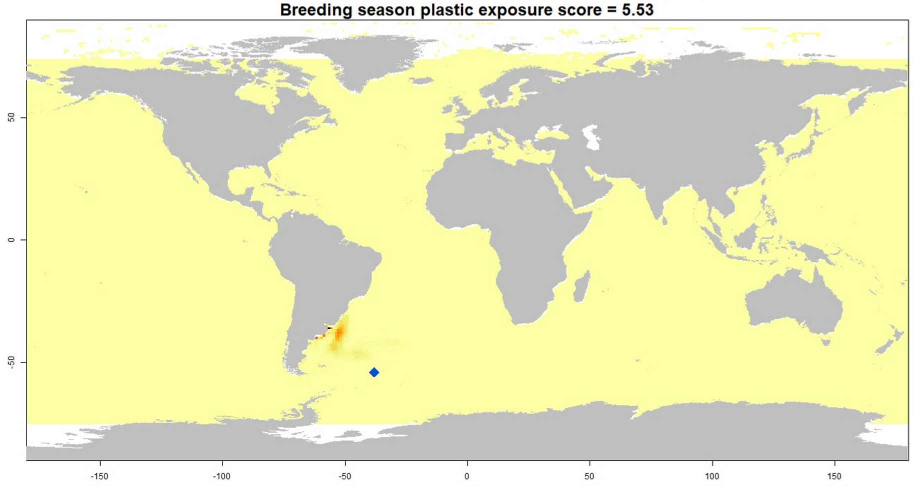

Grey Petrel Procellaria cinerea, Antipodes Islands
Breeding season plastic exposure score = 3.65

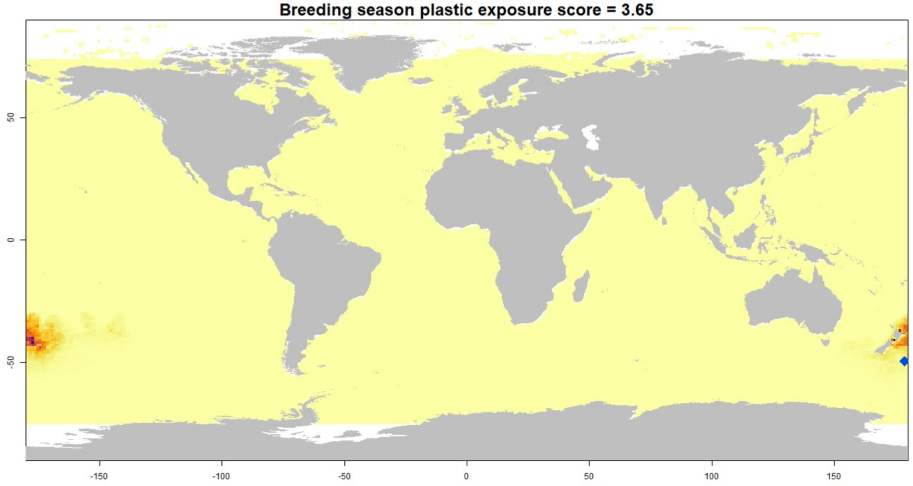

Grey Petrel Procellaria cinerea, Gough
Non-breeding season plastic exposure score = 44.75

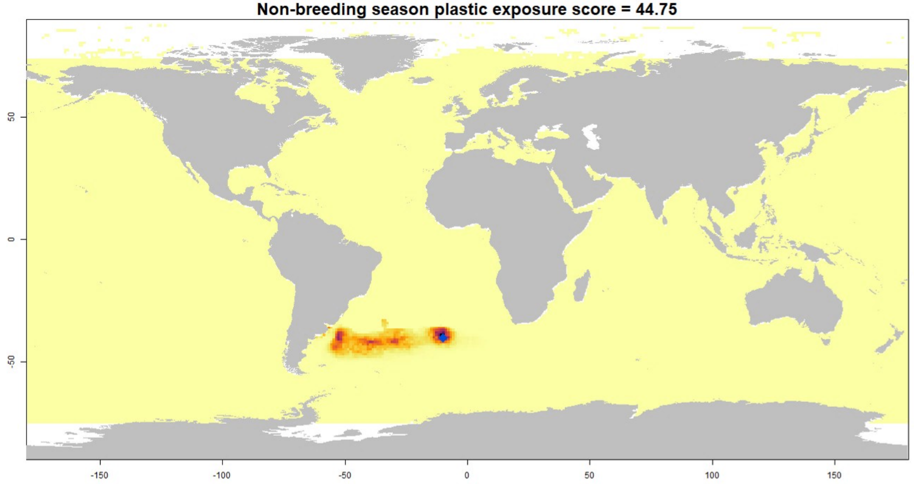

Grey Petrel Procellaria cinerea, Kerguelen
Non-breeding season plastic exposure score = 15.75

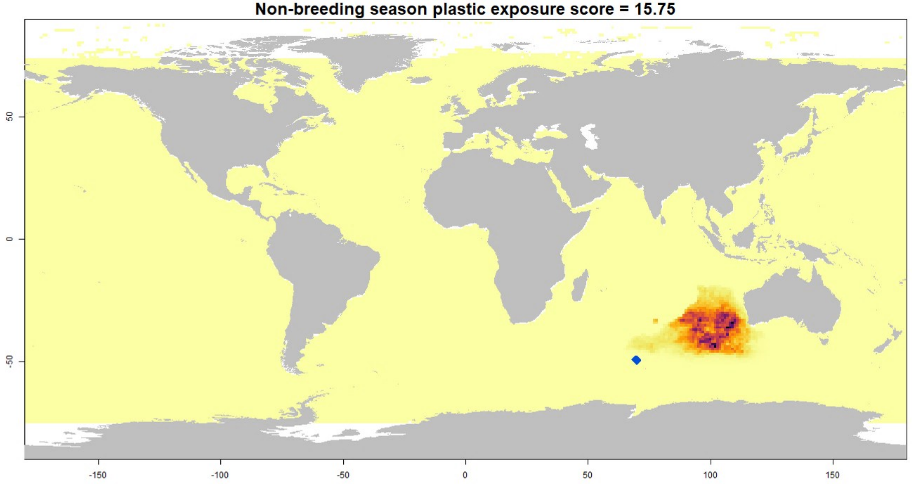

Grey Petrel Procellaria cinerea, Gough
Breeding season plastic exposure score = 44.75

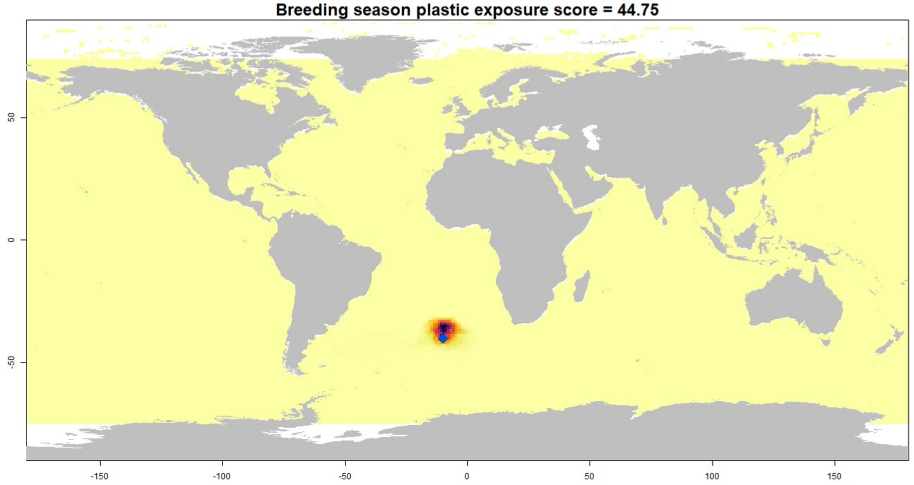

Grey Petrel Procellaria cinerea, Kerguelen
Breeding season plastic exposure score = 15.75

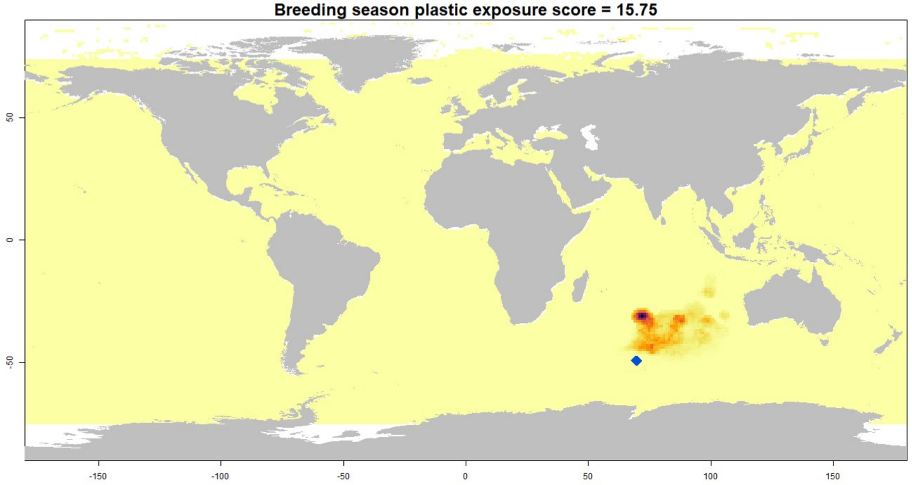

Grey Petrel Procellaria cinerea, Prince Edward Islands
Non-breeding season plastic exposure score = 14.13

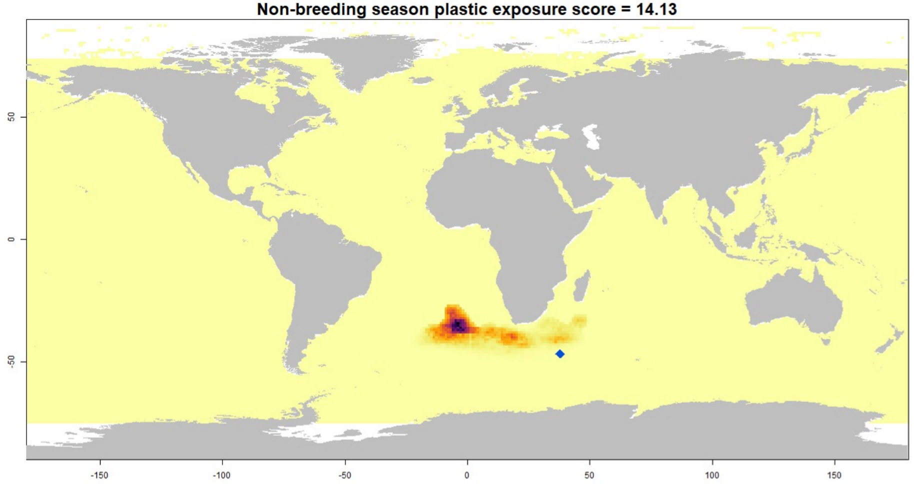

Spectacled Petrel Procellaria conspicillata, Tristan da Cunha
Non-breeding season plastic exposure score = 247.77

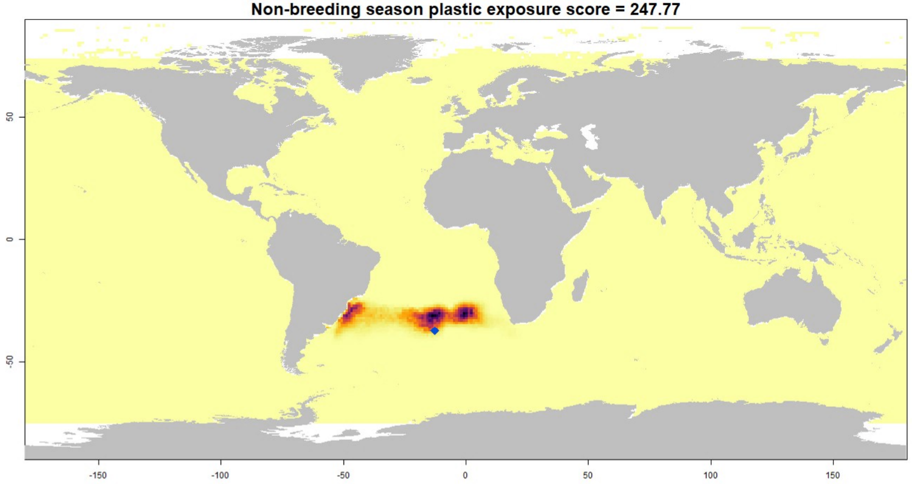

Grey Petrel Procellaria cinerea, Prince Edward Islands
Breeding season plastic exposure score = 14.13

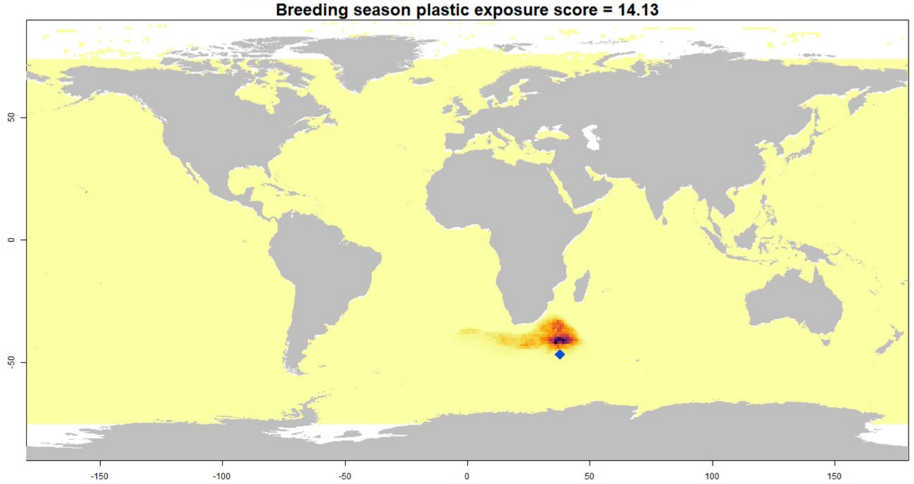

Spectacled Petrel Procellaria conspicillata, Tristan da Cunha
Breeding season plastic exposure score = 247.77

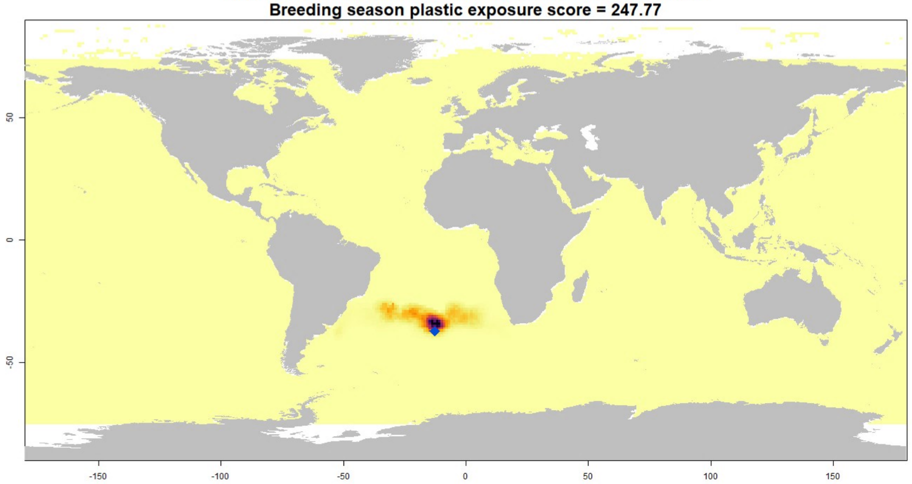

Black Petrel Procellaria parkinsoni, Great Barrier Island
Non-breeding season plastic exposure score = 14.95

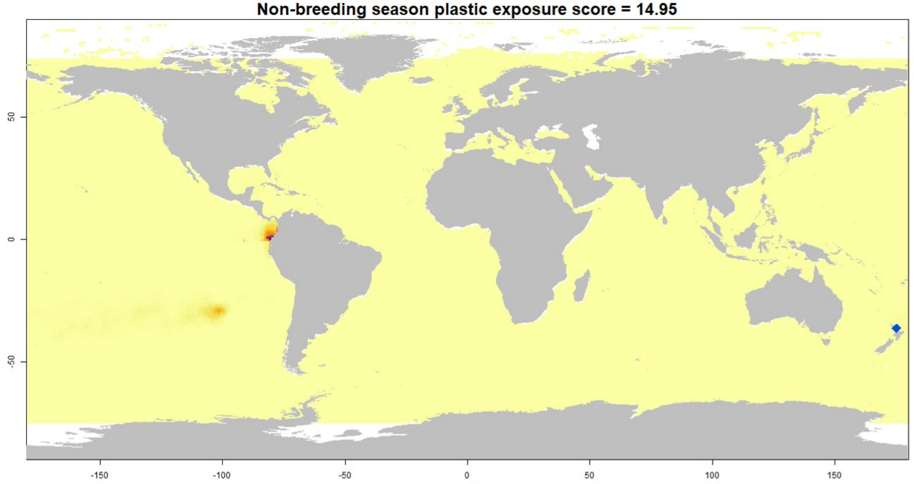

Westland Petrel Procellaria westlandica, Punakaiki
Non-breeding season plastic exposure score = 23.48

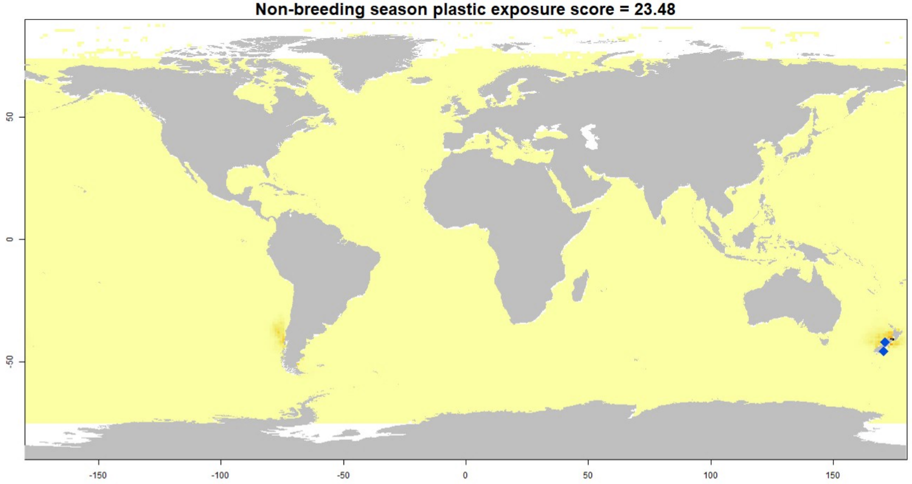

Black Petrel Procellaria parkinsoni, Great Barrier Island
Breeding season plastic exposure score = 14.95

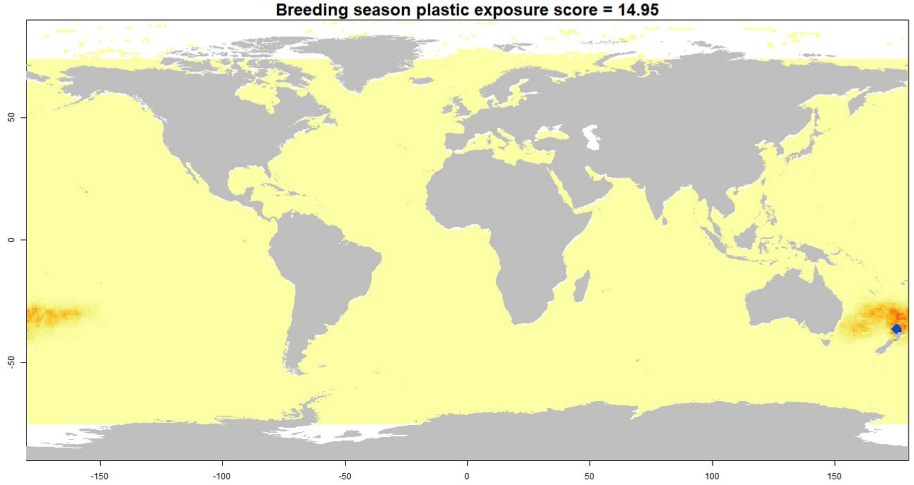

Westland Petrel Procellaria westlandica, Punakaiki
Breeding season plastic exposure score = 23.48

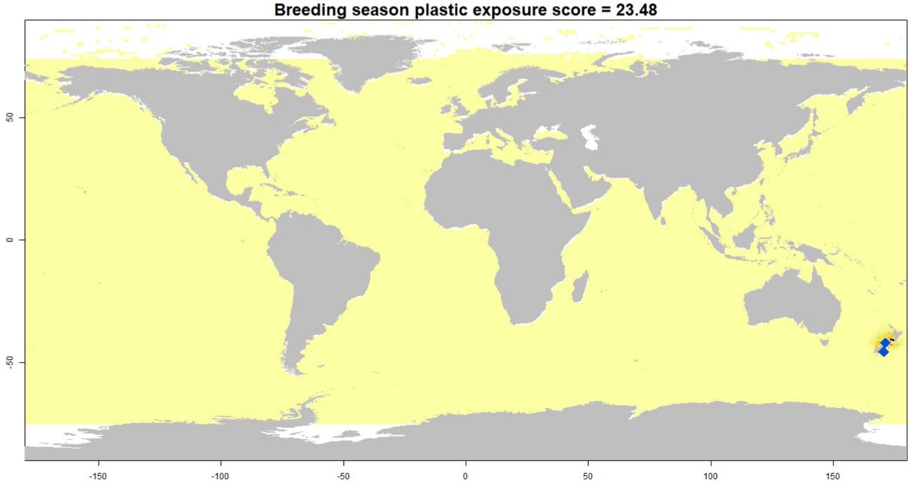

Trindade Petrel Pterodroma arminjoniana, Round Island
Breeding season plastic exposure score = 131.02

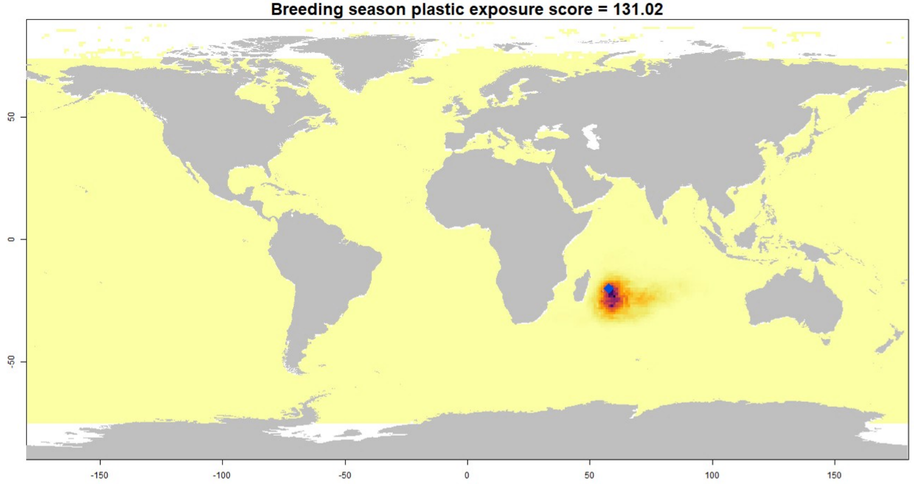

Trindade Petrel Pterodroma arminjoniana, Trindade Island
Breeding season plastic exposure score = 219.34

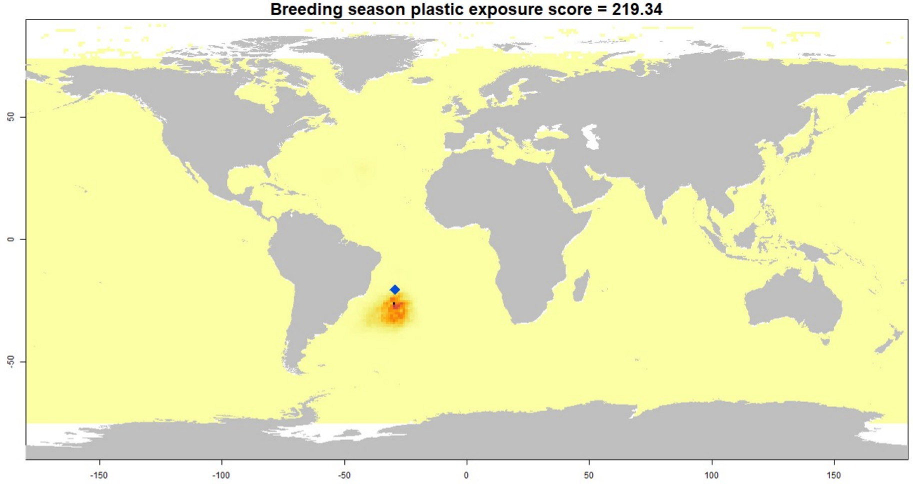

Tahiti Petrel Pseudobulweria rostrata, New Calendonia
Breeding season plastic exposure score = 10.61

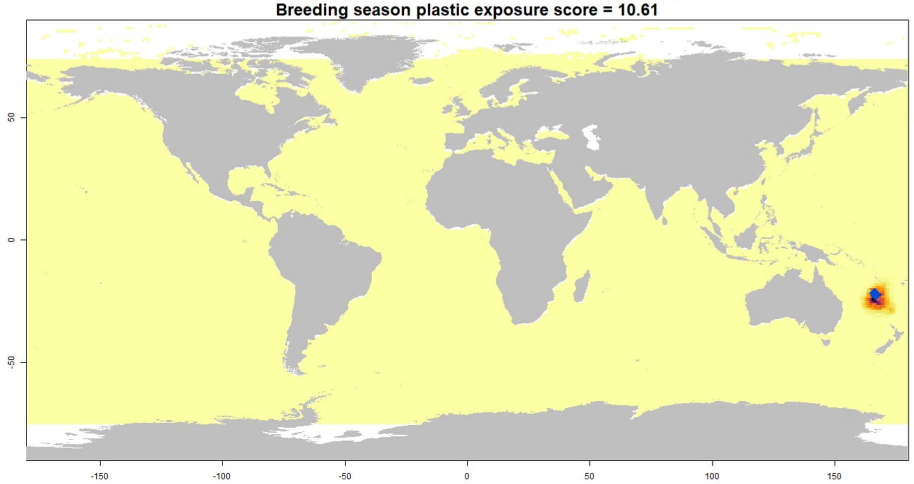

Trindade Petrel Pterodroma arminjoniana, Round Island
Non-breeding season plastic exposure score = 131.02

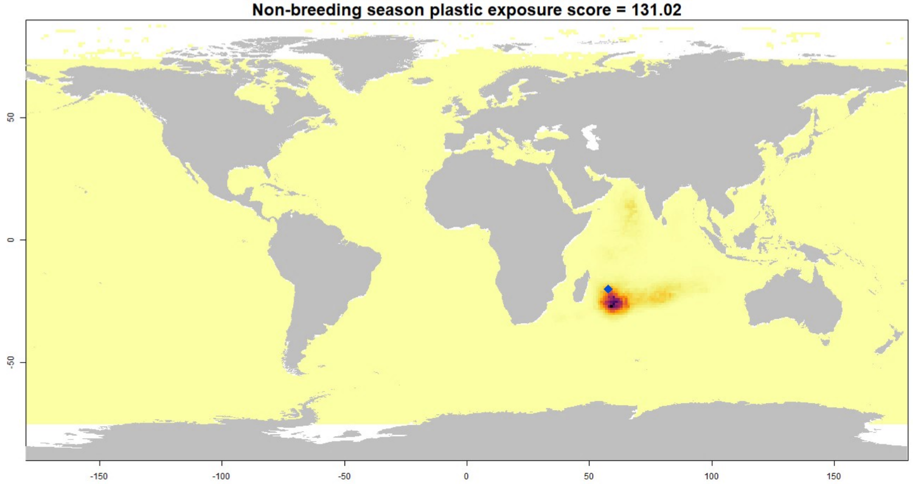

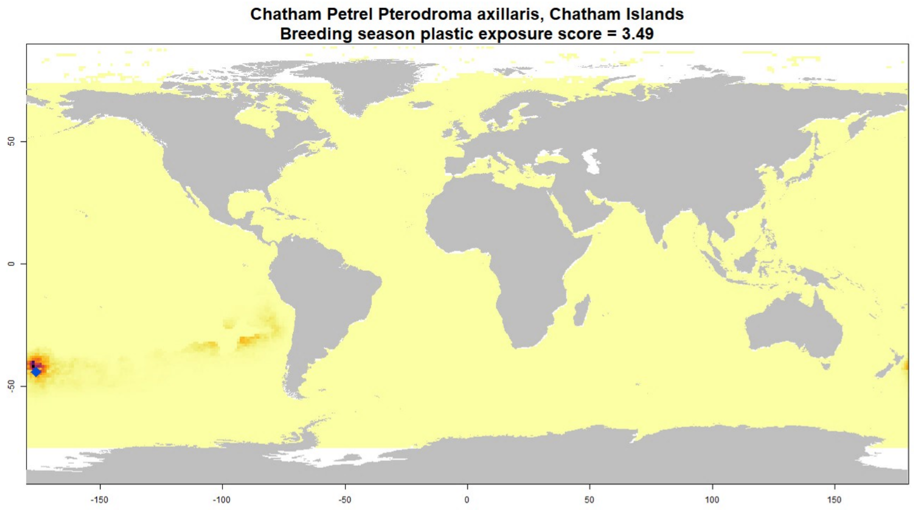
Chatham Petrel Pterodroma axillaris, Chatham Islands
Breeding season plastic exposure score = 3.49
50
0
-50
-150
-100
-50
0
50
100
150

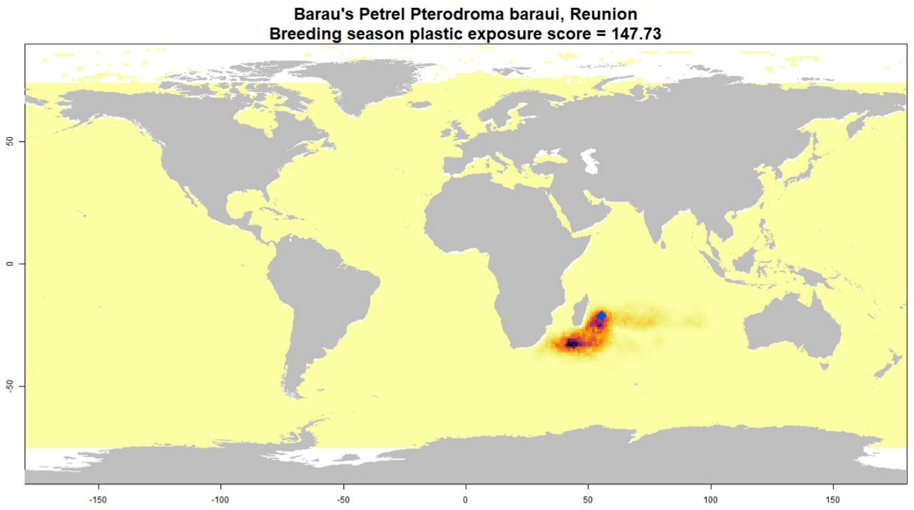
Barau's Petrel Pterodroma baraui, Reunion
Breeding season plastic exposure score = 147.73
50
0
-50
-150
-100
-50
0
50
100
150

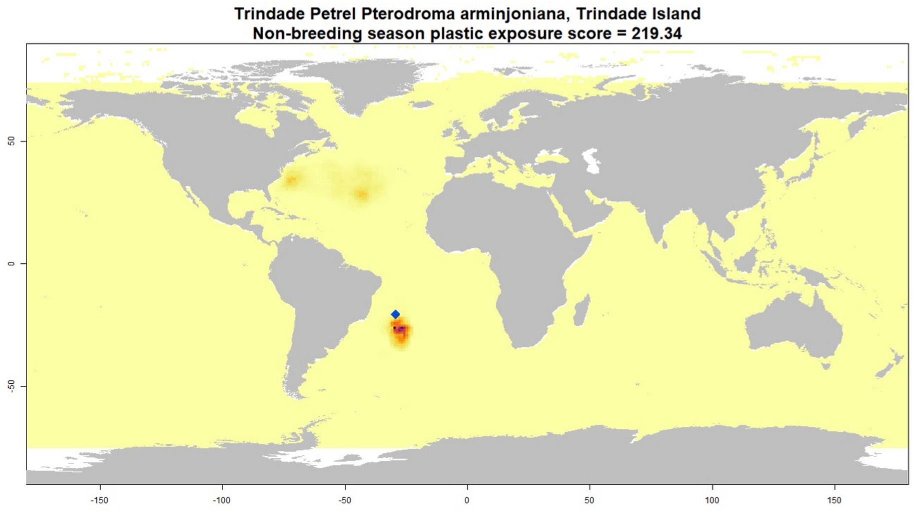
Trindade Petrel Pterodroma arminjoniana, Trindade Island
Non-breeding season plastic exposure score = 219.34
50
0
-50
-150
-100
-50
0
50
100
150

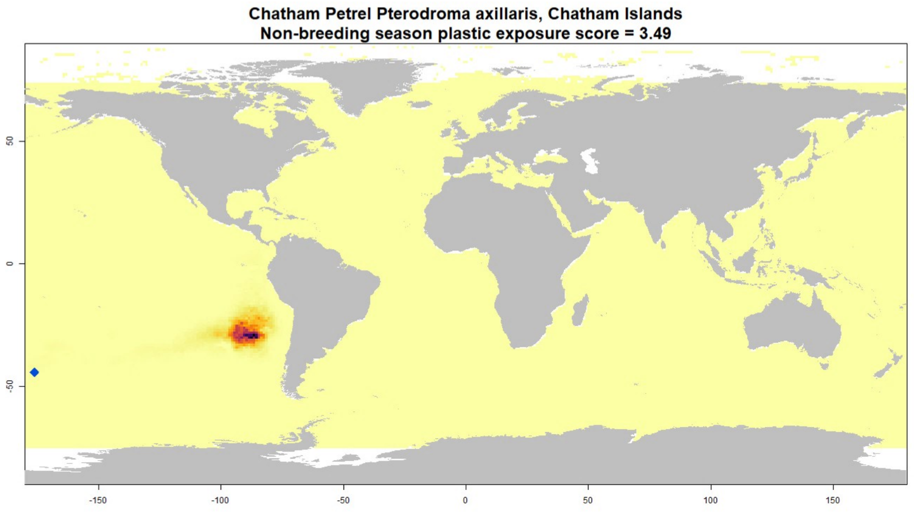
Chatham Petrel Pterodroma axillaris, Chatham Islands
Non-breeding season plastic exposure score = 3.49
50
0
-50
-150
-100
-50
0
50
100
150

Bermuda Petrel Pterodroma cahow, Bermuda Island
Breeding season plastic exposure score = 81.47

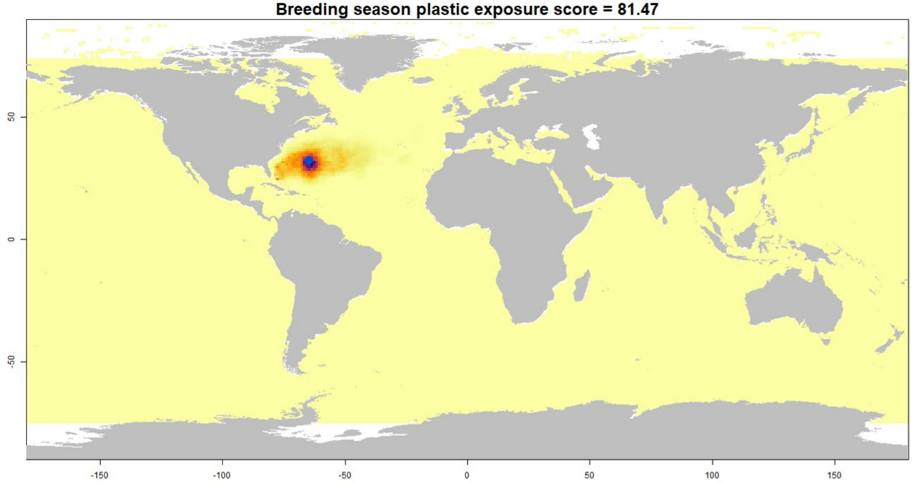

White-necked Petrel Pterodroma cervicalis, Phillip Island
Breeding season plastic exposure score = 14.97

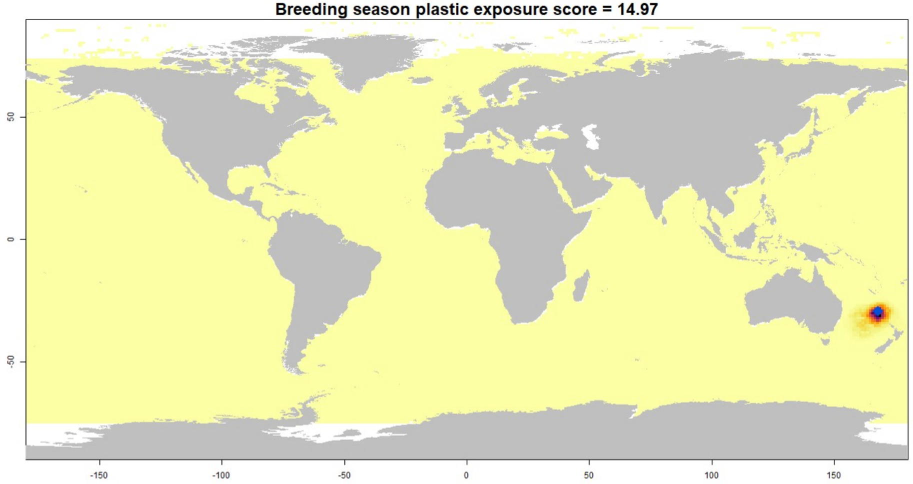

Barau's Petrel Pterodroma baraui, Reunion
Non-breeding season plastic exposure score = 147.73

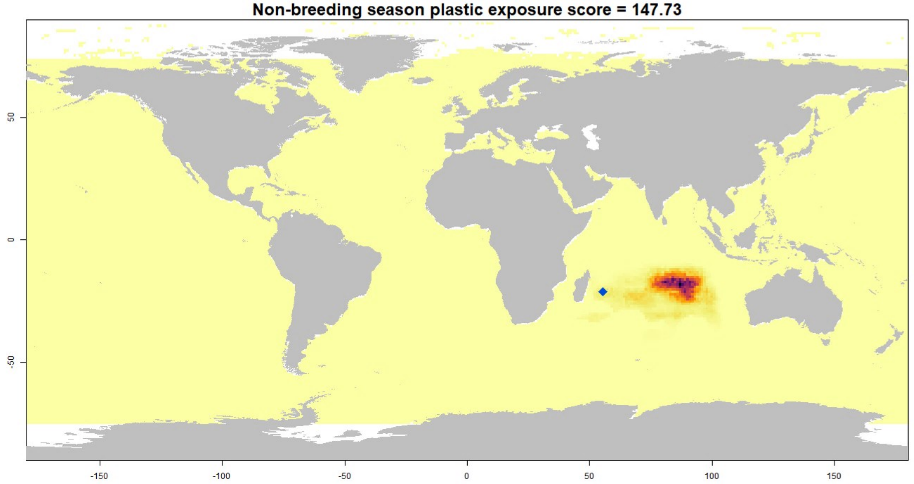

Bermuda Petrel Pterodroma cahow, Bermuda Island
Non-breeding season plastic exposure score = 81.47

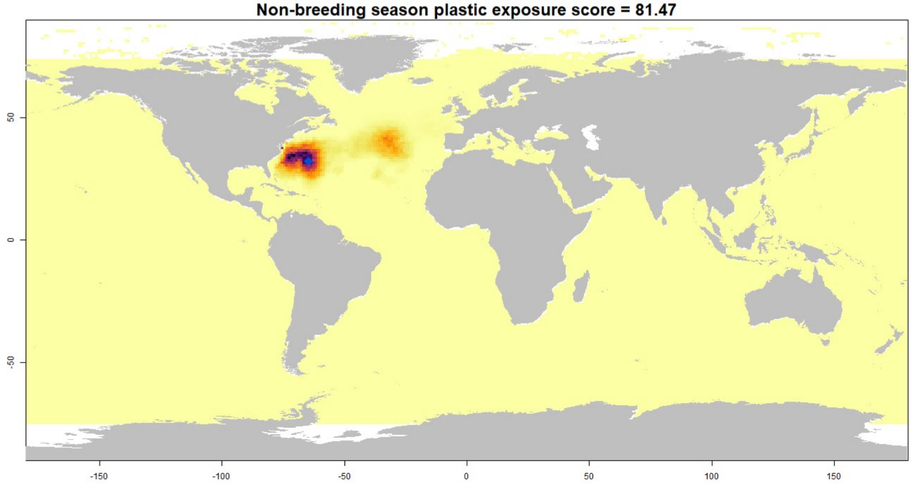

Cook's Petrel Pterodroma cookii, Codfish Island
Non-breeding season plastic exposure score = 12.27

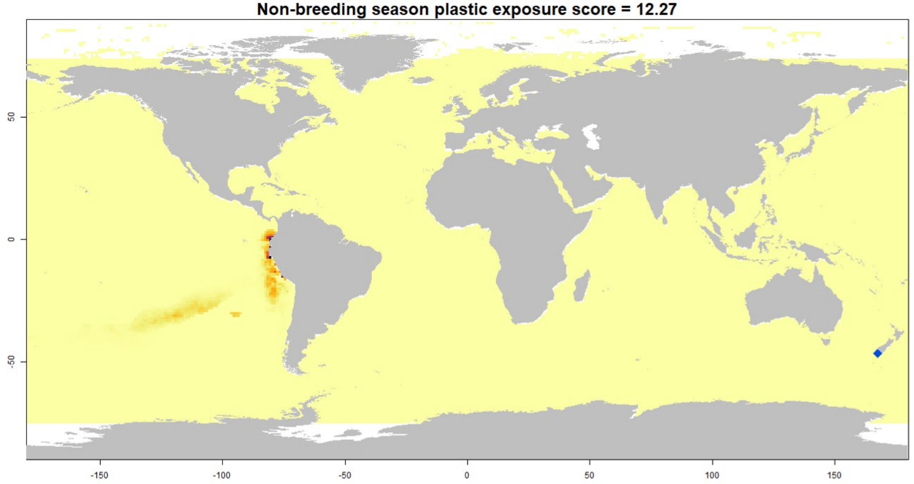

Cook's Petrel Pterodroma cookii, Little Barrier Island
Non-breeding season plastic exposure score = 33.19

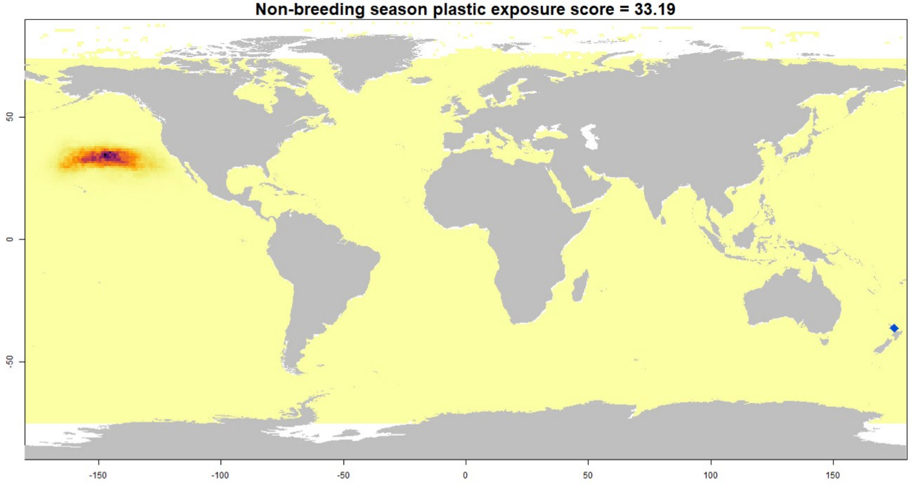

Cook's Petrel Pterodroma cookii, Codfish Island
Breeding season plastic exposure score = 12.27

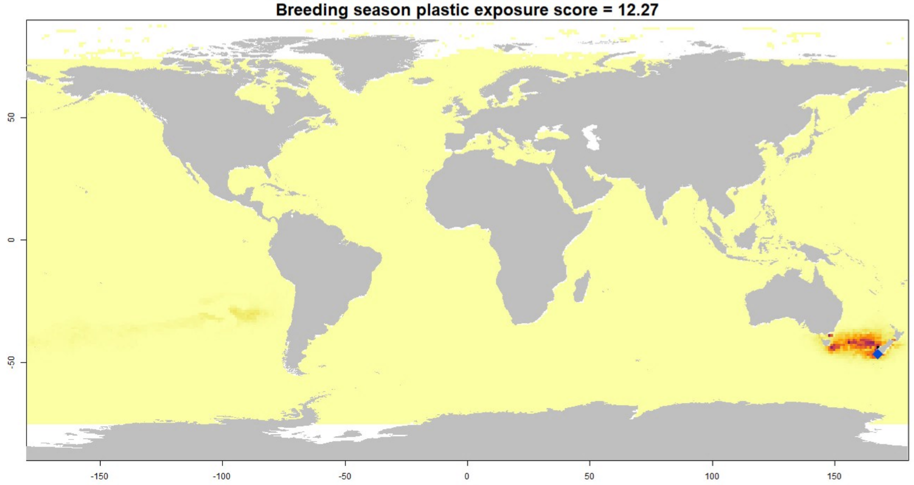

Cook's Petrel Pterodroma cookii, Little Barrier Island
Breeding season plastic exposure score = 33.19

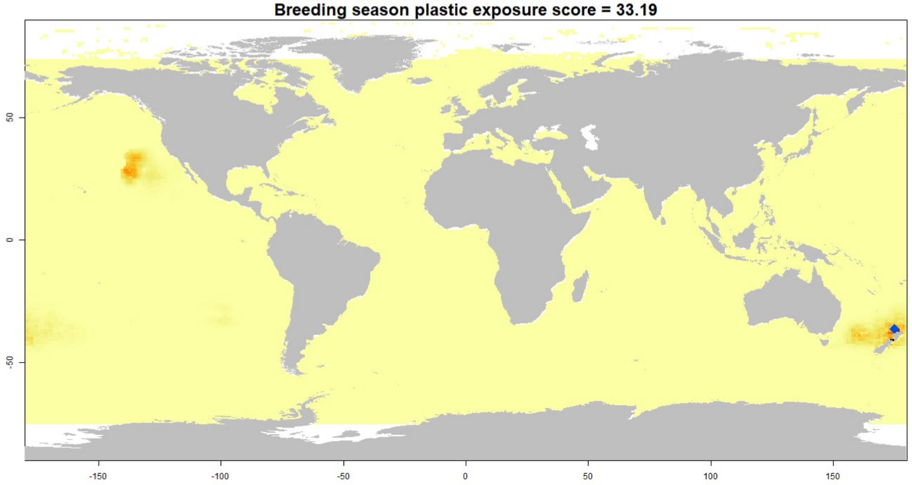

Desertas Petrel Pterodroma deserta, Madeira
Non-breeding season plastic exposure score = 36.51

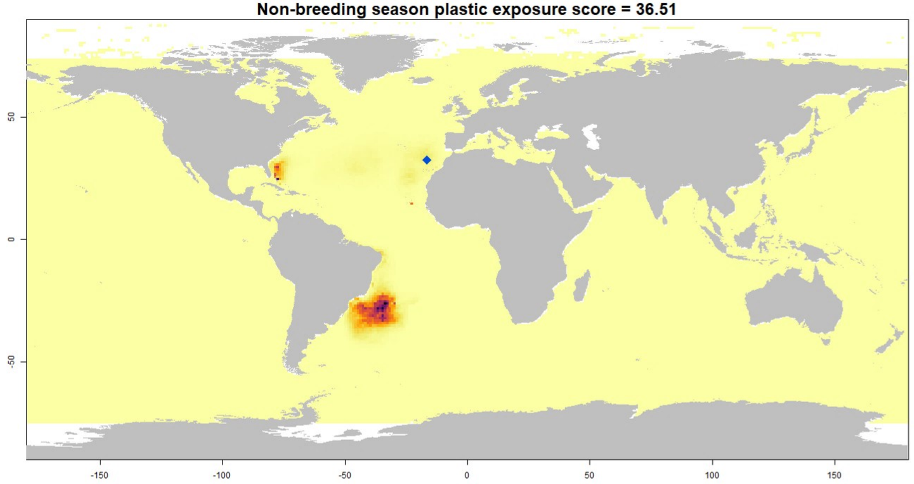

Cape Verde Petrel Pterodroma feae, Cape Verde
Non-breeding season plastic exposure score = 9.1

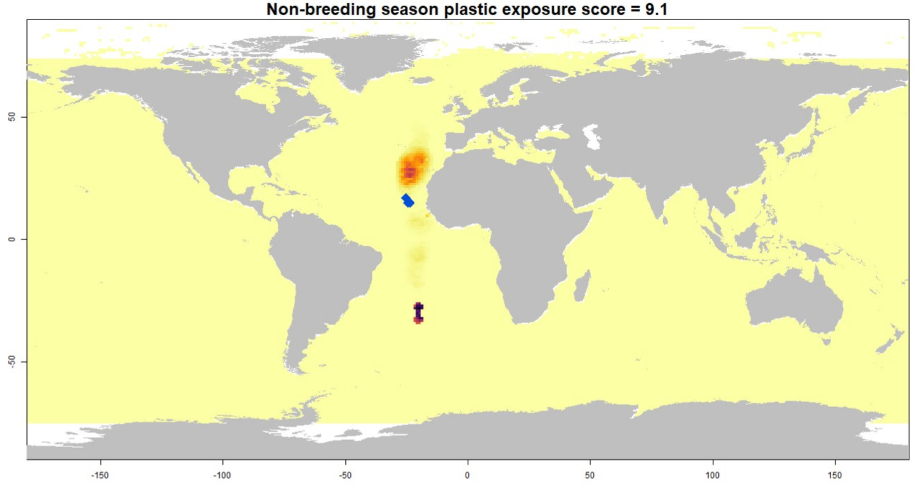

Desertas Petrel Pterodroma deserta, Madeira
Breeding season plastic exposure score = 36.51

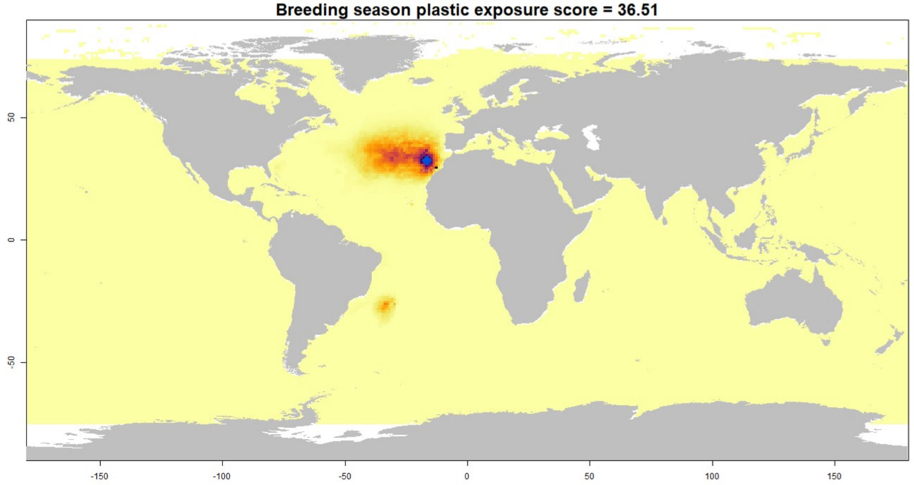

Cape Verde Petrel Pterodroma feae, Cape Verde
Breeding season plastic exposure score = 9.1

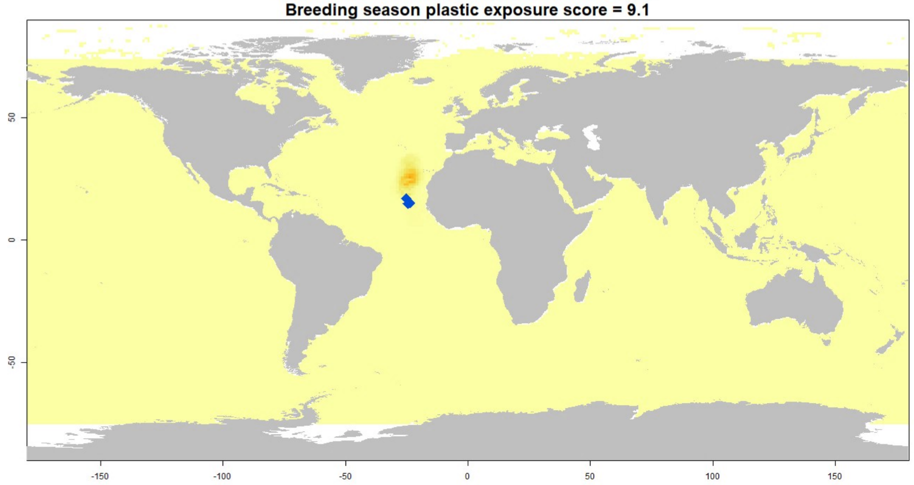

Black-capped Petrel Pterodroma hasitata, Sierra de Bahoruco
Breeding season plastic exposure score = 27.3

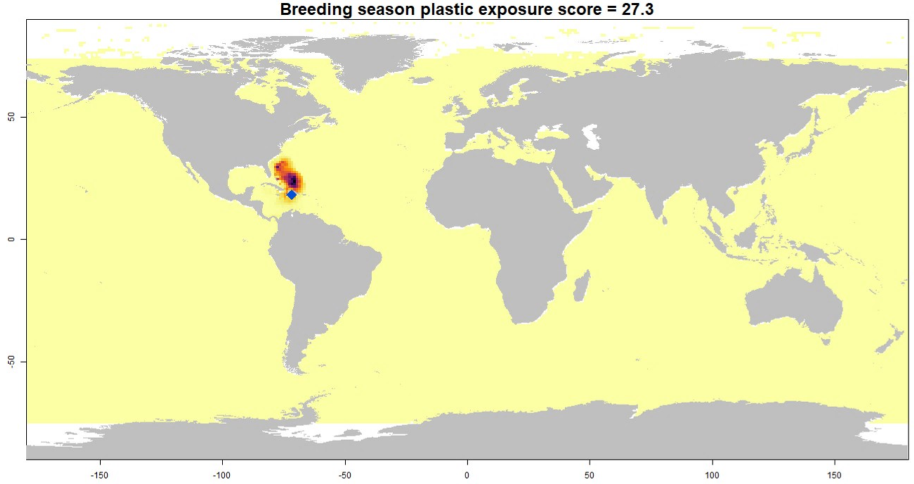

Atlantic Petrel Pterodroma incerta, Gough
Breeding season plastic exposure score = 123.12

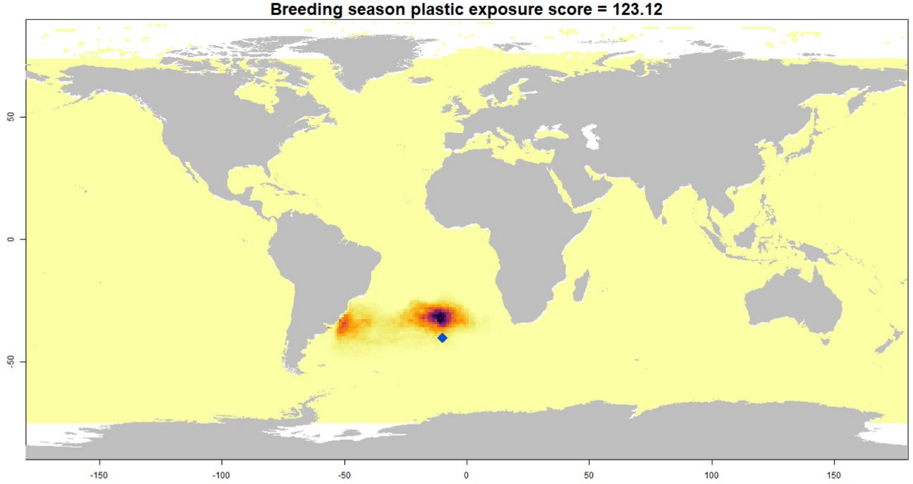

Grey-faced Petrel Pterodroma gouldi, Bethells Beach
Breeding season plastic exposure score = 11.91

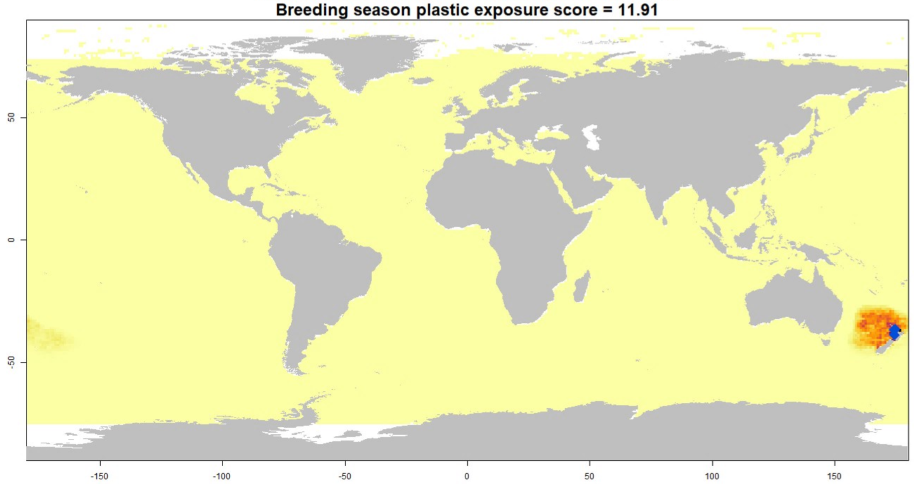

Black-capped Petrel Pterodroma hasitata, Sierra de Bahoruco
Non-breeding season plastic exposure score = 27.3

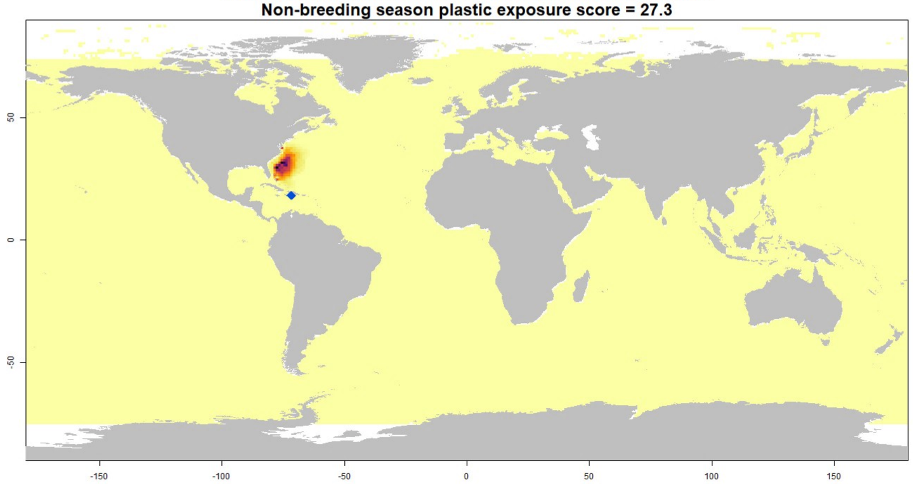

Mottled Petrel Pterodroma inexpectata, Lake HaurokoPetrel Island
Breeding season plastic exposure score = 22.12

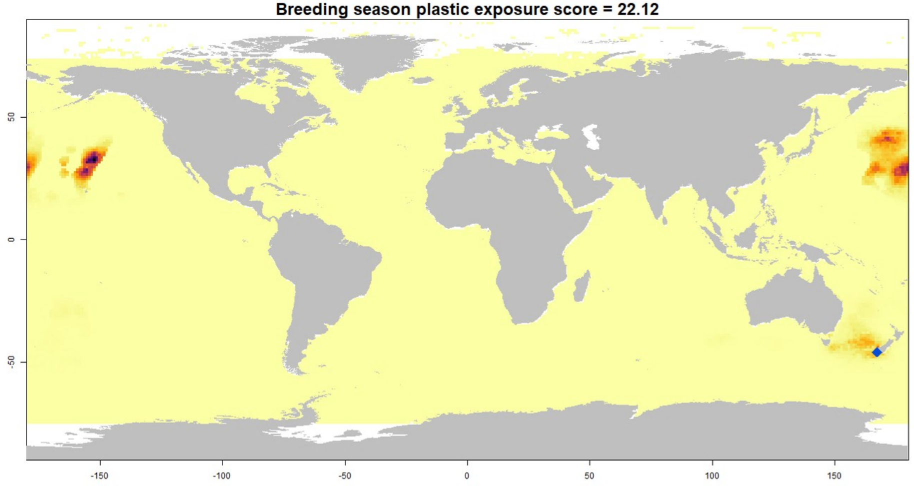

White-headed Petrel Pterodroma lessonii, Adams Island
Breeding season plastic exposure score = 1.37

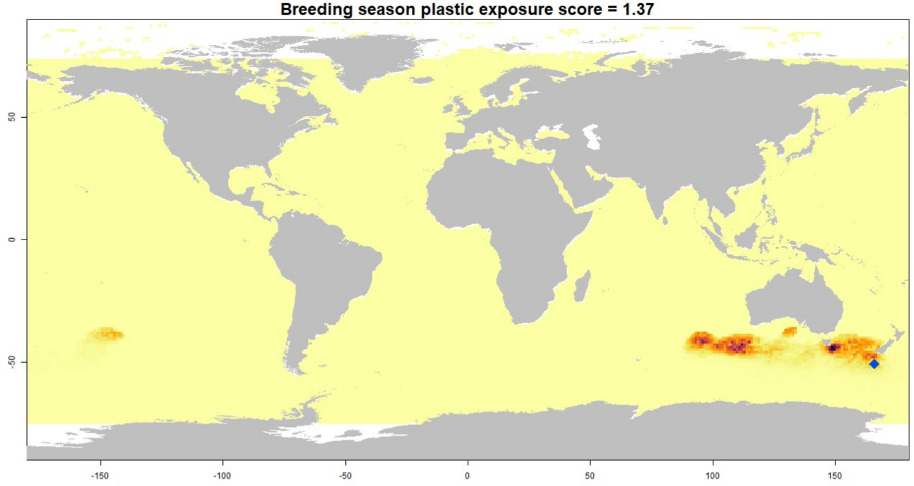

Atlantic Petrel Pterodroma incerta, Gough
Non-breeding season plastic exposure score = 123.12

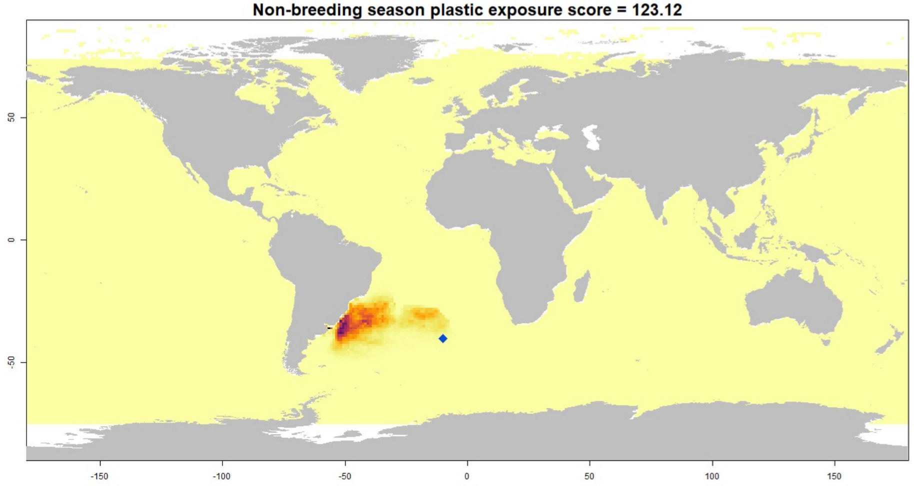

Mottled Petrel Pterodroma inexpectata, Lake HaurokoPetrel Island
Non-breeding season plastic exposure score = 22.12

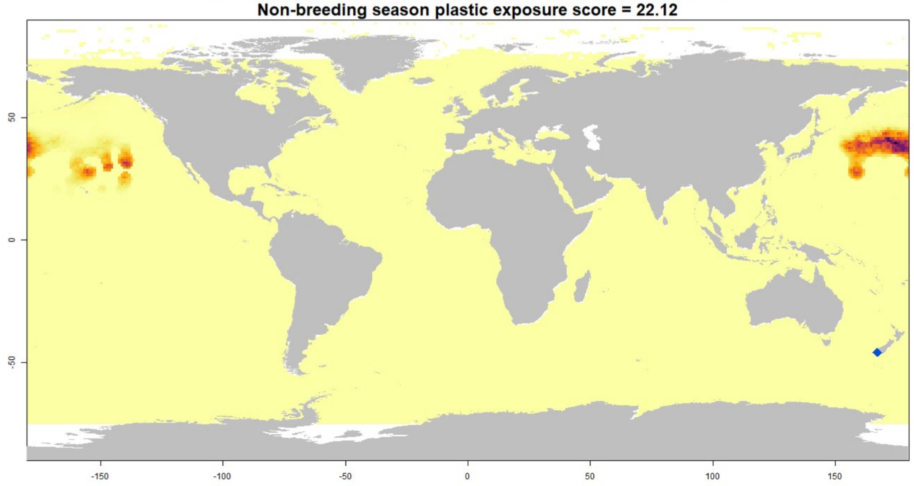

White-winged Petrel Pterodroma leucoptera, Cabbage Tree Island
Breeding season plastic exposure score = 27.37

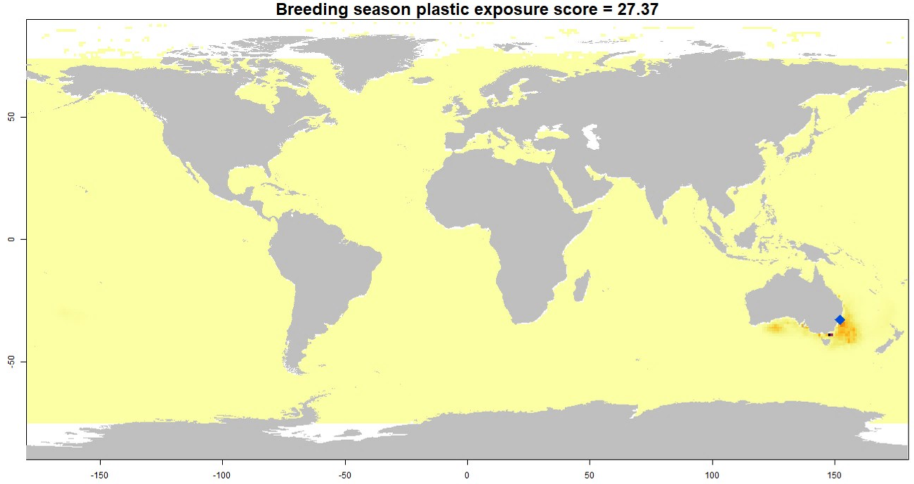

White-winged Petrel Pterodroma leucoptera, New Caledonia
Breeding season plastic exposure score = 21.37

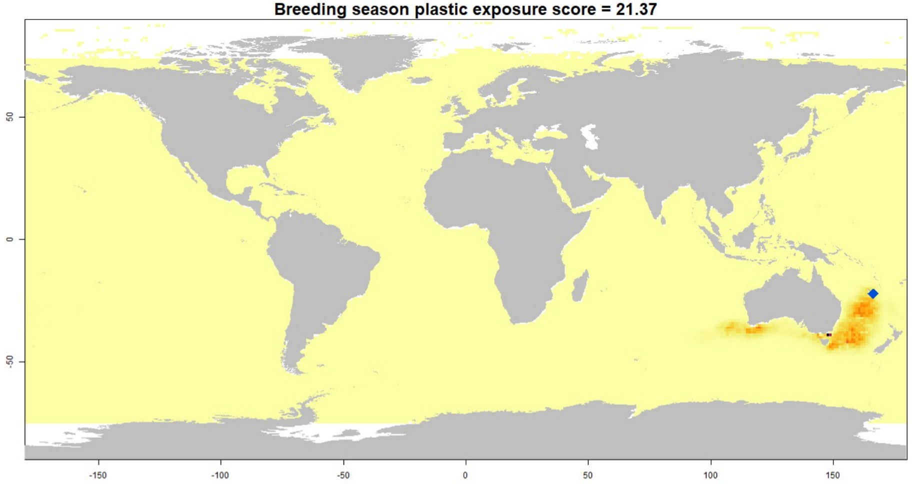

White-headed Petrel Pterodroma lessonii, Adams Island
Non-breeding season plastic exposure score = 1.37

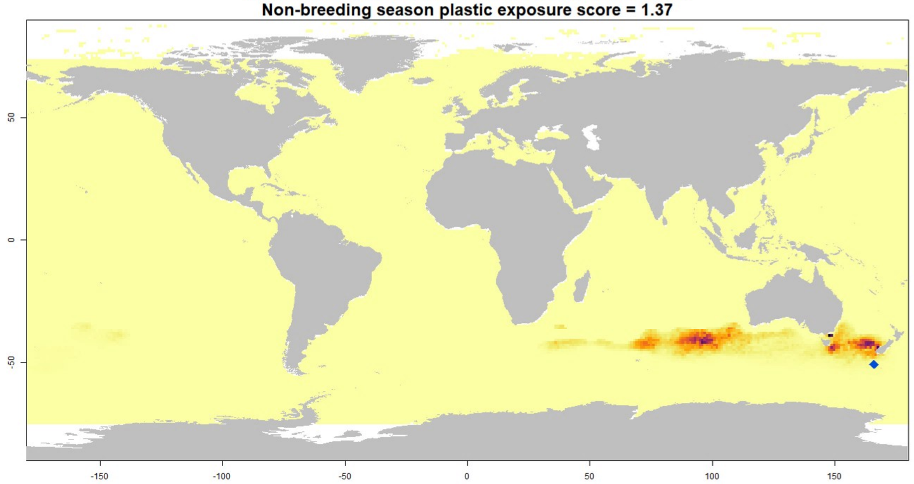

White-winged Petrel Pterodroma leucoptera, Cabbage Tree Island
Non-breeding season plastic exposure score = 27.37

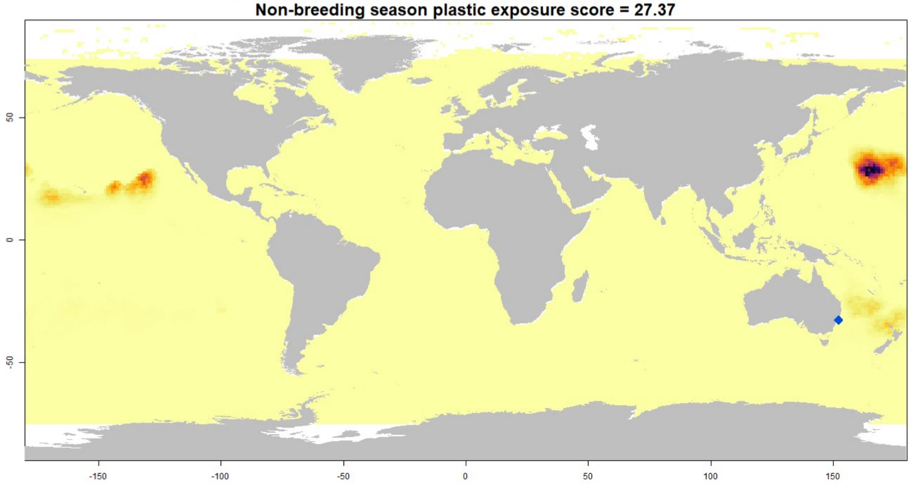

Great-winged Petrel Pterodroma macroptera, Prince Edward Islands
Breeding season plastic exposure score = 11.31

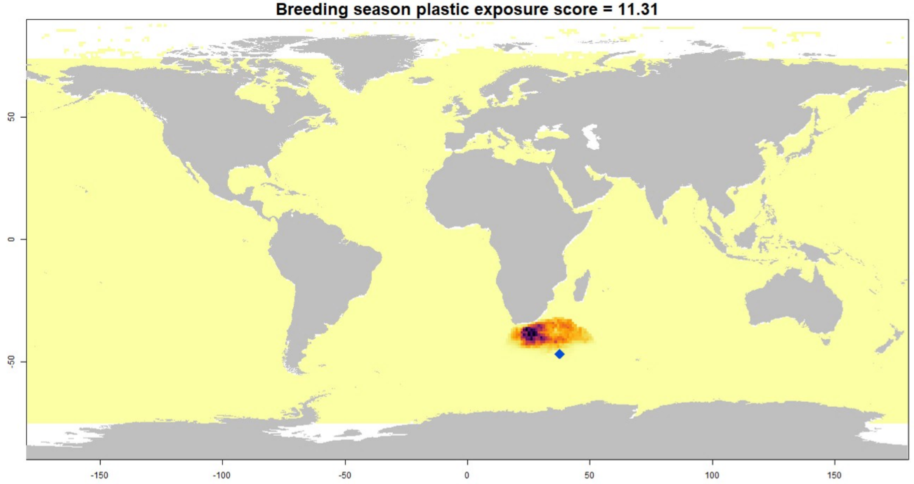

Zino's Petrel Pterodroma madeira, Madeira
Non-breeding season plastic exposure score = 34.34

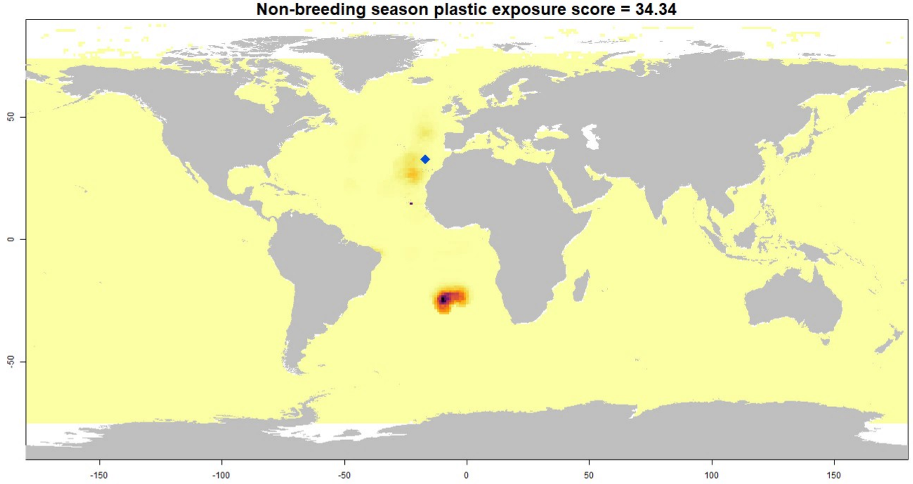

White-winged Petrel Pterodroma leucoptera, New Caledonia
Non-breeding season plastic exposure score = 21.37

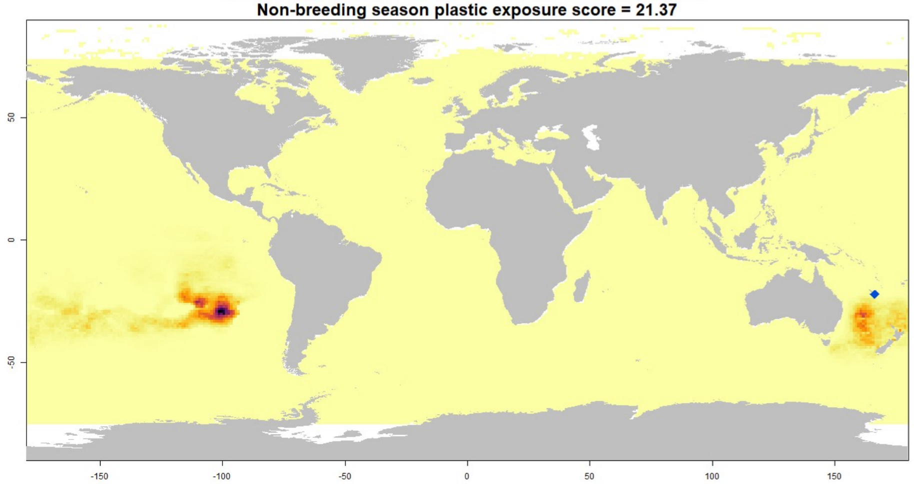

Zino's Petrel Pterodroma madeira, Madeira
Breeding season plastic exposure score = 34.34

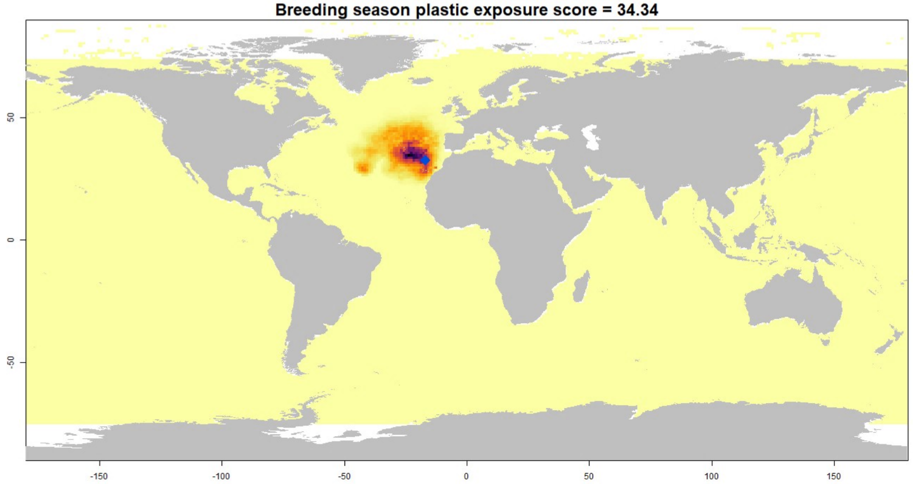

Magenta Petrel Pterodroma magentae, Chatham Islands
Non-breeding season plastic exposure score = 8.79

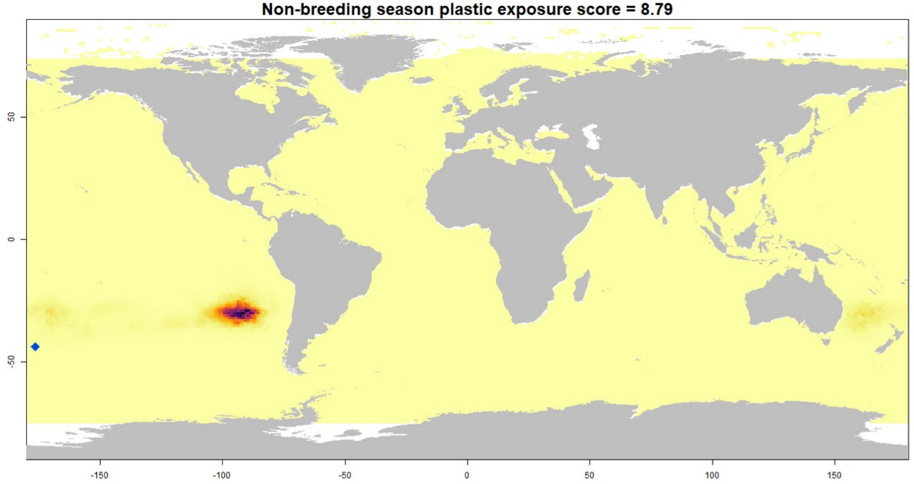

Soft-plumaged Petrel Pterodroma mollis, Antipodes
Non-breeding season plastic exposure score = 7.87

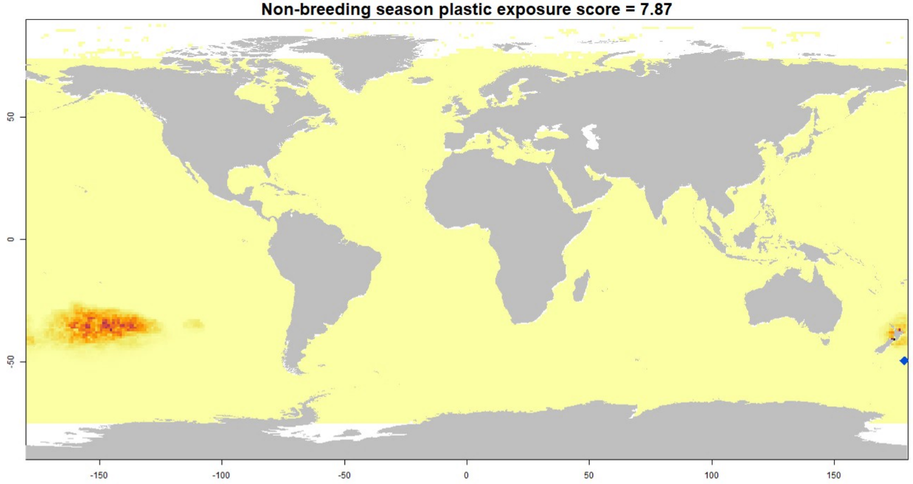

Magenta Petrel Pterodroma magentae, Chatham Islands
Breeding season plastic exposure score = 8.79

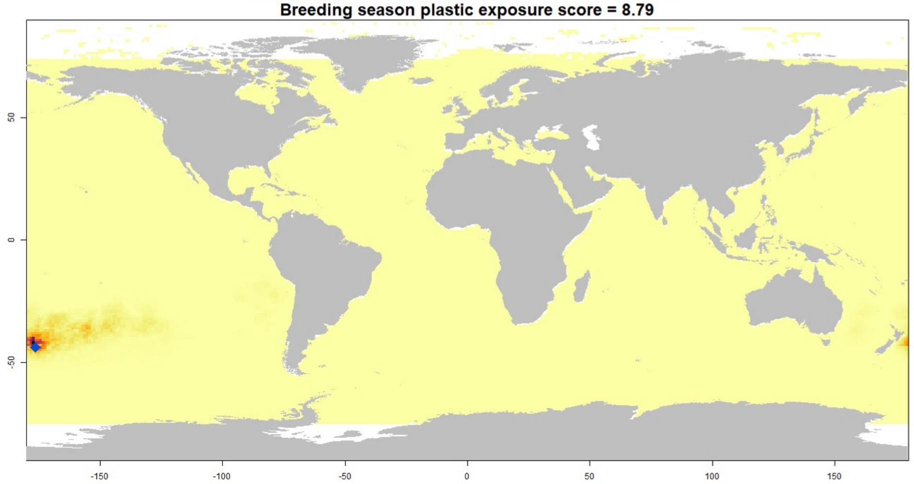

Soft-plumaged Petrel Pterodroma mollis, Antipodes
Breeding season plastic exposure score = 7.87

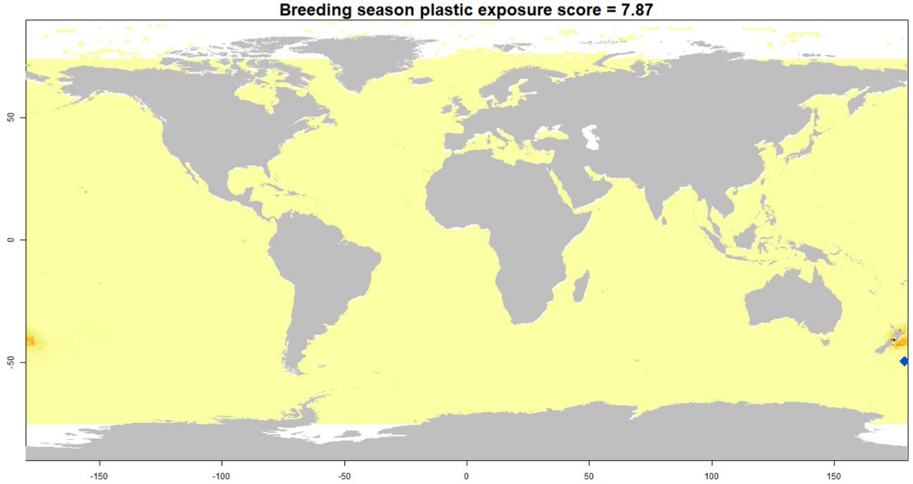

Soft-plumaged Petrel Pterodroma mollis, Gough
Non-breeding season plastic exposure score = 30.35

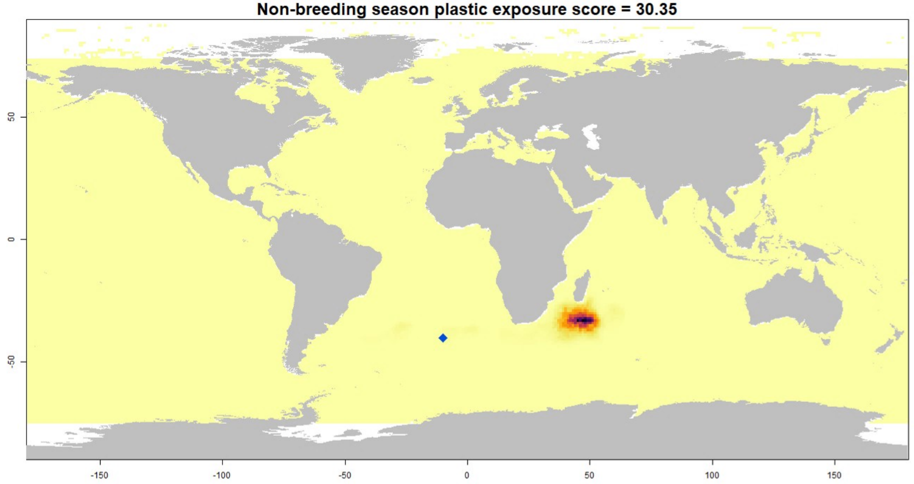

Black-winged Petrel Pterodroma nigripennis, Chatham Islands
Breeding season plastic exposure score = 34.61

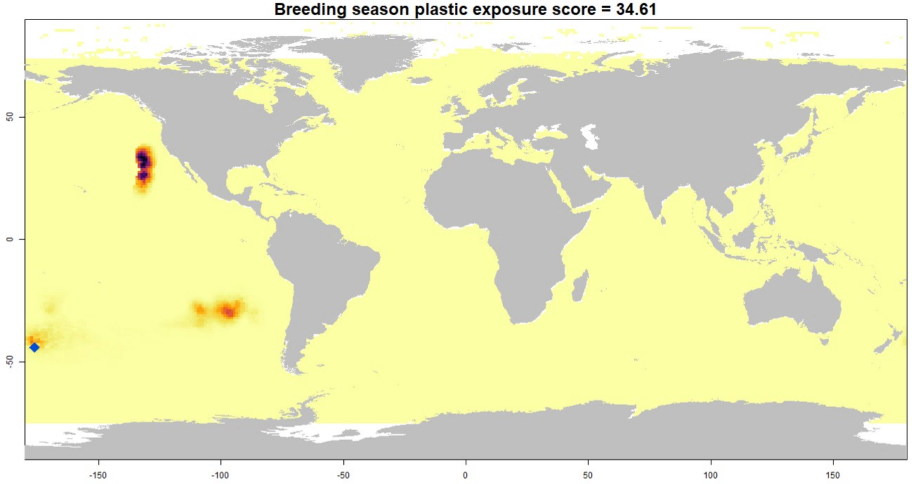

Soft-plumaged Petrel Pterodroma mollis, Gough
Breeding season plastic exposure score = 30.35

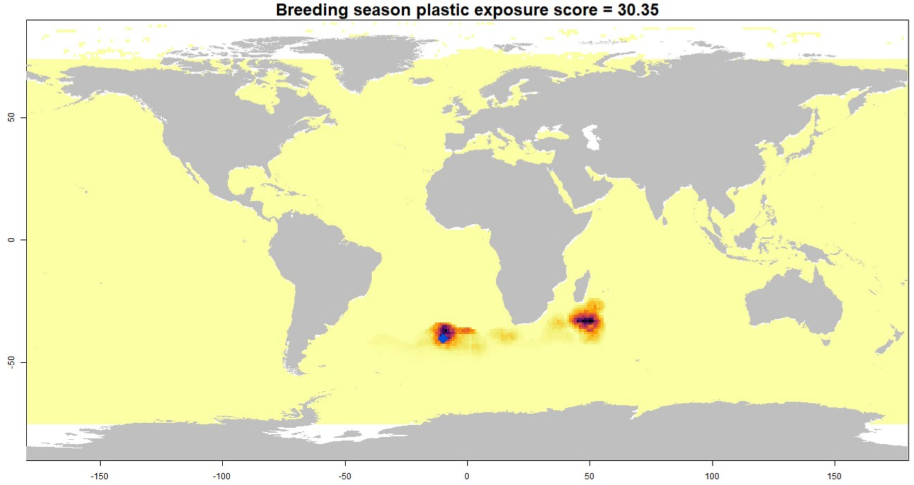

Kermadec Petrel Pterodroma neglecta, Phillip Island
Breeding season plastic exposure score = 4.96

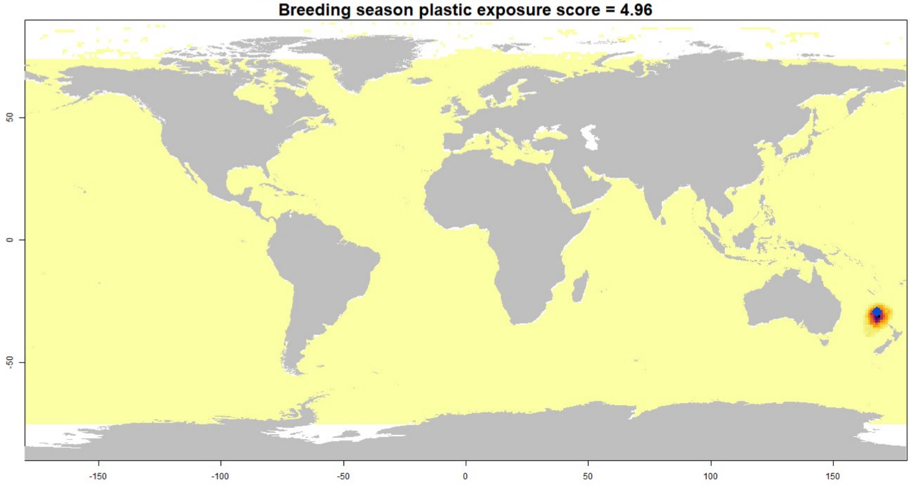

Black-winged Petrel Pterodroma nigripennis, Phillip Island
Breeding season plastic exposure score = 9.2

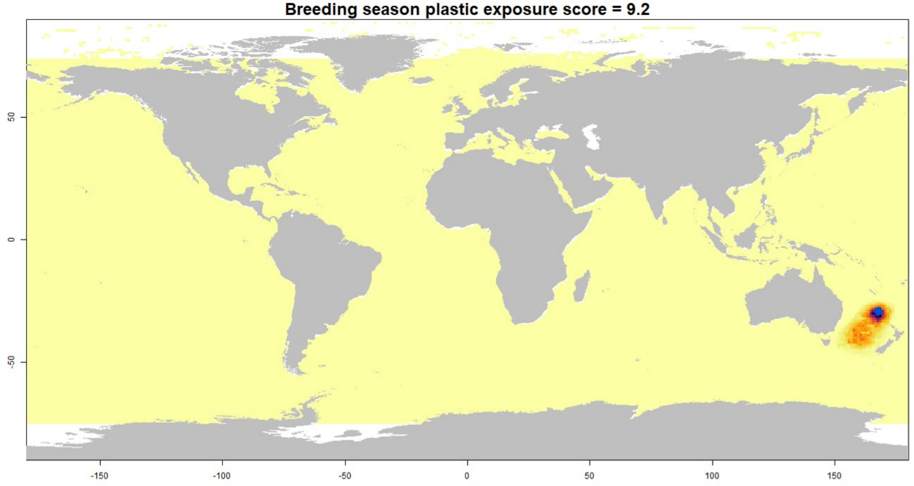

Pycroft's Petrel Pterodroma pycrofti, Red Mercury Island
Breeding season plastic exposure score = 18.03

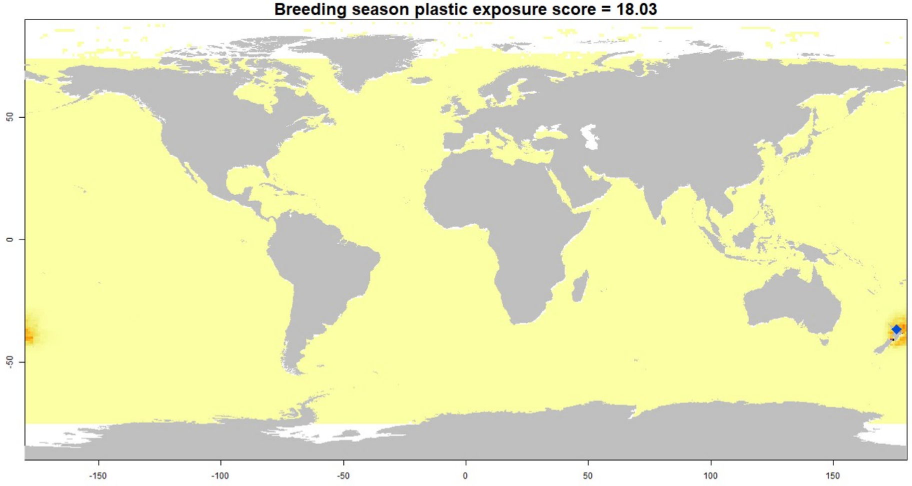

Black-winged Petrel Pterodroma nigripennis, Chatham Islands
Non-breeding season plastic exposure score = 34.61

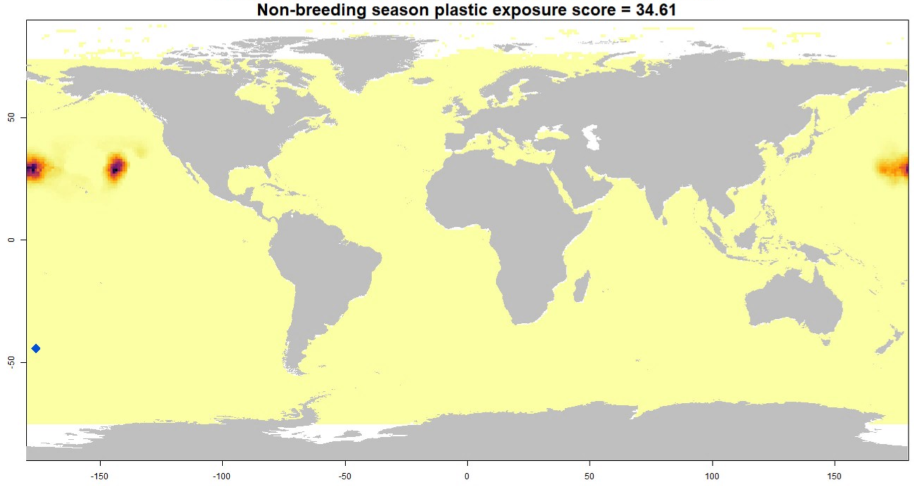

Galapagos Petrel Pterodroma phaeopygia, Galapagos
Breeding season plastic exposure score = 2.01

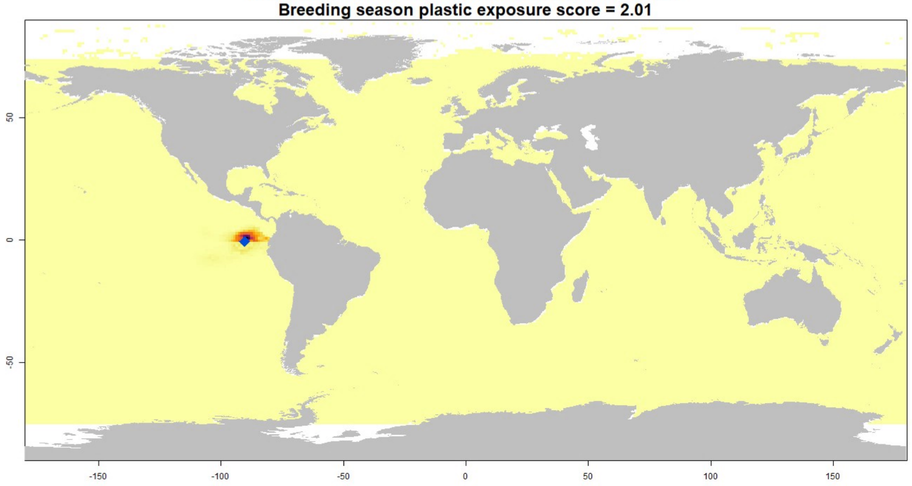

Hawaiian Petrel Pterodroma sandwichensis, Hawaii
Breeding season plastic exposure score = 507.86

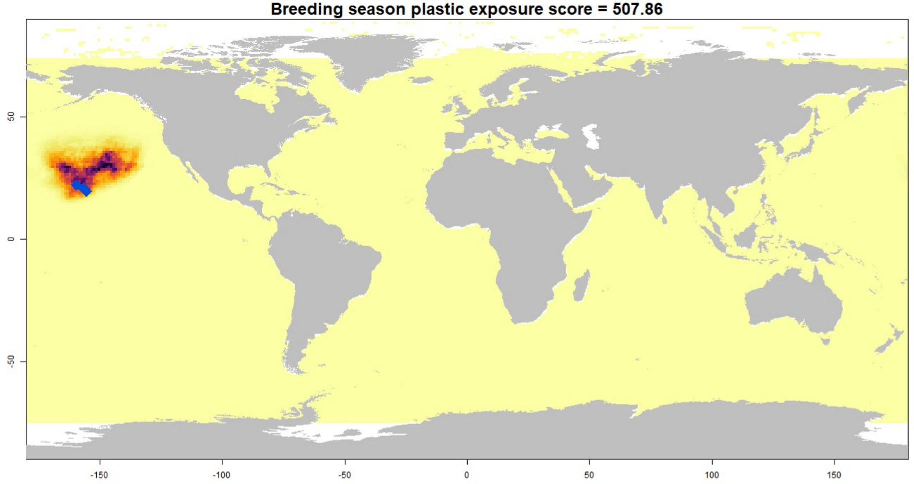

Providence Petrel Pterodroma solandri, Lord Howe Island
Non-breeding season plastic exposure score = 16.23

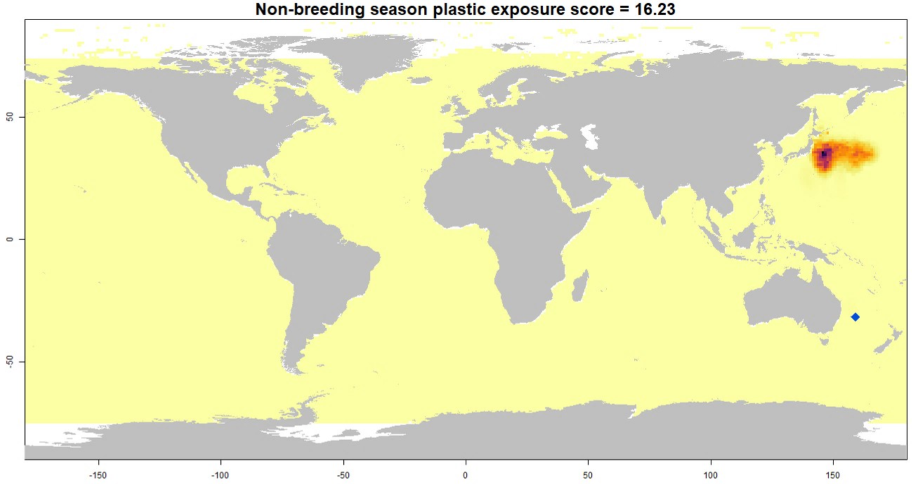

Pycroft's Petrel Pterodroma pycrofti, Red Mercury Island
Non-breeding season plastic exposure score = 18.03

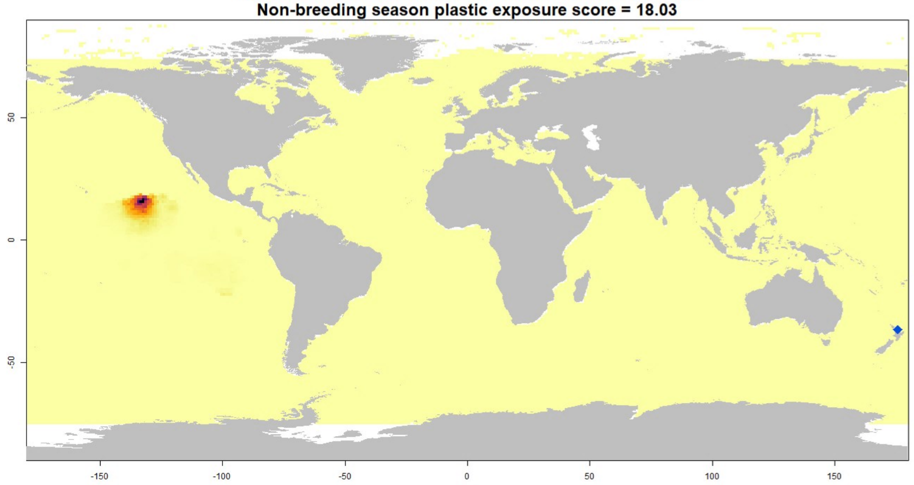

Providence Petrel Pterodroma solandri, Lord Howe Island
Breeding season plastic exposure score = 16.23

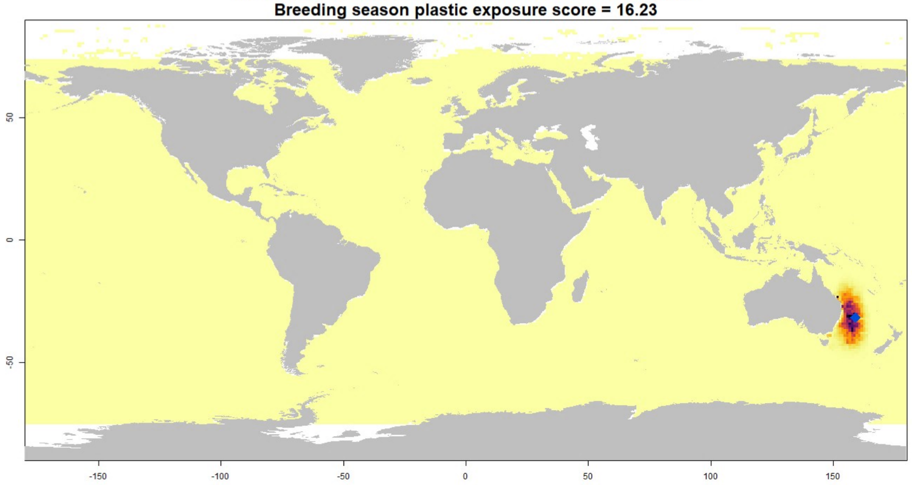

Murphy's Petrel Pterodroma ultima, Henderson Island
Non-breeding season plastic exposure score = 61.48

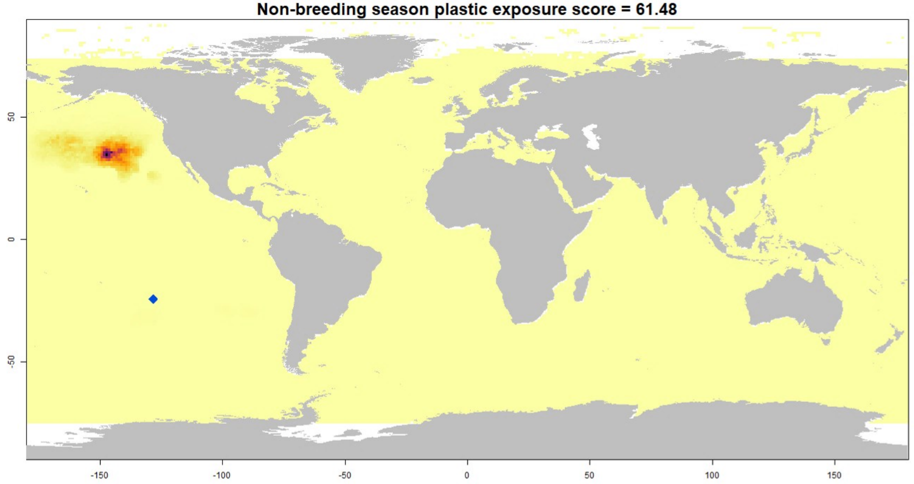

Little Shearwater Puffinus assimilis, E of Aukland
Non-breeding season plastic exposure score = 30.77

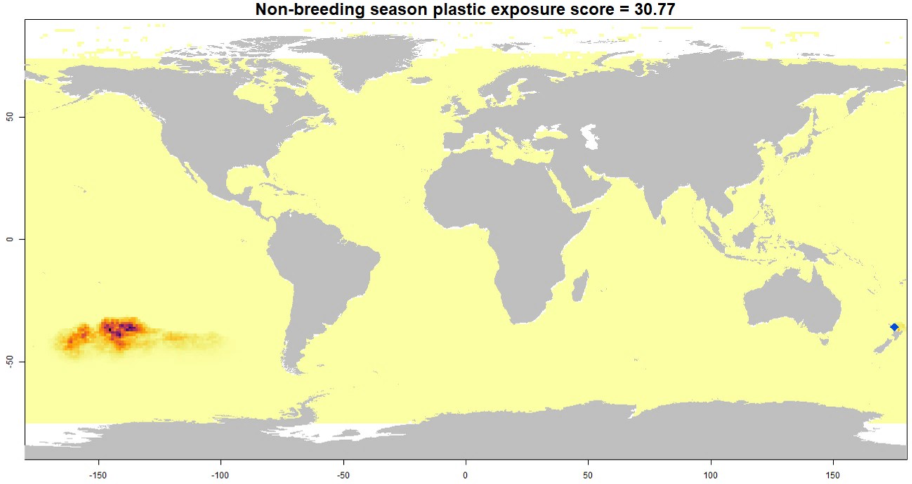

Murphy's Petrel Pterodroma ultima, Henderson Island
Breeding season plastic exposure score = 61.48

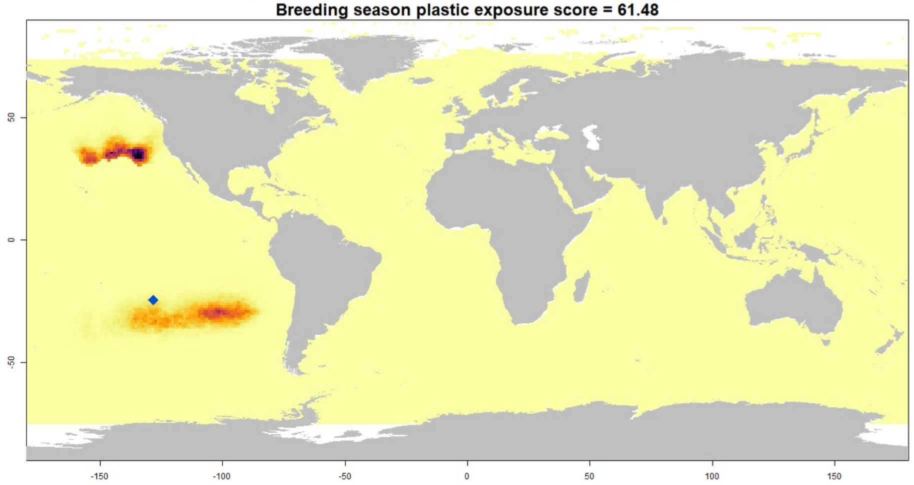

Little Shearwater Puffinus assimilis, E of Aukland
Breeding season plastic exposure score = 30.77

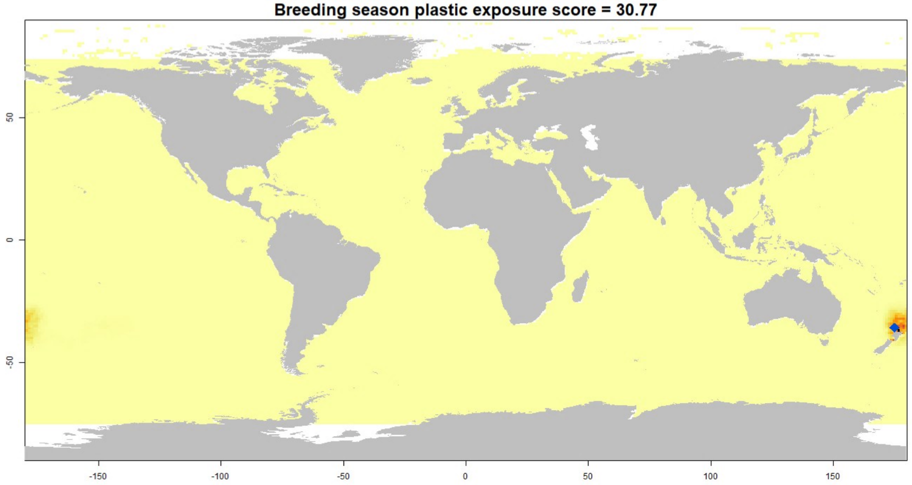

Little Shearwater Puffinus assimilis, Lord Howe Island
Non-breeding season plastic exposure score = 23.41

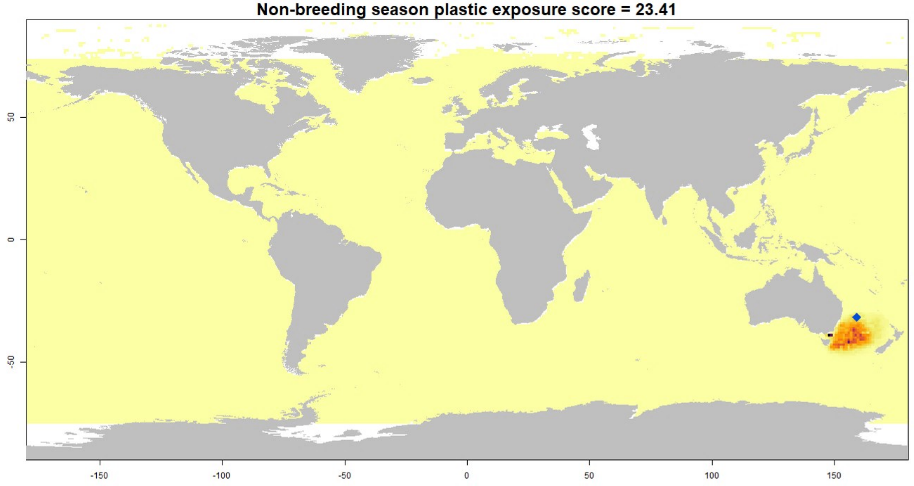

Fluttering Shearwater Puffinus gavia, E of Aukland
Non-breeding season plastic exposure score = 39.88

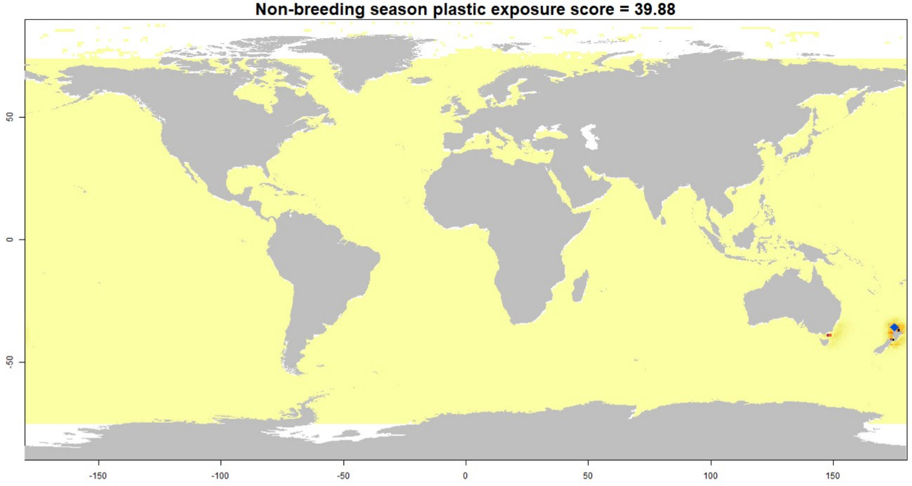

Little Shearwater Puffinus assimilis, Lord Howe Island
Breeding season plastic exposure score = 23.41

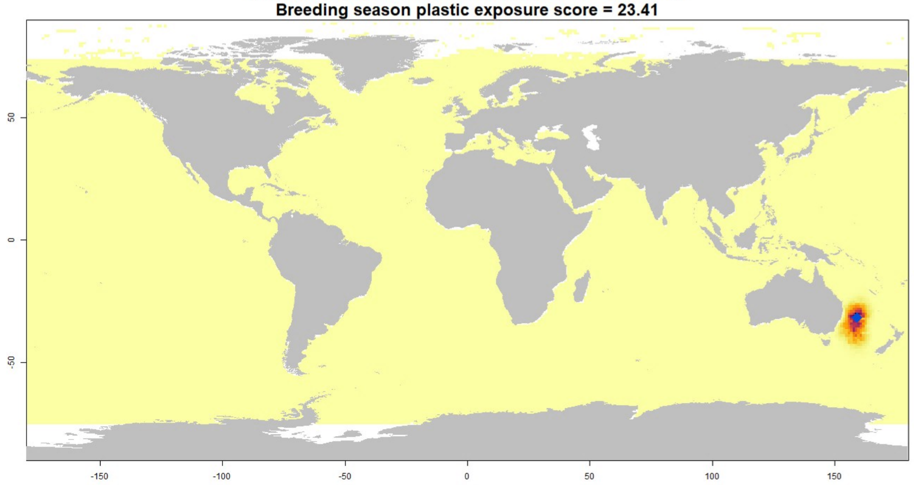

Fluttering Shearwater Puffinus gavia, E of Aukland
Breeding season plastic exposure score = 39.88

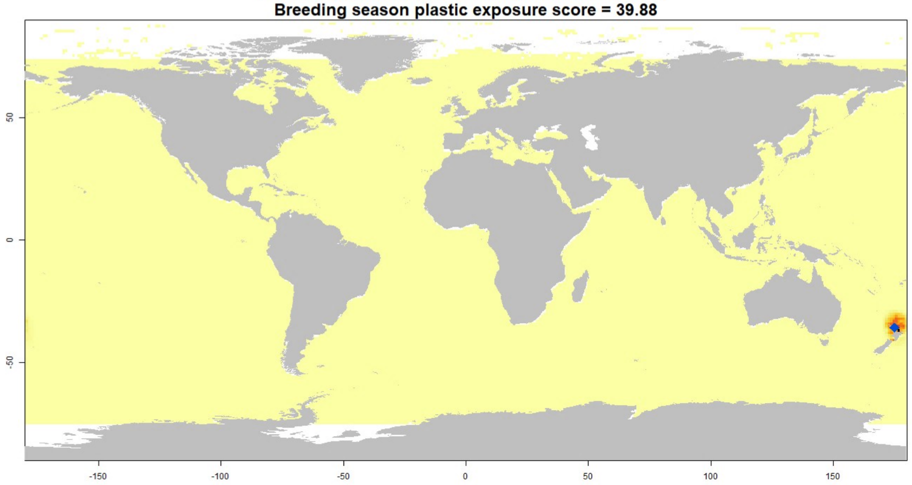

Audubon's Shearwater Puffinus lherminieri, Azores
Breeding season plastic exposure score = 54.19

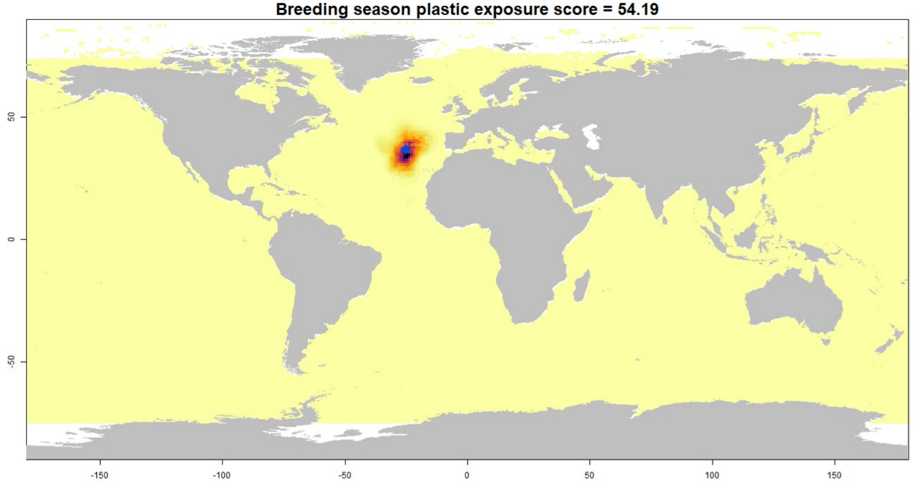

Audubon's Shearwater Puffinus lherminieri, Cape Verde
Breeding season plastic exposure score = 6.61

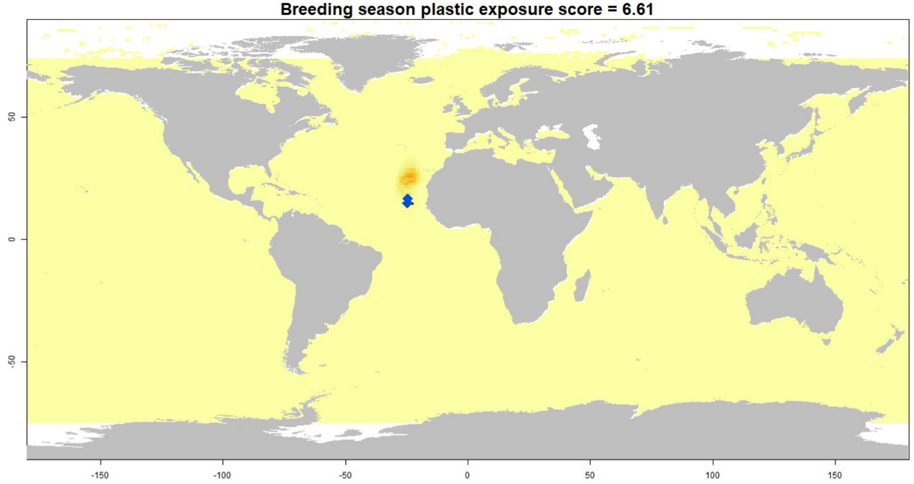

Hutton's Shearwater Puffinus huttoni, Te Rae o Atiu
Breeding season plastic exposure score = 6.16

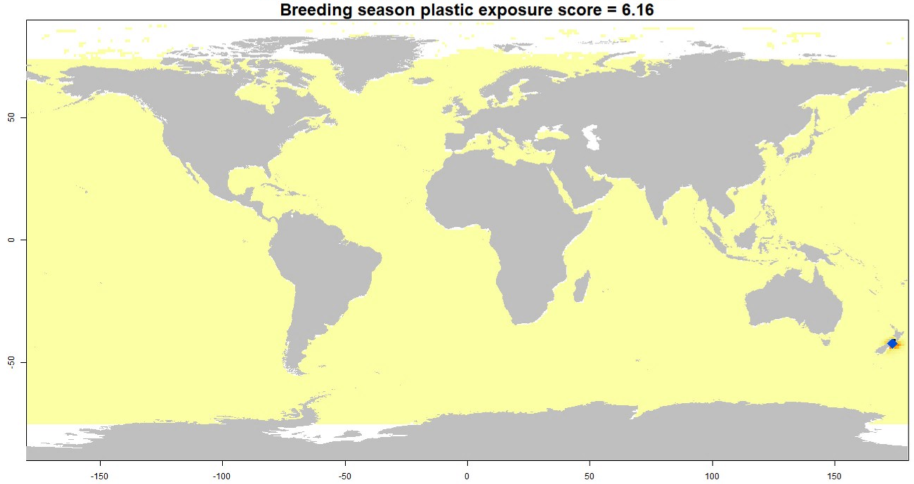

Audubon's Shearwater Puffinus lherminieri, Azores
Non-breeding season plastic exposure score = 54.19

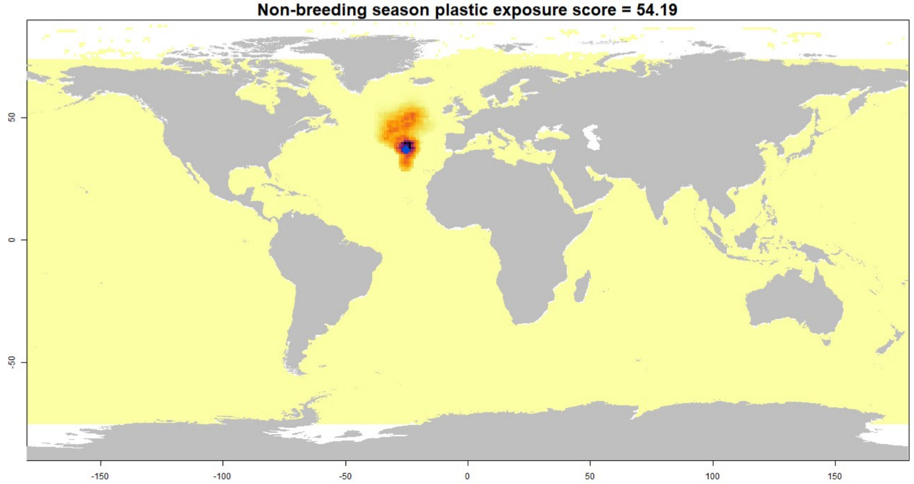

Audubon's Shearwater Puffinus lherminieri, Madeira
Breeding season plastic exposure score = 30.06

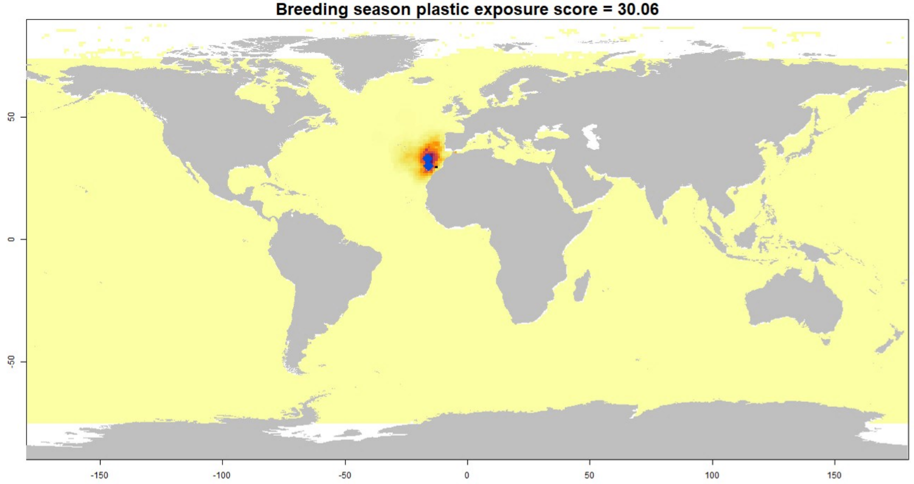

Balearic Shearwater Puffinus mauretanicus, Balearic Archipelago
Breeding season plastic exposure score = 2043.6

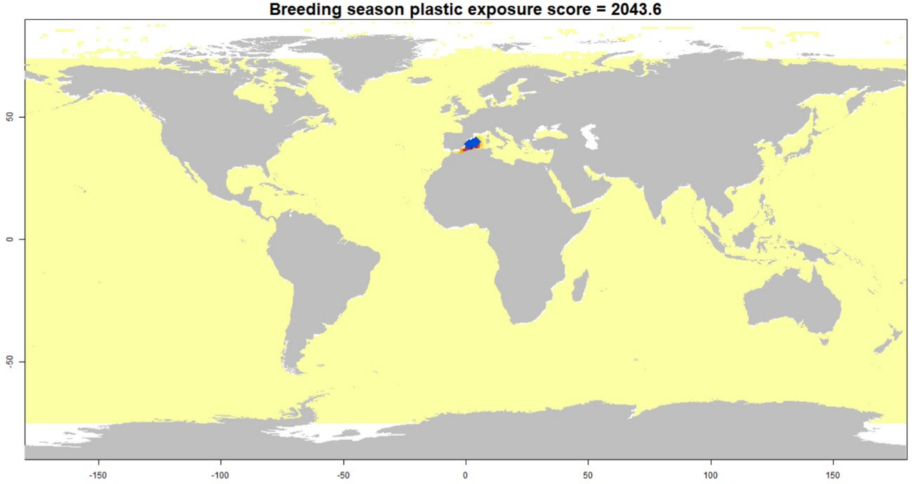

Audubon's Shearwater Puffinus lherminieri, Cape Verde
Non-breeding season plastic exposure score = 6.61

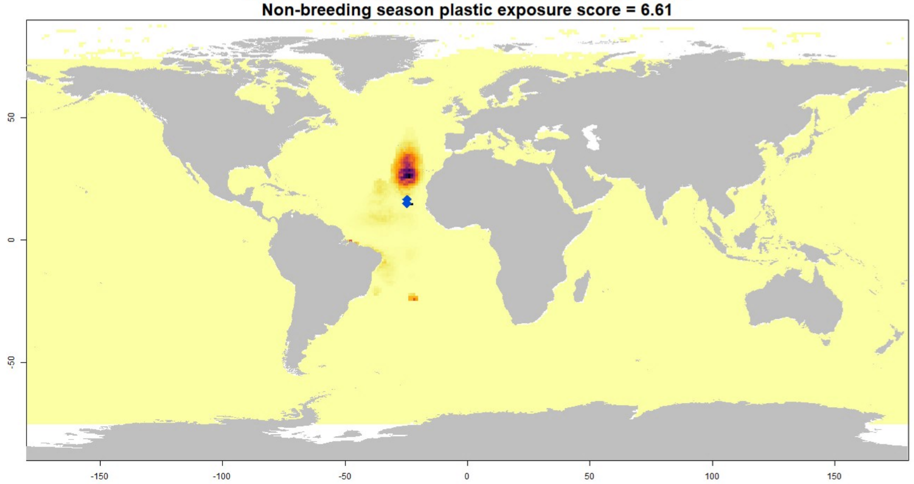

Audubon's Shearwater Puffinus lherminieri, Madeira
Non-breeding season plastic exposure score = 30.06

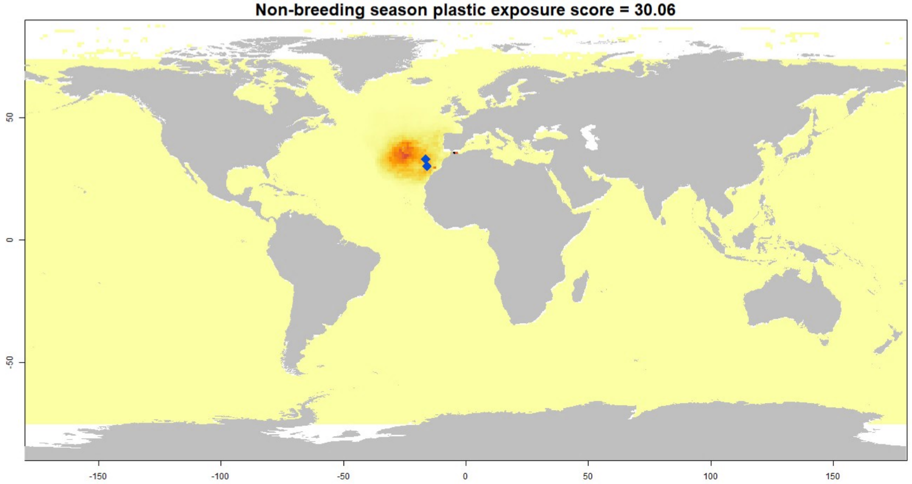

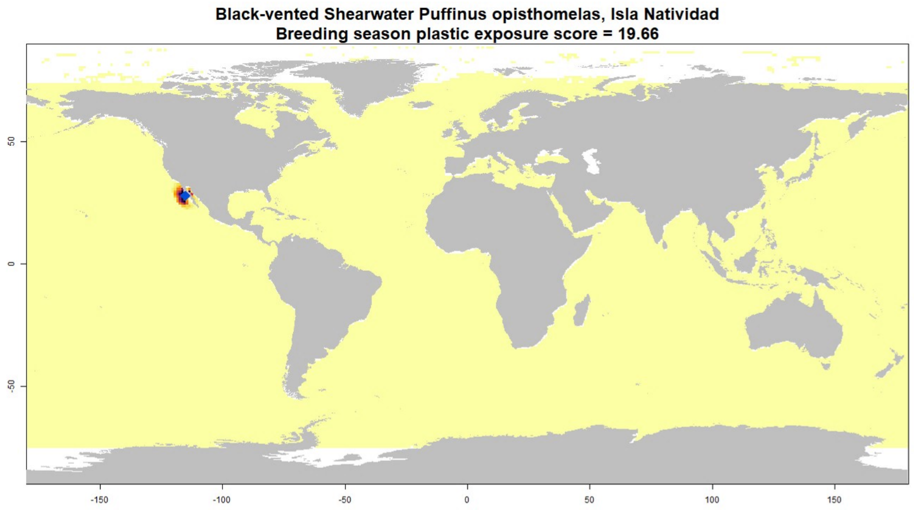
Black-vented Shearwater Puffinus opisthomelas, Isla Natividad
Breeding season plastic exposure score = 19.66
50
0
-50
-150
-100
-50
0
50
100
150

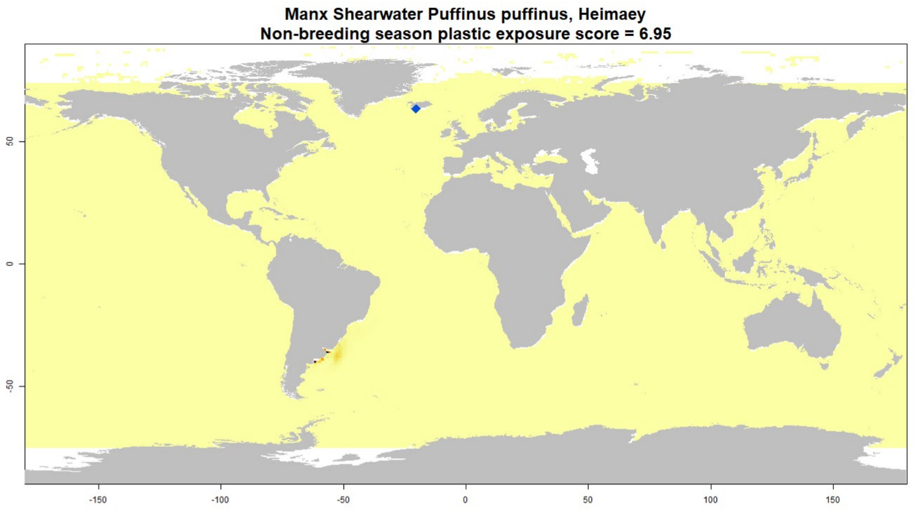
Manx Shearwater Puffinus puffinus, Heimaey
Non-breeding season plastic exposure score = 6.95
50
0
-50
-150
-100
-50
0
50
100
150

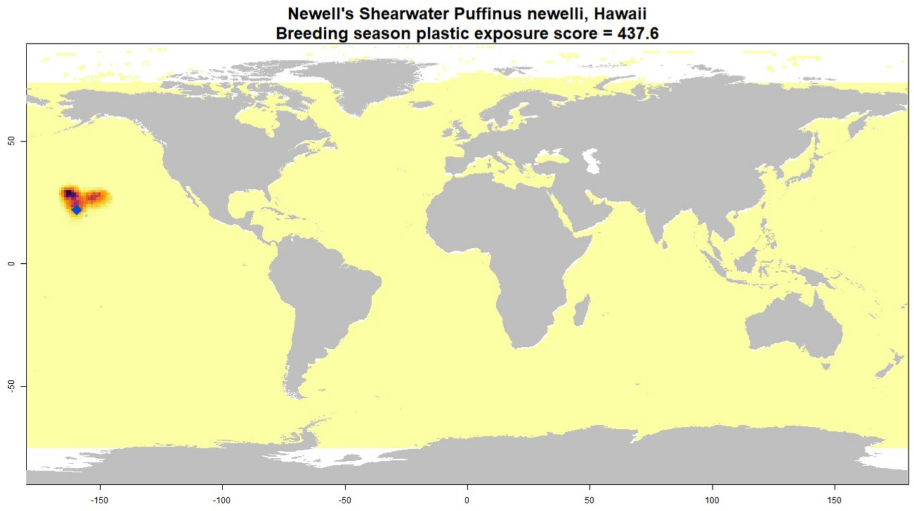
Newell's Shearwater Puffinus newelli, Hawaii
Breeding season plastic exposure score = 437.6
50
0
-50
-150
-100
-50
0
50
100
150

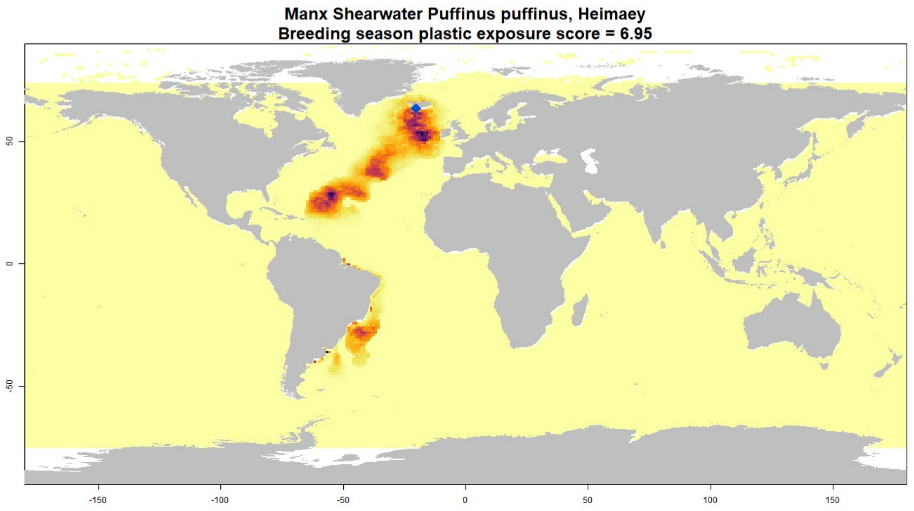
Manx Shearwater Puffinus puffinus, Heimaey
Breeding season plastic exposure score = 6.95
50
0
-50
-150
-100
-50
0
50
100
150

Manx Shearwater Puffinus puffinus, UK
Breeding season plastic exposure score = 43.02

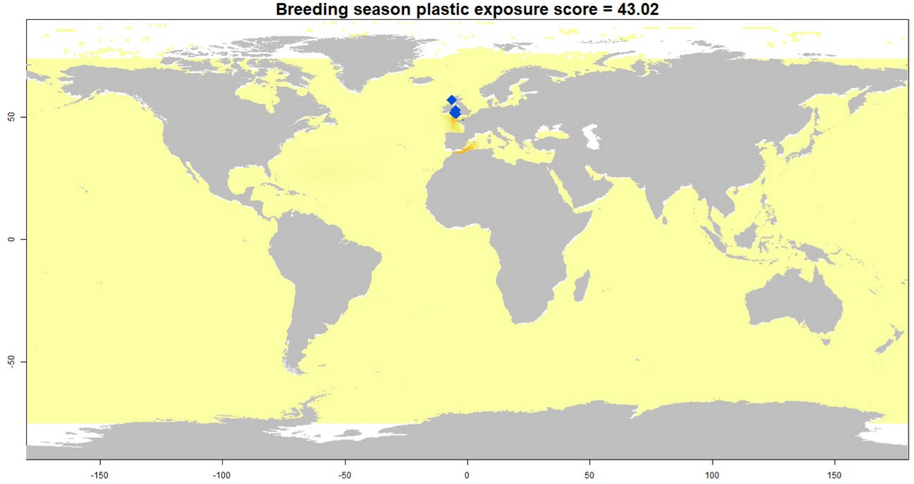

Yelkouan Shearwater Puffinus yelkouan, Iles Hyeres
Breeding season plastic exposure score = 2371.29

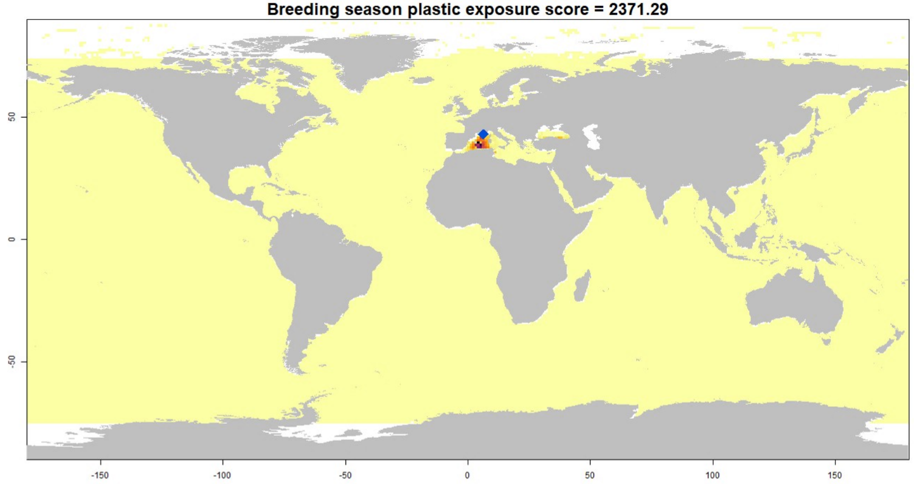

Manx Shearwater Puffinus puffinus, Ireland
Breeding season plastic exposure score = 5.53

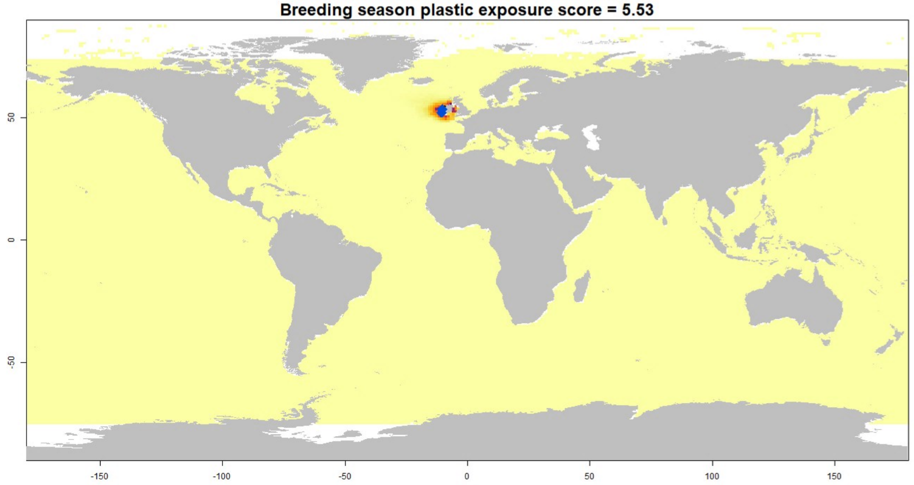

Manx Shearwater Puffinus puffinus, UK
Non-breeding season plastic exposure score = 43.02

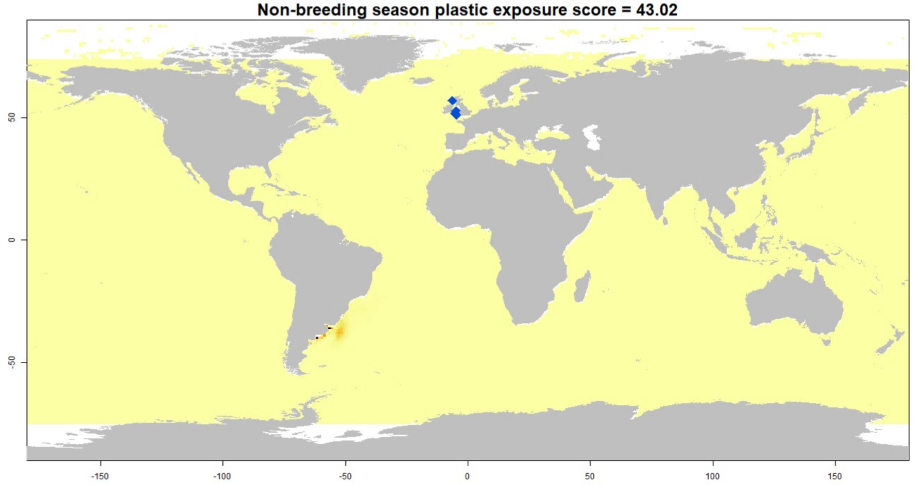

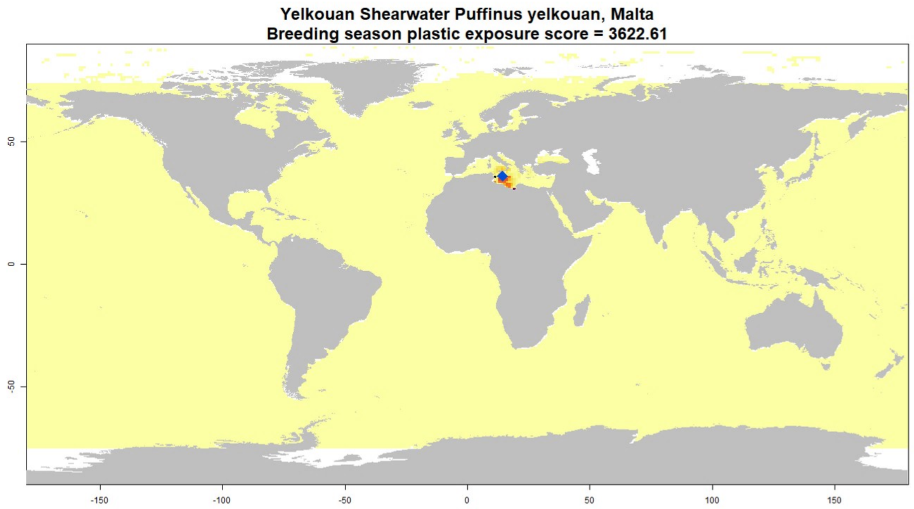
Yelkouan Shearwater Puffinus yelkouan, Malta
Breeding season plastic exposure score = 3622.61

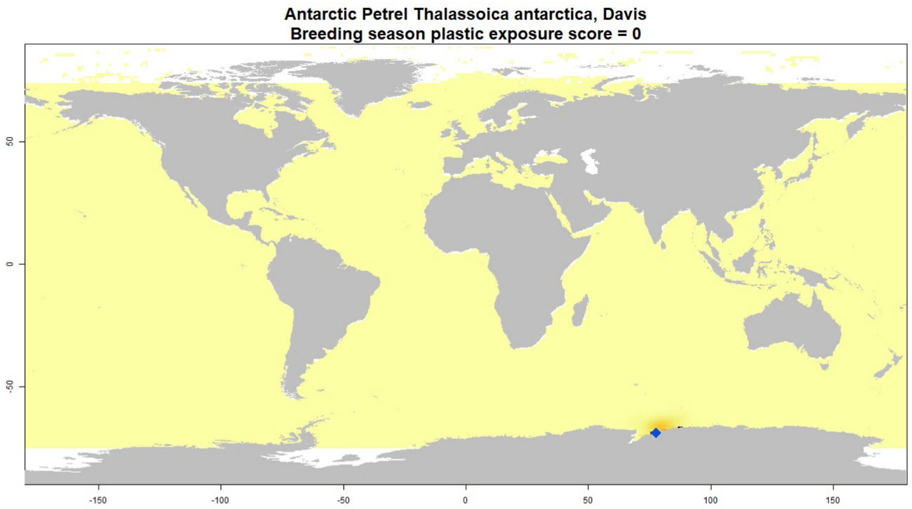
Antarctic Petrel Thalassoica antarctica, Davis
Breeding season plastic exposure score = 0

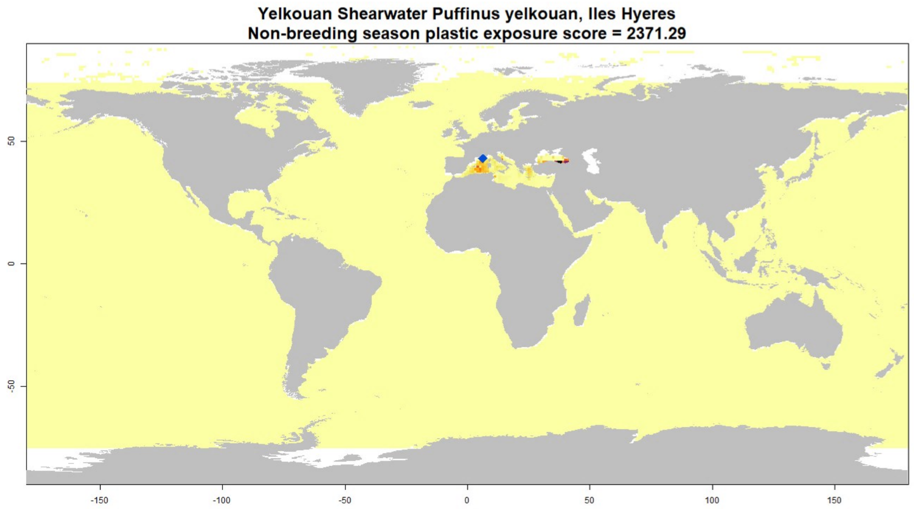
Yelkouan Shearwater Puffinus yelkouan, Iles Hyeres
Non-breeding season plastic exposure score = 2371.29

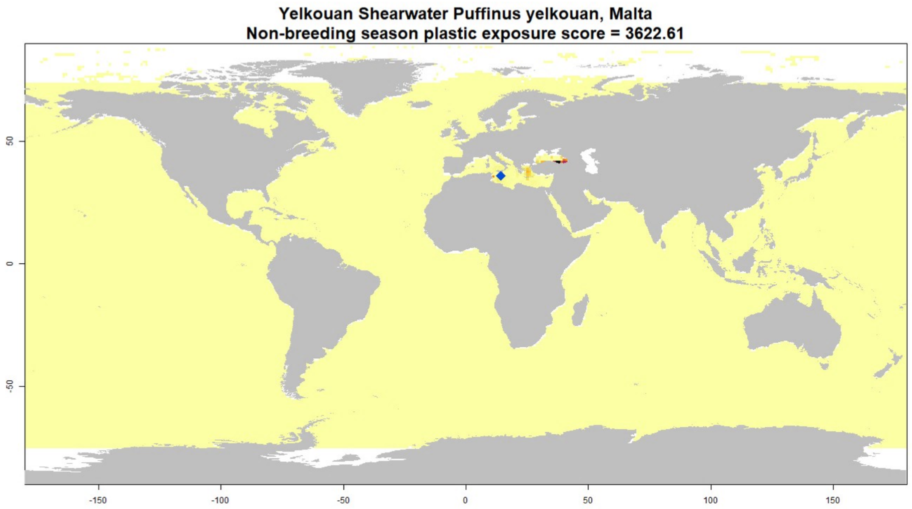
Yelkouan Shearwater Puffinus yelkouan, Malta
Non-breeding season plastic exposure score = 3622.61

Antarctic Petrel Thalassoica antarctica, Svarthamaren
Non-breeding season plastic exposure score = 0

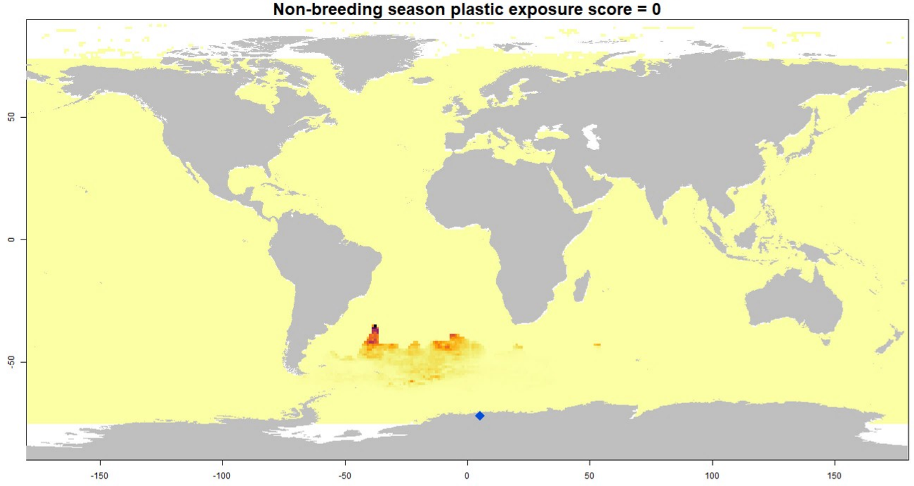

Buller's Shearwater Ardenna bulleri, Aorangi Island
Non-breeding season plastic exposure score = 22.56

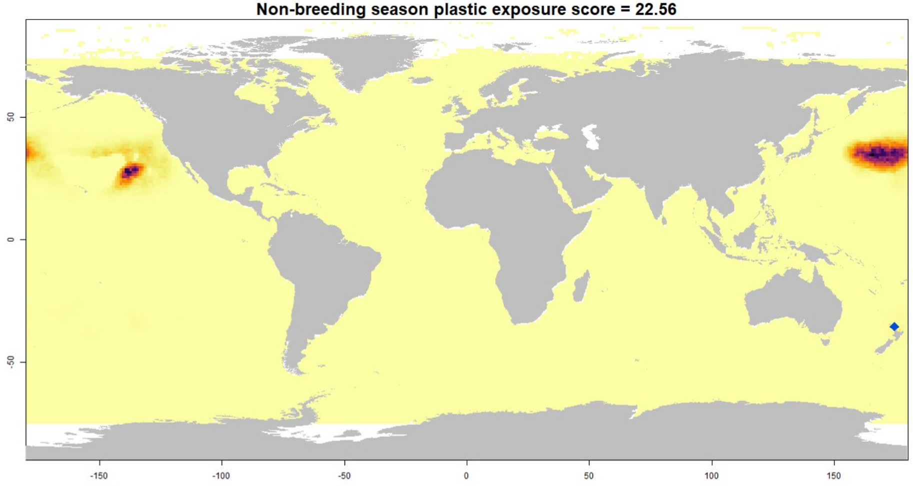

Antarctic Petrel Thalassoica antarctica, Svarthamaren
Breeding season plastic exposure score = 0

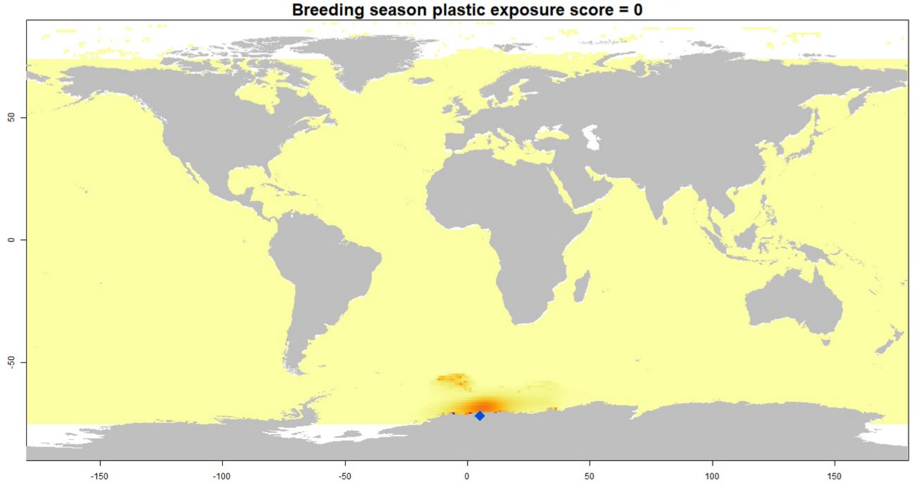

Buller's Shearwater Ardenna bulleri, Aorangi Island
Breeding season plastic exposure score = 22.56

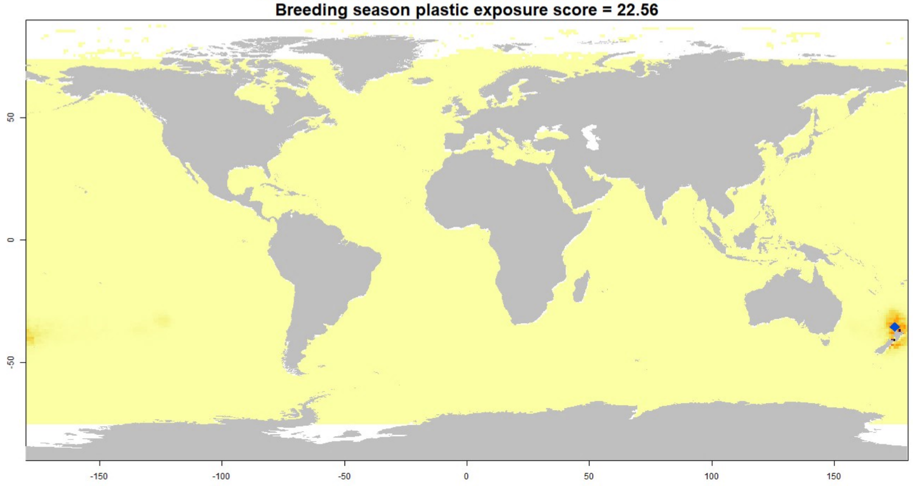

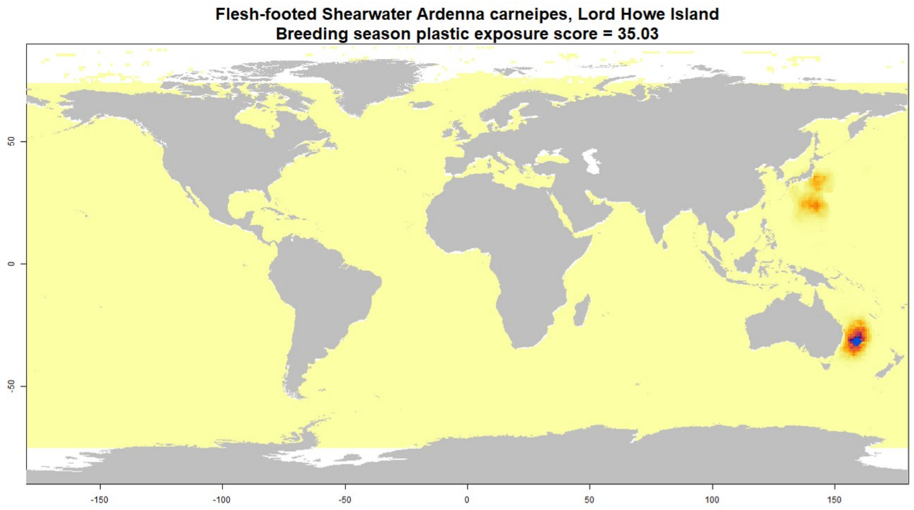
Flesh-footed Shearwater Ardenna carneipes, Lord Howe Island
Breeding season plastic exposure score = 35.03
50
0
-50
-150
-100
-50
0
50
100
150

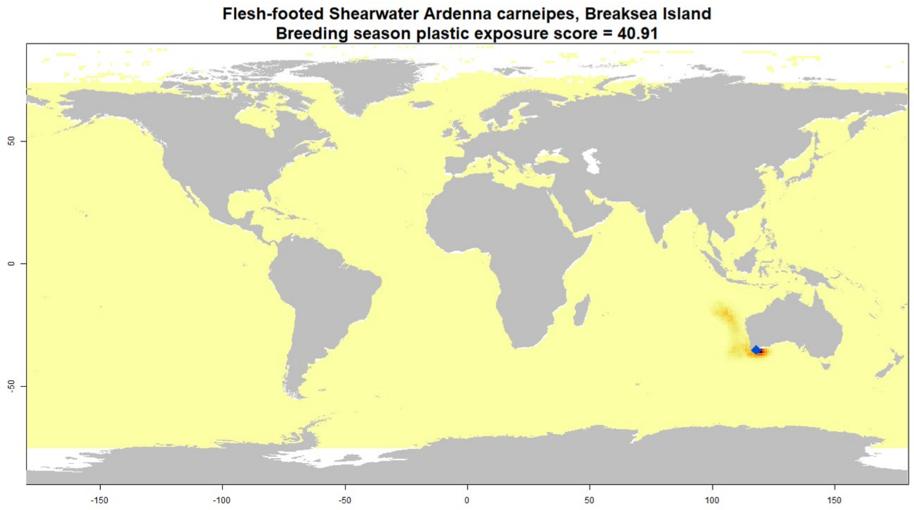
Flesh-footed Shearwater Ardenna carneipes, Breaksea Island
Breeding season plastic exposure score = 40.91
50
0
-50
-150
-100
-50
0
50
100
150
