# Supplementary material for: Global assessment of marine plastic exposure risk for oceanic birds
Source: Nat Commun. 2023 Jul 4;14:3665. doi: 10.1038/s41467-023-38900-z (PMC10319801; doi:10.1038/s41467-023-38900-z)
Supplement: Supplementary file 3 — Description of Additional Supplementary Files [file 41467_2023_38900_MOESM3_ESM.pdf]

Description of Additional Supplementary Files

Supplementary Data 1 Percentage of the plastic exposure risk for each tracked petrel species from each breeding country in each EEZ (theoretical EEZ in the Mediterranean) and the high seas accounting for more than 5% of the total.

Supplementary Data 2 Tracking data sample sizes for the number of individuals, locations, unique months, years and year ranges for each population.

Supplementary Data 3 Tracking data sample sizes for the number of individuals, locations and populations, list of populations and mean tracking year for each species.

Supplementary Data 4 Breeding schedules by month for species tracked in both the breeding and breeding seasons from published sources and from the method in this study using tracking data.

Supplementary Data 5 Plastic exposure risk scores for each population and breeding and non-breeding season.

Supplementary Data 6 Estimated population sizes for species with multiple tracked populations, or arithmetic means if only a maximum and minimum value were available.

Supplementary Data 7 Plastic exposure risk scores for each species, with populations weighted by population size and whether or not populations were tracked in both the breeding and non-breeding season.
