# Supplementary material for: Global assessment of marine plastic exposure risk for oceanic birds
Source: Nat Commun. 2023 Jul 4;14:3665. doi: 10.1038/s41467-023-38900-z (PMC10319801; doi:10.1038/s41467-023-38900-z)
Supplement: Supplementary file 11 — Reporting Summary [file 41467_2023_38900_MOESM11_ESM.pdf]

nature portfolio

Corresponding author(s): Bethany L Clark
Ana PB Carneiro
Elizabeth J Pearmain

Last updated by author(s): Mar 7, 2023

Reporting Summary

Nature Portfolio wishes to improve the reproducibility of the work that we publish. This form provides structure for consistency and transparency in reporting. For further information on Nature Portfolio policies, see our Editorial Policies and the Editorial Policy Checklist.

Statistics

For all statistical analyses, confirm that the following items are present in the figure legend, table legend, main text, or Methods section.

n/a	Confirmed	
☐	☒	The exact sample size (n) for each experimental group/condition, given as a discrete number and unit of measurement
☐	☒	A statement on whether measurements were taken from distinct samples or whether the same sample was measured repeatedly
☐	☒	The statistical test(s) used AND whether they are one- or two-sided<br>*Only common tests should be described solely by name; describe more complex techniques in the Methods section.*
☐	☒	A description of all covariates tested
☐	☒	A description of any assumptions or corrections, such as tests of normality and adjustment for multiple comparisons
☐	☒	A full description of the statistical parameters including central tendency (e.g. means) or other basic estimates (e.g. regression coefficient) AND variation (e.g. standard deviation) or associated estimates of uncertainty (e.g. confidence intervals)
☐	☒	For null hypothesis testing, the test statistic (e.g. F, t, r) with confidence intervals, effect sizes, degrees of freedom and P value noted<br>*Give P values as exact values whenever suitable.*
☒	☐	For Bayesian analysis, information on the choice of priors and Markov chain Monte Carlo settings
☒	☐	For hierarchical and complex designs, identification of the appropriate level for tests and full reporting of outcomes
☐	☒	Estimates of effect sizes (e.g. Cohen's d, Pearson's r), indicating how they were calculated

Our web collection on statistics for biologists contains articles on many of the points above.

Software and code

Policy information about availability of computer code

Data collection	No codes were used to collect data.
Data analysis	All analysis was carried out using R version 4.1.2, using the following packages: adehabitatHR v0.4.19, DHARMa v0.2.7, sp v1.3-2, rgdal v1.4-8, raster v3.1-5, rgeos v0.5-3, geosphere v1.5-14, tidyverse 1.3.2, RColorBrewer v1.1-2, stringr v1.4.0, trip v1.8.5, viridis v0.5.1, cowplot v1.1.1, sf v0.9-4, ggtext v0.1.1, geomerge v0.3.2, maptools v1.1-3, gridExtra v2.3. The R codes used to produce the analysis can be accessed at https://github.com/BirdLifeInternational/petrels-plastics and has been archived at https://doi.org/10.5281/zenodo.7852143.

For manuscripts utilizing custom algorithms or software that are central to the research but not yet described in published literature, software must be made available to editors and reviewers. We strongly encourage code deposition in a community repository (e.g. GitHub). See the Nature Portfolio guidelines for submitting code & software for further information.

Data

Policy information about availability of data

All manuscripts must include a data availability statement. This statement should provide the following information, where applicable:
- Accession codes, unique identifiers, or web links for publicly available datasets
- A description of any restrictions on data availability
- For clinical datasets or third party data, please ensure that the statement adheres to our policy

The plastic exposure risk data generated in this study and the plastic density data used in this study are provided in the Supplementary Data files, available at https://github.com/BirdLifeInternational/petrels-plastics and have been deposited in the Zenodo database at https://doi.org/10.5281/zenodo.7852143. The seabird

tracking data are available under restricted access because the data were collected for other purposes that vary between datasets and revealing the exact locations of sensitive species may put them at risk. Access can be obtained by making a request to the owners of each dataset using the mechanisms provided by each database. Zoatrack (https://zoatrack.org/) under dataset IDs: 57, 93, 102-112, 159, 253, 254, 762, 817. Movebank (https://www.movebank.org/) under dataset IDs: 944960474, 200628745. SEATRACK (https://seapop.no/en/seatrack/) for relevant northern fulmar data. Seabird Tracking Database (https://www.seabirdtracking.org/) under dataset IDs: 434, 438, 439, 448, 466, 467, 506–511, 517, 518, 554, 555, 561, 571, 607, 609, 610, 627, 628, 634, 635, 637, 639, 658, 659, 662, 663, 667, 668, 670, 672–678, 683, 684, 686, 694–696, 704–706, 708–715, 736, 741, 783–786, 788, 789, 826, 827, 829–831, 836–842, 844, 854, 858–872, 879, 883–886, 888–893, 900, 945, 946, 949, 951–954, 959–963, 966, 967, 970–983, 986–998, 1004, 1028, 1029, 1031–1033, 1055–1061, 1081, 1083, 1084, 1086–1091, 1120, 1121, 1140–1142, 1233–1236, 1238, 1239, 1258, 1259, 1279, 1280, 1282, 1285–1289, 1298, 1314, 1317, 1326, 1343–1347, 1360–1362, 1375, 1386, 1401, 1404, 1409, 1410, 1413–1415, 1422–1425, 1440, 1443, 1452, 1453, 1460, 1461, 1463, 1481, 1482, 1485–1488, 1494, 1497–1500, 1520–1523, 1541, 1544, 1546, 1549–1551, 1553–1558, 1562–1570, 1574–1577, 1579–1582, 1585–1592, 1599, 1600, 1602, 1603, 1618, 1619, 1621–1625, 1630, 1668–1672.

Human research participants

Policy information about studies involving human research participants and Sex and Gender in Research.

Reporting on sex and gender	No human research participants were involved in this study.
Population characteristics	*Describe the covariate-relevant population characteristics of the human research participants (e.g. age, genotypic information, past and current diagnosis and treatment categories). If you filled out the behavioural & social sciences study design questions and have nothing to add here, write "See above."*
Recruitment	*Describe how participants were recruited. Outline any potential self-selection bias or other biases that may be present and how these are likely to impact results.*
Ethics oversight	*Identify the organization(s) that approved the study protocol.*

Note that full information on the approval of the study protocol must also be provided in the manuscript.

Field-specific reporting

Please select the one below that is the best fit for your research. If you are not sure, read the appropriate sections before making your selection.

☐ Life sciences ☐ Behavioural & social sciences ☒ Ecological, evolutionary & environmental sciences

For a reference copy of the document with all sections, see nature.com/documents/nr-reporting-summary-flat.pdf

Ecological, evolutionary & environmental sciences study design

All studies must disclose on these points even when the disclosure is negative.

Study description	This study used tracking data to quantify the relative exposure to plastic for petrels on a global scale.
Research sample	We chose to sample petrel species (Oceanitidae, Hydrobatidae and Procellariidae) because this group is wide-ranging, highly threatened and particularly vulnerable to ingesting plastic. We excluded data for the two giant petrel species Macronectes giganteus and Macronectes halli because our analyses focused on marine areas and they regularly feed on land. To best represent the movement of tracked petrels, we requested data for all populations of species that we were aware had appropriate data. We collated 1,736,880 tracked locations (at 12-hourly intervals) for 7,137 individuals of 77 petrel species (64% of target species), from 148 populations in 27 countries plus Antarctica. The request to use the dataset for 1 tracked species was declined. This included only adult birds, including males and females. Data were collected to study natural behaviour in the wild, so no experiment manipulations were made to test the effect on animal movements.
Sampling strategy	No sample size calculation was performed. We did not test any hypotheses. We included all possible data sources to cover the largest possible sample. We believe this is sufficient and the sample size is similar to or larger than those found in published studies at the time of submission.
Data collection	We searched for all available datasets for our target species using relevant databases (Seabird Tracking Database (www.seabirdtracking.org), ZoaTrack (www.zoatrack.org) and Movebank (www.movebank.org)), scientific publications, conference presentations, social media and through personal contacts. We recorded information about each dataset in a spreadsheet and contacted data owners via database mechanisms or email. The datasets used in the study involved collecting the locations of petrels at sea using tracking devices attached to the birds. We collated tracking data collected using Global Positioning System (GPS) loggers, Platform Terminal Transmitters (PTTs) and Global Location Sensor (GLS) loggers deployed on adult petrels. Bird locations were recorded by the loggers, along with metadata recorded by researchers in the field (colony location, species, age, breeding stage).
Timing and spatial scale	Data were collated between 2020-03-03 and 2020-08-21. Tracking data was collected from 1995-08-11 to 2020-03-09. The spatial scale is global, but restricted to marine areas only.
Data exclusions	For Geolocator data, we removed locations around the equinoxes (March equinox: -21, +7 days; September equinox: -7, +21 days) which are unreliable, unless latitudes were estimated using additional information such as sea surface temperature prior to our analysis. For GPS and PTT data, we filtered the locations for unrealistic speeds (>90 km/h) and removed locations that were clear

outliers. We removed locations within 5 km of the colony for GPS data or within 15 km of the colony for PTT data, but not for GLS locations due to large location error for these devices. For each population, we pooled all locations for all individuals across all years by month, and then removed months with fewer than five locations. After calculating utilisation distributions, we trimmed all cells that fell over land and rescaled the remaining cells because these species do not forage in these environments and it is extremely rare for them to travel over land, so any locations are most likely due to device error.

Reproducibility	We did not conduct experiments and the results cannot be reproduced because they are based on the natural behaviour of wild individuals. The analytical methods can be reproduced using the code provided at https://github.com/BirdLifeInternational/petrels-plastics and https://doi.org/10.5281/zenodo.7852143.
Randomization	Individuals were randomly sampled in the wild from accessible populations.
Blinding	Blinding was not required because the data were collected by tracking devices. Blinding was not possible for the allocation of data collation due to the need to communicate with data owners. The analysis was conducted in an automated manner such that it is run using the same procedures for all datasets and blinding is not relevant.

Did the study involve field work? ☒ Yes ☐ No

Field work, collection and transport

Field conditions	Conditions varied across the 148 study populations. Information on location field conditions was not relevant to our study.
Location	The study sites are plotted in Fig 1a, and the latitudes and longitudes are provided in the supplementary materials.
Access & import/export	All access and permits were granted by the relevant parties at each study site. No import or export of samples was involved in this study.
Disturbance	There was disturbance at study sites for logger deployment and retrieval (if necessary). Standard steps for seabird tracking studies were taken to minimise disturbance. This particular study did not add additional disturbance because we only used data that were collected for other purposes.

Reporting for specific materials, systems and methods

We require information from authors about some types of materials, experimental systems and methods used in many studies. Here, indicate whether each material, system or method listed is relevant to your study. If you are not sure if a list item applies to your research, read the appropriate section before selecting a response.

Materials & experimental systems

n/a	Involved in the study	
☒	☐	Antibodies
☒	☐	Eukaryotic cell lines
☒	☐	Palaeontology and archaeology
☒	☐	Animals and other organisms
☒	☐	Clinical data
☒	☐	Dual use research of concern

Methods

n/a	Involved in the study	
☒	☐	ChIP-seq
☒	☐	Flow cytometry
☒	☐	MRI-based neuroimaging
