# Supplementary material for: Global assessment of marine plastic exposure risk for oceanic birds
Source: Nat Commun. 2023 Jul 4;14:3665. doi: 10.1038/s41467-023-38900-z (PMC10319801; doi:10.1038/s41467-023-38900-z)
Supplement: Supplementary file 2 — Peer Review File [file 41467_2023_38900_MOESM2_ESM.pdf]

nature portfolio

Peer Review File

Global assessment of marine plastic exposure risk for oceanic birds

Open Access This file is licensed under a Creative Commons Attribution 4.0 International License, which permits use, sharing, adaptation, distribution and reproduction in any medium or format, as long as you give appropriate credit to the original author(s) and the source, provide a link to the Creative Commons license, and indicate if changes were made. In the cases where the authors are anonymous, such as is the case for the reports of anonymous peer reviewers, author attribution should be to 'Anonymous Referee' followed by a clear attribution to the source work. The images or other third party material in this file are included in the article's Creative Commons license, unless indicated otherwise in a credit line to the material. If material is not included in the article's Creative Commons license and your intended use is not permitted by statutory regulation or exceeds the permitted use, you will need to obtain permission directly from the copyright holder. To view a copy of this license, visit http://creativecommons.org/licenses/by/4.0/.

Editorial Note: This manuscript has been previously reviewed at another journal that is not operating a transparent peer review scheme. This document only contains reviewer comments and rebuttal letters for versions considered at *Nature Communications*.

REVIEWERS' COMMENTS

Reviewer #4 (Remarks to the Author):

In Fig 4d the colours don't always go from dark to light, for example in the first row, Yelkouan shearwater in Malta, why is there a black bar after the gray bards moving left to right.

Also in Fig 4, there are two explanations for b in the figure legend, but nothing for c.

"We identified priority species for future research that would be useful bioindicators in each of these regions (Extended Data Table 4)" - what is this based on? There is a whole science and process around the selection of bioindicators. Suggest removing this section based on no methods given for the selection of bioindicators, and there are already several detailed conversations about plastics and indicators (GESAMP, several published papers with methodologies).

I think in general that the paper is improved with the edits, but I still think that it is weak in terms of in presenting novel conclusions, rather it builds on existing knowledge, and reinforces discussions for other sources in a unified model. I think an emphasis is needed on what is novel here, not just empirically, but theoretically - what is the new news from this paper? If it just reinforces existing ideas, that is fine, but this needs to be recognized and stated. Varying exposure to plastics by region and species is something that has been shown in many cases, and discussed in dozens of papers. Supporting these at the large scale is a valid piece of information, but formulating this as the purpose of the paper is a critical piece that is still missing.

One piece that is missing is the recognition of the missing data - for example the lack of data in the SE Asia is recognized, but the bird tracking data is also sparse in the region - so the interpretation of the maps is that this is not a high priority area -

Point by point responses to the reviewer's comments are provided below in blue. Line numbers refer to those in the tracked changes document.

REVIEWERS' COMMENTS

Reviewer #4 (Remarks to the Author):

In Fig 4d the colours don't always go from dark to light, for example in the first row, Yelkouan shearwater in Malta, why is there a black bar after the gray bards moving left to right.

Response: The colours in Fig 4c are set to match Fig 4b, see L375 "Bars are coloured according to b.", so the colours will not always go from dark to light as the colour represents the % across all species and the bars in c are for specific populations. To make this clearer, we have included an additional legend in the figure.

Also in Fig 4, there are two explanations for b in the figure legend, but nothing for c.

Response: The explanation was incorrectly labelled as "C", which we have corrected to "c".

"We identified priority species for future research that would be useful bioindicators in each of these regions (Extended Data Table 4)" - what is this based on? There is a whole science and process around the selection of bioindicators. Suggest removing this section based on no methods given for the selection of bioindicators, and there are already several detailed conversations about plastics and indicators (GESAMP, several published papers with methodologies).

Response: L640, we thank the reviewer for highlighting that this term is linked to a research field beyond the scope of our study. We have removed the text "that would be useful bioindicators", retaining the table to refer to priorities for future research as this applies to research in general, rather than specifically bioindicators.

I think in general that the paper is improved with the edits, but I still think that it is weak in terms of in presenting novel conclusions, rather it builds on existing knowledge, and reinforces discussions for other sources in a unified model. I think an emphasis is needed on what is novel here, not just empirically, but theoretically - what is the new news from this paper? If it just reinforces existing ideas, that is fine, but this needs to be recognized and stated. Varying exposure to plastics by region and species is something that has been shown in many cases, and discussed in dozens of papers. Supporting these at the large scale is a valid piece of information, but formulating this as the purpose of the paper is a critical piece that is still missing.

Response: Our study does reinforce existing ideas that plastic exposure risk varies by region and species. However, we also provide a great deal of new information by creating unified model using novel techniques and an unprecedented dataset. For example, exposure risk to be divided into breeding and non-breeding seasons, which are key to species populations. The regions of concern do not match those highlighted by the previous best risk assessment (Wilcox et al. 2015). This is because the previous study used range maps that only account for species presence, while we incorporate the relative importance of parts of the range indicated by the proportion of time the birds spend in area. Additionally, our tracking dataset includes locations outside the published range maps that were used in the previous risk assessment, where the species were not previously known to use.

One piece that is missing is the recognition of the missing data - for example the lack of data in the SE Asia is recognized, but the bird tracking data is also sparse in the region - so the interpretation of the maps is that this is not a high priority area –

Response: Please see L637 "Additionally, both petrel tracking and ingestion data are sparse in coastal waters around east and southeast Asia, where high plastic densities occur, and the South Pacific and North Atlantic gyres, where moderate plastic densities occur[8,15] (Fig. 1). We identified priority species for future research in each of these regions (Extended Data Table 4)."
